# Systems Analysis Unfolds the Relationship between the Phosphoketolase Pathway and Growth in *Aspergillus nidulans*

**DOI:** 10.1371/journal.pone.0003847

**Published:** 2008-12-04

**Authors:** Gianni Panagiotou, Mikael R. Andersen, Thomas Grotkjær, Torsten B. Regueira, Gerald Hofmann, Jens Nielsen, Lisbeth Olsson

**Affiliations:** 1 Center for Microbial Biotechnology, Department of Systems Biology, Technical University of Denmark, Lyngby, Denmark; 2 Fluxome Sciences A/S, Lyngby, Denmark; Newcastle University, United Kingdom

## Abstract

**Background:**

*Aspergillus nidulans* is an important model organism for studies on fundamental eukaryotic cell biology and on industrial processes due to its close relation to *A. niger* and *A. oryzae*. Here we identified the gene coding for a novel metabolic pathway in *A. nidulans*, namely the phosphoketolase pathway, and investigated the role of an increased phosphoketolase activity.

**Methodology/Principal Findings:**

Over-expression of the phosphoketolase gene (*phk*) improved the specific growth rate on xylose, glycerol and ethanol. Transcriptome analysis showed that a total of 1,222 genes were significantly affected by over-expression of the *phk*, while more than half of the affected genes were carbon source specific. During growth on glucose medium, the transcriptome analysis showed that the response to *phk* over-expression is targeted to neutralize the effect of the over-expression by regulating the acetate metabolism and initiate a growth dampening response.

**Conclusions/Significance:**

Metabolic flux analysis using ^13^C-labelled glucose, showed that over-expression of phosphoketolase added flexibility to the central metabolism. Our findings further suggests that *A. nidulans* is not optimized for growth on xylose, glycerol or ethanol as the sole carbon sources.

## Introduction

Filamentous fungi are prominent users of the sugar xylose, but currently little work has been devoted to study the metabolism of this sugar. This is in sharp contrast to the large and continued commercial interest for xylose utilization in bioethanol production and as cheap raw material in industrial fermentations.

It is known that xylose enters the pentose phosphate (PP) pathway, but it is not clear how it interacts with the remaining part of the metabolism. Phosphoketolases (EC 4.1.2.9, EC 4.1.2.22) are a class of key enzymes of the phosphoketolase pathway of heterofermentative and facultatively homofermentative lactic acid bacteria, as well as the D-fructose 6-phosphate shunt of bifidobacteria, and xylose fermenting yeast [1], [2]. The first type of phosphoketolase (PHK, EC 4.1.2.9) catalyses an irreversible thiamine diphosphate dependent phosphorolytic reaction: in the presence of inorganic phosphate, D-xylulose-5-phosphate is cleaved into acetyl phosphate and glyceraldehyde-3-phosphate. The study of this PHK has been hampered by the fact that the substrate D-xylulose-5-phosphate initially was expensive, and since the beginning of 2001 it has been withdrawn from the commercial market.

Recently, our group [3], [4] provided evidence of the PHK pathway existence in *Aspergillus nidulans*, which could be induced under certain cultivation conditions. Induction of PHK in Aspergilli increases the carbon flux towards the precursors acetyl-CoA or acetate, and consequently there is a potential to improve the yields and productivities of a long series of industrially important secondary metabolites derived from acetyl-CoA. For a comprehensive evaluation of genes that are transcriptionally modulated during growth in the presence of an active PHK pathway, we constructed a strain that constitutively expressed the gene putatively encoding PHK (*phk*, AN4913.3) and performed a large-scale analysis of gene expression in *A. nidulans* by using DNA microarrays. Despite the wide use of DNA microarrays, only a few studies have been reported on transcriptome profiling in *Aspergilli*, e.g. *A. oryzae* [5], *A. flavus* [6]–[8], *A. nidulans* [9]–[11].

In order to accurately determine the effects of over-expressing *phk* on transcription, we grew the cells on a broad range of carbon sources (glucose, xylose, glycerol and ethanol) in well controlled bioreactors. To further understand the effects on glucose, we performed labelling experiments with [1-^13^C] glucose to quantify the metabolic fluxes in the central carbon metabolism. Quantification of metabolic fluxes is one of the most direct measures on operation of metabolic networks, and it is especially valuable in connection with studies of metabolite production where the aim is to direct carbon from the substrate into the metabolic product, e.g. secondary metabolites [12].

## Results

### Construction of the recombinant strain over-expressing *phk*

An *A. nidulans* mutant, which constitutively over-expresses *phk* (AR1*phk*GP74), was constructed. The vector that contained the corresponding gene harboured the strong constitutive glycolytic promoter from glyceraldehyde-3-phosphate dehydrogenase (*gpdA*) of *A. nidulans*. Northern blot analysis clearly indicated a strong over-expression of *phk* in the transformant compared to the reference strain (data not shown). To further verify that the over-expressed gene actually is coding for PHK, the wild type (A4) and the mutant (AR1*phk*GP74) strains were cultivated on glucose in the presence of 1 mM of iodo acetate, a specific and strong inhibitor of glyceraldehyde-3-phosphate dehydrogenase [13]. While iodo acetate prohibited growth of the A4 cells (specific growth rate of approximately 0.02 h^−1^) it had only a minor effect on the specific growth rate of the AR1*phk*GP74 mutant (specific growth rate of 0.19 h^−1^). However, growth of the AR1*phk*GP74 mutant ceased after consumption of half the carbon source, probably due to accumulation of glyceraldehyde-3 phosphate or drain of intracellular free phosphate.

### Functional significance of phosphoketolase in *A. nidulans*

A set of experiments was carried out to investigate the metabolism of the *A. nidulans* wild type (A4) and the *A. nidulans* AR1*phk*GP74 strains on various carbon sources (glucose, xylose, glycerol and ethanol). Substrate and product concentrations in the medium were determined for all cultivations as well as the maximum specific growth rate and the biomass yield on carbon source during the fully aerobic growth phase (Table 1). When glucose was used as carbon source, the A4 strain and the AR1*phk*GP74 strain had similar maximum specific growth rates and biomass yields on glucose (Y_sx_). In contrast, over-expression of *phk* significantly increased the specific growth rate and Y_sx_ to a lesser extent when the cells were cultivated on xylose and glycerol. In the cultivations on ethanol, over-expression of *phk* surprisingly led to a significant increase in both the specific growth rate and Y_sx_. These results show that over-expression of *phk* significantly improves growth performance on all substrates except from glucose. Growth performance was not expected to be improved on the C-2 compound ethanol since acetyl-CoA is formed as an intermediate during assimilation without the use of phosphoketolase.

**Table 1:** Specific growth rates and biomass yields of *A. nidulans* A4 (wild type) and AR1*phk*GP74 (mutant) cultivated in four different carbon sources.

Carbon source	µ (h^−1^)	Ysx (g g^−1^)
	Aspergillus nidulans A4/AR1*phk*GP74	Aspergillus nidulans A4/AR1*phk*GP74
glucose	0.23/0.23	0.47/0.48
xylose	0.16/0.19	0.46/0.47
glycerol	0.11/0.14	0.42/0.44
ethanol	0.12/0.17	0.23/0.35

The standard deviation was below 3% in all cultivations (triplicates).

### *In vivo* characterization using metabolic network analysis

**Figure 1:**
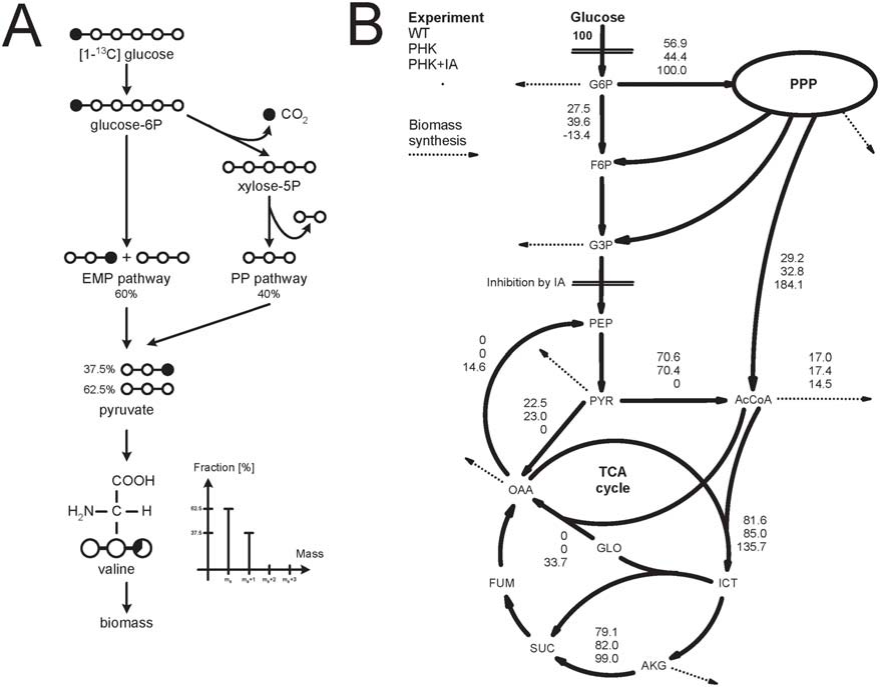
A) Principles of metabolic network analysis with [1-^13^C] glucose. The ^13^C labelling pattern of pyruvate is dependent on the active metabolic pathways. For instance, pyruvate formed in the EMP pathway contains ^13^C carbon in position 3, while activity of the PP pathway results in loss of labelled carbon. Pyruvate is the precursor of valine, which is incorporated into biomass. Based on the labelling pattern of valine and other proteinogenic amino acids the fluxes in the central carbon metabolism can be resolved. B) Major metabolic fluxes of the *A. nidulans* strains investigated. The two strains, A4 (WT) and AR1*phk*GP74 (PHK). IA indicates the presence of iodo acetate in the cultivation medium. All fluxes are relative to a glucose uptake rate of 100 moles (arbitrary number).

The impact of phosphoketolase in glucose cultivations was further studied with ^13^C labelling experiments in three conditions: strains A4, AR1*phk*GP74, and AR1*phk*GP74 in the presence of the glyceraldehyde-3-phosphate dehydrogenase inhibitor iodo acetate (1 mM). The mass spectra obtained from the GC-MS analysis of the hydrolysed biomass were converted into summed fractional labellings (SFL) (Supplementary Table S1). The SFL of a molecule or a fragment hereof reflects the enrichment of ^13^C, i.e. the enrichment of ^13^C in each carbon atom in a given fragment. For instance, if a fragment contains three carbon atoms which each contain 20% ^13^C and 80% ^12^C then the corresponding SFL is 60% (Figure 1A). It can be observed that addition of iodo acetate results in a significantly lower enrichment of ^13^C in the fragments. This observation can be explained by a higher flux through the PP pathway, which results in a direct and significant loss of ^13^C labelled carbon dioxide and formation of 2 moles NADPH. Consequently, the remaining fragments will not be enriched by ^13^C to the same degree. When iodo acetate is added and blocks the lower part of the Embden-Meyerhoff-Parnas (EMP) pathway, an efficient PHK and glyoxylate cycle are required for growth. All metabolites in the lower part of the EMP pathway and the TCA cycle must be formed from acetyl-CoA, and consequently the glyoxylate cycle is needed to form C-4 compounds from acetyl-CoA.

Using the SFL data, we estimated the fluxes in the central carbon metabolism by considering a simple non-compartmentalised metabolic model of *A. nidulans* with the EMP pathway, the PP pathway and the TCA cycle. We also estimated the fluxes with a more detailed metabolic model which included compartmentalisation of acetyl-CoA, pyruvate and oxaloacetate. However, the SFL data were only slightly better fitted to the larger model. In order to avoid over-fitting of the limited number of data points, we chose the simpler but more robust version of the two models (Supplementary Table S2, S3). The measured and simulated SFLs are listed in the Suppl. Table. S1, while major fluxes are depicted in Figure 1B. In general, a good fit between measured and calculated SFL, were found though the fragments Asp188 and Thr175 had a higher deviation (<15%) between measured and simulated fragments in all experiments. This discrepancy may be explained by the simplicity of the model since oxaloacetate, the precursor of Asp188 and Thr175, is not compartmentalised. However, the value of these fragments only had minor impact on the calculated flux distribution (simulation results not shown).

The simulation results showed that the wild type strain (A4) had a relative flux into the oxidative branch of the PP pathway of 56.9 compared to 44.4 when *phk* was over-expressed (Figure 1). Despite a reduction of the flux into the oxidative branch of the PP pathway, the flux through PHK remained largely constant (29.2 vs. 32.8 when *phk* was over-expressed). Since the specific growth rate, as well as the biomass yield on glucose, was similar for the two strains, there must be an alternative source of NADPH production in the strain with over-expressed *phk* (AR1*phk*GP74). This may be achieved through a switch in isoenzyme preference, e.g. the use of NADP-dependent isocitrate dehydrogenase rather than the corresponding NAD-dependent enzyme or a higher flux through malic enzyme. In either case, the results demonstrate that this *A. nidulans* strain has a high degree of metabolic flexibility with respect to fluxes in the central carbon metabolism. It is likely that over-expression of *phk* enhances this metabolic flexibility as it opens for an alternative route from sugars to C-2 and C-3 compounds. The metabolic network analysis demonstrates that NADPH mainly is provided through the PP pathway, but there are alternative sources.

Addition of iodo acetate to the medium had a dramatic impact on the fluxes for the strain over-expressing *phk*, and consequently a large deviation from the typical flux distribution was anticipated. Initial simulations demonstrated that the flux through glyceraldehyde-3-phosphate dehydrogenase was negligible and could be omitted (results not shown). When glycolysis is blocked, acetyl-CoA must be formed exclusively through PHK, and consequently is a six-fold increase in the flux expected. The flux through the TCA cycle increased from 82 to 99 and the glyoxylate cycle was very active (Figure 1). As mentioned in previous section, growth ceased after consumption of half the carbon source, and consequently the specific growth rate and the biomass yield could not be determined accurately. However, a lower biomass yield was expected since inhibition of glyceraldehyde 3-phosphate dehydrogenase blocks the lower part of glycolysis and results in low ATP production and accumulation of glyceraldehyde-3-phosphate.

In conclusion, with over-expression of *phk* it is possible to obtain quite high conversion yields of sugars to secondary metabolites originating from acetyl-CoA at the expense of a lower flux through glycolysis (either with chemical or genetic inhibition).

### Transcriptome analysis

**Figure 2:**
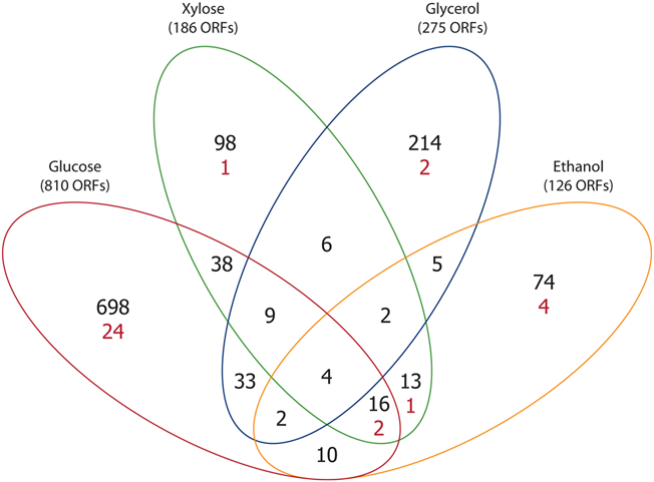
Venn diagram of genes with significant differential regulation in comparison of growth of *A. nidulans* A4 and phosphoketolase (PHK) over-expressing *A. nidulans* AR1*phk*GP74 on four different carbon sources. The over-expressed phosphoketolase, and argB, the transformation marker, are considered to be artefacts and were removed from the central group. Black numbers are the number of differentially regulated genes within each subgroup. Red numbers (below if present) are the number of putative transcription factors identified in that group.

The strains *A. nidulans* A4 and AR1*phk*GP74 were grown on the four carbon (glucose, xylose, glycerol or ethanol) sources in triplicates. From each of the 24 cultivations samples were taken for transcriptome analysis. Statistical analysis of the data was done in order to identify genes that exhibited different expression between the two strains in the four conditions. Figure 2 reveals that surprisingly few genes shared regulation between the sources of carbon when *phk* was over-expressed. A total of 1,222 unique genes were affected by the up-regulation of *phk*. Only four genes were significant on all four carbon-sources (AN0158.3, AN2165.3, AN2555.3 and AN6798.3), and only 29 genes appeared to be significant between three carbon sources. Indeed, for all carbon sources more than 50% of the regulated genes are specific to that carbon source. On the glucose medium, this percentage is as high as 86%. It is thus apparent that the response to the over-expression of *phk*, is highly dependent on the carbon source used for growth with glucose having the highest number of responding genes.

Analysis of the 1,222 regulated genes revealed several interesting facts on the response to the *phk*-over-expression. The *phk*-over-expressing strain had an increased specific growth rate and Y_sx_ compared to the wild type during growth on xylose, glycerol and ethanol. For this reason, one might expect to see a common response between those three sets of experiments. However, these three comparisons only share significant regulation of two genes, both of them being putative dehydrogenases. No common response coordinating the increase in biomass on these carbon sources can therefore be elucidated based on transcriptome data. When examining significantly changed expression level of putative transcription factors (Figure 2), it is apparent that these are mostly found in the genes unique to the four carbon sources. This supports that the PHK-response is to a very high degree carbon-source specific.

An examination of the expression indices of *phk* in the wild type strain on the four carbon sources showed that it is, indeed active, and mostly so on glucose and glycerol.

Among the genes showing significant differential regulation only on glucose, 24 were transcription factors (22 of them putative) and nine genes were putatively involved in cell growth or cell cycle regulation. The two characterized transcription factors are BrlA, a transcriptional regulator involved in sporulation, inducing the cessation of vegetative growth [14], and CreA, a global carbon repressor [15]. This result suggests that the increased biomass-production on xylose, glycerol and ethanol is in fact not induced by regulation on these media, but rather that *phk* over-expression directly increases the specific growth rate and Y_sx_, and this effect is repressed on glucose by a complex array of regulatory factors.

**Figure 3:**
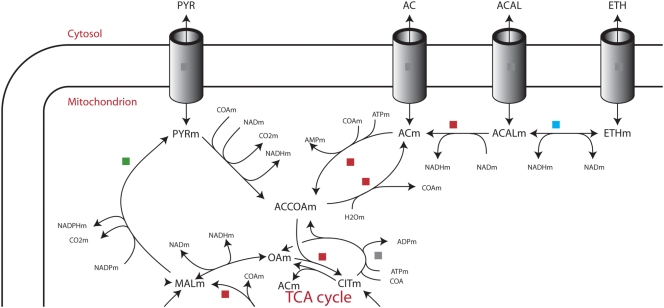
Response of central metabolism to phosphoketolase over-expression during growth on a glucose medium. A green box means that one or more isoenzymes of this reaction is down-regulated in the phosphoketolase over-expressing strain relative to the wild type. A red box indicates up-regulation. A blue box shows that some genes with this function are up-regulated while others are down-regulated. A grey box indicates that a gene could not be assigned to this function. The map was adapted from a map of *A. niger* metabolism published by Andersen et al [31].

To further investigate the effect of over-expressing *phk* on metabolism, we examined differentially expressed metabolic genes (Supplementary Figure S1, S2, S3, S4). A pathway analysis of all significantly expressed genes (p<0.05) showed the metabolic response to be highly carbon-source dependent. On glycerol and ethanol, no differential regulation is found in the central metabolism (with the exception of one step in the GABA shunt on glycerol). Over-expression of *phk* on xylose shows regulation around acetaldehyde (connecting pyruvate and ethanol). On glucose, scattered response was found throughout metabolism, including glycolysis, TCA and C_5_ metabolism, but an especially strong response was found in all steps of the C-2 metabolism pathways connecting acetyl-CoA, acetate, acetaldehyde and ethanol, suggesting strong regulation of the levels of these metabolites (Figure 3). To validate the results statistically, and eliminate the possibility of simply seeing an effect of the higher number of regulated genes in the glucose comparison, we employed the statistical reporter metabolite algorithm [16], [17] (Supplementary Table S4, S5, S6, S7). The algorithm identified acetaldehyde and ethanol to be highly significant nodes (among the ten most regulated metabolites, p<0.01) when grown on glucose (acetyl-CoA was not found as a significant reporter metabolite even though all reactions connecting it to the TCA were induced on glucose, since it is involved in a large number of fatty acid reactions with no significant regulation). Regulation around acetaldehyde and ethanol was only observed to a small extent in the gene expression data from growth on xylose medium (among the 60 most regulated metabolites, p<0.05), and not at all on glycerol (p>0.70) and ethanol medium (p>0.97). Since we do not observe increased fluxes around acetyl CoA in the labelling study (Figure 1B), we hypothesise the glucose repression response as having the purpose of homeostasis of acetyl CoA in spite of *phk*-over-expression.

## Discussion

To the best of our knowledge, we provide the first evidence that the phosphoketolase pathway plays a significant role in the central carbon metabolism of filamentous fungi. The transcriptome analysis showed that *phk* was expressed in the wild type cultivations on all carbon sources and mostly so on glucose and glycerol. To determine the role of the phosphoketolase in *A. nidulans* cells, we integrated the gene into the genome under a strong constitutive promoter. Interestingly, this mutant grew with a higher specific growth rate on xylose, glycerol and ethanol compared to the wild type, while over-expression of *phk* had no effect on the growth rate on glucose. Faster growth was also followed by a significant higher biomass yield in the mutant compared to the wild type strain. In the PP/glycolytic pathway, 5 mol of ATP are produced in the conversion of 3 moles of xylulose-5-P to 5 moles of pyruvate. Therefore the ATP yield per 1 mol of xylulose-5-P is 1.67 moles. On the other hand, in the cleavage of 1 mol of xylulose-5-P to pyruvate and acetyl-P by the phosphoketolase pathway, 2 mol of ATP are produced and 1 mol is further produced by the conversion of acetyl-P to acetate. Looking more into the total stoichiometry from xylulose-5-P to acetyl-CoA it is obvious that the PHK pathway is an energetically efficient route leading to improved specific growth rates on xylose, glycerol and ethanol.

3 X5P→5 PYR+5 NADH+5 ATP→5 Ac-CoA+5 CO_2_+5 NADH+5 ATP [PPP+Glycolysis]

3 X5P→3 PYR+3 Ac-CoA+3 NADH+6 ATP→6 Ac-CoA+3 CO_2_+3 NADH+6 ATP [Phosphoketolase]

More interesting is that the less favorable the carbon source is for growth of *A. nidulans* cells (lower biomass yields), the more profound is the effect of over-expressing *phk* on the specific growth rate (Table 1). Consequently, this strategy is important for processes where a less energetic carbon source than glucose is needed. However, the question still remains, why over-expression of *phk* had no effect on the specific growth rate on glucose. While during growth on ethanol the TCA cycle is very active making the extra acetyl-CoA an important source of energy (1 mol of acetyl-CoA yields 4 mol of NADH), the same effect is not expected when cells are growing on glucose. Furthermore, Panagiotou et al [18] have demonstrated that a balance between ATP and the ratio of the redox cofactors NADH/NADPH is crucial for improving the specific growth rate on glucose. In that study, over-expression of a NADH kinase led to a 1.2-fold improvement of the specific growth rate; this large increase was rather surprising since the NADH kinase is catalyzing an ATP dependent reaction, meaning that over-expression of this enzyme is associated with cost of energy. Bridging the two studies, it seems that the specific growth rate of *A. nidulans* on glucose is not limited by ATP and during growth on glucose there is already an efficient supply of all necessary precursors for biomass production and therefore inducing the phosphoketolase pathway does therefore not have a positive effect.

The above hypothesis was confirmed by the transcriptome analysis, where the response to *phk* over-expression on the glucose medium seems targeted to neutralize the effect by regulating the acetate metabolism and initiating a growth dampening response. On the other three substrates, we observed that the induction of the PHK pathway increases growth without initiating inhibiting responses, thus supporting that it is a pathway that adds flexibility to the central metabolism as observed in the flux study. It also suggests that *A. nidulans* is not optimized for growth on glycerol or ethanol as sole carbon sources. As these carbon sources, and especially glycerol and ethanol, are seldom found alone in the dead biomass that the saphrophytic *A. nidulans* grows on in nature, it is not surprising that the cells have not been evolved to grow fast on these carbon sources. It also suggests that production strains that grow faster on these substrates can be evolved or constructed by *phk* over-expression. The major difference between growth in nature and growth in aerobic, stirred bioreactors is probably that oxygen can be taken up efficiently in the bioreactor, and consequently respiration is expected to be much more efficient in our experiments compared to nature. If this is the case then less carbon is needed for ATP generation and instead carbon can be used for synthesis of biomass. When *A. nidulans* is growing on xylose, a pentose sugar, then PHK offers a direct way of generating cytosolic acetyl-CoA. When ATP generation is efficient, then PHK is an efficient way of generating acetyl-CoA since the route via pyruvate decarboxylase results in loss of carbon and a corresponding lower biomass yield. There is no requirement for an efficient PHK when growing on glucose.

## Materials and Methods

### Strain construction and plasmids

*Aspergillus nidulans* A4 was used as wild type reference strain. *Aspergillus nidulans* AR1 [*pyrG89*; *argB2*; *veA1*], IBT 27263, was used for the transformation. In order to over-express *phk* in *A. nidulans* AR1, the gene was cloned into an integrative vector derived from pBARGPE1 (M.L. Pall, J.P. Brunelli http://www.fgsc.net/fgn/pall.html), obtained from the FGSC, USA. For the construction of the plasmid, standard molecular biotechnology techniques were applied according to Sambrook et al. [19] and the final construct was verified by sequencing. *A. nidulans phk* (XP_662517) was amplified from genomic DNA with PCR using the Fphk_FW (5′-CGAGTCGGCCGGCCATGCCAGGAGAAGTCATCG-3′) and Fphk_RV (5′-TTGGCGCGCCCTAGCTCCAGTTTCCATCTGC-3′) primers, which introduced *Fse*I and *Asc*I restriction sites. The resulting strain was named AR1*phk*GP74 (over-expressing *phk*).

### Media

Minimal medium (MM) for transformation: 10 g/L glucose; 10 mM NaNO_3_; 0.52 g/L KCL; 0.52 g/L MgSO_4_; 1.52 g/L KH_2_PO_4_; 4·10^−4^ g/L CuSO_4_·5H_2_O; 4·10^−5^ g/L Na_2_B_2_O_7_·10H_2_O; 8·10^−4^ g/L FeSO_4_·7H_2_O; 8·10^−4^ g/L MnSO_4_·2H_2_O; 8·10^−4^ g/L Na_2_MoO_4_·2H_2_O; 8·10^−4^ g/L ZnSO_4_·7H_2_O. Arginine (4 mM) was supplemented in the MM used for the selection of transformants.

For bioreactor cultivations a previously described defined medium was used [3]. Carbon sources used were glucose, xylose, glycerol and ethanol (20 g l^−1^). Arginine, 0.7 g/L, was added to all bioreactor cultivations by sterile filtration.

### Transformation of *A. nidulans*

Genetic transformation of *A. nidulans* protoplasts was performed as previously described [20], except that protoplasting was achieved by using the enzyme, Glucanex, (Novozymes A/S), at a concentration of 40 mg/ml in protoplasting buffer. *Pvu*I was used for linearization of the plasmid as only one restriction site is present in the vector and which is located in the ampicillin resistance gene.

Transformants were purified by streaking out spores to obtain single colonies on selective minimal medium and incubated at 37°C for 3–4 days. The resulting recombinants were further purified twice by streaking out spores on fresh plates with selective medium.

### Cultivation conditions and analysis of substrates and products

To determine the physiological characteristics as well as for sampling for gene expression analysis, cultivations were performed in well-controlled 1.5 l bioreactors with a working volume of 1.2 l. The bioreactors were equipped with two disc-turbine impellers rotating at 350 rpm and sparged with air at a constant flow rate of 1.0 vvm (volume of gas per volume of liquid per minute). The pH was kept constant at 5.5 by addition of 2 M NaOH or HCl and the temperature was maintained at 30°C. For selected experiments, iodo acetate was added by sterile filtration to the sterilized growth medium giving a final concentration of 1 mM.

Cell dry weight was determined using nitrocellulose filters (pore size 0.45 µm, Gelman Sciences). Fermentation samples were immediately filtered and stored at −20°C until analysis. The concentrations of glucose, xylose, glycerol, acetate, succinate, and pyruvate were determined by HPLC as described previously [3].

### Analysis of fractional ^13^C enrichments and computational methods

For the ^13^C labelling experiments *A. nidulans* A4 and AR1*phk*GP74, respectively, were cultivated with 5 g/L of [1-^13^C] glucose and an aeration rate of 1.0 vvm. In the late exponential phase, biomass samples from the cultivations were harvested, hydrolyzed, derivatized and analyzed by gas GC-MS for determination of labelling patterns of intracellular metabolites. All fragments were derived from either glucose-6-phosphate or proteinogenic amino acids [21].

#### Calculation of drain fluxes for biomass formation and metabolic model

The biomass composition of *A. nidulans* was approximated by the biomass composition of *A. oryzae* (strain A1560) grown in glucose limited, continuous cultivations (specific growth rate of 0.17 h^−1^) with ammonia as nitrogen source [22]. The simulation results were not sensitive to the biomass composition (simulation results not shown). The metabolic model of Pedersen et al [22] was used to calculate the precursor requirement for biomass formation. The 9 precursors were assumed to be α-ketoglutarate, acetyl-CoA, erythrose-4-phosphate, glucose-6-phosphate, glyceraldehyde-3-phosphate, mannose, oxaloacetate, pyruvate and ribose-5-phosphate. More details on the precursor calculations can be found in Supplementary Table S2.

A modified version of the metabolic model of David et al [23] was used for simulation of the metabolic fluxes. In all cases, the reaction for phosphoketolase activity was included in the model (Supplementary Table S3). In the special case, where iodo acetate was added to the medium, the reaction representing glyceraldehyde 3-phosphate dehydrogenase was removed and the reactions in the glyoxylate cycle were included (Supplementary Table S3).

#### Computational method

The fluxes were determined by an in-house software based on the metabolic network analysis framework developed by Wiechert [24] implemented in MatLab 7.0 (Mathwork Inc., Natick, MA). The fluxes were found by least square minimisation with the non-linear Levenberg-Marquardt algorithm, where the discrepancies between measured and calculated fluxes as well as measured and calculated SFLs were minimised. Multiple initial guesses for the Levenberg-Marquardt algorithm were generated with a genetic algorithm in order to verify the existence of a global minimum and to address the significance of the simulated result. Selected metabolic fluxes are visualised in the Figure 1 while the complete list can be found in Supplementary Table S3.

### DNA microarrays

#### Harvesting of mycelium and RNA extraction

The cultures were harvested in the late exponential phase at a biomass dry-weight concentration of 6–7 g/L with the exception of the cultivation on ethanol, where the biomass concentration was 3–4 g/L. Mycelium was harvested by filtration through sterile Mira-Cloth (Calbiochem). The mycelium was quickly dried by squeezing, and subsequently frozen in liquid nitrogen. Samples were stored at −80°C until they were used for RNA extraction. Total RNA was isolated using the Qiagen RNeasy Mini Kit, according to the protocol for isolation of total RNA from plant and fungi [11].

#### Microarray hybridization

Fifteen µg of fragmented biotin-labeled cRNA was prepared from 5 µg of total RNA and hybridized to the 3AspergDTU Affymetrix GeneChip [25] according to the Affymetrix GeneChip Expression Analysis Technical Manual [26].

#### Analysis of the transcriptome data

The scanned probe array images (.DAT files) were converted into CEL data files using the GeneChip Operating Software from Affymetrix, and subsequently pre-processed using the statistical language and environment R (www.R-project.org) version 2.5. The probe intensities were normalised for background noise with the robust multiarray average (RMA) method [27] using only perfect match (PM) probes. Normalisation was performed subsequently using the quantiles algorithm [28]. Gene expression values were calculated from the PM probes with the median polish summary method [27].

Statistical analysis with the limma package [29] was applied to determine differentially expressed genes. Moderated *t*-tests between two strains grown on the same carbon sources were used for the comparison of the PHK effect on four different carbon sources. For all comparisons were empirical Bayesian statistics employed to moderate standard errors within each gene and Benjamini-Hochberg's method [30] to adjust p-values for multi-testing. Where nothing else is stated, an adjusted cut-off value of *p*<0.05 was used to determine statistically significant changes.

### Manual annotation

General function (KOG/Interpro/PFAM) was assigned by comparing PFAM predictions to a manual lookup of the bi-directional best hits in the *A. niger* ATCC 1015 genome sequence (http://genome.jgi-psf.org/Aspni1/Aspni1.home.html). Additionally was added *A. nidulans* genes found in the SwissProt database (Supplementary Table S8).

### Pathway analysis

Metabolic pathway analysis was done using an adaptation of a metabolic network map for *A. niger* [31]. Bi-directional best blast hits were used to find *A. nidulans* homologues to metabolic genes. *p*<.05 was employed as a cut-off value for statistical significance.

### Reporter metabolite analysis

The statistical significance of regulation around metabolites was assessed using the reporter feature algorithm with default settings [16], [17]. To assign metabolite-metabolite interactions, an *A. nidulans* metabolic network reconstruction was used [23].

## Supporting Information

Table S1 File containing the measured and calculated SFL (%) of 16 fragments
(0.04 MB PDF)

Table S2 File containing the modified version of the metabolic model from Pedersen et al [22] with details on the precursor calculations.
(0.19 MB PDF)

Table S3 File containing the modified version of the metabolic model of David et al [23] that was used for simulation of the metabolic fluxes as well as calculations of all the metabolic fluxes.
(0.28 MB PDF)

Table S4 Results of reporter feature algorithm for the examination of phosphoketolase over-expression on glucose.
(0.35 MB PDF)

Table S5 Results of reporter feature algorithm for the examination of phosphoketolase over-expression on xylose.
(0.36 MB PDF)

Table S6 Results of reporter feature algorithm for the examination of phosphoketolase over-expression on glycerol.
(0.35 MB PDF)

Table S7 Results of reporter feature algorithm for the examination of phosphoketolase over-expression on ethanol
(0.35 MB PDF)

Table S8 General functional (KOG/Interpro/PFAM) assignment and SwissProt gene names for 2805 genes from A. nidulans.
(0.38 MB PDF)

Figure S1 Metabolic map of differentially regulated genes in a comparison of wild type and a phosphoketolase over-expressing (PHK) strain on glucose medium.
(0.38 MB PDF)

Figure S2 Metabolic map of differentially regulated genes in a comparison of wild type and a phosphoketolase over-expressing (PHK) strain on xylose medium.
(0.38 MB PDF)

Figure S3 Metabolic map of differentially regulated genes in a comparison of wild type and a phosphoketolase over-expressing (PHK) strain on glycerol medium.
(0.38 MB PDF)

Figure S4 Metabolic map of differentially regulated genes in a comparison of wild type and a phosphoketolase over-expressing (PHK) strain on ethanol medium.
(0.38 MB PDF)
