# Supplementary material for: Systems Analysis Unfolds the Relationship between the Phosphoketolase Pathway and Growth in Aspergillus nidulans
Source: PLoS One. 2008 Dec 4;3(12):e3847. doi: 10.1371/journal.pone.0003847 (PMC2585806; doi:10.1371/journal.pone.0003847)
Supplement: Table S1 — File containing the measured and calculated SFL (%) of 16 fragments (0.04 MB PDF) [file pone.0003847.s001.pdf]

Table: Measured (upper value) and calculated (lower value) SFL (%) of 16 fragments. Abbreviation of precursors in the central carbon metabolism: G6P, glucose-6-phosphate; G3P, glyceraldehyde-3-phosphate; PYR, pyruvate; ACCOA, acetyl-CoA; OAA, oxaloacetate; AKG, alpha-ketoglutarate; PEP, phosphoenolpyruvate and E4P, erythrose-4-phosphate.

		A. nidulans strains	A4	gp*ndk*74	gp*ndk*74
**#**	**Fragment**	**Precursors**	**WT**	**PHK**	**PHK+Iodo**
1	ALA116	PYR#2#3	28.7	30.9	26.4
			28.7	30.7	26.5
2	ALA158	PYR#1#2#3	33.2	36.3	30.0
			33.4	36.4	35.0
3	ASP188	OAA#2#3#4	45.4	47.8	39.5
			49.0	52.8	35.0
4	GLC331	G6P#1#2#3#4#5#6	86.9	94.0	77.8
			90.5	98.1	83.9
5	GLU230	AKG#1#2#3#4#5	72.3	80.7	*28.8
			72.0	77.7	53.9
6	ILE158	OAA#2#3#4PYR#2#3	72.2	76.7	65.2
			77.8	83.5	61.6
7	LEU158	ACCOA#2PYR#2#2#3#3	77.2	85.5	67.4
			79.1	85.0	67.3
8	LYS156	AKG#2#3#4#5ACCOA#2	81.1	86.6	69.2
			82.7	89.7	59.7
9	PHE143	PEP#1#2	9.1	8.0	#NA
			6.1	7.0	21.7
10	PHE192	E4P#1#2#3#4PEP#2#2#3#3	70.7	79.4	73.3
			68.2	74.7	80.8
11	THR175	OAA#1#2	35.4	37.6	33.0
			30.0	32.2	21.7
12	VAL143	PYR#1#2	5.1	6.4	*6.2
			6.1	7.0	21.7
13	VAL144	PYR#2#2#3#3	56.4	59.1	52.5
			57.5	61.4	53.1
14	VAL186	PYR#1#2#2#3#3	72.0	77.8	53.9
			62.1	67.1	61.6

*Fragment is likely to be an outlier and was given low weight in the flux estimation procedure
