# Supplementary material for: Systems Analysis Unfolds the Relationship between the Phosphoketolase Pathway and Growth in Aspergillus nidulans
Source: PLoS One. 2008 Dec 4;3(12):e3847. doi: 10.1371/journal.pone.0003847 (PMC2585806; doi:10.1371/journal.pone.0003847)
Supplement: Table S2 — File containing the modified version of the metabolic model from Pedersen et al [22] with details on the precursor calculations. (0.19 MB PDF) [file pone.0003847.s002.pdf]

% Central metabolism of Aspergillus nidulans
% Model adopted from Pedersen et al. (1998)
%
% PUBLICATION
%Systems analysis unfolds the growth effects of the phosphoketolase pathway in Aspergillus nidulans
%
%
% AUTHORS
% Gianni Panagiotou, Thomas Grotkjær, Mikael Rørdam Andersen,
% Torsten Bak Regueira, Gerald Hofmann, Jens Nielsen & Lisbeth Olsson

Transport
trp01 : so4_ext + 4 atp + 4 nadph_cyt -> h2s

Glycolysis
gly01 : dihydroxyacetone-p <-> glyceraldehyde-3-p
gly02 : glyceraldehyde-3-p <-> 13-diphosphoglycerate +
nadh_cyt
gly03 : 13-diphosphoglycerate <-> 3-phosphoglycerate + atp
gly04 : 3-phosphoglycerate <-> phosphoenolpyruvate

TCA cycle
tca01 : acetyl-coa_mit + oxaloacetate_mit -> citrate
tca02 : citrate -> a-ketoglutarate_cyt + nadph_cyt + co2
tca03 : succinyl-coa -> malate + atp + fadh2
tca04 : malate -> oxaloacetate_mit + nadh_mit

Amino acid synthesis
aas01 : a-ketoglutarate_cyt + nadph_cyt + nh4_ext + atp -> glutamate
aas02 : glutamate + nh4_ext + 2 atp -> glutamine
aas03 : glutamate + 2 nadph_cyt + atp -> proline
aas04 : aspartate + glutamate + glutamine + co2 + nh4_ext + nadph_mit + 5 atp -> arginine + a-ketoglutarate_cyt + malate
aas05 : acetyl-coa_mit + 2 glutamate + a-ketoglutarate_mit + 2 atp + 2 nadph_cyt -> lysine + 2 a-ketoglutarate_cyt + nadh_cyt
aas06 : glutamate + 0.59 oxaloacetate_cyt + 0.41 oxaloacetate_mit -> aspartate + 0.59 a-ketoglutarate_cyt + 0.41 a-ketoglutarate_mit
aas07 : aspartate + a-ketoglutarate_mit <-> glutamate + oxaloacetate_mit
aas08 : aspartate + glutamine + 2 atp -> aspargine + glutamate
aas09 : aspartate + 2 nadph_cyt + atp -> homoserine
aas10 : homoserine + atp -> threonine
aas11 : glutamate + pyruvate + threonine + nadph_mit -> isoleucine + a-ketoglutarate_cyt + nh4_ext + co2 + atp
aas12 : homoserine + h2s + 2 atp -> homocysteine
aas13 : homocysteine + nadh_cyt + n5,n10-methylene-thf -> methionine
aas14 : methionine + atp -> s-adenosylmethionine
aas15 : s-adenosylcysteine + 2 atp -> homocysteine
aas16 : 3-phosphoglycerate + glutamate -> serine + a-ketoglutarate_cyt + nadh_cyt
aas17 : serine -> glycine + n5,n10-methylene-thf
aas18 : homocysteine + serine -> cysteine + succinyl-coa + nadh_cyt
aas19 : pyruvate + glutamate -> alanine + a-ketoglutarate_cyt
aas20 : 2 pyruvate + nadph_mit -> ketoisovalerate + co2

aas21 : ketoisovalerate + glutamate -> valine + a-ketoglutarate_mit

aas22 : ketoisovalerate + acetyl-coa_mit + glutamate -> leucine + a-ketoglutarate_mit + co2 + nadh_cyt

aas23 : ribose-5-p + 2 atp -> prpp

aas24 : prpp + atp + glutamine -> histidine + 59-aminoimidazole-4-carboxamide-ribonucleotide + a-ketoglutarate_cyt + nadh_cyt

aas25 : 2 phosphoenolpyruvate + erythrose-4-p + atp + nadph_cyt -> chorismate

aas26 : chorismate + glutamate -> tyrosine + a-ketoglutarate_cyt + nadh_cyt

aas27 : chorismate + glutamate -> phenylalanine + a-ketoglutarate_cyt

aas28 : prpp + serine + chorismate + glutamine -> tryptophan + pyruvate + glutamate + glyceraldehyde-3-p + co2

Nucleotides

nct01 : aspartate + glutamine + prpp + 4 atp -> utp + glutamate + nadh_cyt

nct02 : atp + glutamine + utp -> ctp + glutamate

nct03 : aspartate + 4 atp + 2 glutamine + glycine + prpp + n5,n10-methylene-thf <-> 59-aminoimidazole-4-carboxamide-ribonucleotide + glutamate + malate + nadph_cyt

nct04 : n5,n10-methylene-thf + 59-aminoimidazole-4-carboxamide-ribonucleotide -> imp + nadph_cyt

nct05 : aspartate + 3 atp + imp -> adenosine triphosphate + malate

nct06 : 4 atp + glutamine + imp -> gtp + glutamate + nadh_cyt

nct07 : glycine <-> n5,n10-methylene-thf + co2 + nh4_ext + atp + nadh_mit

Biomass formation

bio01 : 0.095 alanine + 0.044 arginine + 0.046 aspargine + 0.046 aspartate + 0.011 cysteine + 0.080 glutamate + 0.080 glutamine + 0.094 glycine + 0.020 histidine + 0.045 isoleucine + 0.069 leucine + 0.057 lysine + 0.014 methionine + 0.031 phenylalanine + 0.047 proline + 0.066 serine + 0.048 threonine + 0.018 tryptophan + 0.028 tyrosine + 0.064 valine + 4 atp -> protein

bio02 : glucose-6-p + atp -> carbohydrate

bio03 : 18.12 acetyl-coa_cyt + 18 atp + 0.623 dihydroxyacetone-p + 0.623 fadh2 + 0.3 glucose-6-p + 1.7 nadh_cyt + 22.4 nadph_cyt + 1.1 s-adenosylmethionine + 0.4 serine + 2.6 o2 -> lipid + 3.8 co2 + 1.1 s-adenosylcysteine

bio04 : 0.256 adenosine triphosphate + 0.196 ctp + 0.286 gtp + 0.262 utp + atp -> rna

bio05 : 0.242 adenosine triphosphate + 0.258 ctp + 0.258 gtp + 0.242 utp + 1.242 nadph_cyt + 0.242 n5,n10-methylene-thf + atp -> dna

bio06 : 140.242 protein + 46.391 carbohydrate + 2.494 rna + 0.423 dna + 0.357 lipid + 3.279 mannitol -> 1000 biomass

List of metabolites in metabolic model
Lower and upper bounds for exchange fluxes
A negative flux is uptake
A positive flux is excretion
Columns:
1. Number
2. Metabolite
3. Lower flux
4. Upper flux

1	13-diphosphoglycerate	0	0
2	3-phosphoglycerate	0	0
3	59-aminoimidazole-4-carboxamide-ribonucleotide	0	0
4	a-ketoglutarate_cyt	0	0
5	a-ketoglutarate_mit	-1000	0
6	acetyl-coa_cyt	-1000	0
7	acetyl-coa_mit	-1000	0
8	adenosine triphosphate	0	0
9	alanine	0	0
10	arginine	0	0
11	aspargine	0	0
12	aspartate	0	0
13	atp	-1000	1000
14	biomass	0	1000
15	carbohydrate	0	0
16	chorismate	0	0
17	citrate	0	0
18	co2	0	1000
19	ctp	0	0
20	cysteine	0	0
21	dihydroxyacetone-p	0	0
22	dna	0	0
23	erythrose-4-p	-1000	0
24	fadh2	-1000	1000
25	glucose-6-p	-1000	0
26	glutamate	0	0
27	glutamine	0	0
28	glyceraldehyde-3-p	-1000	0
29	glycine	0	0
30	gtp	0	0
31	h2s	0	0
32	histidine	0	0
33	homocysteine	0	0
34	homoserine	0	0
35	imp	0	0
36	isoleucine	0	0
37	ketoisovalerate	0	0
38	leucine	0	0
39	lipid	0	0
40	lysine	0	0
41	malate	0	0

42	mannitol	-1000	0
43	methionine	0	0
44	n5,n10-methylene-thf	0	0
45	nadh_cyt	-1000	1000
46	nadh_mit	-1000	1000
47	nadph_cyt	-1000	1000
48	nadph_mit	-1000	1000
49	nh4_ext	-1000	0
50	o2	-1000	0
51	oxaloacetate_cyt	-1000	0
52	oxaloacetate_mit	0	0
53	phenylalanine	0	0
54	phosphoenolpyruvate	0	0
55	proline	0	0
56	protein	0	0
57	prpp	0	0
58	pyruvate	-1000	0
59	ribose-5-p	-1000	0
60	rna	0	0
61	s-adenosylcysteine	0	0
62	s-adenosylmethionine	0	0
63	serine	0	0
64	so4_ext	-1000	0
65	succinyl-coa	0	0
66	threonine	0	0
67	tryptophan	0	0
68	tyrosine	0	0
69	utp	0	0
70	valine	0	0

List of simulated fluxes:
1. Uptake/excretion fluxes
2. Balanced fluxes
#
Columns
1. Number
2. Index (reaction number, exchange and balanced)
3. Name of internal reactions (ORF, enzyme etc.)
4. Reaction (extension EXT indicates exchange reaction)
5. Lower boundary for flux
6. Upper boundary for flux
7. Simulated flux
8. Shadow prices (duals)

List of EXCHANGE REACTIONS:

Number	Index	Reaction	Lower	Upper	Flux	Shadow
1	1	a-ketoglutarate_mit_EXCH_REAC	-1000	0	-12,0	0
2	2	acetyl-coa_cyt_EXCH_REAC	-1000	0	-6,5	0
3	3	acetyl-coa_mit_EXCH_REAC	-1000	0	-47,6	0
4	4	atp_EXCH_REAC	-1000	1000	-941,9	0
5	5	biomass_EXCH_REAC	0	1000	1000,0	0
6	6	co2_EXCH_REAC	0	1000	57,7	0
7	7	erythrose-4-p_EXCH_REAC	-1000	0	-10,8	0
8	8	fadh2_EXCH_REAC	-1000	1000	1,3	0
9	9	glucose-6-p_EXCH_REAC	-1000	0	-46,5	0
10	10	glyceraldehyde-3-p_EXCH_REAC	-1000	0	-40,1	0
11	11	mannitol_EXCH_REAC	-1000	0	-3,3	0
12	12	nadh_cyt_EXCH_REAC	-1000	1000	88,4	0
13	13	nadh_mit_EXCH_REAC	-1000	1000	2,6	0
14	14	nadph_cyt_EXCH_REAC	-1000	1000	-228,2	0
15	15	nadph_mit_EXCH_REAC	-1000	1000	-31,1	0
16	16	nh4_ext_EXCH_REAC	-1000	0	-196,7	0
17	17	o2_EXCH_REAC	-1000	0	-0,9	0
18	18	oxaloacetate_cyt_EXCH_REAC	-1000	0	-59,2	0
19	19	pyruvate_EXCH_REAC	-1000	0	-54,4	0
20	20	ribose-5-p_EXCH_REAC	-1000	0	-5,4	0
21	21	so4_ext_EXCH_REAC	-1000	0	-3,5	0

List of BALANCED REACTIONS:

Number	Index	Reaction	Lower	Upper	Flux	Shadow	Name
1	1	so4_ext + 4 atp + 4 nadph_cyt -> h2s	0	1000	3,5	0	trp01
2	2	dihydroxyacetone-p <-> glyceraldehyde-3-p	-1000	1000	-0,2	0	gly01
3	3	glyceraldehyde-3-p <-> 13-diphosphoglycerate + nadh_cyt	-1000	1000	42,4	0	gly02
4	4	13-diphosphoglycerate <-> 3-phosphoglycerate + atp	-1000	1000	42,4	0	gly03
5	5	3-phosphoglycerate <-> phosphoenolpyruvate	-1000	1000	21,6	0	gly04
6	6	acetyl-coa_mit + oxaloacetate_mit -> citrate	0	1000	29,9	0	tca01
7	7	citrate -> a-ketoglutarate_cyt + nadph_cyt + co2	0	1000	29,9	0	tca02
8	8	succinyl-coa -> malate + atp + fadh2	0	1000	1,5	0	tca03
9	9	malate -> oxaloacetate_mit + nadh_mit	0	1000	7,2	0	tca04
10	10	a-ketoglutarate_cyt + nadph_cyt + nh4_ext + atp -> glutamate	0	1000	162,8	0	aas01
11	11	glutamate + nh4_ext + 2 atp -> glutamine	0	1000	29,5	0	aas02
12	12	glutamate + 2 nadph_cyt + atp -> proline	0	1000	6,6	0	aas03
13	13	aspartate + glutamate + glutamine + co2 + nh4_ext + nadph_mit + 5 atp -> arginine + a-ketoglutarat	0	1000	6,2	0	aas04
14	14	acetyl-coa_mit + 2 glutamate + a-ketoglutarate_mit + 2 atp + 2 nadph_cyt -> lysine + 2 a-ketoglutarat	0	1000	8,0	0	aas05
15	15	glutamate + 0.59 oxaloacetate_cyt + 0.41 oxaloacetate_mit -> aspartate + 0.59 a-ketoglutarate_cyt +	0	1000	100,3	0	aas06
16	16	aspartate + a-ketoglutarate_mit <-> glutamate + oxaloacetate_mit	-1000	1000	63,8	0	aas07
17	17	aspartate + glutamine + 2 atp -> aspargine + glutamate	0	1000	6,5	0	aas08
18	18	aspartate + 2 nadph_cyt + atp -> homoserine	0	1000	16,5	0	aas09
19	19	homoserine + atp -> threonine	0	1000	13,0	0	aas10
20	20	glutamate + pyruvate + threonine + nadph_mit -> isoleucine + a-ketoglutarate_cyt + nh4_ext + co2 +	0	1000	6,3	0	aas11
21	21	homoserine + h2s + 2 atp -> homocysteine	0	1000	3,5	0	aas12
22	22	homocysteine + nadh_cyt + n5,n10-methylene-thf -> methionine	0	1000	2,4	0	aas13
23	23	methionine + atp -> s-adenosylmethionine	0	1000	0,4	0	aas14
24	24	s-adenosylcysteine + 2 atp -> homocysteine	0	1000	0,4	0	aas15
25	25	3-phosphoglycerate + glutamate -> serine + a-ketoglutarate_cyt + nadh_cyt	0	1000	20,8	0	aas16
26	26	serine -> glycine + n5,n10-methylene-thf	0	1000	7,4	0	aas17
27	27	homocysteine + serine -> cysteine + succinyl-coa + nadh_cyt	0	1000	1,5	0	aas18
28	28	pyruvate + glutamate -> alanine + a-ketoglutarate_cyt	0	1000	13,3	0	aas19
29	29	2 pyruvate + nadph_mit -> ketoisovalerate + co2	0	1000	18,7	0	aas20
30	30	ketoisovalerate + glutamate -> valine + a-ketoglutarate_mit	0	1000	9,0	0	aas21
31	31	ketoisovalerate + acetyl-coa_mit + glutamate -> leucine + a-ketoglutarate_mit + co2 + nadh_cyt	0	1000	9,7	0	aas22
32	32	ribose-5-p + 2 atp -> prpp	0	1000	5,4	0	aas23
33	33	prpp + atp + glutamine -> histidine + 59-aminoimidazole-4-carboxamide-ribonucleotide + a-ketogluta	0	1000	2,8	0	aas24
34	34	2 phosphoenolpyruvate + erythrose-4-p + atp + nadph_cyt -> chorismate	0	1000	10,8	0	aas25
35	35	chorismate + glutamate -> tyrosine + a-ketoglutarate_cyt + nadh_cyt	0	1000	3,9	0	aas26

**Miscellaneous information**				**Wild type**	**PHK**	**PHK+HA**
Molecular weight of dry biomass (g/C-mole)	24,75		Yield (g/g)	0,480	0,490	0,410
Molecular weight of glucose (g/C-mole)	30,00		Yield ash free (g/g)	0,432	0,441	0,369
Ash content of biomass	10%		Yield (C-mol/C-mol)	0,524	0,535	0,447

**#**	**Reaction**	**Lower**	**Upper**	**Flux**			
1	glucose (uptake)	-1000	0	-100,00	100	100	100
1	a-ketoglutarate_mit_EXCH_REAC	-1000	0	-12,04	3,78	3,86	3,23
2	acetyl-coa_cyt_EXCH_REAC	-1000	0	-6,47	2,03	2,07	1,74
3	acetyl-coa_mit_EXCH_REAC	-1000	0	-47,58	14,95	15,26	12,77
4	erythrose-4-p_EXCH_REAC	-1000	0	-10,80	3,39	3,46	2,90
5	glucose-6-p_EXCH_REAC	-1000	0	-46,50	14,61	14,91	12,48
6	glyceraldehyde-3-p_EXCH_REAC	-1000	0	-40,12	12,61	12,87	10,77
7	mannitol_EXCH_REAC	-1000	0	-3,28	1,03	1,05	0,88
8	oxaloacetate_cyt_EXCH_REAC	-1000	0	-59,17	18,59	18,98	15,88
9	pyruvate_EXCH_REAC	-1000	0	-54,41	17,10	17,45	14,60
10	ribose-5-p_EXCH_REAC	-1000	0	-5,44	1,71	1,75	1,46
1	biomass_EXCH_REAC	0	1000	1000,00	314,18	320,73	268,36
2	co2_EXCH_REAC	0	1000	57,68	18,12	18,50	15,48
