# Supplementary material for: Systems Analysis Unfolds the Relationship between the Phosphoketolase Pathway and Growth in Aspergillus nidulans
Source: PLoS One. 2008 Dec 4;3(12):e3847. doi: 10.1371/journal.pone.0003847 (PMC2585806; doi:10.1371/journal.pone.0003847)
Supplement: Table S3 — File containing the modified version of the metabolic model of David et al [23] that was used for simulation of the metabolic fluxes as well as calculations of all the metabolic fluxes. (0.28 MB PDF) [file pone.0003847.s003.pdf]

Standard Model

```
% METABOLIC MODEL
% Central metabolism of Aspergillus nidulans
%
% PUBLICATION
% Systems analysis unfolds the growth effects of the phosphoketolase pathway in Aspergillus
nidulans
%
%
% AUTHORS
% Gianni Panagiotou, Thomas Grotkjær, Mikael Rørdam Andersen,
% Torsten Bak Regueira, Gerald Hofmann, Jens Nielsen & Lisbeth Olsson
%
% December 2007
%
% ------------------------------------------------------------------------
    #NAME?

% Embden-Meyerhoff-Parnas Pathway
EMP1: GLC <-> G6P
abcdef <-> abcdef
net = 100.0; exch = 0.0;

EMP2: G6P <-> F6P
abcdef <-> abcdef
net 0.0 100.0;

EMP3: F6P <-> G3P + G3P
abcdef <-> cba + def
net 0.0 100.0; exch = 0.0;

EMP4: G3P <-> PEP
abc <-> abc
net 0.0 200.0;

EMP5: PEP <-> PYR
abc <-> abc
net 0.0 200.0; exch = 0.0;

% Pentose-Phosphate Pathway
PPP1: G6P <-> CO2 + R5P
abcdef <-> a + bcdef
net 0.0 100.0; exch = 0.0;

PPP2: R5P + R5P <-> S7P + G3P
abcde + fghij <-> fgabcde + hij
net 0.0 100.0;

PPP3: R5P + E4P <-> F6P + G3P
abcde + fghi <-> abfghi + cde
```

```
net 0.0 100.0;

PPP4: S7P + G3P <-> F6P + E4P
abcdefg + hij <-> abchij + defg
net 0.0 100.0;

% Tricarboxylic Acid Cycle
TCA1: OAA + ACCOA <-> CIT
abcd + ef <-> dcbafe
net 0.0 200.0; exch = 0.0;

TCA2: CIT <-> AKG + CO2
abcdef <-> abcef + d
net 0.0 200.0; exch = 0.0;

TCA3: AKG <-> CO2 + SUC
abcde <-> a + bcde
net 0.0 200.0; exch = 0.0;

TCA4: SUC + SUC <-> OAA + OAA
abcd + efgh <-> abcd + hgfe
net 0.0 200.0;

% Additional reactions
%
% PHOKL: Phosphoketolase
% PYRCL: Pyruvate carboxylase
% CAKIN: PEP carbokinase
% 6MSAS: Product formation
PHOKL: R5P <-> G3P + ACCOA
abcde <-> abc + de
net 0.0 200.0; exch = 0.0;

PYRCL: PYR + CO2 <-> OAA
abc + d <-> abcd
net -200.0 200.0; exch = 0.0;

PYRDH: PYR <-> CO2 + ACCOA
abc <-> a + bc
net 0.0 200.0; exch = 0.0;




% Drain fluxes for biomass formation
%
% See listed fluxes
DRAIN_AKG: AKG <-> AKGBIO
abcde <-> abcde
net 0.0 100.0; exch = 0.0;
```

DRAIN_ACCOA: ACCOA <-> ACCOABIO
ab <-> ab
net 0.0 100.0; exch = 0.0;

DRAIN_E4P: E4P <-> E4PBIO
abcd <-> abcd
net 0.0 100.0; exch = 0.0;

DRAIN_G6P: G6P <-> G6PBIO
abcdef <-> abcdef
net 0.0 100.0; exch = 0.0;

DRAIN_G3P: G3P <-> G3PBIO
abc <-> abc
net 0.0 100.0; exch = 0.0;

DRAIN_MAN: G6P <-> MANBIO
abcdef <-> abcdef
net 0.0 100.0; exch = 0.0;

DRAIN_OAA: OAA <-> OAABIO
abcd <-> abcd
net 0.0 100.0; exch = 0.0;

DRAIN_PYR: PYR <-> PYRBIO
abc <-> abc
net 0.0 100.0; exch = 0.0;

DRAIN_R5P: R5P <-> R5PBIO
abcde <-> abcde
net 0.0 100.0; exch = 0.0;

DRAIN_CO2: CO2 <-> CO2EXT
a <-> a
net 0.0 600.0; exch = 0.0;

% --
#NAME?
GLC: 99.0 1.1 1.1 1.1 1.1 1.1;

% --
#NAME?

AKGBIO
ACCOABIO
E4PBIO
G6PBIO
G3PBIO

MANBIO
OAABIO
PYRBIO
R5PBIO
CO2EXT

#NAME?

Iodoacetate Model

```
% METABOLIC MODEL
% Central metabolism of Aspergillus nidulans
%
% PUBLICATION
% Metabolic network and gene expression analysis in
% Aspergillus nidulans in response to an active phosphoketolase pathway
%
% AUTHORS
% Gianni Panagiotou, Thomas Grotkjær, Mikael Rørdam Andersen,
% Torsten Bak Regueira, Gerald Hofmann, Jens Nielsen & Lisbeth Olsson
%
% May 2007
%
% ----------------------------------------------------------------------
--
 #NAME?

% Embden-Meyerhoff-Parnas Pathway
EMP1: GLC <-> G6P
abcdef <-> abcdef
net = 100.0; exch = 0.0;

EMP2: G6P <-> F6P
abcdef <-> abcdef
net -200.0 200.0;

EMP3: F6P <-> G3P + G3P
abcdef <-> cba + def
net -200.0 200.0; exch = 0.0;

% Reaction not present due to inhibition!
% EMP4: G3P <-> PEP
% abc <-> abc
% net = 0.0; exch = 0.0;

EMP5: PEP <-> PYR
abc <-> abc
```

```
net 0.0 300.0; exch = 0.0;

% Pentose-Phosphate Pathway
PPP1: G6P <-> CO2 + R5P
abcdef <-> a + bcdef
net 0.0 100.0; exch = 0.0;

PPP2: R5P + R5P <-> S7P + G3P
abcde + fghij <-> fgabcde + hij
net -200.0 0.0;

PPP3: R5P + E4P <-> F6P + G3P
abcde + fghi <-> abfghi + cde
net -200.0 0.0;

PPP4: S7P + G3P <-> F6P + E4P
abcdefg + hij <-> abchij + defg
net -200.0 0.0;

% Tricarboxylic Acid Cycle
TCA1: OAA + ACCOA <-> CIT
abcd + ef <-> dcbafe
net 0.0 200.0; exch = 0.0;

TCA2: CIT <-> AKG + CO2
abcdef <-> abcef + d
net 0.0 200.0; exch = 0.0;

TCA3: AKG <-> CO2 + SUC
abcde <-> a + bcde
net 0.0 200.0; exch = 0.0;

TCA4: SUC + SUC <-> OAA + OAA
abcd + efgh <-> abcd + hgfe
net 0.0 200.0;

% Glyoxylate cycle
GLY1: CIT <-> GLX + OAA
abcdef <-> ab + cdef
net 0.0 200.0; exch = 0.0;

GLY2: GLX + ACCOA <-> OAA
ab + cd <-> abdc
net 0.0 200.0; exch = 0.0;

% Additional reactions
%
% PHOKL: Phosphoketolase
% CAKIN: PEP carbokinase
PHOKL: R5P <-> G3P + ACCOA
abcde <-> abc + de
net 0.0 300.0; exch = 0.0;
```

```
CAKIN: OAA <-> PEP + CO2
abcd <-> abc + d
net 0.0 300.0; exch = 0.0;

% Drain fluxes for biomass formation
%
% See listed fluxes
DRAIN_AKG: AKG <-> AKGBIO
abcde <-> abcde
net 0.0 100.0; exch = 0.0;

DRAIN_ACCOA: ACCOA <-> ACCOABIO
ab <-> ab
net 0.0 100.0; exch = 0.0;

DRAIN_E4P: E4P <-> E4PBIO
abcd <-> abcd
net 0.0 100.0; exch = 0.0;

DRAIN_G6P: G6P <-> G6PBIO
abcdef <-> abcdef
net 0.0 100.0; exch = 0.0;

DRAIN_G3P: G3P <-> G3PBIO
abc <-> abc
net 0.0 100.0; exch = 0.0;

DRAIN_MAN: G6P <-> MANBIO
abcdef <-> abcdef
net 0.0 100.0; exch = 0.0;

DRAIN_OAA: OAA <-> OAABIO
abcd <-> abcd
net 0.0 100.0; exch = 0.0;

DRAIN_PYR: PYR <-> PYRBIO
abc <-> abc
net 0.0 100.0; exch = 0.0;

DRAIN_R5P: R5P <-> R5PBIO
abcde <-> abcde
net 0.0 100.0; exch = 0.0;

DRAIN_CO2: CO2 <-> CO2EXT
a <-> a
net 0.0 600.0; exch = 0.0;

% ----------------------------------------------------------------------
--
 #NAME?
GLC: 99.0 1.1 1.1 1.1 1.1 1.1;
```

% ---
--
#NAME?

AKGBIO
ACCOABIO
E4PBIO
G6PBIO
G3PBIO
MANBIO
OAABIO
PYRBIO
R5PBIO
CO2EXT

#NAME?

Calculated Fluxes

Calculated net fluxes

#	Abbreviation	Reaction	WT	PHK	PHK_Iodo
1	EMP1	GLC <-> G6P	100,0	100,0	100,0
2	EMP2	G6P <-> F6P	27,5	39,6	-13,4
3	EMP3	F6P <-> G3P + G3P	43,6	45,0	-71,4
4	EMP4	G3P <-> PEP	110,2	110,9	---
5	EMP5	PEP <-> PYR	110,2	110,9	14,6
6	PPP1	G6P <-> CO2 + R5P	56,9	44,4	100,0
7	PPP2	R5P + R5P <-> S7P + G3P	9,8	4,4	-27,6
8	PPP3	R5P + E4P <-> F6P + G3P	6,4	1,0	-30,5
9	PPP4	S7P + G3P <-> F6P + E4P	9,8	4,4	-27,6
10	TCA1	OAA + ACCOA <-> CIT	82,8	85,9	135,9
11	TCA2	CIT <-> AKG + CO2	82,8	85,9	102,2
12	TCA3	AKG <-> CO2 + SUC	79,1	82,0	99,0
13	TCA4	SUC + SUC <-> OAA + OAA	39,5	41,0	49,5
14	GLY1	CIT <-> GLX + OAA	---	---	33,7
15	GLY2	GLX + ACCOA <-> OAA	---	---	33,7
16	PHOKL	R5P <-> G3P + ACCOA	29,2	32,8	184,1
17	PYRCL	PYR + CO2 <-> OAA	22,5	23,0	---
18	PYRDH	PYR <-> CO2 + ACCOA	70,6	70,4	---
19	CAKIN	OAA <-> PEP + CO2	---	---	14,6
21	DRAIN_AKG		3,8	3,9	3,2
22	DRAIN_ACCOA		17,0	17,4	14,5

23	DRAIN_E4P	3,4	3,5	2,9
24	DRAIN_G6P	14,6	14,9	12,5
25	DRAIN_G3P	12,6	12,9	10,8
26	DRAIN_MAN	1,0	1,1	0,9
27	DRAIN_OAA	18,7	19,1	15,9
28	DRAIN_PYR	17,1	17,5	14,6
29	DRAIN_R5P	1,7	1,8	1,5
30	DRAIN_CO2	266,9	259,6	315,8
