# Supplementary material for: Systems Analysis Unfolds the Relationship between the Phosphoketolase Pathway and Growth in Aspergillus nidulans
Source: PLoS One. 2008 Dec 4;3(12):e3847. doi: 10.1371/journal.pone.0003847 (PMC2585806; doi:10.1371/journal.pone.0003847)
Supplement: Table S4 — Results of reporter feature algorithm for the examination of phosphoketolase over-expression on glucose. (0.35 MB PDF) [file pone.0003847.s004.pdf]

#Total number of ORFs used = 576

#Total number of significantly changed ORFs = 0

#Mean_all = -0.018646 #Std_all= 1.341

#kmax, imax = 100, 10000

#Feature	Number of neighbors	Z-score	P-value	Average Z	StdDev Z	Significance count
DGLCe	4	-0.167949	0.566688	-0.131583	1.24728	1
GLCe	19	0.141572	0.443709	0.0247255	1.45827	4
bDGLCe	3	0.724191	0.234474	0.541957	0.51127	0
DGLC	2	-0.464448	0.678837	-0.459826	1.6793	0
GLC	20	1.23974	0.107535	0.352569	1.72116	6
bDGLC	7	0.0237083	0.490543	-0.00685958	1.29051	1
ATP	89	-0.330727	0.629575	-0.0656144	1.13453	8
ADP	59	-0.127337	0.550663	-0.0409121	1.16397	7
G6P	10	-0.160074	0.563589	-0.0866832	1.42598	1
bDG6P	5	-0.92317	0.822041	-0.572583	1.34867	0
H2O	73	0.705236	0.240332	0.0917381	1.68815	15
PI	58	-0.799126	0.787891	-0.159166	1.33164	7
F6P	14	0.295618	0.383761	0.0870593	1.01554	1
FDP	3	0.217378	0.413957	0.149385	1.87545	1

S7P	6	0.304862	0.380236	0.147993	1.06647	1
S17P	3	0.217378	0.413957	0.149385	1.87545	1
T3P2	7	-1.34074	0.909998	-0.698282	1.15722	1
T3P1	11	0.463169	0.321622	0.168335	1.53783	2
E4P	7	-1.37243	0.915036	-0.714341	0.87163	0
NAD	72	-0.15586	0.561929	-0.0432971	1.41655	10
13PDG	6	-0.139816	0.555597	-0.0954285	1.36889	1
NADH	72	-0.15586	0.561929	-0.0432971	1.41655	10
3PG	5	-0.357379	0.639596	-0.233252	1.74929	1
2PG	3	-1.8955	0.970987	-1.48723	0.707148	0
23PDG	2	-1.34067	0.909986	-1.29134	0.877432	0
PEP	6	-2.08224	0.98134	-1.15873	0.739704	0
PYR	17	0.346414	0.364516	0.0937703	1.51681	3
CO2	41	0.527836	0.298807	0.0916221	1.28292	7
OA	12	0.748931	0.22695	0.270912	1.10308	2
ATPm	22	-0.344941	0.634931	-0.117281	1.25452	3
PYRm	9	0.58331	0.279842	0.241783	1.20775	2
CO2m	14	0.247106	0.402413	0.0696862	0.940674	2
ADPm	14	-1.72164	0.957432	-0.635357	0.863311	1

PIm	15	-0.930778	0.824016	-0.34081	1.09586	2
OAm	10	0.230631	0.408801	0.0789157	1.36711	2
GTP	10	-1.63948	0.949443	-0.713724	1.14356	0
GDP	7	-1.51253	0.9348	-0.785331	1.46238	0
NADP	64	1.28895	0.0987079	0.196905	1.55123	13
D6PGL	2	-0.631015	0.735985	-0.617896	0.416901	0
NADPH	64	1.28895	0.0987079	0.196905	1.55123	13
D6PGC	7	-0.112047	0.544607	-0.0756521	1.38081	1
RL5P	5	-0.639112	0.738625	-0.40222	1.2328	1
XUL5P	6	1.44218	0.0746251	0.770575	1.8881	2
R5P	15	-0.84582	0.801173	-0.311418	0.976097	0
ACTP	2	1.35394	0.0878785	1.26579	3.46938	1
ACCOAm	11	1.50774	0.0658106	0.590436	0.980494	2
H2Om	19	-0.176485	0.570044	-0.0730236	1.41029	2
CITm	5	0.18404	0.426991	0.0914623	0.746358	0
COAm	13	0.911243	0.181084	0.319859	1.12549	2
ACOm	3	-0.174873	0.56941	-0.154448	0.413739	0
ICITm	5	-0.383907	0.649476	-0.249162	0.403231	0
NADm	22	1.3639	0.0863	0.370721	1.80538	6

AKGm	9	-0.296746	0.61667	-0.151436	0.855433	0
NADHm	22	1.3639	0.0863	0.370721	1.80538	6
ICIT	2	-0.392167	0.652533	-0.391232	0.490652	0
AKG	19	0.110199	0.456126	0.0150834	1.33604	2
NADPm	17	0.363071	0.358276	0.0991829	1.56196	2
NADPHm	17	0.363071	0.358276	0.0991829	1.56196	2
ICITg	2	0.782578	0.216938	0.723583	1.08594	0
NADPg	1	-0.018648	0.507439	-0.0442891	0	0
AKGg	1	-0.018648	0.507439	-0.0442891	0	0
CO2g	1	-0.018648	0.507439	-0.0442891	0	0
NADPHg	1	-0.018648	0.507439	-0.0442891	0	0
LIPOm	2	0.122304	0.451329	0.0969927	0.193725	0
SUCDLIP	2	-0.83769	0.798898	-0.814027	1.09465	0
SUCCOAm	2	-1.21102	0.887057	-1.16832	0.593613	0
DHLIPOm	2	-0.89249	0.813935	-0.866032	1.02111	0
GDPm	2	-1.02268	0.84677	-0.989577	0.340838	0
GTPm	2	-1.02268	0.84677	-0.989577	0.340838	0
SUCCm	3	-0.494189	0.689414	-0.401787	0.306389	0
Qm	9	-1.03547	0.849776	-0.481508	0.71713	0

FUMm	3	-0.64706	0.741204	-0.5202	0.509046	0
QH2m	9	-1.03547	0.849776	-0.481508	0.71713	0
FADH2m	7	-0.301671	0.618549	-0.171743	1.70908	1
FADm	7	-0.301671	0.618549	-0.171743	1.70908	1
FUM	5	-0.843165	0.800432	-0.5246	1.06768	0
SUCC	6	-0.312257	0.622577	-0.189824	1.0326	0
MALm	4	-0.856332	0.804093	-0.593253	1.75924	1
MAL	4	0.297229	0.383146	0.180392	1.51792	1
MALg	2	1.40732	0.079667	1.31645	0.576848	1
NADg	1	0.690937	0.244802	0.908557	0	0
OAg	1	0.690937	0.244802	0.908557	0	0
NADHg	1	0.690937	0.244802	0.908557	0	0
SUCCg	1	1.12502	0.13029	1.49146	0	0
GLXg	2	1.71443	0.0432247	1.6079	0.164676	1
ACCOAg	2	1.4453	0.0741867	1.3525	0.525868	1
H2Og	1	1.29845	0.0970658	1.72434	0	1
COAg	2	1.4453	0.0741867	1.3525	0.525868	1
Hm	11	0.230177	0.408977	0.074185	1.75661	2
CIT	1	0.175327	0.430411	0.216185	0	0

COA	37	0.894263	0.185591	0.178095	1.00474	4
ACCOA	24	0.412222	0.340088	0.093932	1.11271	3
CAR	2	0.977553	0.164148	0.908611	0.985047	0
ACAR	2	0.977553	0.164148	0.908611	0.985047	0
ACARm	2	0.648521	0.258324	0.596366	0.543464	0
CARm	2	0.648521	0.258324	0.596366	0.543464	0
ACARg	2	0.648521	0.258324	0.596366	0.543464	0
CARg	2	0.648521	0.258324	0.596366	0.543464	0
OXAL	3	-0.581872	0.719673	-0.469705	0.462621	0
AC	17	0.957233	0.169225	0.292248	1.52451	4
FOR	7	-0.668801	0.748189	-0.357782	0.874984	0
FORm	2	-0.796396	0.787099	-0.77484	0.326351	0
METHOL	4	2.82614	0.00235566	1.87642	1.36027	2
FALD	8	1.64436	0.0500507	0.760504	1.5543	2
ADHLIPOm	2	0.0675035	0.47309	0.0449881	0.26727	0
ACAL	13	2.40131	0.0081683	0.873656	1.8857	4
RGT	7	0.0685934	0.472657	0.0158855	1.06759	1
FGT	2	0.419041	0.337593	0.378592	0.374265	0
H+	25	0.121579	0.451616	0.0138003	1.68342	4

HCIT	1	0.776251	0.2188	1.02312	0	0
MTHGXL	3	-1.07876	0.859653	-0.85459	0.713413	0
LACAL	5	0.114774	0.454312	0.04992	2.34262	1
LAC	4	0.272669	0.392554	0.16392	2.69437	1
LGT	2	-1.11674	0.867948	-1.07884	0.89642	0
LLAC	3	0.338575	0.367465	0.243263	1.19499	0
PROP	2	1.6712	0.047341	1.56687	1.03466	1
AMP	38	-0.188402	0.574719	-0.0596634	1.14849	2
PPI	45	0.592013	0.276921	0.0994066	1.03769	3
PROPCOA	3	1.93535	0.0264734	1.48011	0.74689	1
2MCIT	1	0.987348	0.161736	1.30658	0	0
GLU	34	0.564789	0.286109	0.110946	1.29332	5
GABA	3	1.8396	0.0329135	1.40594	2.44974	2
SUCCSAL	5	-1.00113	0.841618	-0.619339	1.06182	0
METTHF	6	-1.40373	0.919801	-0.78731	0.783466	0
METHF	2	-0.913087	0.819402	-0.885578	0.482959	0
METTHFm	6	-0.333971	0.630799	-0.201711	0.776865	0
MTHFm	2	0.716771	0.236758	0.661134	0.420373	0
METHFm	2	-0.913087	0.819402	-0.885578	0.482959	0

FTHFm	1	-0.390839	0.652042	-0.544075	0	0
FTHF	2	0.313537	0.376936	0.27847	1.16325	0
THFm	3	-0.537262	0.704457	-0.435151	0.261735	0
AHTD	2	-1.26405	0.896893	-1.21863	0.776338	0
DHP	2	-1.08974	0.862087	-1.05322	1.01026	0
AHHMP	1	-0.238014	0.594065	-0.338859	0	0
GLAL	1	-0.238014	0.594065	-0.338859	0	0
CHOR	3	0.449177	0.326652	0.328934	0.477213	0
GLN	13	-1.12322	0.869328	-0.436269	0.672513	0
PABA	1	-0.238014	0.594065	-0.338859	0	0
AHHMD	1	-0.238014	0.594065	-0.338859	0	0
DHPT	1	-0.238014	0.594065	-0.338859	0	0
DHF	1	-0.56921	0.715393	-0.783595	0	0
THF	8	-0.535992	0.704018	-0.272898	0.918706	0
MTHF	1	-0.557284	0.711333	-0.767581	0	0
THFG	2	0.123661	0.450792	0.0982806	0.1213	0
OIVAL	2	-1.68019	0.95354	-1.61355	0.632718	0
AKP	1	-1.52046	0.935802	-2.06095	0	0
PANT	1	-1.36857	0.914433	-1.85699	0	0

AKPm	1	0.537665	0.295404	0.70274	0	0
PANTm	1	0.537665	0.295404	0.70274	0	0
bALA	1	-1.36857	0.914433	-1.85699	0	0
PNTO	2	-1.91298	0.972124	-1.83446	0.0318692	0
4PPNTO	1	-1.33501	0.909063	-1.81192	0	0
CTP	7	-1.3329	0.908717	-0.694306	1.32411	0
CYS	7	-0.961958	0.831965	-0.506336	0.436834	0
CMP	8	0.767065	0.221521	0.3447	1.53419	1
ASP	18	-0.253333	0.599994	-0.0987819	1.08612	0
PAP	3	-1.5452	0.938852	-1.21589	0.446353	0
ACP	10	0.742906	0.228769	0.296042	0.691584	1
ALA	6	-0.279646	0.610125	-0.171973	1.30479	0
CHCOA	1	-0.522326	0.699278	-0.720638	0	0
AONA	1	-0.522326	0.699278	-0.720638	0	0
DTB	1	-0.479782	0.684309	-0.66351	0	0
BT	1	-0.479782	0.684309	-0.66351	0	0
ETH	4	2.82614	0.00235566	1.87642	1.36027	2
ETHm	4	2.82614	0.00235566	1.87642	1.36027	2
ACALm	9	2.00741	0.0223533	0.878086	2.11698	3

ACm	9	1.0639	0.143686	0.456518	1.99338	3
AMPm	9	1.3065	0.0956909	0.564914	1.44285	2
PPIm	11	1.58524	0.0564562	0.621753	1.20735	2
ACTPm	1	-0.869946	0.807835	-1.18743	0	0
GLYN	11	0.00346969	0.498616	-0.0174254	0.894167	1
GL	14	1.50005	0.0668012	0.518387	1.28577	2
GLYAL	9	1.1605	0.122924	0.499677	1.62226	2
O2	34	1.17728	0.119542	0.251596	1.97015	10
H2O2	10	0.156504	0.437818	0.0474975	1.99049	2
GL3P	7	-0.674203	0.749909	-0.360519	0.904701	0
TAR	2	-0.807027	0.790175	-0.784929	2.22843	0
OXGLY	2	-0.807027	0.790175	-0.784929	2.22843	0
G	1	1.61577	0.0530717	2.15045	0	1
E	6	-0.948391	0.828535	-0.538051	0.612997	0
EOL	6	-0.948391	0.828535	-0.538051	0.612997	0
LXUL	6	0.347452	0.364126	0.171307	1.75163	1
XOL	6	0.202571	0.419735	0.0919974	0.529649	0
XUL	7	0.858088	0.195422	0.415954	0.984078	1
AOL	6	0.202571	0.419735	0.0919974	0.529649	0

XYL	3	0.280958	0.389371	0.198633	0.554325	0
ARAB	2	-0.54956	0.708689	-0.540596	0.138327	0
ARABLAC	2	-0.54956	0.708689	-0.540596	0.138327	0
LAOL	3	0.485736	0.313577	0.357252	2.68455	1
RIB	3	0.110351	0.456066	0.0664829	0.810432	0
R1P	2	0.00246666	0.499016	-0.0167309	1.85074	0
RL	2	0.51355	0.303783	0.468279	1.77237	1
O2e	7	1.12402	0.130502	0.550712	2.34243	2
GLCN15LACe	3	1.80735	0.0353536	1.38096	0.944044	1
H2O2e	7	1.12402	0.130502	0.550712	2.34243	2
H2Oe	19	1.03564	0.150186	0.299499	1.93145	7
GLCNTe	1	1.85389	0.0318775	2.47019	0	1
GLCN15LAC	1	1.85389	0.0318775	2.47019	0	1
GLCNT	6	1.2699	0.10206	0.676265	1.58457	2
GLAC	18	0.901415	0.183684	0.265849	1.76087	4
GALOL	5	-0.832126	0.797331	-0.517979	0.683143	0
GAL1P	2	0.281974	0.388982	0.248518	0.850479	0
UTP	7	-0.521049	0.698834	-0.28291	1.52975	0
UDPGAL	4	-0.0110329	0.504401	-0.0263463	0.960587	0

UDPG	10	-0.0669607	0.526693	-0.0472174	1.31543	1
G1P	4	1.3362	0.0907416	0.877187	0.822222	0
MELI	4	2.09155	0.0182395	1.38377	2.36271	2
GALN14LAC	3	0.769494	0.2208	0.577048	2.44836	1
GALNT	2	0.284466	0.388027	0.250883	1.22474	0
2D3DGALT	2	0.284466	0.388027	0.250883	1.22474	0
SOR	8	-0.267749	0.605554	-0.145761	0.718347	0
SOT	4	-0.239537	0.594655	-0.179594	0.590588	0
MAN6P	5	0.643875	0.259828	0.367246	0.612615	0
MAN	4	0.371336	0.355194	0.230093	0.675407	0
FRU	8	0.811616	0.208506	0.365815	0.88099	1
MNT6P	1	-0.570301	0.715763	-0.785061	0	0
MNT	4	0.776081	0.218851	0.501538	1.14267	1
F26P	1	0.880883	0.189191	1.16362	0	0
MAN1P	1	-0.184077	0.573023	-0.26643	0	0
GDPMAN	2	-0.604877	0.72737	-0.593091	0.461969	0
IDOL	3	-0.147997	0.558827	-0.13363	1.12937	0
UDP	17	-1.52376	0.936216	-0.513918	1.36505	1
TRE6P	3	0.341311	0.366435	0.245382	1.96662	1

TRE	3	-0.602575	0.726604	-0.485742	1.18829	0
MLT	5	0.784668	0.216324	0.451687	1.63522	2
MLTe	5	0.784668	0.216324	0.451687	1.63522	2
LACT	4	-0.230489	0.591144	-0.173526	2.04418	1
LACTe	4	-0.230489	0.591144	-0.173526	2.04418	1
GLACe	5	-0.215542	0.585328	-0.148186	1.77122	1
13GLUCAN	2	-1.18525	0.882041	-1.14386	0.480358	0
GA6P	4	0.369104	0.356025	0.228596	0.676231	0
NAGA6P	2	-0.200982	0.579644	-0.209801	0.222016	0
NAGA1P	2	-0.0224486	0.508955	-0.0403751	0.46162	0
UDPNAG	9	-0.855521	0.803869	-0.401104	1.00564	0
CHIT	13	0.561411	0.287259	0.189841	1.50764	3
NAG	5	2.15374	0.0156304	1.27278	1.59591	3
GLCN	3	0.440739	0.329701	0.322398	0.795701	0
13GLUCANe	12	-0.617363	0.731502	-0.257653	1.29048	2
GLYCOGEN	2	0.283833	0.388269	0.250282	1.35939	0
STARe	2	1.02203	0.153384	0.950818	0.120982	0
GLYCOGENe	2	1.02203	0.153384	0.950818	0.120982	0
AMYLSe	1	0.449948	0.326374	0.584952	0	0

AMYLPe	1	0.449948	0.326374	0.584952	0	0
CELLUe	8	-0.5707	0.715898	-0.289348	1.09417	1
CELLOBe	8	-0.5707	0.715898	-0.289348	1.09417	1
CELLOTe	4	-0.167949	0.566688	-0.131583	1.24728	1
MANNANe	6	1.02884	0.151777	0.544306	1.40888	2
MANe	7	0.94473	0.172398	0.459859	1.30539	2
PECTATEe	1	-0.429627	0.666267	-0.596161	0	0
GALUNTe	1	-0.429627	0.666267	-0.596161	0	0
ARABINe	5	0.0368962	0.485284	0.0032131	1.52183	1
LARABe	5	0.0368962	0.485284	0.0032131	1.52183	1
XYLANe	6	0.228065	0.409798	0.105953	0.910424	1
XYLe	6	0.228065	0.409798	0.105953	0.910424	1
H+_PO_mit	8	-1.25609	0.895458	-0.614194	0.983614	0
H+_PO	8	-1.25609	0.895458	-0.614194	0.983614	0
FERIm	5	-0.533739	0.703239	-0.339023	1.20193	0
FEROm	5	-0.533739	0.703239	-0.339023	1.20193	0
O2m	1	0.781892	0.217139	1.03069	0	0
K	1	0.390287	0.348162	0.504837	0	0
Km	1	0.390287	0.348162	0.504837	0	0

Ca	1	-0.214269	0.584831	-0.306973	0	0
Cam	1	-0.214269	0.584831	-0.306973	0	0
LLACm	6	0.364137	0.357878	0.18044	2.16481	1
LACm	1	-0.768685	0.77896	-1.05145	0	0
GLUm	10	0.360165	0.359362	0.133818	1.32911	1
ASPm	6	1.44224	0.0746177	0.770604	0.972765	1
ALAm	1	0.891597	0.186305	1.17801	0	0
ASN	5	-0.176008	0.569856	-0.124475	1.30846	0
SAM	12	-2.07015	0.980781	-0.819679	0.530467	0
HCYS	8	-0.206718	0.581885	-0.116835	1.00089	1
SAH	8	-1.8066	0.964588	-0.875117	0.651065	0
MET	4	-0.532661	0.702866	-0.37618	1.45304	1
TRNAm	4	0.759111	0.223893	0.490157	1.83293	1
ASPTRNAm	3	1.73774	0.0411283	1.32704	0.914981	1
TRNA	3	0.682111	0.247584	0.509362	0.696647	0
ASPTRNA	3	0.682111	0.247584	0.509362	0.696647	0
NH3	29	-0.835075	0.798162	-0.226422	1.23091	3
NAGLUm	2	-0.509725	0.694878	-0.502793	1.11147	0
NAGLUPm	1	-0.945376	0.827766	-1.28872	0	0

NAGLUSm	2	-1.28843	0.901202	-1.24177	0.0663893	0
NAORNm	2	-0.460257	0.677334	-0.455849	1.04508	0
ORNm	1	0.225182	0.410919	0.283132	0	0
CAP	5	1.07138	0.141999	0.62364	1.8489	1
ORN	5	3.41036	0.000324391	2.02643	2.62839	3
CITR	2	2.81689	0.00242459	2.65411	1.506	1
GLUGSAL	3	0.522869	0.300533	0.386015	2.88773	1
ARGSUCC	2	0.381181	0.351534	0.342664	1.76288	0
ARG	3	1.33674	0.0906532	1.01643	2.89304	1
PTRSC	2	-0.385342	0.650008	-0.384756	0.0992013	0
DSAM	2	-0.781878	0.782857	-0.761062	0.631379	0
SPRMD	3	-0.657381	0.744532	-0.528194	0.444169	0
5MTA	1	-0.219956	0.587047	-0.31461	0	0
SPRM	2	-0.267459	0.605442	-0.272886	0.0590056	0
GBAD	3	1.36452	0.0862024	1.03795	2.25993	1
GBAT	3	1.36452	0.0862024	1.03795	2.25993	1
UREA	3	0.891061	0.186448	0.671213	3.6355	1
ATRNA	1	-0.276607	0.608959	-0.390682	0	0
ALTRNA	1	-0.276607	0.608959	-0.390682	0	0

DAPRP	1	-0.759267	0.776154	-1.03881	0	0
SLF	1	-1.52025	0.935776	-2.06067	0	0
APS	2	-1.02409	0.847104	-0.990919	1.51286	0
PAPS	2	-0.800952	0.78842	-0.779164	1.2134	0
SER	11	-0.832259	0.797369	-0.355136	0.8771	0
ASER	5	-0.0822412	0.532773	-0.0682391	0.421284	0
H2S	5	0.30126	0.381608	0.161764	0.923391	1
RTHIO	4	0.475484	0.317221	0.29994	1.31084	0
OTHIO	4	0.475484	0.317221	0.29994	1.31084	0
H2SO3	2	-0.914858	0.819867	-0.887259	1.06053	0
GLUGSALm	3	0.711132	0.238501	0.531841	2.19512	1
P5Cm	4	1.51657	0.0646876	0.998153	2.02043	2
PHP	1	-1.246	0.893617	-1.6924	0	0
GLYm	2	-0.381056	0.648419	-0.380688	0.345274	0
GLY	11	-0.362443	0.641489	-0.165287	0.983039	1
GLX	2	-1.66308	0.951852	-1.59731	0.695383	0
BASP	2	-0.853169	0.803217	-0.828716	0.737277	0
ASPSA	2	-0.25967	0.602441	-0.265494	0.0592392	0
HSER	3	0.602955	0.273269	0.448049	0.741811	0

PHSER	2	1.04841	0.147225	0.975852	0.379571	0
THR	8	-0.771982	0.779938	-0.384748	1.07357	0
LLCT	5	-0.384662	0.649756	-0.249615	0.457792	0
OBUT	4	-1.72508	0.957743	-1.17588	0.784912	0
THRm	3	-2.03891	0.979271	-1.59831	0.0562146	0
NH3m	3	-2.03891	0.979271	-1.59831	0.0562146	0
OBUTm	4	-1.84604	0.967557	-1.25701	0.684151	0
PRPP	9	-0.082125	0.532726	-0.0555411	1.19037	1
PRBATP	3	0.08068	0.467848	0.0435002	1.18626	0
PRBAMP	2	-0.425581	0.664793	-0.422942	1.22842	0
PRFP	3	-0.140267	0.555775	-0.127643	1.00802	0
PRLP	2	0.0267547	0.489328	0.00631811	0.645783	0
DIMGP	2	0.169957	0.432522	0.142215	0.83797	0
IMACP	2	1.3108	0.0949621	1.22486	0.693123	1
HISOLP	2	0.243182	0.403932	0.211705	2.12594	1
HISOL	3	-0.895299	0.814686	-0.712483	1.003	0
HIS	4	-0.227924	0.590147	-0.171806	1.33119	0
AICAR	4	-0.205884	0.581559	-0.157025	1.26183	0
HTRNA	1	1.06626	0.143154	1.41254	0	0

HHTRNA	1	1.06626	0.143154	1.41254	0	0
MHIS	1	-0.919435	0.821066	-1.25388	0	0
OICAPm	1	-0.854099	0.803475	-1.16615	0	0
LEUm	1	-0.854099	0.803475	-1.16615	0	0
OMVALm	2	-0.630519	0.735822	-0.617425	0.776014	0
ILEm	1	-0.854099	0.803475	-1.16615	0	0
OMVAL	1	-0.854099	0.803475	-1.16615	0	0
ILE	1	-0.854099	0.803475	-1.16615	0	0
VAL	4	-1.71748	0.957054	-1.17079	1.01441	0
OICAP	3	0.817387	0.206854	0.614145	1.66613	1
LEU	1	-0.854099	0.803475	-1.16615	0	0
ABUTm	2	0.267544	0.394525	0.234823	0.661734	0
ACLACm	2	0.267544	0.394525	0.234823	0.661734	0
DHVALm	2	0.354159	0.36161	0.31702	0.545491	0
DHMVAm	2	0.354159	0.36161	0.31702	0.545491	0
OIVALm	2	-0.319684	0.625396	-0.322447	0.358852	0
IPPMALm	1	-0.414758	0.660841	-0.576194	0	0
CBHCAP	1	0.492711	0.311108	0.642375	0	0
IPPMAL	3	1.59566	0.0552824	1.21699	0.804067	1

PPMAL	1	0.492711	0.311108	0.642375	0	0
HCITm	1	1.04655	0.147655	1.38608	0	0
HACNm	2	0.183505	0.427201	0.155072	1.7409	0
HICITm	2	-0.163737	0.565031	-0.174456	1.27488	0
OXAm	1	0.555746	0.289192	0.72702	0	0
MICIT	1	0.718524	0.236217	0.945601	0	0
AKA	1	-0.52762	0.701119	-0.727748	0	0
AMA	4	-0.625345	0.734128	-0.438339	1.18348	0
AMASA	2	1.7333	0.0415216	1.6258	0.447654	1
SACP	2	0.810181	0.208918	0.749778	1.68654	1
LYS	3	0.359173	0.359733	0.259218	0.70225	0
LTRNA	2	0.66312	0.253627	0.610219	0.497092	0
LLTRNA	2	0.66312	0.253627	0.610219	0.497092	0
LYSm	2	0.66312	0.253627	0.610219	0.497092	0
LTRNAm	2	0.66312	0.253627	0.610219	0.497092	0
LLTRNAm	2	0.66312	0.253627	0.610219	0.497092	0
ADN	7	-0.526675	0.70079	-0.285761	1.70553	1
MTHPTGLU	1	-0.557284	0.711333	-0.767581	0	0
THPTGLU	1	-0.557284	0.711333	-0.767581	0	0

OAHSER	2	1.0882	0.138254	1.01361	0.975958	1
METH	1	1.28309	0.0997296	1.70372	0	1
OSLHSER	1	-0.261544	0.603163	-0.370454	0	0
CALH	1	-0.346647	0.635572	-0.484733	0	0
DPTH	1	-0.346647	0.635572	-0.484733	0	0
3DDAH7P	3	-1.12355	0.869399	-0.889286	0.986901	0
DQT	3	0.237747	0.406039	0.165162	1.28491	1
DHSK	2	-0.586068	0.721085	-0.575241	0.113059	0
QT	1	1.24009	0.107471	1.64597	0	1
SME	1	-0.354514	0.638523	-0.495297	0	0
SME5P	1	-0.354514	0.638523	-0.495297	0	0
3PSME	2	-0.35591	0.639046	-0.356825	0.195829	0
PHEN	2	-0.270194	0.606495	-0.275482	1.32038	0
PHPYR	2	2.02839	0.0212604	1.90584	1.46015	1
PHE	1	0.664725	0.253113	0.873358	0	0
4HPP	6	-0.03403	0.513573	-0.0375201	0.877749	0
TYR	4	0.558062	0.288401	0.355322	0.753312	0
AN	4	0.967215	0.166718	0.629723	1.31027	1
NPRAN	2	0.561786	0.287131	0.514055	2.31421	1

CPAD5P	1	-0.82147	0.794311	-1.12234	0	0
IGP	2	-0.113822	0.545311	-0.127088	1.40749	0
TRP	3	-0.94746	0.828298	-0.752886	1.4135	0
FKYN	3	-0.890732	0.813463	-0.708945	1.48912	0
KYN	4	0.413517	0.339614	0.258381	0.929324	0
HKYN	3	0.0389542	0.484463	0.0111799	0.963749	0
HAN	2	-0.0739712	0.529483	-0.0892693	1.34055	0
CMUSA	1	0.667114	0.25235	0.876567	0	0
AM6SA	2	-0.490663	0.688168	-0.484704	1.92513	0
AMUCO	1	-1.36036	0.913143	-1.84597	0	0
HOMOGEN	2	-1.26767	0.897542	-1.22207	1.10987	0
MACAC	2	-0.727544	0.766554	-0.709501	1.83475	0
FUACAC	2	0.679401	0.248442	0.625669	0.0534657	0
ACTAC	1	0.508425	0.305578	0.663476	0	0
TRPm	1	-1.49033	0.931931	-2.0205	0	0
TRPTRNAm	1	-1.49033	0.931931	-2.0205	0	0
PAD	3	0.75839	0.224109	0.568447	2.36205	1
PAC	3	0.75839	0.224109	0.568447	2.36205	1
IAD	3	0.75839	0.224109	0.568447	2.36205	1

IAC	3	0.75839	0.224109	0.568447	2.36205	1
ASPERMD	2	-0.693816	0.756101	-0.677493	0.631206	0
APRUT	1	-0.822578	0.794626	-1.12382	0	0
APROA	1	-0.822578	0.794626	-1.12382	0	0
GABAL	1	-0.822578	0.794626	-1.12382	0	0
ASPRM	2	-0.693816	0.756101	-0.677493	0.631206	0
GLUP	2	-0.752811	0.774218	-0.733479	0.894723	0
P5C	1	0.515873	0.302972	0.673477	0	0
PRO	1	0.515873	0.302972	0.673477	0	0
PHC	1	0.515873	0.302972	0.673477	0	0
HPRO	1	0.515873	0.302972	0.673477	0	0
PROm	1	1.79945	0.035974	2.39709	0	1
GABALm	5	0.163955	0.434883	0.0794162	2.40447	1
GABAm	5	0.163955	0.434883	0.0794162	2.40447	1
LACALm	5	0.163955	0.434883	0.0794162	2.40447	1
APROP	1	0.593975	0.276264	0.778355	0	0
TCOA	1	-0.506021	0.693579	-0.698744	0	0
GLP	1	-0.506021	0.693579	-0.698744	0	0
TGLP	1	-0.506021	0.693579	-0.698744	0	0

PEPD	1	-0.533754	0.703244	-0.735985	0	0
APEP	1	-0.533754	0.703244	-0.735985	0	0
GC	2	0.757377	0.224412	0.699668	1.3839	1
OGT	2	-0.0676427	0.526965	-0.0832636	0.672171	0
cAMP	3	0.0205007	0.491822	-0.00311399	0.751258	0
GMP	6	0.377321	0.352968	0.187657	1.63882	1
DGMP	2	0.521146	0.301133	0.475488	3.57017	1
DGDP	2	0.655516	0.256068	0.603003	0.927462	0
DATP	2	0.433286	0.332403	0.392111	0.629215	0
DADP	4	-0.0158652	0.506329	-0.0295871	1.01768	0
PRAM	1	-0.436189	0.66865	-0.604971	0	0
GAR	1	-0.436189	0.66865	-0.604971	0	0
FGAR	1	-0.906971	0.817789	-1.23715	0	0
FGAM	2	-0.950476	0.829065	-0.92106	0.447017	0
AIR	2	-0.323559	0.626864	-0.326125	0.394349	0
CAIR	2	-0.604386	0.727206	-0.592625	0.771238	0
SAICAR	3	-1.0155	0.845066	-0.805589	1.18497	0
PRFICA	1	0.834261	0.202067	1.10102	0	0
IMP	9	0.246075	0.402812	0.0911027	1.50209	1

ASUC	3	-1.24722	0.893842	-0.985078	1.29606	0
XMP	5	0.330185	0.37063	0.179112	1.83431	1
cdAMP	1	0.334023	0.369181	0.429285	0	0
DAMP	5	0.0501817	0.479989	0.0111811	1.93035	1
cIMP	1	0.334023	0.369181	0.429285	0	0
cGMP	1	0.334023	0.369181	0.429285	0	0
cCMP	1	0.334023	0.369181	0.429285	0	0
ATN	1	-0.619247	0.732123	-0.850786	0	0
ATT	2	-1.96979	0.975569	-1.88837	1.46737	0
UGC	2	-2.10405	0.982313	-2.01578	1.28719	0
CAASP	3	-0.298673	0.617405	-0.250343	0.887877	0
DOROA	1	-0.293368	0.61538	-0.413189	0	0
OROA	2	0.0909294	0.463774	0.0672189	0.6794	0
OMP	2	-0.0781117	0.53113	-0.0931986	0.906264	0
UMP	6	-0.267064	0.60529	-0.165085	1.72026	1
URA	6	-0.748922	0.773048	-0.42886	1.27054	1
CYTS	2	1.5719	0.0579864	1.47264	0.245122	1
URI	4	0.0532468	0.478768	0.0167634	2.12895	1
CYTD	4	0.0532468	0.478768	0.0167634	2.12895	1

DU	4	-0.25045	0.59888	-0.186913	2.23267	1
DR1P	1	-0.972694	0.834647	-1.3254	0	0
DT	3	-0.136608	0.55433	-0.124809	2.73022	1
THY	1	-0.972694	0.834647	-1.3254	0	0
DC	3	0.273147	0.39237	0.192583	2.5716	1
DTMP	4	-0.306844	0.620519	-0.224734	2.21715	1
DTDP	2	-0.569548	0.715508	-0.559565	0.716657	0
OTHIOm	1	0.566213	0.285624	0.741075	0	0
RTHIOm	1	0.566213	0.285624	0.741075	0	0
DUTP	3	1.36777	0.0856921	1.04047	1.20791	1
DUMP	5	0.550577	0.290962	0.311291	2.21862	2
DCMP	3	0.025758	0.489725	0.000958284	2.65492	1
DCDP	3	0.135507	0.446106	0.0859686	1.10999	0
CDP	4	-1.13318	0.87143	-0.77892	1.95282	0
PURI5P	3	-1.32891	0.908061	-1.04835	0.821195	0
AD	5	-1.41623	0.921646	-0.868295	0.536189	0
INS	5	-0.477135	0.683367	-0.305075	2.0448	1
DA	4	-0.656423	0.744224	-0.459182	2.32737	1
DIN	2	-1.44868	0.926287	-1.39385	0.0968045	0

HYXN	3	-1.28999	0.901473	-1.0182	0.654233	0
DG	4	-0.216452	0.585682	-0.164112	2.2306	1
GN	3	-1.02139	0.846465	-0.810152	0.529802	0
GSN	6	-0.325744	0.627691	-0.197207	1.76639	1
XAN	2	-0.818857	0.793566	-0.796155	0.748468	0
XTSINE	3	-0.136608	0.55433	-0.124809	2.73022	1
ITP	2	-0.0571336	0.522781	-0.0732907	0.0289619	0
IDP	2	-0.0571336	0.522781	-0.0732907	0.0289619	0
ITPm	1	-0.902084	0.816494	-1.23059	0	0
IDPm	1	-0.902084	0.816494	-1.23059	0	0
DGTP	3	0.240635	0.404919	0.1674	0.591136	0
DUDP	3	0.135507	0.446106	0.0859686	1.10999	0
DCTP	2	0.433286	0.332403	0.392111	0.629215	0
DTTP	1	-0.0249948	0.50997	-0.0528116	0	0
LCCA	4	-0.25154	0.599302	-0.187644	0.588428	0
ACOA	7	-0.667421	0.747748	-0.357082	0.900801	0
HACOA	3	-1.82495	0.965996	-1.43258	0.771946	0
OACOA	6	-1.8065	0.96458	-1.00779	1.08041	0
AACCOA	3	0.384207	0.350412	0.278609	0.825854	0

AACCOAm	2	0.0894061	0.46438	0.0657734	1.04512	0
ACACPm	2	0.265873	0.395168	0.233238	0.285179	0
MALACPm	2	0.265873	0.395168	0.233238	0.285179	0
C100ACPm	1	0.0378564	0.484901	0.0315862	0	0
ACPm	2	0.265873	0.395168	0.233238	0.285179	0
C120ACPm	1	0.0378564	0.484901	0.0315862	0	0
C140ACPm	1	0.0378564	0.484901	0.0315862	0	0
C141ACPm	1	0.0378564	0.484901	0.0315862	0	0
C160ACPm	1	0.0378564	0.484901	0.0315862	0	0
C161ACPm	1	0.0378564	0.484901	0.0315862	0	0
C180ACPm	1	0.0378564	0.484901	0.0315862	0	0
C181ACPm	1	0.0378564	0.484901	0.0315862	0	0
C182ACPm	1	0.0378564	0.484901	0.0315862	0	0
C150ACPm	1	0.0378564	0.484901	0.0315862	0	0
C162ACPm	1	0.0378564	0.484901	0.0315862	0	0
C170ACPm	1	0.0378564	0.484901	0.0315862	0	0
C183ACPm	1	0.0378564	0.484901	0.0315862	0	0
C200ACPm	1	0.0378564	0.484901	0.0315862	0	0
MALCOA	7	-0.670024	0.748579	-0.358401	0.913062	0

MALACP	4	0.412534	0.339974	0.257722	0.588854	0
ACACP	4	0.69378	0.24391	0.446342	0.87378	1
3OACPm	1	0.338197	0.367607	0.43489	0	0
C100ACP	1	0.121879	0.451497	0.144413	0	0
C120ACP	9	-0.376392	0.646687	-0.187023	0.876679	0
C140ACP	9	-0.376392	0.646687	-0.187023	0.876679	0
C141ACP	9	-0.376392	0.646687	-0.187023	0.876679	0
C160ACP	9	-0.376392	0.646687	-0.187023	0.876679	0
C161ACP	9	-0.376392	0.646687	-0.187023	0.876679	0
C180ACP	9	-0.376392	0.646687	-0.187023	0.876679	0
C181ACP	9	-0.376392	0.646687	-0.187023	0.876679	0
C182ACP	9	-0.376392	0.646687	-0.187023	0.876679	0
3HPACP	3	0.730682	0.232487	0.546985	1.04138	1
2HDACP	3	0.730682	0.232487	0.546985	1.04138	1
AACP	3	0.730682	0.232487	0.546985	1.04138	1
23DAACP	3	0.730682	0.232487	0.546985	1.04138	1
C150ACP	6	-0.111182	0.544264	-0.079754	1.07736	0
C162ACP	9	-0.376392	0.646687	-0.187023	0.876679	0
C170ACP	9	-0.376392	0.646687	-0.187023	0.876679	0

C183ACP	9	-0.376392	0.646687	-0.187023	0.876679	0
C200ACP	9	-0.376392	0.646687	-0.187023	0.876679	0
C140	1	-1.03807	0.850381	-1.41319	0	0
C160	1	-1.03807	0.850381	-1.41319	0	0
C180	1	-1.03807	0.850381	-1.41319	0	0
AGL3P	3	0.643666	0.259896	0.479583	1.28509	1
AT3P2	2	0.965585	0.167126	0.897255	1.50208	1
PA	5	-1.01408	0.844727	-0.627103	0.312904	0
PAm	1	-0.280993	0.610642	-0.396571	0	0
CTPm	2	-0.216673	0.585768	-0.224691	0.243074	0
CDPDGm	3	0.0396798	0.484174	0.011742	0.578288	0
CDPDG	2	0.262269	0.396557	0.229817	0.885847	0
PS	3	1.48035	0.0693902	1.12767	1.86353	1
CMPm	3	-0.197663	0.578346	-0.172101	0.813025	0
PSm	1	0.197706	0.421638	0.246235	0	0
PE	6	0.91635	0.179742	0.482728	1.45099	1
PEm	1	0.197706	0.421638	0.246235	0	0
PMME	2	-0.95262	0.829609	-0.923094	0.622096	0
PDME	1	-1.00068	0.841509	-1.36298	0	0

PC	3	-0.563496	0.713451	-0.455471	1.0408	0
CHO	1	0.259072	0.39779	0.32864	0	0
PCHO	2	-0.377904	0.647249	-0.377697	0.998911	0
CDPCHO	2	-0.192455	0.576307	-0.201708	1.2478	0
DAGLY	11	-0.566735	0.714553	-0.24784	1.01203	0
PETHM	2	-0.807048	0.79018	-0.784948	0.957585	0
CDPETN	2	0.321884	0.37377	0.286392	0.557518	0
MI1P	2	-1.33666	0.909333	-1.28754	1.47229	0
MYOI	2	-0.755678	0.775079	-0.736199	2.252	0
PINS	7	-0.271704	0.607075	-0.156557	0.833645	0
PINSP	4	-0.241616	0.595461	-0.180989	0.845448	0
PINS4P	3	-0.285322	0.612301	-0.240001	0.854799	0
D45PI	3	-0.649211	0.741899	-0.521866	1.24488	0
TPI	2	-0.191041	0.575753	-0.200367	1.57457	0
GL3Pm	1	0.515873	0.302972	0.673477	0	0
PGPm	2	-0.101297	0.540342	-0.115201	1.11536	0
PGm	2	-0.583473	0.720213	-0.572779	0.468245	0
CLm	1	-0.165645	0.565782	-0.24168	0	0
DGPP	2	-0.79395	0.786388	-0.772518	0.421939	0

LPC	1	-0.495077	0.689727	-0.684048	0	0
LPE	1	-0.495077	0.689727	-0.684048	0	0
CDPm	2	-0.507262	0.694015	-0.500456	0.633065	0
PALCOA	2	-0.457958	0.676509	-0.453667	0.644233	0
DHSPH	3	-0.861639	0.805557	-0.68641	0.608298	0
SPH	4	-0.766206	0.778223	-0.532809	1.93937	1
PSPH	3	0.947529	0.171685	0.714952	1.89192	1
C260COA	1	0.891597	0.186305	1.17801	0	0
CER2	2	2.3203	0.0101622	2.18286	1.42108	1
CER3	2	1.09268	0.137268	1.01786	3.06864	1
IPC	2	-1.07146	0.858019	-1.03587	0.164219	0
MIPC	2	-1.07146	0.858019	-1.03587	0.164219	0
MIP2C	1	-0.843556	0.800541	-1.15199	0	0
DHSP	3	-2.0298	0.978811	-1.59125	0.311152	0
PHSP	1	-1.00256	0.841963	-1.36551	0	0
C16A	1	-1.07447	0.858693	-1.46206	0	0
H3MCOA	3	1.45836	0.0723709	1.11064	0.633783	1
MVL	3	-0.296493	0.616573	-0.248654	2.75712	1
PMVL	2	-0.456048	0.675822	-0.451855	4.13195	1

PPMVL	2	1.43849	0.075147	1.34604	1.58935	1
IPPP	3	-0.801295	0.78852	-0.639668	0.775365	0
DMPP	2	-1.10806	0.866082	-1.0706	0.29691	0
GPP	1	-0.626597	0.734538	-0.860655	0	0
FPP	1	-0.626597	0.734538	-0.860655	0	0
S23E	1	-1.41504	0.921472	-1.9194	0	0
LNST	4	0.190291	0.424541	0.108673	2.415	1
IGST	5	2.13934	0.016204	1.26415	1.93984	2
DMZYMST	5	2.85775	0.0021333	1.69501	1.60422	3
IMZYMST	4	1.56993	0.0582155	1.03394	1.94419	2
IIMZYMST	2	-1.95371	0.974632	-1.87311	2.12199	0
MZYMST	4	0.451272	0.325897	0.283702	2.97411	2
IZYMST	4	1.56993	0.0582155	1.03394	1.94419	2
IIZYMST	2	-1.95371	0.974632	-1.87311	2.12199	0
ZYMST	2	-2.37689	0.99127	-2.2747	1.55405	0
FEST	2	-0.112564	0.544812	-0.125893	1.48482	0
EPST	3	2.83506	0.00229083	2.17702	1.34424	2
ERTROL	3	2.343	0.00956477	1.79587	1.91717	2
ERTEOL	2	0.522201	0.300765	0.476489	0.984155	0

ERGOST	1	0.887415	0.187428	1.17239	0	0
TAGLY	5	-0.176195	0.56993	-0.124588	1.19825	0
MAGLY	4	-0.505608	0.693434	-0.358037	1.24542	0
PHACAL	6	1.05002	0.146855	0.5559	2.44692	2
PHAC	6	-0.0861102	0.534311	-0.0660294	2.17993	1
PHACCOA	2	-1.02393	0.847066	-0.990765	0.279318	0
LLDACV	2	0.101495	0.459579	0.0772458	1.73078	0
IPN	2	0.0795373	0.468303	0.056408	1.76024	0
PENG	1	-0.870574	0.808007	-1.18827	0	0
NOR	4	-0.223885	0.588577	-0.169097	2.17075	1
AVN	5	-0.482109	0.685136	-0.308058	1.89974	1
HAVN	4	-1.25019	0.894386	-0.8574	0.573718	0
AVF	2	-1.0043	0.842384	-0.97214	1.02157	0
VHA	2	-0.842675	0.800295	-0.818758	1.23849	0
VERB	3	-1.67376	0.952911	-1.31547	1.1381	0
VERA	2	-0.78024	0.782375	-0.759509	0.857911	0
DMST	2	-0.78024	0.782375	-0.759509	0.857911	0
DHDMST	2	-0.78024	0.782375	-0.759509	0.857911	0
ST	1	-0.670607	0.748764	-0.919753	0	0

DHST	1	-0.670607	0.748764	-0.919753	0	0
OMST	3	0.467523	0.320063	0.343145	2.49005	1
DHOMST	3	0.467523	0.320063	0.343145	2.49005	1
AFB1	2	1.04708	0.147531	0.974594	3.1636	1
AFG1	2	1.04708	0.147531	0.974594	3.1636	1
AFB2	2	1.04708	0.147531	0.974594	3.1636	1
AFG2	2	1.04708	0.147531	0.974594	3.1636	1
HNO3	3	-0.827268	0.795957	-0.659786	1.24785	0
HNO2	2	2.1489	0.0158213	2.0202	1.78397	1
NH4OH	1	2.45818	0.00698206	3.28165	0	1
UREAC	1	0.457981	0.323483	0.595738	0	0
ACNL	1	0.593975	0.276264	0.778355	0	0
INAC	1	0.593975	0.276264	0.778355	0	0
NH3e	2	0.315122	0.376335	0.279974	2.06118	1
HNO3e	2	-1.42255	0.922567	-1.36905	0.30973	0
FRUe	1	-0.0205353	0.508192	-0.0468234	0	0
SORe	1	-0.0205353	0.508192	-0.0468234	0	0

#Results for Up-regulated only genes

#Feature	Number of neighbors	Z-score	P-value	Average Z	StdDev Z	Significance count
DGLCe	2	-0.567436	0.714791	-0.55756	0.536435	0
GLCe	13	-0.3062	0.620274	-0.132615	1.2749	2
DGLC	2	-0.464448	0.678837	-0.459826	1.6793	0
GLC	12	1.12701	0.129869	0.417177	1.43551	4
bDGLC	3	1.1175	0.13189	0.846609	1.2291	1
ATP	59	0.345035	0.365034	0.0413961	1.14587	6
ADP	40	-0.191535	0.575947	-0.0592864	1.18531	5
G6P	6	-0.426221	0.665027	-0.252209	1.25334	0
bDG6P	3	-0.523262	0.699604	-0.424307	1.49336	0
H2O	41	0.708143	0.239428	0.129321	1.51643	7
PI	34	-0.153789	0.561112	-0.0540646	1.28769	4
F6P	9	-0.551439	0.709334	-0.265236	0.848846	0
FDP	2	-0.852939	0.803153	-0.828498	1.13889	0
S7P	5	-0.374365	0.645934	-0.243439	0.522058	0
S17P	2	-0.852939	0.803153	-0.828498	1.13889	0
T3P2	3	-0.651657	0.742689	-0.52376	1.90698	1
T3P1	9	0.625957	0.265672	0.260838	1.66633	2
E4P	7	-1.37243	0.915036	-0.714341	0.87163	0

NAD	42	0.834673	0.201951	0.153685	1.48966	7
13PDG	4	-0.715233	0.762768	-0.498624	1.5595	1
NADH	42	0.834673	0.201951	0.153685	1.48966	7
3PG	3	-0.161489	0.564146	-0.144081	2.08173	1
2PG	2	-1.34067	0.909986	-1.29134	0.877432	0
23PDG	2	-1.34067	0.909986	-1.29134	0.877432	0
PEP	4	-1.67832	0.953357	-1.14452	0.769429	0
PYR	11	0.0095273	0.496199	-0.0149776	1.27963	1
CO2	21	-0.108745	0.543298	-0.0505642	0.970478	2
OA	7	0.319572	0.374646	0.143066	1.16225	1
ATPm	14	-0.245081	0.596803	-0.106575	1.28162	2
PYRm	4	0.381515	0.351411	0.236919	1.22689	1
CO2m	6	0.310007	0.378278	0.150809	0.966022	1
ADPm	8	-1.16204	0.877391	-0.569622	1.0046	1
PIm	7	-0.579123	0.718747	-0.312338	1.11901	1
OAm	4	0.504964	0.306792	0.319711	1.38898	1
GTP	7	-1.28315	0.90028	-0.669096	1.32838	0
GDP	5	-1.07232	0.858212	-0.662037	1.7238	0
NADP	30	-0.781429	0.782725	-0.209808	1.35182	2

D6PGL	1	-0.22628	0.589508	-0.323102	0	0
NADPH	30	-0.781429	0.782725	-0.209808	1.35182	2
D6PGC	3	0.160433	0.43627	0.105276	1.69204	0
RL5P	1	1.29636	0.0974254	1.72153	0	1
XUL5P	5	2.00118	0.0226866	1.18128	1.78635	2
R5P	10	0.426594	0.334838	0.161974	0.800124	0
ACTP	2	1.35394	0.0878785	1.26579	3.46938	1
ACCOAm	9	1.78906	0.0368028	0.780525	0.987155	2
H2Om	10	0.279983	0.389745	0.0998337	1.81733	2
CITm	4	0.342584	0.365956	0.21081	0.804844	0
COAm	10	1.5252	0.0636042	0.627616	1.04882	2
ACOm	3	-0.174873	0.56941	-0.154448	0.413739	0
ICITm	4	-0.160978	0.563945	-0.126908	0.342277	0
NADm	12	1.89907	0.0287779	0.715854	2.00948	4
AKGm	4	-0.643499	0.74005	-0.450514	0.545527	0
NADHm	12	1.89907	0.0287779	0.715854	2.00948	4
ICIT	1	-0.018648	0.507439	-0.0442891	0	0
AKG	10	1.19797	0.115465	0.488918	1.43825	2
NADPm	14	0.310842	0.37796	0.0925113	1.73197	2

NADPHm	14	0.310842	0.37796	0.0925113	1.73197	2
ICITg	2	0.782578	0.216938	0.723583	1.08594	0
NADPg	1	-0.018648	0.507439	-0.0442891	0	0
AKGg	1	-0.018648	0.507439	-0.0442891	0	0
CO2g	1	-0.018648	0.507439	-0.0442891	0	0
NADPHg	1	-0.018648	0.507439	-0.0442891	0	0
LIPOm	1	-0.0154476	0.506162	-0.0399914	0	0
SUCDLIP	1	-0.0154476	0.506162	-0.0399914	0	0
SUCCOAm	1	-0.543125	0.706478	-0.748568	0	0
GDPm	2	-1.02268	0.84677	-0.989577	0.340838	0
GTPm	2	-1.02268	0.84677	-0.989577	0.340838	0
SUCCm	3	-0.494189	0.689414	-0.401787	0.306389	0
Qm	4	-0.42493	0.664556	-0.30393	0.356497	0
FUMm	3	-0.64706	0.741204	-0.5202	0.509046	0
QH2m	4	-0.42493	0.664556	-0.30393	0.356497	0
FADH2m	3	-0.801735	0.788647	-0.640009	0.314139	0
FADm	3	-0.801735	0.788647	-0.640009	0.314139	0
FUM	5	-0.843165	0.800432	-0.5246	1.06768	0
SUCC	4	0.440972	0.329617	0.276794	0.938201	0

MALm	1	-0.80767	0.79036	-1.1038	0	0
MAL	2	-0.056972	0.522716	-0.0731373	1.45758	0
MALg	2	1.40732	0.079667	1.31645	0.576848	1
NADg	1	0.690937	0.244802	0.908557	0	0
OAg	1	0.690937	0.244802	0.908557	0	0
NADHg	1	0.690937	0.244802	0.908557	0	0
SUCCg	1	1.12502	0.13029	1.49146	0	0
GLXg	2	1.71443	0.0432247	1.6079	0.164676	1
ACCOAg	2	1.4453	0.0741867	1.3525	0.525868	1
H2Og	1	1.29845	0.0970658	1.72434	0	1
COAg	2	1.4453	0.0741867	1.3525	0.525868	1
Hm	7	-0.0786702	0.531353	-0.0587389	2.07768	1
COA	21	1.98917	0.0233412	0.562671	0.919092	3
ACCOA	15	1.22553	0.110189	0.405177	1.20026	3
CAR	2	0.977553	0.164148	0.908611	0.985047	0
ACAR	2	0.977553	0.164148	0.908611	0.985047	0
ACARm	2	0.648521	0.258324	0.596366	0.543464	0
CARm	2	0.648521	0.258324	0.596366	0.543464	0
ACARg	2	0.648521	0.258324	0.596366	0.543464	0

CARg	2	0.648521	0.258324	0.596366	0.543464	0
OXAL	2	-0.193589	0.576751	-0.202785	0.0236356	0
AC	12	0.854042	0.196541	0.311575	1.75476	3
FOR	3	-0.0813161	0.532405	-0.0819801	0.667174	0
FORm	1	-0.390839	0.652042	-0.544075	0	0
METHOL	3	1.94284	0.0260176	1.48591	1.364	1
FALD	6	1.33927	0.0902407	0.71424	1.23499	1
ACAL	8	1.25635	0.104495	0.576601	2.13022	2
RGT	4	0.297653	0.382984	0.180676	1.41962	1
FGT	2	0.419041	0.337593	0.378592	0.374265	0
H+	13	-0.954092	0.829981	-0.37341	1.55794	1
HCIT	1	0.776251	0.2188	1.02312	0	0
MTHGXL	2	-1.09625	0.863515	-1.0594	0.875354	0
LACAL	4	0.0162955	0.493499	-0.00801833	2.70088	1
LAC	3	0.18547	0.42643	0.124669	3.29852	1
LGT	1	-1.26112	0.896368	-1.71271	0	0
LLAC	2	1.0033	0.157859	0.933043	0.0346292	0
PROP	2	1.6712	0.047341	1.56687	1.03466	1
AMP	25	1.09817	0.136064	0.275397	1.16989	2

PPI	31	1.56266	0.0590661	0.357079	1.04545	3
PROPCOA	3	1.93535	0.0264734	1.48011	0.74689	1
2MCIT	1	0.987348	0.161736	1.30658	0	0
GLU	20	0.548301	0.291743	0.145456	1.2085	3
SUCCSAL	2	0.300412	0.381931	0.266015	1.33199	0
METTHF	5	-1.49821	0.932961	-0.917463	0.80014	0
METHF	2	-0.913087	0.819402	-0.885578	0.482959	0
METTHFm	5	-0.32652	0.627985	-0.214745	0.867827	0
MTHFm	2	0.716771	0.236758	0.661134	0.420373	0
METHFm	2	-0.913087	0.819402	-0.885578	0.482959	0
FTHFm	1	-0.390839	0.652042	-0.544075	0	0
FTHF	2	0.313537	0.376936	0.27847	1.16325	0
THFm	2	-0.595776	0.724338	-0.584454	0.0571053	0
AHTD	2	-1.26405	0.896893	-1.21863	0.776338	0
DHP	1	-1.30199	0.90354	-1.76759	0	0
CHOR	2	0.655067	0.256212	0.602577	0.0786173	0
GLN	9	-0.708609	0.760716	-0.335462	0.664822	0
DHF	1	-0.56921	0.715393	-0.783595	0	0
THF	6	-0.644655	0.740425	-0.371783	1.06038	0

MTHF	1	-0.557284	0.711333	-0.767581	0	0
THFG	1	0.0236491	0.490566	0.0125084	0	0
OIVAL	1	-1.52046	0.935802	-2.06095	0	0
AKP	1	-1.52046	0.935802	-2.06095	0	0
PANT	1	-1.36857	0.914433	-1.85699	0	0
AKPm	1	0.537665	0.295404	0.70274	0	0
PANTm	1	0.537665	0.295404	0.70274	0	0
bALA	1	-1.36857	0.914433	-1.85699	0	0
PNTO	1	-1.36857	0.914433	-1.85699	0	0
CTP	5	-1.00015	0.841382	-0.618754	1.60772	0
CYS	3	-0.348224	0.636164	-0.288724	0.369969	0
CMP	4	2.04463	0.0204458	1.3523	1.15769	1
ASP	11	0.039248	0.484346	-0.00296776	1.20264	0
PAP	2	-1.50763	0.934175	-1.44979	0.264993	0
ACP	3	0.787605	0.215464	0.591076	1.05578	1
ALA	2	-0.0739712	0.529483	-0.0892693	1.34055	0
DTB	1	-0.479782	0.684309	-0.66351	0	0
BT	1	-0.479782	0.684309	-0.66351	0	0
ETH	3	1.94284	0.0260176	1.48591	1.364	1

ETHm	3	1.94284	0.0260176	1.48591	1.364	1
ACALm	6	1.40321	0.0802766	0.749242	2.45148	2
ACm	7	1.09422	0.136928	0.535613	2.29386	3
AMPm	7	0.554373	0.289662	0.26205	1.46546	1
PPIm	8	0.955484	0.169667	0.434003	1.26198	1
ACTPm	1	-0.869946	0.807835	-1.18743	0	0
GLYN	10	0.14984	0.440445	0.0446728	0.917192	1
GL	11	0.979427	0.163685	0.37695	1.16195	1
GLYAL	7	0.625751	0.265739	0.298219	1.47087	1
O2	9	-2.2745	0.988532	-1.03512	0.648382	0
H2O2	5	-1.1296	0.870678	-0.696391	0.585444	0
GL3P	3	-0.348911	0.636422	-0.289256	1.15027	0
TAR	1	0.603252	0.27317	0.790812	0	0
OXGLY	1	0.603252	0.27317	0.790812	0	0
G	1	1.61577	0.0530717	2.15045	0	1
E	5	-0.832126	0.797331	-0.517979	0.683143	0
EOL	5	-0.832126	0.797331	-0.517979	0.683143	0
LXUL	5	0.233633	0.407635	0.121205	1.95356	1
XOL	5	0.0749469	0.470128	0.0260339	0.563938	0

XUL	6	0.792585	0.21401	0.414977	1.078	1
AOL	5	0.0749469	0.470128	0.0260339	0.563938	0
XYL	3	0.280958	0.389371	0.198633	0.554325	0
ARAB	1	-0.315408	0.623774	-0.442784	0	0
ARABLAC	1	-0.315408	0.623774	-0.442784	0	0
LAOL	3	0.485736	0.313577	0.357252	2.68455	1
RIB	2	0.566784	0.28543	0.518798	0.293371	0
R1P	2	0.00246666	0.499016	-0.0167309	1.85074	0
RL	1	1.29636	0.0974254	1.72153	0	1
O2e	1	-1.01529	0.845017	-1.38261	0	0
GLCN15LACe	1	1.85389	0.0318775	2.47019	0	1
H2O2e	1	-1.01529	0.845017	-1.38261	0	0
H2Oe	11	0.107405	0.457234	0.0245739	1.50571	3
GLCNTe	1	1.85389	0.0318775	2.47019	0	1
GLCN15LAC	1	1.85389	0.0318775	2.47019	0	1
GLCNT	4	1.06679	0.143033	0.696506	1.81848	1
GLAC	12	-0.582199	0.719784	-0.24405	1.1128	1
GALOL	5	-0.832126	0.797331	-0.517979	0.683143	0
GAL1P	2	0.281974	0.388982	0.248518	0.850479	0

UTP	6	-0.360692	0.640835	-0.216338	1.66461	0
UDPGAL	2	-0.22705	0.589807	-0.234538	0.167334	0
UDPG	6	-0.759472	0.776215	-0.434635	1.01576	0
G1P	3	1.0131	0.155506	0.765745	0.969307	0
MELI	2	1.1626	0.122495	1.08422	1.86494	1
GALN14LAC	1	0.152555	0.439375	0.185606	0	0
GALNT	2	0.284466	0.388027	0.250883	1.22474	0
2D3DGALT	2	0.284466	0.388027	0.250883	1.22474	0
SOR	5	-0.257457	0.601587	-0.173324	0.865579	0
SOT	2	-0.225286	0.589122	-0.232865	0.658879	0
MAN6P	3	0.331776	0.370029	0.237996	0.803712	0
MAN	2	0.342019	0.366168	0.305499	1.12453	0
FRU	5	0.763626	0.222545	0.439067	1.14375	1
MNT	3	0.706317	0.239996	0.528112	1.39797	1
GDPMAN	1	-0.670607	0.748764	-0.919753	0	0
IDOL	3	-0.147997	0.558827	-0.13363	1.12937	0
UDP	13	-2.17078	0.985026	-0.825602	1.17258	0
TRE6P	2	-0.900226	0.816	-0.873373	0.474894	0
TRE	3	-0.602575	0.726604	-0.485742	1.18829	0

MLT	4	1.45987	0.0721635	0.960123	1.35716	2
MLTe	4	1.45987	0.0721635	0.960123	1.35716	2
LACT	3	-1.50001	0.933194	-1.18088	0.423573	0
LACTe	3	-1.50001	0.933194	-1.18088	0.423573	0
GLACe	3	-1.50001	0.933194	-1.18088	0.423573	0
13GLUCAN	2	-1.18525	0.882041	-1.14386	0.480358	0
GA6P	3	0.264728	0.395609	0.186062	0.821632	0
NAGA6P	2	-0.200982	0.579644	-0.209801	0.222016	0
NAGA1P	2	-0.0224486	0.508955	-0.0403751	0.46162	0
UDPNAG	8	-1.22148	0.889047	-0.597791	0.870558	0
CHIT	8	-0.805088	0.789616	-0.400439	1.21167	1
NAG	1	1.40309	0.0802947	1.86485	0	1
GLCN	2	0.342019	0.366168	0.305499	1.12453	0
13GLUCANe	5	-1.27355	0.898589	-0.782725	0.522461	0
STARe	2	1.02203	0.153384	0.950818	0.120982	0
GLYCOGENe	2	1.02203	0.153384	0.950818	0.120982	0
CELLUe	3	-0.451115	0.674047	-0.368422	0.501199	0
CELLOBe	3	-0.451115	0.674047	-0.368422	0.501199	0
CELLOTe	2	-0.567436	0.714791	-0.55756	0.536435	0

MANNANe	4	0.348377	0.363778	0.214695	1.55381	1
MANe	4	0.348377	0.363778	0.214695	1.55381	1
PECTATEe	1	-0.429627	0.666267	-0.596161	0	0
GALUNTe	1	-0.429627	0.666267	-0.596161	0	0
ARABINe	4	0.2592	0.39774	0.154888	1.71306	1
LARABe	4	0.2592	0.39774	0.154888	1.71306	1
XYLANe	2	0.0631853	0.474809	0.0408902	0.689642	0
XYLe	2	0.0631853	0.474809	0.0408902	0.689642	0
FERIm	1	0.524872	0.299836	0.685561	0	0
FEROm	1	0.524872	0.299836	0.685561	0	0
LLACm	4	0.297866	0.382903	0.180819	2.79352	1
GLUm	6	0.0834927	0.46673	0.026813	1.56605	1
ASPm	3	0.528018	0.298743	0.390004	0.877202	0
ASN	3	-0.364013	0.642076	-0.300954	1.81815	0
SAM	8	-1.71983	0.957269	-0.833992	0.578342	0
HCYS	5	-0.570257	0.715748	-0.360925	0.859295	0
SAH	5	-1.3312	0.908439	-0.817299	0.800655	0
MET	3	-1.35618	0.91248	-1.06948	0.532	0
TRNAm	3	-0.11643	0.546344	-0.109179	1.6983	0

ASPTRNAm	2	0.912081	0.180863	0.84648	0.537349	0
TRNA	2	0.912081	0.180863	0.84648	0.537349	0
ASPTRNA	2	0.912081	0.180863	0.84648	0.537349	0
NH3	16	-1.25948	0.896071	-0.440662	1.10694	1
NAGLUm	2	-0.509725	0.694878	-0.502793	1.11147	0
NAGLUPm	1	-0.945376	0.827766	-1.28872	0	0
NAGLUSm	2	-1.28843	0.901202	-1.24177	0.0663893	0
NAORNm	2	-0.460257	0.677334	-0.455849	1.04508	0
ORNm	1	0.225182	0.410919	0.283132	0	0
CAP	4	-0.195713	0.577583	-0.150204	0.752105	0
ORN	4	2.41887	0.00778441	1.60329	2.83153	2
CITR	1	1.19782	0.115494	1.58921	0	0
GLUGSAL	3	0.522869	0.300533	0.386015	2.88773	1
ARGSUCC	2	0.381181	0.351534	0.342664	1.76288	0
ARG	3	1.33674	0.0906532	1.01643	2.89304	1
PTRSC	2	-0.385342	0.650008	-0.384756	0.0992013	0
DSAM	2	-0.781878	0.782857	-0.761062	0.631379	0
SPRMD	1	-0.219956	0.587047	-0.31461	0	0
5MTA	1	-0.219956	0.587047	-0.31461	0	0

SPRM	1	-0.219956	0.587047	-0.31461	0	0
UREA	2	2.62267	0.00436224	2.4698	2.65032	1
ATRNA	1	-0.276607	0.608959	-0.390682	0	0
ALTRNA	1	-0.276607	0.608959	-0.390682	0	0
APS	1	0.0730443	0.470885	0.0788374	0	0
PAPS	2	-0.800952	0.78842	-0.779164	1.2134	0
SER	6	-0.653038	0.743134	-0.376372	1.16456	0
ASER	2	0.230709	0.40877	0.199868	0.174847	0
H2S	2	-0.0121059	0.504829	-0.0305601	0.151028	0
RTHIO	4	0.475484	0.317221	0.29994	1.31084	0
OTHIO	4	0.475484	0.317221	0.29994	1.31084	0
H2SO3	2	-0.914858	0.819867	-0.887259	1.06053	0
GLUGSALm	3	0.711132	0.238501	0.531841	2.19512	1
P5Cm	4	1.51657	0.0646876	0.998153	2.02043	2
GLYm	1	-0.45098	0.673998	-0.624834	0	0
GLY	8	0.0419857	0.483255	0.00104075	0.880118	1
GLX	1	-0.809007	0.790744	-1.1056	0	0
BASP	1	-0.214574	0.58495	-0.307383	0	0
ASPSA	2	-0.25967	0.602441	-0.265494	0.0592392	0

HSER	3	0.602955	0.273269	0.448049	0.741811	0
PHSER	2	1.04841	0.147225	0.975852	0.379571	0
THR	7	-0.942987	0.827156	-0.496723	1.10798	0
LLCT	2	0.169473	0.432712	0.141756	0.0926638	0
OBUT	3	-1.69929	0.955367	-1.33524	0.878499	0
THRm	1	-1.13716	0.872264	-1.54625	0	0
NH3m	1	-1.13716	0.872264	-1.54625	0	0
OBUTm	2	-0.917401	0.820534	-0.889672	0.928543	0
PRPP	5	0.773884	0.2195	0.445219	1.26032	1
PRBATP	3	0.08068	0.467848	0.0435002	1.18626	0
PRBAMP	2	-0.425581	0.664793	-0.422942	1.22842	0
PRFP	3	-0.140267	0.555775	-0.127643	1.00802	0
PRLP	2	0.0267547	0.489328	0.00631811	0.645783	0
DIMGP	2	0.169957	0.432522	0.142215	0.83797	0
IMACP	2	1.3108	0.0949621	1.22486	0.693123	1
HISOLP	1	1.29148	0.0982692	1.71497	0	1
HISOL	2	-0.425581	0.664793	-0.422942	1.22842	0
HIS	3	0.268375	0.394205	0.188887	1.37022	0
AICAR	4	-0.205884	0.581559	-0.157025	1.26183	0

HTRNA	1	1.06626	0.143154	1.41254	0	0
HHTRNA	1	1.06626	0.143154	1.41254	0	0
OMVALm	1	-0.0368273	0.514689	-0.0687006	0	0
VAL	1	0.0567719	0.477363	0.0569865	0	0
OICAP	2	1.60526	0.0542187	1.50429	0.893185	1
ABUTm	2	0.267544	0.394525	0.234823	0.661734	0
ACLACm	2	0.267544	0.394525	0.234823	0.661734	0
DHVALm	2	0.354159	0.36161	0.31702	0.545491	0
DHMVAm	2	0.354159	0.36161	0.31702	0.545491	0
OIVALm	2	-0.319684	0.625396	-0.322447	0.358852	0
IPPMALm	1	-0.414758	0.660841	-0.576194	0	0
CBHCAP	1	0.492711	0.311108	0.642375	0	0
IPPMAL	3	1.59566	0.0552824	1.21699	0.804067	1
PPMAL	1	0.492711	0.311108	0.642375	0	0
HCITm	1	1.04655	0.147655	1.38608	0	0
HACNm	2	0.183505	0.427201	0.155072	1.7409	0
HICITm	2	-0.163737	0.565031	-0.174456	1.27488	0
OXAm	1	0.555746	0.289192	0.72702	0	0
MICIT	1	0.718524	0.236217	0.945601	0	0

AMA	1	0.989342	0.161248	1.30926	0	0
AMASA	2	1.7333	0.0415216	1.6258	0.447654	1
SACP	2	0.810181	0.208918	0.749778	1.68654	1
LYS	2	-0.0768815	0.530641	-0.0920311	0.496039	0
LTRNA	1	0.207004	0.418003	0.258722	0	0
LLTRNA	1	0.207004	0.418003	0.258722	0	0
LYSm	1	0.207004	0.418003	0.258722	0	0
LTRNAm	1	0.207004	0.418003	0.258722	0	0
LLTRNAm	1	0.207004	0.418003	0.258722	0	0
ADN	4	0.903931	0.183016	0.587281	1.7894	1
MTHPTGLU	1	-0.557284	0.711333	-0.767581	0	0
THPTGLU	1	-0.557284	0.711333	-0.767581	0	0
OAHSER	1	0.255247	0.399266	0.323503	0	0
OSLHSER	1	-0.261544	0.603163	-0.370454	0	0
3DDAH7P	2	-1.12458	0.869616	-1.08628	1.30962	0
DQT	1	-0.473583	0.682102	-0.655186	0	0
DHSK	1	-0.473583	0.682102	-0.655186	0	0
PHEN	1	0.504473	0.306965	0.658168	0	0
PHPYR	1	0.664725	0.253113	0.873358	0	0

PHE	1	0.664725	0.253113	0.873358	0	0
4HPP	2	0.154893	0.438453	0.127919	1.05421	0
TYR	2	0.154893	0.438453	0.127919	1.05421	0
AN	4	0.967215	0.166718	0.629723	1.31027	1
NPRAN	2	0.561786	0.287131	0.514055	2.31421	1
CPAD5P	1	-0.82147	0.794311	-1.12234	0	0
IGP	2	-0.113822	0.545311	-0.127088	1.40749	0
TRP	1	0.660854	0.254353	0.86816	0	0
KYN	2	-0.0739712	0.529483	-0.0892693	1.34055	0
HKYN	2	-0.0739712	0.529483	-0.0892693	1.34055	0
HAN	2	-0.0739712	0.529483	-0.0892693	1.34055	0
CMUSA	1	0.667114	0.25235	0.876567	0	0
AM6SA	2	-0.490663	0.688168	-0.484704	1.92513	0
AMUCO	1	-1.36036	0.913143	-1.84597	0	0
HOMOGEN	1	-1.48018	0.930587	-2.00687	0	0
MACAC	2	-0.727544	0.766554	-0.709501	1.83475	0
FUACAC	2	0.679401	0.248442	0.625669	0.0534657	0
ACTAC	1	0.508425	0.305578	0.663476	0	0
TRPm	1	-1.49033	0.931931	-2.0205	0	0

TRPTRNAm	1	-1.49033	0.931931	-2.0205	0	0
PAD	1	-0.161522	0.564159	-0.236143	0	0
PAC	1	-0.161522	0.564159	-0.236143	0	0
IAD	1	-0.161522	0.564159	-0.236143	0	0
IAC	1	-0.161522	0.564159	-0.236143	0	0
ASPERMD	1	-0.822578	0.794626	-1.12382	0	0
APRUT	1	-0.822578	0.794626	-1.12382	0	0
APROA	1	-0.822578	0.794626	-1.12382	0	0
GABAL	1	-0.822578	0.794626	-1.12382	0	0
ASPRM	1	-0.822578	0.794626	-1.12382	0	0
GLUP	2	-0.752811	0.774218	-0.733479	0.894723	0
PROm	1	1.79945	0.035974	2.39709	0	1
GABALm	3	0.0407517	0.483747	0.0125722	3.39644	1
GABAm	3	0.0407517	0.483747	0.0125722	3.39644	1
LACALm	3	0.0407517	0.483747	0.0125722	3.39644	1
TCOA	1	-0.506021	0.693579	-0.698744	0	0
GLP	1	-0.506021	0.693579	-0.698744	0	0
TGLP	1	-0.506021	0.693579	-0.698744	0	0
PEPD	1	-0.533754	0.703244	-0.735985	0	0

APEP	1	-0.533754	0.703244	-0.735985	0	0
GC	2	0.757377	0.224412	0.699668	1.3839	1
cAMP	3	0.0205007	0.491822	-0.00311399	0.751258	0
GMP	5	1.0903	0.13779	0.634989	1.36249	1
DGMP	1	2.24842	0.0122747	2.99998	0	1
DGDP	2	0.655516	0.256068	0.603003	0.927462	0
DATP	2	0.433286	0.332403	0.392111	0.629215	0
DADP	4	-0.0158652	0.506329	-0.0295871	1.01768	0
PRAM	1	-0.436189	0.66865	-0.604971	0	0
GAR	1	-0.436189	0.66865	-0.604971	0	0
FGAR	1	-0.906971	0.817789	-1.23715	0	0
FGAM	2	-0.950476	0.829065	-0.92106	0.447017	0
AIR	2	-0.323559	0.626864	-0.326125	0.394349	0
CAIR	2	-0.604386	0.727206	-0.592625	0.771238	0
SAICAR	3	-1.0155	0.845066	-0.805589	1.18497	0
PRFICA	1	0.834261	0.202067	1.10102	0	0
IMP	6	1.26306	0.103284	0.672519	1.27609	1
ASUC	2	-0.653673	0.743339	-0.639397	1.6256	0
XMP	3	1.07209	0.14184	0.811436	1.8954	1

cdAMP	1	0.334023	0.369181	0.429285	0	0
DAMP	4	0.812894	0.208139	0.526227	1.78882	1
cIMP	1	0.334023	0.369181	0.429285	0	0
cGMP	1	0.334023	0.369181	0.429285	0	0
cCMP	1	0.334023	0.369181	0.429285	0	0
ATN	1	-0.619247	0.732123	-0.850786	0	0
ATT	1	-0.619247	0.732123	-0.850786	0	0
UGC	1	-0.809007	0.790744	-1.1056	0	0
CAASP	3	-0.298673	0.617405	-0.250343	0.887877	0
DOROA	1	-0.293368	0.61538	-0.413189	0	0
OROA	2	0.0909294	0.463774	0.0672189	0.6794	0
OMP	2	-0.0781117	0.53113	-0.0931986	0.906264	0
UMP	2	1.21398	0.112378	1.13298	2.64034	1
URA	3	0.11266	0.45515	0.0682715	1.49421	1
CYTS	2	1.5719	0.0579864	1.47264	0.245122	1
URI	1	2.24842	0.0122747	2.99998	0	1
CYTD	1	2.24842	0.0122747	2.99998	0	1
DU	2	0.902395	0.183424	0.837288	3.0585	1
DR1P	1	-0.972694	0.834647	-1.3254	0	0

DT	2	0.902395	0.183424	0.837288	3.0585	1
THY	1	-0.972694	0.834647	-1.3254	0	0
DC	1	2.24842	0.0122747	2.99998	0	1
DTMP	3	0.519435	0.301729	0.383355	2.27047	1
DTDP	2	-0.569548	0.715508	-0.559565	0.716657	0
OTHIOm	1	0.566213	0.285624	0.741075	0	0
RTHIOm	1	0.566213	0.285624	0.741075	0	0
DUTP	3	1.36777	0.0856921	1.04047	1.20791	1
DUMP	3	1.98408	0.0236235	1.51785	2.02048	2
DCMP	1	2.24842	0.0122747	2.99998	0	1
DCDP	2	0.655516	0.256068	0.603003	0.927462	0
CDP	3	-0.908266	0.818131	-0.722526	2.38771	0
PURI5P	1	-0.0718676	0.528646	-0.115753	0	0
AD	2	-0.962538	0.83211	-0.932506	0.555639	0
INS	3	0.879137	0.189664	0.661976	2.1839	1
DA	2	0.902395	0.183424	0.837288	3.0585	1
DIN	1	-0.972694	0.834647	-1.3254	0	0
HYXN	2	-0.818857	0.793566	-0.796155	0.748468	0
DG	3	0.623786	0.266384	0.464184	2.25718	1

GN	2	-0.818857	0.793566	-0.796155	0.748468	0
GSN	4	0.663414	0.253533	0.425976	1.84456	1
XAN	2	-0.818857	0.793566	-0.796155	0.748468	0
XTSINE	2	0.902395	0.183424	0.837288	3.0585	1
ITP	2	-0.0571336	0.522781	-0.0732907	0.0289619	0
IDP	2	-0.0571336	0.522781	-0.0732907	0.0289619	0
ITPm	1	-0.902084	0.816494	-1.23059	0	0
IDPm	1	-0.902084	0.816494	-1.23059	0	0
DGTP	3	0.240635	0.404919	0.1674	0.591136	0
DUDP	2	0.655516	0.256068	0.603003	0.927462	0
DCTP	2	0.433286	0.332403	0.392111	0.629215	0
DTTP	1	-0.0249948	0.50997	-0.0528116	0	0
LCCA	3	-0.0126943	0.505064	-0.0288265	0.606657	0
ACOA	5	0.0387134	0.484559	0.00430297	0.684043	0
HACOA	3	-1.82495	0.965996	-1.43258	0.771946	0
OACOA	5	-1.36564	0.913973	-0.83795	1.11479	0
AACCOA	3	0.384207	0.350412	0.278609	0.825854	0
AACCOAm	2	0.0894061	0.46438	0.0657734	1.04512	0
MALCOA	3	-1.0313	0.8488	-0.817828	1.08192	0

MALACP	1	0.311794	0.377598	0.399436	0	0
ACACP	1	1.30233	0.096401	1.72955	0	1
C120ACP	4	-0.739448	0.770182	-0.514863	1.17735	0
C140ACP	4	-0.739448	0.770182	-0.514863	1.17735	0
C141ACP	4	-0.739448	0.770182	-0.514863	1.17735	0
C160ACP	4	-0.739448	0.770182	-0.514863	1.17735	0
C161ACP	4	-0.739448	0.770182	-0.514863	1.17735	0
C180ACP	4	-0.739448	0.770182	-0.514863	1.17735	0
C181ACP	4	-0.739448	0.770182	-0.514863	1.17735	0
C182ACP	4	-0.739448	0.770182	-0.514863	1.17735	0
3HPACP	1	1.30233	0.096401	1.72955	0	1
2HDACP	1	1.30233	0.096401	1.72955	0	1
AACP	1	1.30233	0.096401	1.72955	0	1
23DAACP	1	1.30233	0.096401	1.72955	0	1
C150ACP	3	-0.708639	0.760726	-0.567898	1.43609	0
C162ACP	4	-0.739448	0.770182	-0.514863	1.17735	0
C170ACP	4	-0.739448	0.770182	-0.514863	1.17735	0
C183ACP	4	-0.739448	0.770182	-0.514863	1.17735	0
C200ACP	4	-0.739448	0.770182	-0.514863	1.17735	0

AGL3P	1	-0.250601	0.598939	-0.35576	0	0
PA	3	-0.659919	0.745347	-0.530159	0.267505	0
PAm	1	-0.280993	0.610642	-0.396571	0	0
CTPm	2	-0.216673	0.585768	-0.224691	0.243074	0
CDPDGm	3	0.0396798	0.484174	0.011742	0.578288	0
CDPDG	1	-0.280993	0.610642	-0.396571	0	0
PS	1	0.374882	0.353874	0.484152	0	0
CMPm	2	0.247601	0.402221	0.215898	0.647114	0
PE	3	0.317822	0.37531	0.227188	0.623012	0
PMME	2	-0.95262	0.829609	-0.923094	0.622096	0
PDME	1	-1.00068	0.841509	-1.36298	0	0
PC	2	-0.339427	0.632856	-0.341183	1.44504	0
CDPCHO	1	0.52119	0.301117	0.680617	0	0
DAGLY	7	-0.600432	0.725891	-0.323137	1.16094	0
PETHM	2	-0.807048	0.79018	-0.784948	0.957585	0
CDPETN	2	0.321884	0.37377	0.286392	0.557518	0
PINS	6	-0.559833	0.712203	-0.32535	0.771122	0
PINSP	4	-0.241616	0.595461	-0.180989	0.845448	0
PINS4P	3	-0.285322	0.612301	-0.240001	0.854799	0

D45PI	3	-0.649211	0.741899	-0.521866	1.24488	0
TPI	2	-0.191041	0.575753	-0.200367	1.57457	0
GL3Pm	1	0.515873	0.302972	0.673477	0	0
PGPm	2	-0.101297	0.540342	-0.115201	1.11536	0
PGm	2	-0.583473	0.720213	-0.572779	0.468245	0
CLm	1	-0.165645	0.565782	-0.24168	0	0
CDPm	1	-0.0249948	0.50997	-0.0528116	0	0
DHSPH	1	-0.843484	0.800521	-1.1519	0	0
SPH	2	-1.30627	0.904269	-1.2587	0.151046	0
PSPH	2	-0.0786926	0.531361	-0.0937499	1.79854	0
C260COA	1	0.891597	0.186305	1.17801	0	0
CER2	1	0.891597	0.186305	1.17801	0	0
CER3	1	-0.843556	0.800541	-1.15199	0	0
IPC	2	-1.07146	0.858019	-1.03587	0.164219	0
MIPC	2	-1.07146	0.858019	-1.03587	0.164219	0
MIP2C	1	-0.843556	0.800541	-1.15199	0	0
DHSP	2	-1.46969	0.929177	-1.41378	0.068276	0
PHSP	1	-1.00256	0.841963	-1.36551	0	0
C16A	1	-1.07447	0.858693	-1.46206	0	0

H3MCOA	2	0.805668	0.210217	0.745495	0.0582857	0
MVL	2	-1.34287	0.910343	-1.29344	2.94177	0
PMVL	1	-2.49798	0.993755	-3.37359	0	0
PPMVL	1	0.179807	0.428652	0.2222	0	0
IPPP	3	-0.801295	0.78852	-0.639668	0.775365	0
DMPP	2	-1.10806	0.866082	-1.0706	0.29691	0
GPP	1	-0.626597	0.734538	-0.860655	0	0
FPP	1	-0.626597	0.734538	-0.860655	0	0
LNST	1	-0.652147	0.742847	-0.894965	0	0
IGST	1	-0.652147	0.742847	-0.894965	0	0
IIMZYMST	1	-2.49798	0.993755	-3.37359	0	0
MZYMST	1	-2.49798	0.993755	-3.37359	0	0
IIZYMST	1	-2.49798	0.993755	-3.37359	0	0
ZYMST	1	-2.49798	0.993755	-3.37359	0	0
TAGLY	3	-0.708639	0.760726	-0.567898	1.43609	0
MAGLY	3	-0.708639	0.760726	-0.567898	1.43609	0
PHACAL	3	0.0407517	0.483747	0.0125722	3.39644	1
PHAC	3	0.0407517	0.483747	0.0125722	3.39644	1
NOR	2	-1.5882	0.943879	-1.52625	0.203146	0

AVN	1	-1.01529	0.845017	-1.38261	0	0
VHA	1	0.0567719	0.477363	0.0569865	0	0
VERB	1	-0.099511	0.539634	-0.152874	0	0
VERA	1	-0.099511	0.539634	-0.152874	0	0
DMST	1	-0.099511	0.539634	-0.152874	0	0
DHDMST	1	-0.099511	0.539634	-0.152874	0	0
ST	1	-0.670607	0.748764	-0.919753	0	0
DHST	1	-0.670607	0.748764	-0.919753	0	0
OMST	2	-1.12964	0.870685	-1.09108	0.242294	0
DHOMST	2	-1.12964	0.870685	-1.09108	0.242294	0
AFB1	1	-0.925782	0.82272	-1.26241	0	0
AFG1	1	-0.925782	0.82272	-1.26241	0	0
AFB2	1	-0.925782	0.82272	-1.26241	0	0
AFG2	1	-0.925782	0.82272	-1.26241	0	0
HNO3	2	-1.42255	0.922567	-1.36905	0.30973	0
UREAC	1	0.457981	0.323483	0.595738	0	0
NH3e	1	-0.862551	0.805808	-1.1775	0	0
HNO3e	2	-1.42255	0.922567	-1.36905	0.30973	0

#Results for Down-regulated only genes

#Feature	Number of neighbors	Z-score	P-value	Average Z	StdDev Z	Significance count
DGLCe	2	0.330316	0.370581	0.294393	1.91142	1
GLCe	6	0.702439	0.241203	0.36563	1.88438	2
bDGLCe	3	0.724191	0.234474	0.541957	0.51127	0
GLC	8	0.579196	0.281229	0.255657	2.18739	2
bDGLC	4	-0.936415	0.82547	-0.646961	1.0242	0
ATP	28	-0.825283	0.795394	-0.227627	1.11728	2
ADP	18	0.139188	0.444651	0.0251631	1.17544	2
G6P	4	0.269218	0.393881	0.161606	1.82899	1
bDG6P	2	-0.817636	0.793218	-0.794997	1.62803	0
H2O	32	0.263341	0.396144	0.0435843	1.90986	8
PI	23	-1.00665	0.84295	-0.299918	1.43673	3
F6P	5	1.23403	0.108595	0.721191	1.06244	1
FDP	1	1.58204	0.0568198	2.10515	0	1
S7P	1	1.58204	0.0568198	2.10515	0	1
S17P	1	1.58204	0.0568198	2.10515	0	1
T3P2	4	-1.20811	0.886497	-0.829173	0.447892	0
T3P1	2	-0.241161	0.595285	-0.24793	1.00617	0

NAD	27	-1.08442	0.86091	-0.298257	1.31977	3
13PDG	2	0.769278	0.220864	0.710962	0.349905	0
NADH	27	-1.08442	0.86091	-0.298257	1.31977	3
3PG	2	-0.366641	0.643056	-0.367008	1.87439	0
2PG	1	-1.38496	0.916968	-1.879	0	0
PEP	1	-1.38496	0.916968	-1.879	0	0
PYR	6	0.570017	0.284333	0.293141	2.0034	2
CO2	19	1.0127	0.155603	0.292449	1.58324	5
OA	5	0.781681	0.217201	0.449895	1.11862	1
ATPm	5	0.199856	0.420797	0.100947	1.52669	1
PYRm	5	0.441169	0.329545	0.245674	1.33729	1
CO2m	6	0.271184	0.393125	0.129557	1.10654	1
ADPm	3	-1.15681	0.876325	-0.915048	0.59224	0
PIm	5	-0.41295	0.660178	-0.26658	1.37267	1
OAm	6	-0.114581	0.545611	-0.0816143	1.45921	1
GTP	3	-1.03134	0.848808	-0.817855	0.75352	0
GDP	2	-1.13226	0.871237	-1.09357	0.824306	0
NADP	33	2.60139	0.00464228	0.587621	1.65947	11
D6PGL	1	-0.665346	0.747086	-0.912689	0	0

NADPH	33	2.60139	0.00464228	0.587621	1.65947	11
D6PGC	4	-0.286884	0.6129	-0.211348	1.35915	1
RL5P	4	-1.36316	0.913583	-0.933159	0.383543	0
XUL5P	1	-0.941096	0.826672	-1.28297	0	0
R5P	5	-2.06635	0.980602	-1.2582	0.462037	0
ACCOAm	2	-0.259111	0.602225	-0.264964	0.171068	0
H2Om	6	-0.206902	0.581957	-0.132152	0.837424	0
CITm	1	-0.273067	0.607599	-0.385928	0	0
COAm	3	-0.886927	0.812441	-0.705997	0.77341	0
NADm	8	0.302462	0.38115	0.124496	1.67994	2
AKGm	3	1.03299	0.150804	0.781152	0.556989	0
NADHm	8	0.302462	0.38115	0.124496	1.67994	2
AKG	7	-0.723434	0.765293	-0.385467	1.16778	0
NADPm	2	0.209438	0.417053	0.179682	0.144237	0
NADPHm	2	0.209438	0.417053	0.179682	0.144237	0
LIPOm	1	0.188577	0.425212	0.233977	0	0
SUCDLIP	1	-1.1683	0.878657	-1.58806	0	0
SUCCOAm	1	-1.1683	0.878657	-1.58806	0	0
DHLIPOm	2	-0.89249	0.813935	-0.866032	1.02111	0

Qm	2	-0.609886	0.729031	-0.597845	0.623616	0
QH2m	2	-0.609886	0.729031	-0.597845	0.623616	0
FADH2m	3	0.941574	0.173205	0.710339	2.52934	1
FADm	3	0.941574	0.173205	0.710339	2.52934	1
SUCC	2	-1.16334	0.877653	-1.12306	0.279094	0
MALm	3	-0.521664	0.699048	-0.423069	2.1139	1
MAL	2	0.477345	0.316558	0.433922	2.12852	1
Hm	4	0.485715	0.313584	0.306802	1.24043	1
CIT	1	0.175327	0.430411	0.216185	0	0
COA	14	-0.615129	0.730765	-0.239096	0.918092	1
ACCOA	7	-0.460071	0.677267	-0.25201	0.730131	0
OXAL	1	-0.733007	0.768223	-1.00354	0	0
AC	5	0.441481	0.329432	0.245861	0.908282	1
FOR	4	-0.813659	0.79208	-0.564634	1.04944	0
FORm	1	-0.73454	0.76869	-1.0056	0	0
METHOL	1	2.28415	0.0111813	3.04796	0	1
FALD	2	0.967736	0.166588	0.899296	3.03867	1
ADHLIPOm	2	0.0675035	0.47309	0.0449881	0.26727	0
ACAL	5	2.28073	0.0112822	1.34894	1.50584	2

RGT	3	-0.238633	0.594305	-0.203836	0.519153	0
H+	12	1.16863	0.121276	0.433278	1.77935	3
MTHGXL	1	-0.317043	0.624395	-0.44498	0	0
LACAL	1	0.224096	0.411341	0.281673	0	0
LAC	1	0.224096	0.411341	0.281673	0	0
LGT	1	-0.317043	0.624395	-0.44498	0	0
LLAC	1	-0.831868	0.797258	-1.1363	0	0
AMP	12	-1.61839	0.947211	-0.644912	0.809474	0
PPI	13	-1.02211	0.846636	-0.398691	0.764788	0
GLU	13	0.470609	0.31896	0.156093	1.46538	2
GABA	3	1.8396	0.0329135	1.40594	2.44974	2
SUCCSAL	3	-1.53705	0.937859	-1.20958	0.247793	0
METTHF	1	-0.0873497	0.534803	-0.136543	0	0
METTHFm	1	-0.0873497	0.534803	-0.136543	0	0
THFm	1	-0.0873497	0.534803	-0.136543	0	0
DHP	1	-0.238014	0.594065	-0.338859	0	0
AHHMP	1	-0.238014	0.594065	-0.338859	0	0
GLAL	1	-0.238014	0.594065	-0.338859	0	0
CHOR	1	-0.148274	0.558937	-0.218353	0	0

GLN	4	-0.960458	0.831588	-0.663086	0.728955	0
PABA	1	-0.238014	0.594065	-0.338859	0	0
AHHMD	1	-0.238014	0.594065	-0.338859	0	0
DHPT	1	-0.238014	0.594065	-0.338859	0	0
THF	2	0.0451288	0.482002	0.0237549	0.226696	0
THFG	1	0.151398	0.439831	0.184053	0	0
PNTO	1	-1.33501	0.909063	-1.81192	0	0
4PPNTO	1	-1.33501	0.909063	-1.81192	0	0
CTP	2	-0.910567	0.818738	-0.883187	0.28404	0
CYS	4	-0.97009	0.833999	-0.669546	0.455549	0
CMP	4	-0.960178	0.831517	-0.662898	1.20187	0
ASP	7	-0.454816	0.675379	-0.249347	0.942616	0
PAP	1	-0.542779	0.706359	-0.748103	0	0
ACP	6	0.537857	0.295338	0.275537	0.49615	0
ALA	4	-0.289831	0.614027	-0.213324	1.49386	0
CHCOA	1	-0.522326	0.699278	-0.720638	0	0
AONA	1	-0.522326	0.699278	-0.720638	0	0
ETH	1	2.28415	0.0111813	3.04796	0	1
ETHm	1	2.28415	0.0111813	3.04796	0	1

ACALm	3	1.49081	0.0680051	1.13577	1.65914	1
ACm	2	0.209438	0.417053	0.179682	0.144237	0
AMPm	2	1.73239	0.0416022	1.62494	0.93794	1
PPIm	3	1.47357	0.0702987	1.12242	1.09428	1
GLYN	1	-0.461089	0.677633	-0.638408	0	0
GL	3	1.36328	0.0863965	1.03699	1.86595	1
GLYAL	2	1.28964	0.0985881	1.20478	2.60666	1
O2	24	2.8906	0.00192255	0.771515	2.11138	10
H2O2	5	1.35107	0.0883359	0.791386	2.68112	2
GL3P	4	-0.589004	0.722071	-0.413967	0.863713	0
TAR	1	-1.74366	0.959391	-2.36067	0	0
OXGLY	1	-1.74366	0.959391	-2.36067	0	0
E	1	-0.461089	0.677633	-0.638408	0	0
EOL	1	-0.461089	0.677633	-0.638408	0	0
LXUL	1	0.32846	0.371282	0.421815	0	0
XOL	1	0.32846	0.371282	0.421815	0	0
XUL	1	0.32846	0.371282	0.421815	0	0
AOL	1	0.32846	0.371282	0.421815	0	0
ARAB	1	-0.461089	0.677633	-0.638408	0	0

ARABLAC	1	-0.461089	0.677633	-0.638408	0	0
RIB	1	-0.609835	0.729014	-0.838147	0	0
RL	1	-0.570238	0.715742	-0.784975	0	0
O2e	6	1.62917	0.0516386	0.872932	2.39002	2
GLCN15LACe	2	0.901403	0.183687	0.836347	0.0529229	0
H2O2e	6	1.62917	0.0516386	0.872932	2.39002	2
H2Oe	8	1.46928	0.0708788	0.677521	2.46412	4
GLCNT	2	0.690059	0.245078	0.635785	1.62137	1
GLAC	6	2.38311	0.00858358	1.28565	2.43844	3
UTP	1	-0.493805	0.689278	-0.68234	0	0
UDPGAL	2	0.211718	0.416163	0.181846	1.60213	0
UDPG	4	0.824348	0.204871	0.533908	1.64954	1
G1P	1	0.91655	0.179689	1.21152	0	0
MELI	2	1.7939	0.0364149	1.68331	3.59308	1
GALN14LAC	2	0.834408	0.202026	0.772769	3.42916	1
SOR	3	-0.104352	0.541555	-0.0998232	0.550057	0
SOT	2	-0.113017	0.544991	-0.126323	0.775185	0
MAN6P	2	0.611383	0.270473	0.561122	0.289809	0
MAN	2	0.183099	0.42736	0.154686	0.284978	0

FRU	3	0.339177	0.367238	0.243729	0.253756	0
MNT6P	1	-0.570301	0.715763	-0.785061	0	0
MNT	1	0.32846	0.371282	0.421815	0	0
F26P	1	0.880883	0.189191	1.16362	0	0
MAN1P	1	-0.184077	0.573023	-0.26643	0	0
GDPMAN	1	-0.184077	0.573023	-0.26643	0	0
UDP	4	0.772384	0.219944	0.499058	1.62761	1
TRE6P	1	1.86335	0.0312067	2.48289	0	1
MLT	1	-1.16383	0.877753	-1.58206	0	0
MLTe	1	-1.16383	0.877753	-1.58206	0	0
LACT	1	2.13565	0.0163541	2.84854	0	1
LACTe	1	2.13565	0.0163541	2.84854	0	1
GLACe	2	1.49626	0.0672926	1.40086	2.04733	1
GA6P	1	0.279593	0.389895	0.356196	0	0
UDPNAG	1	0.887415	0.187428	1.17239	0	0
CHIT	5	1.92282	0.0272514	1.13429	1.56135	2
NAG	4	1.70536	0.0440641	1.12476	1.80273	2
GLCN	1	0.279593	0.389895	0.356196	0	0
13GLUCANe	7	0.268918	0.393996	0.117398	1.57395	2

GLYCOGEN	2	0.283833	0.388269	0.250282	1.35939	0
AMYLSe	1	0.449948	0.326374	0.584952	0	0
AMYLPe	1	0.449948	0.326374	0.584952	0	0
CELLUe	5	-0.371805	0.644981	-0.241904	1.40072	1
CELLOBe	5	-0.371805	0.644981	-0.241904	1.40072	1
CELLOTe	2	0.330316	0.370581	0.294393	1.91142	1
MANNANe	2	1.28832	0.0988167	1.20353	1.1739	1
MANe	3	1.04021	0.14912	0.786744	1.10007	1
ARABINe	1	-0.435082	0.668248	-0.603485	0	0
LARABe	1	-0.435082	0.668248	-0.603485	0	0
XYLANe	4	0.234742	0.407204	0.138485	1.10394	1
XYLe	4	0.234742	0.407204	0.138485	1.10394	1
H+_PO_mit	3	-0.305266	0.619918	-0.25545	1.26117	0
H+_PO	3	-0.305266	0.619918	-0.25545	1.26117	0
FERIm	3	-0.198619	0.57872	-0.172842	1.07843	0
FEROm	3	-0.198619	0.57872	-0.172842	1.07843	0
O2m	1	0.781892	0.217139	1.03069	0	0
Ca	1	-0.214269	0.584831	-0.306973	0	0
Cam	1	-0.214269	0.584831	-0.306973	0	0

LLACm	2	0.209438	0.417053	0.179682	0.144237	0
LACm	1	-0.768685	0.77896	-1.05145	0	0
GLUm	3	1.03299	0.150804	0.781152	0.556989	0
ASPm	3	1.51073	0.0654282	1.1512	1.07779	1
ALAm	1	0.891597	0.186305	1.17801	0	0
ASN	2	0.167879	0.433339	0.140243	0.0582475	0
SAM	4	-1.15127	0.875189	-0.791055	0.49969	0
HCYS	3	0.398888	0.344988	0.289981	1.2776	1
SAH	3	-1.22967	0.890589	-0.97148	0.423338	0
MET	1	1.28309	0.0997296	1.70372	0	1
TRNAm	1	1.71833	0.0428681	2.28816	0	1
ASPTRNAm	1	1.71833	0.0428681	2.28816	0	1
TRNA	1	-0.108447	0.543179	-0.164873	0	0
ASPTRNA	1	-0.108447	0.543179	-0.164873	0	0
NH3	13	0.150867	0.44004	0.0372582	1.36644	2
CAP	1	2.78389	0.00268557	3.71902	0	1
ORN	1	2.78389	0.00268557	3.71902	0	1
CITR	1	2.78389	0.00268557	3.71902	0	1
SPRMD	2	-0.649024	0.741838	-0.634986	0.571091	0

SPRM	1	-0.157813	0.562698	-0.231163	0	0
GBAD	3	1.36452	0.0862024	1.03795	2.25993	1
GBAT	3	1.36452	0.0862024	1.03795	2.25993	1
UREA	1	-2.16463	0.984792	-2.92596	0	0
DAPRP	1	-0.759267	0.776154	-1.03881	0	0
SLF	1	-1.52025	0.935776	-2.06067	0	0
APS	1	-1.52025	0.935776	-2.06067	0	0
SER	5	-0.518115	0.697811	-0.329653	0.475932	0
ASER	3	-0.294328	0.615747	-0.246977	0.468905	0
H2S	3	0.398888	0.344988	0.289981	1.2776	1
PHP	1	-1.246	0.893617	-1.6924	0	0
GLYm	1	-0.0873497	0.534803	-0.136543	0	0
GLY	3	-0.761482	0.776815	-0.608829	1.30956	0
GLX	1	-1.54136	0.938385	-2.08902	0	0
BASP	1	-0.991049	0.839169	-1.35005	0	0
THR	1	0.311526	0.377701	0.399075	0	0
LLCT	3	-0.634575	0.737147	-0.510528	0.399461	0
OBUT	1	-0.505326	0.693335	-0.69781	0	0
THRm	2	-1.69157	0.954636	-1.62434	0.0474824	0

NH3m	2	-1.69157	0.954636	-1.62434	0.0474824	0
OBUTm	2	-1.69157	0.954636	-1.62434	0.0474824	0
PRPP	4	-0.987902	0.8384	-0.681491	0.848776	0
HISOLP	1	-0.947495	0.828307	-1.29156	0	0
HISOL	1	-0.947495	0.828307	-1.29156	0	0
HIS	1	-0.919435	0.821066	-1.25388	0	0
MHIS	1	-0.919435	0.821066	-1.25388	0	0
VAL	2	-1.86296	0.968766	-1.78699	0.905655	0
AKA	1	-0.52762	0.701119	-0.727748	0	0
AMA	3	-1.29343	0.902069	-1.02087	0.254707	0
LYS	1	0.730525	0.232535	0.961717	0	0
LTRNA	1	0.730525	0.232535	0.961717	0	0
LLTRNA	1	0.730525	0.232535	0.961717	0	0
LYSm	1	0.730525	0.232535	0.961717	0	0
LTRNAm	1	0.730525	0.232535	0.961717	0	0
LLTRNAm	1	0.730525	0.232535	0.961717	0	0
ADN	3	-1.8472	0.967641	-1.44982	0.605523	0
OAHSER	1	1.28309	0.0997296	1.70372	0	1
METH	1	1.28309	0.0997296	1.70372	0	1

CALH	1	-0.346647	0.635572	-0.484733	0	0
DPTH	1	-0.346647	0.635572	-0.484733	0	0
DQT	1	1.24009	0.107471	1.64597	0	1
QT	1	1.24009	0.107471	1.64597	0	1
3PSME	1	-0.148274	0.558937	-0.218353	0	0
PHEN	1	-0.886108	0.81222	-1.20913	0	0
PHPYR	1	2.2025	0.013815	2.93832	0	1
4HPP	4	-0.151035	0.560026	-0.12024	0.941408	0
TYR	2	0.634147	0.262993	0.582724	0.619865	0
TRP	2	-1.62736	0.948169	-1.56341	0.232958	0
FKYN	3	-0.890732	0.813463	-0.708945	1.48912	0
KYN	2	0.658707	0.255042	0.606031	0.557134	0
HKYN	1	0.172269	0.431613	0.212078	0	0
HOMOGEN	1	-0.311305	0.622216	-0.437275	0	0
PAD	2	1.04302	0.148469	0.970742	3.19179	1
PAC	2	1.04302	0.148469	0.970742	3.19179	1
IAD	2	1.04302	0.148469	0.970742	3.19179	1
IAC	2	1.04302	0.148469	0.970742	3.19179	1
ASPERMD	1	-0.157813	0.562698	-0.231163	0	0

ASPRM	1	-0.157813	0.562698	-0.231163	0	0
P5C	1	0.515873	0.302972	0.673477	0	0
PRO	1	0.515873	0.302972	0.673477	0	0
PHC	1	0.515873	0.302972	0.673477	0	0
HPRO	1	0.515873	0.302972	0.673477	0	0
GABALm	2	0.209438	0.417053	0.179682	0.144237	0
GABAm	2	0.209438	0.417053	0.179682	0.144237	0
LACALm	2	0.209438	0.417053	0.179682	0.144237	0
APROP	1	0.593975	0.276264	0.778355	0	0
OGT	2	-0.0676427	0.526965	-0.0832636	0.672171	0
GMP	1	-1.51156	0.934677	-2.049	0	0
DGMP	1	-1.51156	0.934677	-2.049	0	0
IMP	3	-1.35909	0.912941	-1.07173	1.38264	0
ASUC	1	-1.23411	0.891419	-1.67644	0	0
XMP	2	-0.790636	0.785422	-0.769374	1.80967	0
DAMP	1	-1.51156	0.934677	-2.049	0	0
ATT	1	-2.16463	0.984792	-2.92596	0	0
UGC	1	-2.16463	0.984792	-2.92596	0	0
UMP	4	-1.18566	0.882121	-0.814116	0.960927	0

URA	3	-1.17094	0.879188	-0.925991	1.03035	0
URI	3	-1.23762	0.892072	-0.977641	0.930367	0
CYTD	3	-1.23762	0.892072	-0.977641	0.930367	0
DU	2	-1.25612	0.895464	-1.21111	1.18495	0
DT	1	-1.51156	0.934677	-2.049	0	0
DC	2	-1.25612	0.895464	-1.21111	1.18495	0
DTMP	1	-1.51156	0.934677	-2.049	0	0
DUMP	2	-1.55901	0.940503	-1.49855	0.778454	0
DCMP	2	-1.55901	0.940503	-1.49855	0.778454	0
DCDP	1	-0.691718	0.755443	-0.948101	0	0
CDP	1	-0.691718	0.755443	-0.948101	0	0
PURI5P	2	-1.57598	0.942485	-1.51465	0.210024	0
AD	3	-1.04119	0.851106	-0.825487	0.64324	0
INS	2	-1.82993	0.96637	-1.75565	0.414858	0
DA	2	-1.82993	0.96637	-1.75565	0.414858	0
DIN	1	-1.07464	0.858733	-1.4623	0	0
HYXN	1	-1.07464	0.858733	-1.4623	0	0
DG	1	-1.51156	0.934677	-2.049	0	0
GN	1	-0.609835	0.729014	-0.838147	0	0

GSN	2	-1.50108	0.933332	-1.44357	0.856203	0
XTSINE	1	-1.51156	0.934677	-2.049	0	0
DUDP	1	-0.691718	0.755443	-0.948101	0	0
LCCA	1	-0.48022	0.684464	-0.664097	0	0
ACOA	2	-1.30821	0.904599	-1.26055	0.843505	0
OACOA	1	-1.36857	0.914433	-1.85699	0	0
ACACPm	1	0.338197	0.367607	0.43489	0	0
MALACPm	1	0.338197	0.367607	0.43489	0	0
ACPm	1	0.338197	0.367607	0.43489	0	0
MALCOA	2	0.576863	0.282016	0.528362	0.600625	0
MALACP	2	0.598337	0.274808	0.548741	0.571806	0
ACACP	3	0.04854	0.480643	0.018605	0.217907	0
3OACPm	1	0.338197	0.367607	0.43489	0	0
C100ACP	1	0.121879	0.451497	0.144413	0	0
C120ACP	5	0.157008	0.437619	0.0752495	0.551409	0
C140ACP	5	0.157008	0.437619	0.0752495	0.551409	0
C141ACP	5	0.157008	0.437619	0.0752495	0.551409	0
C160ACP	5	0.157008	0.437619	0.0752495	0.551409	0
C161ACP	5	0.157008	0.437619	0.0752495	0.551409	0

C180ACP	5	0.157008	0.437619	0.0752495	0.551409	0
C181ACP	5	0.157008	0.437619	0.0752495	0.551409	0
C182ACP	5	0.157008	0.437619	0.0752495	0.551409	0
3HPACP	2	-0.0265837	0.510604	-0.0442993	0.26688	0
2HDACP	2	-0.0265837	0.510604	-0.0442993	0.26688	0
AACP	2	-0.0265837	0.510604	-0.0442993	0.26688	0
23DAACP	2	-0.0265837	0.510604	-0.0442993	0.26688	0
C150ACP	3	0.551755	0.290558	0.40839	0.352893	0
C162ACP	5	0.157008	0.437619	0.0752495	0.551409	0
C170ACP	5	0.157008	0.437619	0.0752495	0.551409	0
C183ACP	5	0.157008	0.437619	0.0752495	0.551409	0
C200ACP	5	0.157008	0.437619	0.0752495	0.551409	0
AGL3P	2	0.965585	0.167126	0.897255	1.50208	1
AT3P2	2	0.965585	0.167126	0.897255	1.50208	1
PA	2	-0.79395	0.786388	-0.772518	0.421939	0
CDPDG	1	0.651951	0.257216	0.856206	0	0
PS	2	1.54744	0.0608786	1.44943	2.51483	1
CMPm	1	-0.691718	0.755443	-0.948101	0	0
PSm	1	0.197706	0.421638	0.246235	0	0

PE	3	0.97763	0.164129	0.738268	2.1632	1
PEm	1	0.197706	0.421638	0.246235	0	0
PC	1	-0.495077	0.689727	-0.684048	0	0
CHO	1	0.259072	0.39779	0.32864	0	0
PCHO	2	-0.377904	0.647249	-0.377697	0.998911	0
CDPCHO	1	-0.792947	0.786096	-1.08403	0	0
DAGLY	4	-0.144818	0.557573	-0.11607	0.825888	0
MI1P	2	-1.33666	0.909333	-1.28754	1.47229	0
MYOI	2	-0.755678	0.775079	-0.736199	2.252	0
PINS	1	0.651951	0.257216	0.856206	0	0
DGPP	2	-0.79395	0.786388	-0.772518	0.421939	0
LPC	1	-0.495077	0.689727	-0.684048	0	0
LPE	1	-0.495077	0.689727	-0.684048	0	0
CDPm	1	-0.691718	0.755443	-0.948101	0	0
PALCOA	2	-0.457958	0.676509	-0.453667	0.644233	0
DHSPH	2	-0.457958	0.676509	-0.453667	0.644233	0
SPH	2	0.22356	0.41155	0.193083	3.02539	1
PSPH	1	1.75124	0.0399522	2.33236	0	1
CER2	1	2.38823	0.00846485	3.18772	0	1

CER3	1	2.38823	0.00846485	3.18772	0	1
DHSP	1	-1.43499	0.924356	-1.94619	0	0
H3MCOA	1	1.38527	0.0829857	1.84092	0	1
MVL	1	1.38527	0.0829857	1.84092	0	1
PMVL	1	1.85365	0.0318944	2.46988	0	1
PPMVL	1	1.85365	0.0318944	2.46988	0	1
S23E	1	-1.41504	0.921472	-1.9194	0	0
LNST	3	0.596719	0.275347	0.443219	2.84198	1
IGST	4	2.71803	0.00328355	1.80392	1.75354	2
DMZYMST	5	2.85775	0.0021333	1.69501	1.60422	3
IMZYMST	4	1.56993	0.0582155	1.03394	1.94419	2
IIMZYMST	1	-0.263168	0.603789	-0.372635	0	0
MZYMST	3	1.96464	0.0247277	1.5028	2.08587	2
IZYMST	4	1.56993	0.0582155	1.03394	1.94419	2
IIZYMST	1	-0.263168	0.603789	-0.372635	0	0
ZYMST	1	-0.861302	0.805464	-1.17582	0	0
FEST	2	-0.112564	0.544812	-0.125893	1.48482	0
EPST	3	2.83506	0.00229083	2.17702	1.34424	2
ERTROL	3	2.343	0.00956477	1.79587	1.91717	2

ERTEOL	2	0.522201	0.300765	0.476489	0.984155	0
ERGOST	1	0.887415	0.187428	1.17239	0	0
TAGLY	2	0.589524	0.277755	0.540378	0.380187	0
MAGLY	1	0.216554	0.414278	0.271545	0	0
PHACAL	3	1.44363	0.0744215	1.09923	1.59596	1
PHAC	3	-0.162199	0.564425	-0.144631	0.570911	0
PHACCOA	2	-1.02393	0.847066	-0.990765	0.279318	0
LLDACV	2	0.101495	0.459579	0.0772458	1.73078	0
IPN	2	0.0795373	0.468303	0.056408	1.76024	0
PENG	1	-0.870574	0.808007	-1.18827	0	0
NOR	1	2.26492	0.0117588	3.02213	0	1
AVN	4	-0.0305287	0.512177	-0.0394213	2.08108	1
HAVN	4	-1.25019	0.894386	-0.8574	0.573718	0
AVF	2	-1.0043	0.842384	-0.97214	1.02157	0
VHA	1	-1.24756	0.893904	-1.6945	0	0
VERB	2	-1.97863	0.976071	-1.89677	0.750413	0
VERA	1	-1.00303	0.842078	-1.36614	0	0
DMST	1	-1.00303	0.842078	-1.36614	0	0
DHDMST	1	-1.00303	0.842078	-1.36614	0	0

OMST	1	2.40601	0.00806386	3.2116	0	1
DHOMST	1	2.40601	0.00806386	3.2116	0	1
AFB1	1	2.40601	0.00806386	3.2116	0	1
AFG1	1	2.40601	0.00806386	3.2116	0	1
AFB2	1	2.40601	0.00806386	3.2116	0	1
AFG2	1	2.40601	0.00806386	3.2116	0	1
HNO3	1	0.579371	0.281169	0.758744	0	0
HNO2	2	2.1489	0.0158213	2.0202	1.78397	1
NH4OH	1	2.45818	0.00698206	3.28165	0	1
ACNL	1	0.593975	0.276264	0.778355	0	0
INAC	1	0.593975	0.276264	0.778355	0	0
NH3e	1	1.30821	0.0954004	1.73745	0	1
FRUe	1	-0.0205353	0.508192	-0.0468234	0	0
SORe	1	-0.0205353	0.508192	-0.0468234	0	0
