# Supplementary material for: Systems Analysis Unfolds the Relationship between the Phosphoketolase Pathway and Growth in Aspergillus nidulans
Source: PLoS One. 2008 Dec 4;3(12):e3847. doi: 10.1371/journal.pone.0003847 (PMC2585806; doi:10.1371/journal.pone.0003847)
Supplement: Table S5 — Results of reporter feature algorithm for the examination of phosphoketolase over-expression on xylose. (0.36 MB PDF) [file pone.0003847.s005.pdf]

#Total number of ORFs used = 576

#Total number of significantly changed ORFs = 0

#Mean_all = -1.11157 #Std_all= 1.07137

#kmax, imax = 100, 10000

#Feature	Number of neighbors	Z-score	P-value	Average Z	StdDev Z	Significance count
DGLCe	4	-0.174734	0.569356	-1.2038	0.612975	0
GLCe	19	0.842737	0.199688	-0.904439	1.14488	1
bDGLCe	3	1.03285	0.150838	-0.475248	0.459306	0
DGLC	2	-1.0565	0.85463	-1.90554	0.344887	0
GLC	20	4.4421	1.44373e-005	-0.049024	1.62755	3
bDGLC	7	0.888954	0.187014	-0.752344	0.679675	0
ATP	89	-0.884297	0.811732	-1.21191	0.82326	1
ADP	59	-0.885603	0.812084	-1.23489	0.875349	1
G6P	10	-1.01458	0.844847	-1.45362	0.412842	0
bDG6P	5	-0.260225	0.602655	-1.23487	0.266691	0
H2O	73	2.69338	0.00353661	-0.773413	1.37729	4
PI	58	0.689638	0.245211	-1.0143	1.22088	2
F6P	14	0.0516787	0.479392	-1.09633	0.57001	0
FDP	3	-0.105027	0.541823	-1.17513	0.490885	0

S7P	6	0.168606	0.433053	-1.0374	0.360957	0
S17P	3	-0.105027	0.541823	-1.17513	0.490885	0
T3P2	7	-1.00968	0.843677	-1.51813	0.276517	0
T3P1	11	1.30744	0.0955316	-0.689986	1.62161	1
E4P	7	-0.484555	0.686004	-1.30633	0.424077	0
NAD	72	-0.45998	0.677235	-1.16947	1.069	2
13PDG	6	-0.616028	0.731062	-1.37913	0.704407	0
NADH	72	-0.45998	0.677235	-1.16947	1.069	2
3PG	5	-1.13709	0.87225	-1.65306	0.518607	0
2PG	3	-0.860025	0.805112	-1.63951	0.259709	0
23PDG	2	-0.84701	0.801505	-1.74785	0.253917	0
PEP	6	-0.0161111	0.506427	-1.11785	0.452328	0
PYR	17	-1.73506	0.958635	-1.56098	0.620371	0
CO2	41	-0.64652	0.741029	-1.21941	0.850245	0
OA	12	0.247767	0.402157	-1.03465	0.745636	0
ATPm	22	-1.95031	0.97443	-1.55591	0.71541	0
PYRm	9	-1.31733	0.906136	-1.57979	0.834215	0
CO2m	14	-0.771742	0.779866	-1.33149	0.655428	0
ADPm	14	-1.73786	0.958883	-1.60741	0.71416	0

PIm	15	-1.79609	0.96376	-1.60673	0.655776	0
OAm	10	-0.327616	0.628399	-1.22164	0.822763	0
GTP	10	1.19523	0.115998	-0.707383	1.03082	0
GDP	7	1.71458	0.0432111	-0.41934	0.830792	0
NADP	64	-0.186643	0.57403	-1.13635	0.802185	0
D6PGL	2	-0.254777	0.600552	-1.30207	0.204625	0
NADPH	64	-0.186643	0.57403	-1.13635	0.802185	0
D6PGC	7	0.886507	0.187672	-0.753331	0.895599	0
RL5P	5	-1.03471	0.849599	-1.60424	0.73985	0
XUL5P	6	1.91735	0.0275968	-0.275793	1.98827	1
R5P	15	0.130129	0.448232	-1.0752	0.49675	0
ACTP	2	2.45021	0.00713866	0.734012	4.22143	1
ACCOAm	11	0.0478202	0.48093	-1.09563	0.865873	0
H2Om	19	-0.643779	0.740141	-1.26908	0.90172	0
CITm	5	-1.09141	0.862454	-1.63128	0.664539	0
COAm	13	-0.223649	0.588485	-1.17735	0.820092	0
ACOm	3	-1.2223	0.889202	-1.86234	0.820737	0
ICITm	5	-1.64753	0.950275	-1.8965	0.582435	0
NADm	22	1.86021	0.0314277	-0.687015	1.27657	2

AKGm	9	-0.516972	0.697412	-1.29495	0.643942	0
NADHm	22	1.86021	0.0314277	-0.687015	1.27657	2
ICIT	2	-1.11257	0.867053	-1.94774	0.0308982	0
AKG	19	0.476268	0.316942	-0.994333	1.30794	1
NADPm	17	-0.491828	0.688579	-1.23865	0.819678	0
NADPHm	17	-0.491828	0.688579	-1.23865	0.819678	0
ICITg	2	1.5536	0.0601397	0.0591233	2.80723	1
NADPg	1	-0.767721	0.778674	-1.92589	0	0
AKGg	1	-0.767721	0.778674	-1.92589	0	0
CO2g	1	-0.767721	0.778674	-1.92589	0	0
NADPHg	1	-0.767721	0.778674	-1.92589	0	0
LIPOm	2	-0.441243	0.670481	-1.44242	0.669611	0
SUCDLIP	2	-0.103809	0.54134	-1.18843	0.310414	0
SUCCOAm	2	-0.686173	0.753698	-1.62679	0.309512	0
DHLIPOm	2	-0.329492	0.629108	-1.35831	0.0701747	0
GDPm	2	-0.293862	0.615568	-1.33149	0.727127	0
GTPm	2	-0.293862	0.615568	-1.33149	0.727127	0
SUCCm	3	-0.322739	0.626554	-1.30904	0.644623	0
Qm	9	-1.35502	0.912294	-1.5932	0.519012	0

FUMm	3	0.0502576	0.479959	-1.07962	0.45179	0
QH2m	9	-1.35502	0.912294	-1.5932	0.519012	0
FADH2m	7	-0.125815	0.550061	-1.16164	0.733754	0
FADm	7	-0.125815	0.550061	-1.16164	0.733754	0
FUM	5	-0.563488	0.713449	-1.3795	0.725209	0
SUCC	6	-1.07343	0.85846	-1.57833	0.665153	0
MALm	4	-0.170189	0.567569	-1.20138	0.859715	0
MAL	4	0.259745	0.39753	-0.972232	0.708497	0
MALg	2	0.866283	0.193167	-0.458231	1.85124	0
NADg	1	-0.618492	0.731874	-1.76725	0	0
OAg	1	-0.618492	0.731874	-1.76725	0	0
NADHg	1	-0.618492	0.731874	-1.76725	0	0
SUCCg	1	2.96685	0.00150436	2.04414	0	1
GLXg	2	3.39805	0.000339343	1.44746	0.843824	1
ACCOAg	2	1.89101	0.0293112	0.313099	0.760411	0
H2Og	1	1.84428	0.0325715	0.85079	0	0
COAg	2	1.89101	0.0293112	0.313099	0.760411	0
Hm	11	-0.0871535	0.534725	-1.1391	0.934893	0
CIT	1	-0.0519756	0.520726	-1.16502	0	0

COA	37	0.149039	0.440761	-1.08504	1.02544	1
ACCOA	24	0.496729	0.30969	-1.00274	1.06027	1
CAR	2	1.45265	0.0731606	-0.0168654	1.40085	0
ACAR	2	1.45265	0.0731606	-0.0168654	1.40085	0
ACARm	2	0.656676	0.255694	-0.616005	0.553541	0
CARm	2	0.656676	0.255694	-0.616005	0.553541	0
ACARg	2	0.656676	0.255694	-0.616005	0.553541	0
CARg	2	0.656676	0.255694	-0.616005	0.553541	0
OXAL	3	-0.0409292	0.516324	-1.1357	1.11018	0
AC	17	0.107125	0.457345	-1.08336	1.03646	0
FOR	7	-0.21631	0.585627	-1.19814	1.0584	0
FORm	2	-0.878406	0.810138	-1.77148	0.884236	0
METHOL	4	1.84497	0.0325209	-0.127346	2.06431	1
FALD	8	1.19767	0.115524	-0.658955	1.50043	1
ADHLIPOm	2	-0.666926	0.74759	-1.6123	0.429372	0
ACAL	13	0.83772	0.201094	-0.862833	1.35831	1
RGT	7	-2.03907	0.979278	-1.93332	0.646486	0
FGT	2	-1.52638	0.936542	-2.25922	0.484352	0
H+	25	1.18898	0.117224	-0.856819	0.872552	0

HCIT	1	1.02478	0.152734	-0.0203778	0	0
MTHGXL	3	0.715935	0.237016	-0.670174	1.33344	0
LACAL	5	0.837584	0.201132	-0.711308	1.18823	0
LAC	4	0.0135787	0.494583	-1.10343	0.943103	0
LGT	2	-0.429859	0.666351	-1.43386	0.391	0
LLAC	3	-0.90068	0.816121	-1.66452	0.603147	0
PROP	2	-1.07766	0.859406	-1.92146	0.466026	0
AMP	38	0.0536933	0.47859	-1.10195	0.833427	0
PPI	45	-0.965164	0.832769	-1.2654	0.690946	0
PROPCOA	3	-1.02588	0.847526	-1.74153	0.453562	0
2MCIT	1	-0.255769	0.600935	-1.38166	0	0
GLU	34	-0.721048	0.76456	-1.24363	1.15284	1
GABA	3	-0.304664	0.619689	-1.29792	0.839144	0
SUCCSAL	5	-0.127155	0.550591	-1.17141	0.482703	0
METTHF	6	0.641912	0.260465	-0.831271	0.679705	0
METHF	2	-0.632668	0.736525	-1.58651	1.14582	0
METTHFm	6	-0.13951	0.555476	-1.1716	1.05405	0
MTHFm	2	-0.910538	0.818731	-1.79567	0.643922	0
METHFm	2	-0.632668	0.736525	-1.58651	1.14582	0

FTHFm	1	-1.21064	0.886983	-2.39673	0	0
FTHF	2	-1.12051	0.868753	-1.95372	0.626512	0
THFm	3	0.362909	0.358337	-0.887312	1.40762	0
AHTD	2	2.31592	0.0102814	0.632929	0.50589	0
DHP	2	1.68934	0.0455775	0.161292	1.17288	0
AHHMP	1	0.415506	0.338886	-0.668062	0	0
GLAL	1	0.415506	0.338886	-0.668062	0	0
CHOR	3	-0.405053	0.657281	-1.35967	1.34575	0
GLN	13	-1.04878	0.85286	-1.42185	0.874868	0
PABA	1	0.415506	0.338886	-0.668062	0	0
AHHMD	1	0.415506	0.338886	-0.668062	0	0
DHPT	1	0.415506	0.338886	-0.668062	0	0
DHF	1	-0.377166	0.646975	-1.51071	0	0
THF	8	-0.318266	0.624859	-1.23104	0.875298	0
MTHF	1	-0.94271	0.827085	-2.11191	0	0
THFG	2	-0.866539	0.806903	-1.76255	0.979794	0
OIVAL	2	-0.124281	0.549454	-1.20384	0.178097	0
AKP	1	-0.206962	0.58198	-1.32978	0	0
PANT	1	-0.781564	0.782765	-1.94061	0	0

AKPm	1	-0.404434	0.657053	-1.5397	0	0
PANTm	1	-0.404434	0.657053	-1.5397	0	0
bALA	1	-0.781564	0.782765	-1.94061	0	0
PNTO	2	1.32447	0.092674	-0.11335	2.58413	1
4PPNTO	1	2.6562	0.00395134	1.71391	0	1
CTP	7	-0.652295	0.742895	-1.37398	0.929495	0
CYS	7	-0.679372	0.751549	-1.3849	0.461576	0
CMP	8	-0.694797	0.756409	-1.37314	0.740972	0
ASP	18	0.681054	0.247919	-0.939528	0.643697	0
PAP	3	-0.391101	0.652139	-1.35109	0.957237	0
ACP	10	-1.2085	0.886573	-1.5191	1.00923	0
ALA	6	-1.02792	0.848006	-1.55851	0.802836	0
CHCOA	1	0.316743	0.375719	-0.773051	0	0
AONA	1	0.316743	0.375719	-0.773051	0	0
DTB	1	-0.377166	0.646975	-1.51071	0	0
BT	1	-0.377166	0.646975	-1.51071	0	0
ETH	4	1.84497	0.0325209	-0.127346	2.06431	1
ETHm	4	1.84497	0.0325209	-0.127346	2.06431	1
ACALm	9	1.698	0.0447537	-0.50668	1.47594	1

ACm	9	0.228576	0.409599	-1.02962	1.16119	0
AMPm	9	-0.794526	0.786555	-1.39373	0.740724	0
PPIm	11	-0.97968	0.836378	-1.42652	0.670901	0
ACTPm	1	-1.07354	0.858486	-2.25099	0	0
GLYN	11	-0.129329	0.551451	-1.15268	0.820634	0
GL	14	1.01171	0.155839	-0.822158	1.3374	1
GLYAL	9	1.11979	0.131402	-0.712457	1.62891	1
O2	34	0.941504	0.173223	-0.938423	1.42994	2
H2O2	10	1.66484	0.0479721	-0.5488	2.39615	2
GL3P	7	-0.378691	0.647541	-1.26363	0.632325	0
TAR	2	-1.13389	0.871579	-1.96379	0.412677	0
OXGLY	2	-1.13389	0.871579	-1.96379	0.412677	0
G	1	-0.166475	0.566109	-1.28674	0	0
E	6	0.155539	0.438198	-1.0431	0.997345	0
EOL	6	0.155539	0.438198	-1.0431	0.997345	0
LXUL	6	-1.13533	0.871882	-1.60529	0.662812	0
XOL	6	0.168547	0.433077	-1.03743	1.00532	0
XUL	7	0.222437	0.411987	-1.02117	0.918732	0
AOL	6	0.168547	0.433077	-1.03743	1.00532	0

XYL	3	0.833612	0.20225	-0.597793	1.11427	0
ARAB	2	0.15315	0.43914	-0.995017	0.0721831	0
ARABLAC	2	0.15315	0.43914	-0.995017	0.0721831	0
LAOL	3	-1.01286	0.844438	-1.73352	0.586303	0
RIB	3	0.848821	0.19799	-0.588438	0.36448	0
R1P	2	-0.0867804	0.534577	-1.17562	0.224878	0
RL	2	-1.54375	0.938676	-2.2723	0.815342	0
O2e	7	3.49662	0.000235592	0.29942	2.50326	2
GLCN15LACe	3	0.732805	0.231839	-0.659797	0.325561	0
H2O2e	7	3.49662	0.000235592	0.29942	2.50326	2
H2Oe	19	3.03413	0.00120613	-0.366898	1.83611	3
GLCNTe	1	0.428033	0.334313	-0.654745	0	0
GLCN15LAC	1	0.428033	0.334313	-0.654745	0	0
GLCNT	6	1.21119	0.11291	-0.583337	0.91848	0
GLAC	18	5.25885	2.32964e-007	0.214035	1.96317	4
GALOL	5	0.143132	0.443093	-1.0425	1.11506	0
GAL1P	2	-0.247583	0.597772	-1.29665	0.0617928	0
UTP	7	-0.487018	0.686877	-1.30732	1.02245	0
UDPGAL	4	-0.184398	0.573149	-1.20895	0.349293	0

UDPG	10	-0.185882	0.573732	-1.17377	0.935463	0
G1P	4	1.03056	0.151375	-0.561409	0.981091	0
MELI	4	6.40289	2.43299e-010	2.30191	1.10996	2
GALN14LAC	3	1.63595	0.0509246	-0.104289	3.48156	1
GALNT	2	-1.0161	0.845209	-1.87513	0.182542	0
2D3DGALT	2	-1.0161	0.845209	-1.87513	0.182542	0
SOR	8	0.954738	0.169855	-0.750632	0.953748	0
SOT	4	0.0584536	0.476694	-1.07952	1.04992	0
MAN6P	5	-0.496237	0.690136	-1.34743	0.152278	0
MAN	4	0.188974	0.425057	-1.00995	0.626317	0
FRU	8	-0.0945449	0.537662	-1.14661	0.674784	0
MNT6P	1	1.59411	0.0554558	0.584851	0	0
MNT	4	-0.323857	0.626977	-1.28328	0.787633	0
F26P	1	-0.431344	0.666891	-1.56831	0	0
MAN1P	1	-0.551415	0.709326	-1.69595	0	0
GDPMAN	2	-1.23967	0.892452	-2.04341	0.491392	0
IDOL	3	0.946056	0.17206	-0.528631	1.0144	0
UDP	17	-0.569883	0.715622	-1.25889	0.936853	0
TRE6P	3	-0.514801	0.696654	-1.42717	0.288226	0

TRE	3	-0.868336	0.807395	-1.64462	0.513516	0
MLT	5	-0.410849	0.659408	-1.30671	1.1459	0
MLTe	5	-0.410849	0.659408	-1.30671	1.1459	0
LACT	4	2.83995	0.00225601	0.402954	1.19903	1
LACTe	4	2.83995	0.00225601	0.402954	1.19903	1
GLACe	5	2.96259	0.00152531	0.302144	1.06257	1
13GLUCAN	2	-1.35344	0.912043	-2.12905	0.512757	0
GA6P	4	-0.885083	0.811944	-1.5824	0.561841	0
NAGA6P	2	-1.36399	0.913715	-2.13699	0.35905	0
NAGA1P	2	-1.26971	0.897906	-2.06603	0.258687	0
UDPNAG	9	-1.23663	0.891888	-1.55107	0.82629	0
CHIT	13	0.527829	0.298809	-0.954663	1.06688	0
NAG	5	2.03553	0.020899	-0.13999	0.915443	0
GLCN	3	-0.329019	0.628929	-1.3129	0.194254	0
13GLUCANe	12	-0.853881	0.803415	-1.37437	0.60761	0
GLYCOGEN	2	2.62449	0.00433893	0.865197	0.122281	0
STARe	2	-0.710086	0.761175	-1.64479	1.05514	0
GLYCOGENe	2	-0.710086	0.761175	-1.64479	1.05514	0
AMYLSe	1	1.76667	0.0386415	0.768295	0	0

AMYLPe	1	1.76667	0.0386415	0.768295	0	0
CELLUe	8	-0.742157	0.771004	-1.39101	0.610993	0
CELLOBe	8	-0.742157	0.771004	-1.39101	0.610993	0
CELLOTe	4	-0.174734	0.569356	-1.2038	0.612975	0
MANNANe	6	0.622792	0.266711	-0.839598	1.37666	0
MANe	7	0.934193	0.175102	-0.734098	1.28733	0
PECTATEe	1	1.94792	0.0257121	0.960972	0	0
GALUNTe	1	1.94792	0.0257121	0.960972	0	0
ARABINe	5	0.746901	0.227562	-0.754556	0.86233	0
LARABe	5	0.746901	0.227562	-0.754556	0.86233	0
XYLANe	6	2.19379	0.0141253	-0.155398	1.11521	1
XYLe	6	2.19379	0.0141253	-0.155398	1.11521	1
H+_PO_mit	8	-1.2756	0.898951	-1.59233	0.442887	0
H+_PO	8	-1.2756	0.898951	-1.59233	0.442887	0
FERIm	5	-1.78639	0.962982	-1.96272	0.643088	0
FEROm	5	-1.78639	0.962982	-1.96272	0.643088	0
O2m	1	-0.526095	0.700589	-1.66903	0	0
K	1	-0.521914	0.699135	-1.66459	0	0
Km	1	-0.521914	0.699135	-1.66459	0	0

Ca	1	-0.0343065	0.513684	-1.14623	0	0
Cam	1	-0.0343065	0.513684	-1.14623	0	0
LLACm	6	0.50803	0.305716	-0.889579	0.871373	0
LACm	1	-1.4494	0.926388	-2.65055	0	0
GLUm	10	0.080112	0.468074	-1.08395	0.677075	0
ASPm	6	0.546757	0.292273	-0.872712	0.848631	0
ALAm	1	-0.690191	0.754963	-1.84347	0	0
ASN	5	-0.452375	0.6745	-1.32651	0.427063	0
SAM	12	-1.13502	0.871816	-1.46107	0.733707	0
HCYS	8	-0.668245	0.748011	-1.36312	0.782745	0
SAH	8	-0.823963	0.79502	-1.42189	0.697788	0
MET	4	-1.06992	0.857672	-1.68091	1.24095	0
TRNAm	4	0.0179326	0.492846	-1.10111	0.932833	0
ASPTRNAm	3	0.245704	0.402956	-0.959403	1.08847	0
TRNA	3	0.479226	0.315889	-0.815768	0.933531	0
ASPTRNA	3	0.479226	0.315889	-0.815768	0.933531	0
NH3	29	-0.606695	0.727973	-1.2318	0.930103	0
NAGLUm	2	-1.10372	0.865144	-1.94108	0.435381	0
NAGLUPm	1	-1.07162	0.858054	-2.24895	0	0

NAGLUSm	2	-1.02234	0.846691	-1.87983	0.52201	0
NAORNm	2	-0.613344	0.730175	-1.57197	0.0866293	0
ORNm	1	-0.492412	0.688786	-1.63322	0	0
CAP	5	2.52903	0.00571888	0.095372	2.11161	1
ORN	5	5.99052	3.34504e-009	1.74621	2.98307	3
CITR	2	3.50091	0.000231838	1.52489	3.10297	1
GLUGSAL	3	2.19331	0.0141424	0.23853	3.0142	1
ARGSUCC	2	0.25021	0.401213	-0.921959	0.357397	0
ARG	3	2.54975	0.00539003	0.457766	3.35307	1
PTRSC	2	-0.728948	0.766983	-1.65898	0.20969	0
DSAM	2	-1.00529	0.842621	-1.86699	0.0844741	0
SPRMD	3	-0.388295	0.651101	-1.34936	0.556404	0
5MTA	1	-0.656125	0.744128	-1.80726	0	0
SPRM	2	-0.210427	0.583333	-1.26869	0.761656	0
GBAD	3	0.79717	0.212676	-0.620207	1.02559	0
GBAT	3	0.79717	0.212676	-0.620207	1.02559	0
UREA	3	3.22915	0.000620789	0.875653	3.10161	1
ATRNA	1	-0.617761	0.731634	-1.76648	0	0
ALTRNA	1	-0.617761	0.731634	-1.76648	0	0

DAPRP	1	-0.377166	0.646975	-1.51071	0	0
SLF	1	-0.28048	0.610445	-1.40793	0	0
APS	2	-0.633584	0.736824	-1.5872	0.253531	0
PAPS	2	-1.10915	0.866317	-1.94517	0.252707	0
SER	11	-0.481618	0.684961	-1.26613	0.61461	0
ASER	5	-0.192686	0.576398	-1.20266	0.272535	0
H2S	5	-1.10853	0.866184	-1.63944	0.635923	0
RTHIO	4	-0.716236	0.763077	-1.49241	0.552901	0
OTHIO	4	-0.716236	0.763077	-1.49241	0.552901	0
H2SO3	2	-1.37418	0.915308	-2.14466	0.0294182	0
GLUGSALm	3	0.496132	0.309901	-0.805369	0.830606	0
P5Cm	4	1.88633	0.0296252	-0.105303	1.55574	1
PHP	1	-1.20513	0.885924	-2.39088	0	0
GLYm	2	1.29889	0.0969908	-0.132602	0.738422	0
GLY	11	-0.493222	0.689072	-1.26986	1.11622	0
GLX	2	-0.780731	0.78252	-1.69796	0.205782	0
BASP	2	-0.377234	0.647	-1.39424	0.164709	0
ASPSA	2	-0.500279	0.691561	-1.48686	0.295691	0
HSER	3	-0.514851	0.696671	-1.4272	0.243677	0

PHSER	2	0.100287	0.460058	-1.03481	0.262809	0
THR	8	-0.164166	0.5652	-1.17289	0.912015	0
LLCT	5	-0.387637	0.650858	-1.29564	0.270269	0
OBUT	4	-1.16078	0.877135	-1.72934	0.636595	0
THRm	3	-1.24054	0.892613	-1.87356	0.528521	0
NH3m	3	-1.24054	0.892613	-1.87356	0.528521	0
OBUTm	4	-1.09934	0.86419	-1.69659	0.558121	0
PRPP	9	0.631231	0.263945	-0.886326	0.620578	0
PRBATP	3	-0.0801013	0.531922	-1.1598	0.620628	0
PRBAMP	2	-0.538168	0.70477	-1.51538	0.108258	0
PRFP	3	0.148076	0.441141	-1.01945	0.862382	0
PRLP	2	1.1197	0.131422	-0.267485	0.339264	0
DIMGP	2	0.962027	0.168018	-0.386164	0.171426	0
IMACP	2	0.911912	0.180907	-0.423886	0.224772	0
HISOLP	2	-0.038647	0.515414	-1.13939	0.787097	0
HISOL	3	-0.756067	0.775195	-1.57557	0.129336	0
HIS	4	-0.809264	0.790818	-1.54199	0.309851	0
AICAR	4	-0.621927	0.733005	-1.44214	0.904775	0
HTRNA	1	-0.083768	0.53338	-1.19881	0	0

HHTRNA	1	-0.083768	0.53338	-1.19881	0	0
MHIS	1	-0.779461	0.782146	-1.93837	0	0
OICAPm	1	0.0299662	0.488047	-1.07791	0	0
LEUm	1	0.0299662	0.488047	-1.07791	0	0
OMVALm	2	-0.214399	0.584882	-1.27168	0.274028	0
ILEm	1	0.0299662	0.488047	-1.07791	0	0
OMVAL	1	0.0299662	0.488047	-1.07791	0	0
ILE	1	0.0299662	0.488047	-1.07791	0	0
VAL	4	-0.230012	0.590959	-1.23326	0.393994	0
OICAP	3	-0.352396	0.637729	-1.32728	0.298169	0
LEU	1	0.0299662	0.488047	-1.07791	0	0
ABUTm	2	-0.322023	0.626282	-1.35269	0.264474	0
ACLACm	2	-0.322023	0.626282	-1.35269	0.264474	0
DHVALm	2	-0.521148	0.698868	-1.50257	0.0525046	0
DHMVAm	2	-0.521148	0.698868	-1.50257	0.0525046	0
OIVALm	2	-0.501893	0.692129	-1.48808	0.0320079	0
IPPMALm	1	-0.377166	0.646975	-1.51071	0	0
CBHCAP	1	-1.25012	0.894372	-2.43871	0	0
IPPMAL	3	-1.08986	0.862113	-1.78088	0.605653	0

PPMAL	1	-1.25012	0.894372	-2.43871	0	0
HCITm	1	-1.20513	0.885924	-2.39088	0	0
HACNm	2	-0.96244	0.832086	-1.83474	0.786505	0
HICITm	2	-0.138976	0.555265	-1.2149	0.0900709	0
OXAm	1	-0.0389908	0.515551	-1.15121	0	0
MICIT	1	-0.492412	0.688786	-1.63322	0	0
AKA	1	-0.147187	0.558508	-1.26623	0	0
AMA	4	0.283928	0.388233	-0.959343	0.216175	0
AMASA	2	0.574398	0.282849	-0.677938	0.134511	0
SACP	2	0.354201	0.361594	-0.843683	0.368911	0
LYS	3	-0.678406	0.751243	-1.5278	0.424044	0
LTRNA	2	-0.835826	0.798373	-1.73943	0.3015	0
LLTRNA	2	-0.835826	0.798373	-1.73943	0.3015	0
LYSm	2	-0.835826	0.798373	-1.73943	0.3015	0
LTRNAm	2	-0.835826	0.798373	-1.73943	0.3015	0
LLTRNAm	2	-0.835826	0.798373	-1.73943	0.3015	0
ADN	7	0.524292	0.300038	-0.899425	0.653787	0
MTHPTGLU	1	-0.94271	0.827085	-2.11191	0	0
THPTGLU	1	-0.94271	0.827085	-2.11191	0	0

OAHSER	2	-1.01985	0.8461	-1.87795	0.725391	0
METH	1	-1.20513	0.885924	-2.39088	0	0
OSLHSER	1	-1.47866	0.930384	-2.68165	0	0
CALH	1	-0.453569	0.674931	-1.59193	0	0
DPTH	1	-0.453569	0.674931	-1.59193	0	0
3DDAH7P	3	-0.367904	0.643527	-1.33682	0.328578	0
DQT	3	0.0244649	0.490241	-1.09548	0.39268	0
DHSK	2	0.295501	0.383806	-0.887867	0.223096	0
QT	1	-0.377166	0.646975	-1.51071	0	0
SME	1	0.0603413	0.475942	-1.04562	0	0
SME5P	1	0.0603413	0.475942	-1.04562	0	0
3PSME	2	0.906859	0.182241	-0.427689	0.873886	0
PHEN	2	-1.46674	0.928776	-2.21433	0.249679	0
PHPYR	2	-0.485018	0.686168	-1.47538	0.223231	0
PHE	1	-0.492412	0.688786	-1.63322	0	0
4HPP	6	-0.557833	0.711521	-1.35378	0.632475	0
TYR	4	-0.242079	0.59564	-1.23969	0.422388	0
AN	4	-1.31975	0.906541	-1.81406	0.955466	0
NPRAN	2	-1.35754	0.912695	-2.13213	0.168092	0

CPAD5P	1	-1.07354	0.858486	-2.25099	0	0
IGP	2	0.0939779	0.462563	-1.03956	1.71323	0
TRP	3	0.491837	0.311417	-0.808011	1.20795	0
FKYN	3	0.00301427	0.498797	-1.10868	0.920048	0
KYN	4	-0.726063	0.7661	-1.49764	1.07833	0
HKYN	3	-1.04532	0.852063	-1.75349	1.16253	0
HAN	2	-0.525381	0.700341	-1.50576	1.52798	0
CMUSA	1	0.286517	0.387241	-0.805183	0	0
AM6SA	2	-0.0537329	0.521426	-1.15074	0.488692	0
AMUCO	1	-0.363608	0.641925	-1.4963	0	0
HOMOGEN	2	0.526623	0.299228	-0.713898	0.0836553	0
MACAC	2	0.714507	0.237457	-0.572476	0.116346	0
FUACAC	2	0.608069	0.271571	-0.652593	0.229649	0
ACTAC	1	0.277302	0.390774	-0.81498	0	0
TRPm	1	-0.391774	0.652387	-1.52624	0	0
TRPTRNAm	1	-0.391774	0.652387	-1.52624	0	0
PAD	3	1.7852	0.0371147	-0.0124936	0.643446	0
PAC	3	1.7852	0.0371147	-0.0124936	0.643446	0
IAD	3	1.7852	0.0371147	-0.0124936	0.643446	0

IAC	3	1.7852	0.0371147	-0.0124936	0.643446	0
ASPERMD	2	0.458461	0.323311	-0.765205	0.0496249	0
APRUT	1	0.291116	0.385481	-0.800295	0	0
APROA	1	0.291116	0.385481	-0.800295	0	0
GABAL	1	0.291116	0.385481	-0.800295	0	0
ASPRM	2	0.458461	0.323311	-0.765205	0.0496249	0
GLUP	2	-1.10467	0.865349	-1.9418	0.635099	0
P5C	1	-0.551415	0.709326	-1.69595	0	0
PRO	1	-0.551415	0.709326	-1.69595	0	0
PHC	1	-0.551415	0.709326	-1.69595	0	0
HPRO	1	-0.551415	0.709326	-1.69595	0	0
PROm	1	2.92053	0.0017472	1.9949	0	1
GABALm	5	0.630337	0.264237	-0.810147	0.949629	0
GABAm	5	0.630337	0.264237	-0.810147	0.949629	0
LACALm	5	0.630337	0.264237	-0.810147	0.949629	0
APROP	1	-0.724153	0.765514	-1.87958	0	0
TCOA	1	-1.05169	0.85353	-2.22777	0	0
GLP	1	-1.05169	0.85353	-2.22777	0	0
TGLP	1	-1.05169	0.85353	-2.22777	0	0

PEPD	1	-0.128632	0.551176	-1.24651	0	0
APEP	1	-0.128632	0.551176	-1.24651	0	0
GC	2	-1.7795	0.962421	-2.44975	0.283979	0
OGT	2	-0.847553	0.801657	-1.74826	0.908802	0
cAMP	3	-1.38426	0.91686	-1.96196	0.529628	0
GMP	6	0.677881	0.248923	-0.815605	0.905037	0
DGMP	2	0.585782	0.279011	-0.669369	1.18984	0
DGDP	2	0.514329	0.303511	-0.723152	0.626304	0
DATP	2	0.11495	0.454242	-1.02377	1.05144	0
DADP	4	1.48796	0.0683806	-0.317624	0.946746	0
PRAM	1	-1.2658	0.897207	-2.45537	0	0
GAR	1	-1.2658	0.897207	-2.45537	0	0
FGAR	1	-0.377166	0.646975	-1.51071	0	0
FGAM	2	-1.15946	0.876867	-1.98304	0.667974	0
AIR	2	-0.40905	0.658748	-1.41819	1.46679	0
CAIR	2	0.505946	0.306447	-0.729462	0.492779	0
SAICAR	3	-0.811167	0.791365	-1.60946	0.901679	0
PRFICA	1	-0.377166	0.646975	-1.51071	0	0
IMP	9	0.725423	0.234096	-0.852805	0.806963	0

ASUC	3	-0.2963	0.616499	-1.29278	1.27236	0
XMP	5	0.675114	0.249802	-0.788792	0.828498	0
cdAMP	1	-0.677618	0.750993	-1.83011	0	0
DAMP	5	1.0739	0.141435	-0.598607	1.17514	0
cIMP	1	-0.677618	0.750993	-1.83011	0	0
cGMP	1	-0.677618	0.750993	-1.83011	0	0
cCMP	1	-0.677618	0.750993	-1.83011	0	0
ATN	1	-1.02037	0.846223	-2.19446	0	0
ATT	2	-1.11876	0.868378	-1.9524	0.342331	0
UGC	2	-0.692293	0.755623	-1.63139	0.11164	0
CAASP	3	0.674016	0.25015	-0.695957	0.795144	0
DOROA	1	-0.768838	0.779005	-1.92708	0	0
OROA	2	-0.256761	0.601318	-1.30356	0.881783	0
OMP	2	0.348033	0.363908	-0.848326	0.237981	0
UMP	6	-0.11367	0.54525	-1.16034	0.686487	0
URA	6	-0.817496	0.793177	-1.46687	0.310404	0
CYTS	2	-0.254179	0.600322	-1.30162	0.366219	0
URI	4	-0.151649	0.560268	-1.1915	0.916353	0
CYTD	4	-0.151649	0.560268	-1.1915	0.916353	0

DU	4	0.137585	0.445284	-1.03734	0.867855	0
DR1P	1	0.0876363	0.465083	-1.0166	0	0
DT	3	0.529064	0.29838	-0.785114	0.864898	0
THY	1	0.0876363	0.465083	-1.0166	0	0
DC	3	0.107755	0.457095	-1.04425	1.06277	0
DTMP	4	-0.115873	0.546124	-1.17243	0.909632	0
DTDP	2	0.0664534	0.473508	-1.06027	1.10307	0
OTHIOm	1	0.18557	0.426391	-0.912495	0	0
RTHIOm	1	0.18557	0.426391	-0.912495	0	0
DUTP	3	0.0705358	0.471884	-1.06714	0.747268	0
DUMP	5	0.0367836	0.485329	-1.09322	0.722766	0
DCMP	3	0.28727	0.386953	-0.933836	0.95796	0
DCDP	3	0.228975	0.409444	-0.969692	0.615202	0
CDP	4	0.571044	0.283985	-0.806317	0.599234	0
PURI5P	3	-0.748819	0.773017	-1.57111	0.343302	0
AD	5	-0.167886	0.566664	-1.19083	0.295818	0
INS	5	0.678071	0.248863	-0.787382	0.742497	0
DA	4	0.328873	0.371126	-0.935388	0.767482	0
DIN	2	-0.121046	0.548173	-1.20141	0.261353	0

HYXN	3	0.511805	0.304394	-0.79573	0.726552	0
DG	4	-0.0136694	0.505453	-1.11796	0.970481	0
GN	3	0.767056	0.221524	-0.63873	0.568953	0
GSN	6	0.414304	0.339326	-0.930398	0.837464	0
XAN	2	0.810144	0.208929	-0.500488	0.729897	0
XTSINE	3	0.529064	0.29838	-0.785114	0.864898	0
ITP	2	1.94858	0.0256729	0.356427	0.900453	0
IDP	2	1.94858	0.0256729	0.356427	0.900453	0
ITPm	1	0.275088	0.391624	-0.817333	0	0
IDPm	1	0.275088	0.391624	-0.817333	0	0
DGTP	3	-0.451125	0.67405	-1.38801	0.975074	0
DUDP	3	0.228975	0.409444	-0.969692	0.615202	0
DCTP	2	0.11495	0.454242	-1.02377	1.05144	0
DTTP	1	0.78028	0.217613	-0.280289	0	0
LCCA	4	1.46709	0.0711756	-0.328746	0.706237	0
ACOA	7	0.811816	0.208449	-0.783457	0.757666	0
HACOA	3	-0.295288	0.616113	-1.29216	0.357481	0
OACOA	6	-0.528358	0.701375	-1.34095	0.240053	0
AACCOA	3	1.85085	0.0320958	0.027887	1.73163	1

AACCOAm	2	0.25252	0.40032	-0.92022	0.776931	0
ACACPm	2	-0.117601	0.546808	-1.19881	0.000123979	0
MALACPm	2	-0.117601	0.546808	-1.19881	0.000123979	0
C100ACPm	1	-0.083768	0.53338	-1.19881	0	0
ACPm	2	-0.117601	0.546808	-1.19881	0.000123979	0
C120ACPm	1	-0.083768	0.53338	-1.19881	0	0
C140ACPm	1	-0.083768	0.53338	-1.19881	0	0
C141ACPm	1	-0.083768	0.53338	-1.19881	0	0
C160ACPm	1	-0.083768	0.53338	-1.19881	0	0
C161ACPm	1	-0.083768	0.53338	-1.19881	0	0
C180ACPm	1	-0.083768	0.53338	-1.19881	0	0
C181ACPm	1	-0.083768	0.53338	-1.19881	0	0
C182ACPm	1	-0.083768	0.53338	-1.19881	0	0
C150ACPm	1	-0.083768	0.53338	-1.19881	0	0
C162ACPm	1	-0.083768	0.53338	-1.19881	0	0
C170ACPm	1	-0.083768	0.53338	-1.19881	0	0
C183ACPm	1	-0.083768	0.53338	-1.19881	0	0
C200ACPm	1	-0.083768	0.53338	-1.19881	0	0
MALCOA	7	-0.526384	0.700689	-1.3232	0.543838	0

MALACP	4	-1.23936	0.892395	-1.77122	0.81911	0
ACACP	4	-1.82798	0.966224	-2.08494	0.602098	0
3OACPm	1	-0.083768	0.53338	-1.19881	0	0
C100ACP	1	-1.24522	0.893474	-2.43349	0	0
C120ACP	9	0.0256287	0.489777	-1.10185	1.25051	0
C140ACP	9	0.0256287	0.489777	-1.10185	1.25051	0
C141ACP	9	0.0256287	0.489777	-1.10185	1.25051	0
C160ACP	9	0.0256287	0.489777	-1.10185	1.25051	0
C161ACP	9	0.0256287	0.489777	-1.10185	1.25051	0
C180ACP	9	0.0256287	0.489777	-1.10185	1.25051	0
C181ACP	9	0.0256287	0.489777	-1.10185	1.25051	0
C182ACP	9	0.0256287	0.489777	-1.10185	1.25051	0
3HPACP	3	-1.39531	0.918538	-1.96875	0.680286	0
2HDACP	3	-1.39531	0.918538	-1.96875	0.680286	0
AACP	3	-1.39531	0.918538	-1.96875	0.680286	0
23DAACP	3	-1.39531	0.918538	-1.96875	0.680286	0
C150ACP	6	0.416469	0.338534	-0.929456	1.54649	0
C162ACP	9	0.0256287	0.489777	-1.10185	1.25051	0
C170ACP	9	0.0256287	0.489777	-1.10185	1.25051	0

C183ACP	9	0.0256287	0.489777	-1.10185	1.25051	0
C200ACP	9	0.0256287	0.489777	-1.10185	1.25051	0
C140	1	0.0881578	0.464876	-1.01605	0	0
C160	1	0.0881578	0.464876	-1.01605	0	0
C180	1	0.0881578	0.464876	-1.01605	0	0
AGL3P	3	0.345752	0.364764	-0.897865	1.04075	0
AT3P2	2	0.696154	0.243166	-0.58629	1.25851	0
PA	5	-0.172296	0.568397	-1.19294	0.444106	0
PAm	1	0.165045	0.434454	-0.934314	0	0
CTPm	2	0.66824	0.25199	-0.607301	0.462466	0
CDPDGm	3	-0.310849	0.622042	-1.30173	0.687288	0
CDPDG	2	-0.000305926	0.500122	-1.11053	0.249201	0
PS	3	1.5713	0.0580563	-0.144056	0.856752	0
CMPm	3	-0.597239	0.724826	-1.47788	0.609337	0
PSm	1	0.438069	0.330668	-0.644076	0	0
PE	6	0.433574	0.332299	-0.922006	1.07848	0
PEm	1	0.438069	0.330668	-0.644076	0	0
PMME	2	-0.872378	0.808499	-1.76695	0.566632	0
PDME	1	-0.995109	0.840158	-2.16762	0	0

PC	3	-1.39257	0.918125	-1.96707	0.552113	0
CHO	1	-0.551415	0.709326	-1.69595	0	0
PCHO	2	-0.940571	0.826538	-1.81828	0.173	0
CDPCHO	2	-1.40219	0.919571	-2.16574	0.318392	0
DAGLY	11	0.424105	0.335745	-0.974452	1.12833	0
PETHM	2	0.127405	0.44931	-1.0144	0.729739	0
CDPETN	2	-1.1297	0.8707	-1.96064	0.608452	0
MI1P	2	-1.6332	0.948786	-2.33962	0.116076	0
MYOI	2	-0.87928	0.810375	-1.77214	0.686466	0
PINS	7	-0.0224819	0.508968	-1.11996	0.6437	0
PINSP	4	0.12761	0.449229	-1.04266	0.724166	0
PINS4P	3	0.123452	0.450875	-1.0346	0.774493	0
D45PI	3	-0.146856	0.558377	-1.20086	0.674509	0
TPI	2	0.362086	0.358644	-0.837748	0.34472	0
GL3Pm	1	-0.926452	0.822894	-2.09463	0	0
PGPm	2	-1.27784	0.899347	-2.07214	0.0317984	0
PGm	2	-0.468505	0.680288	-1.46295	0.829734	0
CLm	1	0.219679	0.41306	-0.876235	0	0
DGPP	2	0.333872	0.369238	-0.858985	0.182249	0

LPC	1	-0.21913	0.586725	-1.34271	0	0
LPE	1	-0.21913	0.586725	-1.34271	0	0
CDPm	2	0.317206	0.375544	-0.87153	0.836141	0
PALCOA	2	-1.24474	0.893387	-2.04723	0.28816	0
DHSPH	3	-1.15386	0.875722	-1.82025	0.442814	0
SPH	4	0.120432	0.45207	-1.04648	0.22775	0
PSPH	3	-0.327018	0.628173	-1.31167	0.551365	0
C260COA	1	-0.787945	0.784636	-1.94739	0	0
CER2	2	-0.822032	0.794471	-1.72905	0.308778	0
CER3	2	-0.753004	0.774276	-1.67709	0.235297	0
IPC	2	-1.33767	0.909498	-2.11718	0.387075	0
MIPC	2	-1.33767	0.909498	-2.11718	0.387075	0
MIP2C	1	-0.690191	0.754963	-1.84347	0	0
DHSP	3	0.562189	0.286994	-0.76474	0.23989	0
PHSP	1	0.137308	0.445394	-0.9638	0	0
C16A	1	0.575113	0.282607	-0.498392	0	0
H3MCOA	3	2.11297	0.0173015	0.189115	1.5842	1
MVL	3	0.898878	0.184359	-0.557649	0.543848	0
PMVL	2	-0.276793	0.609031	-1.31864	1.41768	0

PPMVL	2	-1.09308	0.862821	-1.93307	0.548736	0
IPPP	3	-1.62322	0.947729	-2.10894	0.488335	0
DMPP	2	-1.70129	0.955556	-2.39088	0.000687845	0
GPP	1	-1.20513	0.885924	-2.39088	0	0
FPP	1	-1.20513	0.885924	-2.39088	0	0
S23E	1	0.128042	0.449058	-0.973651	0	0
LNST	4	0.387438	0.349216	-0.904175	0.588766	0
IGST	5	0.356535	0.36072	-0.940728	0.553454	0
DMZYMST	5	-0.943394	0.82726	-1.56069	0.606234	0
IMZYMST	4	-1.31848	0.906329	-1.81339	0.403943	0
IIMZYMST	2	-0.383185	0.649209	-1.39872	0.158374	0
MZYMST	4	-1.21342	0.887516	-1.75739	0.470006	0
IZYMST	4	-1.31848	0.906329	-1.81339	0.403943	0
IIZYMST	2	-0.383185	0.649209	-1.39872	0.158374	0
ZYMST	2	-0.383185	0.649209	-1.39872	0.158374	0
FEST	2	-0.531963	0.702624	-1.51071	0.000332951	0
EPST	3	-0.568636	0.715198	-1.46029	0.165974	0
ERTROL	3	-0.451898	0.674329	-1.38848	0.17931	0
ERTEOL	2	0.121046	0.451827	-1.01918	0.390493	0

ERGOST	1	0.344954	0.365065	-0.743062	0	0
TAGLY	5	1.0109	0.156031	-0.628649	1.52016	0
MAGLY	4	0.235528	0.4069	-0.985139	1.49464	0
PHACAL	6	0.496247	0.30986	-0.894711	0.874266	0
PHAC	6	0.200532	0.420532	-1.0235	0.997272	0
PHACCOA	2	-0.480786	0.684666	-1.47219	0.874091	0
LLDACV	2	-0.236043	0.5933	-1.28797	0.486478	0
IPN	2	-0.176351	0.569991	-1.24304	0.55002	0
PENG	1	0.240489	0.404976	-0.854114	0	0
NOR	4	-0.113349	0.545123	-1.17108	0.294147	0
AVN	5	0.0307915	0.487718	-1.09608	0.534458	0
HAVN	4	0.39051	0.34808	-0.902538	0.655903	0
AVF	2	0.492691	0.311115	-0.739439	0.506888	0
VHA	2	0.0179527	0.492838	-1.09678	0.00152101	0
VERB	3	-0.806947	0.790151	-1.60687	0.18442	0
VERA	2	-0.521148	0.698868	-1.50257	0.0525046	0
DMST	2	-0.521148	0.698868	-1.50257	0.0525046	0
DHDMST	2	-0.521148	0.698868	-1.50257	0.0525046	0
ST	1	-0.39188	0.652427	-1.52635	0	0

DHST	1	-0.39188	0.652427	-1.52635	0	0
OMST	3	1.48844	0.0683174	-0.195022	1.50996	0
DHOMST	3	1.48844	0.0683174	-0.195022	1.50996	0
AFB1	2	2.10032	0.0178505	0.470644	1.37888	0
AFG1	2	2.10032	0.0178505	0.470644	1.37888	0
AFB2	2	2.10032	0.0178505	0.470644	1.37888	0
AFG2	2	2.10032	0.0178505	0.470644	1.37888	0
HNO3	3	0.213959	0.415289	-0.978928	1.13349	0
HNO2	2	0.80411	0.210667	-0.50503	1.1055	0
NH4OH	1	-0.166475	0.566109	-1.28674	0	0
UREAC	1	1.06543	0.14334	0.0228424	0	0
ACNL	1	-0.724153	0.765514	-1.87958	0	0
INAC	1	-0.724153	0.765514	-1.87958	0	0
NH3e	2	0.849099	0.197913	-0.471166	1.94073	0
HNO3e	2	-0.659527	0.745221	-1.60673	0.45254	0
FRUe	1	0.948843	0.17135	-0.101099	0	0
SORe	1	0.948843	0.17135	-0.101099	0	0

#Results for Up-regulated only genes

#Feature	Number of neighbors	Z-score	P-value	Average Z	StdDev Z	Significance count
DGLCe	2	0.217812	0.413788	-0.946345	0.510855	0
GLCe	3	-2.25398	0.987901	-2.4969	0.312704	0
DGLC	1	-0.977982	0.835958	-2.14941	0	0
GLC	3	-1.04578	0.852169	-1.75377	0.950252	0
bDGLC	2	-0.127456	0.55071	-1.20623	0.0848507	0
ATP	44	-0.381425	0.648556	-1.17289	0.96839	1
ADP	30	-0.487795	0.687153	-1.20655	1.08107	1
G6P	2	-0.127456	0.55071	-1.20623	0.0848507	0
bDG6P	2	-0.127456	0.55071	-1.20623	0.0848507	0
H2O	24	0.575095	0.282614	-0.985631	1.41544	1
PI	28	0.0511788	0.479592	-1.10088	1.26027	1
F6P	4	-0.279853	0.610205	-1.25982	0.322011	0
FDP	1	0.168102	0.433252	-0.931065	0	0
S7P	2	-0.269851	0.606363	-1.31342	0.540726	0
S17P	1	0.168102	0.433252	-0.931065	0	0
T3P2	3	-0.80831	0.790544	-1.6077	0.322322	0
T3P1	6	1.4597	0.0721867	-0.475109	2.21126	1
E4P	3	-0.720242	0.764312	-1.55354	0.244003	0

NAD	32	-0.780544	0.782465	-1.25894	0.962922	1
13PDG	3	-1.09871	0.864053	-1.78632	0.548971	0
NADH	32	-0.780544	0.782465	-1.25894	0.962922	1
3PG	3	-1.23205	0.891035	-1.86834	0.554436	0
2PG	1	-0.769139	0.779095	-1.9274	0	0
23PDG	1	-0.769139	0.779095	-1.9274	0	0
PEP	3	-0.0207004	0.508258	-1.12326	0.65677	0
PYR	8	-1.12829	0.870401	-1.53673	0.797285	0
CO2	17	0.718717	0.236158	-0.924796	1.02033	0
OA	8	0.627521	0.265159	-0.874118	0.858732	0
ATPm	10	-1.72312	0.957566	-1.69288	0.951246	0
PYRm	4	-0.313455	0.623032	-1.27773	1.0816	0
CO2m	7	-0.473381	0.682029	-1.30182	0.826809	0
ADPm	6	-1.97086	0.97563	-1.96918	0.868554	0
PIm	5	-1.82666	0.966125	-1.98193	0.813002	0
OAm	6	0.244778	0.403314	-1.00423	0.989806	0
GTP	7	2.20283	0.0138033	-0.222411	0.811471	0
GDP	6	2.05228	0.020071	-0.217027	0.696016	0
NADP	15	0.106214	0.457706	-1.0818	0.819828	0

D6PGL	1	-0.0447888	0.517862	-1.15738	0	0
NADPH	15	0.106214	0.457706	-1.0818	0.819828	0
D6PGC	1	-0.0447888	0.517862	-1.15738	0	0
RL5P	1	-0.0835647	0.533299	-1.1986	0	0
XUL5P	3	2.25242	0.012148	0.274884	2.99305	1
R5P	8	0.190196	0.424578	-1.03916	0.638669	0
ACTP	2	2.45021	0.00713866	0.734012	4.22143	1
ACCOAm	8	0.253748	0.399845	-1.01517	0.889623	0
H2Om	10	-0.637304	0.738037	-1.32621	1.1573	0
CITm	4	-1.20072	0.88507	-1.75062	0.702751	0
COAm	10	-0.078458	0.531268	-1.1375	0.832287	0
ACOm	2	-1.4697	0.929179	-2.21656	0.770956	0
ICITm	3	-1.66435	0.951979	-2.13424	0.563488	0
NADm	10	2.96618	0.0015076	-0.109349	1.56493	2
AKGm	5	0.128785	0.448764	-1.04935	0.741461	0
NADHm	10	2.96618	0.0015076	-0.109349	1.56493	2
ICIT	1	-0.808827	0.790693	-1.96959	0	0
AKG	11	1.42368	0.0772691	-0.652552	1.606	1
NADPm	9	-0.0802916	0.531997	-1.13955	0.940831	0

NADPHm	9	-0.0802916	0.531997	-1.13955	0.940831	0
ICITg	1	2.96685	0.00150436	2.04414	0	1
LIPOm	1	0.132475	0.447304	-0.968938	0	0
SUCDLIP	2	-0.103809	0.54134	-1.18843	0.310414	0
SUCCOAm	2	-0.686173	0.753698	-1.62679	0.309512	0
DHLIPOm	1	-0.28048	0.610445	-1.40793	0	0
GDPm	1	-0.692235	0.755605	-1.84565	0	0
GTPm	1	-0.692235	0.755605	-1.84565	0	0
SUCCm	2	-0.739016	0.770051	-1.66656	0.253261	0
Qm	6	-1.41138	0.920934	-1.72552	0.366601	0
FUMm	2	-0.281825	0.610961	-1.32243	0.233417	0
QH2m	6	-1.41138	0.920934	-1.72552	0.366601	0
FADH2m	1	-1.20513	0.885924	-2.39088	0	0
FADm	1	-1.20513	0.885924	-2.39088	0	0
FUM	3	-0.0544037	0.521693	-1.14399	0.0391339	0
SUCC	3	-1.41798	0.921902	-1.9827	0.605309	0
MALm	2	0.700959	0.241664	-0.582673	0.812755	0
MAL	3	0.623031	0.266632	-0.727317	0.626937	0
MALg	1	1.84428	0.0325715	0.85079	0	0

SUCCg	1	2.96685	0.00150436	2.04414	0	1
GLXg	2	3.39805	0.000339343	1.44746	0.843824	1
ACCOAg	2	1.89101	0.0293112	0.313099	0.760411	0
H2Og	1	1.84428	0.0325715	0.85079	0	0
COAg	2	1.89101	0.0293112	0.313099	0.760411	0
Hm	7	-0.0668661	0.526656	-1.13786	1.03911	0
COA	16	0.593849	0.276306	-0.952437	1.33871	1
ACCOA	11	1.24328	0.106882	-0.710647	1.44624	1
CAR	2	1.45265	0.0731606	-0.0168654	1.40085	0
ACAR	2	1.45265	0.0731606	-0.0168654	1.40085	0
ACARm	2	0.656676	0.255694	-0.616005	0.553541	0
CARm	2	0.656676	0.255694	-0.616005	0.553541	0
ACARg	2	0.656676	0.255694	-0.616005	0.553541	0
CARg	2	0.656676	0.255694	-0.616005	0.553541	0
OXAL	1	0.35374	0.361767	-0.733722	0	0
AC	7	0.213136	0.41561	-1.02493	1.23161	0
FOR	1	1.30283	0.0963158	0.275211	0	0
FORm	1	-1.21064	0.886983	-2.39673	0	0
METHOL	2	1.93352	0.0265859	0.345095	3.19871	1

FALD	3	1.26055	0.103735	-0.335192	2.55034	1
ACAL	4	1.8482	0.0322865	-0.125623	2.06057	1
RGT	3	-1.96537	0.975314	-2.31939	0.372097	0
FGT	1	-0.759106	0.776105	-1.91673	0	0
H+	6	0.095379	0.462007	-1.0693	0.780223	0
HCIT	1	1.02478	0.152734	-0.0203778	0	0
LACAL	2	0.6828	0.247367	-0.596342	1.27273	0
LAC	2	0.6828	0.247367	-0.596342	1.27273	0
LLAC	1	0.0876363	0.465083	-1.0166	0	0
PROP	1	-0.453569	0.674931	-1.59193	0	0
AMP	21	0.250085	0.401261	-1.05282	0.84726	0
PPI	20	-0.284493	0.611984	-1.17919	0.661848	0
PROPCOA	2	-0.500191	0.69153	-1.4868	0.148685	0
2MCIT	1	-0.255769	0.600935	-1.38166	0	0
GLU	20	-0.166385	0.566073	-1.15095	1.44191	1
SUCCSAL	1	-0.492412	0.688786	-1.63322	0	0
METTHF	4	0.760125	0.22359	-0.705542	0.764626	0
METHF	2	-0.632668	0.736525	-1.58651	1.14582	0
METTHFm	5	-0.344295	0.634688	-1.27497	1.14395	0

MTHFm	2	-0.910538	0.818731	-1.79567	0.643922	0
METHFm	2	-0.632668	0.736525	-1.58651	1.14582	0
FTHFm	1	-1.21064	0.886983	-2.39673	0	0
FTHF	2	-1.12051	0.868753	-1.95372	0.626512	0
THFm	2	0.141752	0.443638	-1.0036	1.97019	0
AHTD	1	1.30283	0.0963158	0.275211	0	0
CHOR	2	0.119143	0.452581	-1.02061	1.71241	0
GLN	9	-1.0212	0.84642	-1.4744	0.997601	0
THF	6	-0.558282	0.711674	-1.35398	0.991384	0
MTHF	1	-0.94271	0.827085	-2.11191	0	0
THFG	1	-1.2658	0.897207	-2.45537	0	0
OIVAL	1	-0.206962	0.58198	-1.32978	0	0
AKP	1	-0.206962	0.58198	-1.32978	0	0
PANT	1	-0.781564	0.782765	-1.94061	0	0
AKPm	1	-0.404434	0.657053	-1.5397	0	0
PANTm	1	-0.404434	0.657053	-1.5397	0	0
bALA	1	-0.781564	0.782765	-1.94061	0	0
PNTO	2	1.32447	0.092674	-0.11335	2.58413	1
4PPNTO	1	2.6562	0.00395134	1.71391	0	1

CTP	4	-0.442442	0.670915	-1.34648	1.26601	0
CYS	3	-0.963511	0.832354	-1.70316	0.479455	0
CMP	4	-1.2082	0.886514	-1.75461	0.479212	0
ASP	12	1.46024	0.0721122	-0.660746	0.599305	0
PAP	1	0.78028	0.217613	-0.280289	0	0
ACP	4	-2.09485	0.981908	-2.22717	0.471369	0
ALA	2	-0.263004	0.603726	-1.30826	0.756902	0
CHCOA	1	0.316743	0.375719	-0.773051	0	0
AONA	1	0.316743	0.375719	-0.773051	0	0
DTB	1	-0.377166	0.646975	-1.51071	0	0
BT	1	-0.377166	0.646975	-1.51071	0	0
ETH	2	1.93352	0.0265859	0.345095	3.19871	1
ETHm	2	1.93352	0.0265859	0.345095	3.19871	1
ACALm	4	1.8482	0.0322865	-0.125623	2.06057	1
ACm	5	0.585531	0.279095	-0.831516	1.34593	0
AMPm	4	-0.314772	0.623532	-1.27844	1.03711	0
PPIm	4	-0.314772	0.623532	-1.27844	1.03711	0
ACTPm	1	-1.07354	0.858486	-2.25099	0	0
GLYN	3	-0.188604	0.574798	-1.22654	0.409956	0

GL	6	1.00832	0.156651	-0.671694	1.64687	1
GLYAL	3	1.61265	0.0534102	-0.118623	2.4002	1
O2	8	-1.84012	0.967125	-1.80537	0.544127	0
H2O2	4	-1.81885	0.965533	-2.08007	0.452237	0
GL3P	4	-0.70488	0.759558	-1.48635	0.338402	0
G	1	-0.166475	0.566109	-1.28674	0	0
E	1	0.0599283	0.476106	-1.04606	0	0
EOL	1	0.0599283	0.476106	-1.04606	0	0
LXUL	2	-0.391313	0.652217	-1.40484	0.198297	0
ARAB	1	0.0599283	0.476106	-1.04606	0	0
ARABLAC	1	0.0599283	0.476106	-1.04606	0	0
LAOL	2	-0.391313	0.652217	-1.40484	0.198297	0
RIB	3	0.848821	0.19799	-0.588438	0.36448	0
O2e	1	-1.2999	0.903183	-2.49162	0	0
GLCN15LACe	1	0.428033	0.334313	-0.654745	0	0
H2O2e	1	-1.2999	0.903183	-2.49162	0	0
H2Oe	5	-0.978815	0.836164	-1.57758	1.04416	0
GLCNTe	1	0.428033	0.334313	-0.654745	0	0
GLCN15LAC	1	0.428033	0.334313	-0.654745	0	0

GLCNT	1	0.428033	0.334313	-0.654745	0	0
GLAC	1	-0.324817	0.62734	-1.45506	0	0
GAL1P	1	-0.134703	0.553577	-1.25296	0	0
UTP	4	-0.119892	0.547716	-1.17457	1.20359	0
UDPGAL	3	-0.386064	0.650275	-1.34799	0.258875	0
UDPG	4	-0.981712	0.836879	-1.6339	0.609633	0
G1P	1	-0.134703	0.553577	-1.25296	0	0
GALN14LAC	1	-0.324817	0.62734	-1.45506	0	0
GALNT	1	-0.841392	0.799936	-2.00421	0	0
2D3DGALT	1	-0.841392	0.799936	-2.00421	0	0
SOR	1	-0.352049	0.637599	-1.48401	0	0
MAN6P	2	-0.127456	0.55071	-1.20623	0.0848507	0
MAN	2	-0.127456	0.55071	-1.20623	0.0848507	0
FRU	3	0.117718	0.453146	-1.03812	0.297291	0
MNT	1	0.38367	0.350612	-0.701906	0	0
MAN1P	1	-0.551415	0.709326	-1.69595	0	0
GDPMAN	1	-0.551415	0.709326	-1.69595	0	0
IDOL	1	-0.352049	0.637599	-1.48401	0	0
UDP	5	-0.645269	0.740624	-1.4185	1.19813	0

TRE	1	-0.977982	0.835958	-2.14941	0	0
MLT	1	-1.63593	0.949072	-2.84883	0	0
MLTe	1	-1.63593	0.949072	-2.84883	0	0
13GLUCAN	1	-1.2999	0.903183	-2.49162	0	0
GA6P	3	-0.797599	0.787448	-1.60112	0.686583	0
NAGA6P	1	-1.20513	0.885924	-2.39088	0	0
UDPNAG	1	-1.62611	0.948037	-2.8384	0	0
CHIT	2	-1.03102	0.848734	-1.88636	1.34639	0
NAG	1	0.165045	0.434454	-0.934314	0	0
GLCN	2	-0.127456	0.55071	-1.20623	0.0848507	0
13GLUCANe	4	-1.29781	0.902824	-1.80237	0.585262	0
STARe	1	-1.20513	0.885924	-2.39088	0	0
GLYCOGENe	1	-1.20513	0.885924	-2.39088	0	0
CELLUe	4	-0.516407	0.697215	-1.3859	0.733543	0
CELLOBe	4	-0.516407	0.697215	-1.3859	0.733543	0
CELLOTe	2	0.217812	0.413788	-0.946345	0.510855	0
MANNANe	1	-0.308847	0.621281	-1.43808	0	0
MANe	1	-0.308847	0.621281	-1.43808	0	0
PECTATEe	1	1.94792	0.0257121	0.960972	0	0

GALUNTe	1	1.94792	0.0257121	0.960972	0	0
ARABINe	3	-0.259283	0.602292	-1.27001	0.24481	0
LARABe	3	-0.259283	0.602292	-1.27001	0.24481	0
H+_PO_mit	1	-1.20513	0.885924	-2.39088	0	0
H+_PO	1	-1.20513	0.885924	-2.39088	0	0
FERIm	2	-1.14034	0.872928	-1.96864	0.964363	0
FEROm	2	-1.14034	0.872928	-1.96864	0.964363	0
LLACm	3	0.461821	0.322105	-0.826474	0.984278	0
GLUm	5	0.740479	0.229505	-0.757619	0.822735	0
ASPm	4	0.973781	0.165083	-0.591669	0.934773	0
ASN	2	0.200475	0.420554	-0.959395	0.225001	0
SAM	3	-0.256473	0.601207	-1.26828	1.25858	0
HCYS	4	-0.20195	0.580022	-1.2183	0.974566	0
SAH	3	-0.0595314	0.523736	-1.14715	1.19673	0
MET	2	0.185136	0.426561	-0.970941	1.61358	0
TRNAm	2	0.432705	0.332615	-0.784592	1.47858	0
ASPTRNAm	2	0.432705	0.332615	-0.784592	1.47858	0
TRNA	1	1.28939	0.0986308	0.260923	0	0
ASPTRNA	1	1.28939	0.0986308	0.260923	0	0

NH3	12	-1.20585	0.886063	-1.48291	0.961167	0
NAGLUm	1	-0.492412	0.688786	-1.63322	0	0
NAGLUSm	1	-0.377166	0.646975	-1.51071	0	0
NAORNm	2	-0.613344	0.730175	-1.57197	0.0866293	0
ORNm	1	-0.492412	0.688786	-1.63322	0	0
CAP	4	0.563123	0.286676	-0.810539	0.688491	0
ORN	3	5.34043	1.4893e-007	2.17425	3.20513	2
CITR	1	0.414397	0.339292	-0.669241	0	0
GLUGSAL	2	2.94195	0.00163076	1.10415	3.69798	1
ARGSUCC	2	0.25021	0.401213	-0.921959	0.357397	0
ARG	2	3.56069	0.000184941	1.56989	3.8814	1
PTRSC	1	-0.656125	0.744128	-1.80726	0	0
DSAM	1	-0.656125	0.744128	-1.80726	0	0
SPRMD	2	-0.728948	0.766983	-1.65898	0.20969	0
5MTA	1	-0.656125	0.744128	-1.80726	0	0
SPRM	1	-0.656125	0.744128	-1.80726	0	0
UREA	2	4.35616	2.14753e-005	2.16865	3.03463	1
DAPRP	1	-0.377166	0.646975	-1.51071	0	0
APS	1	-0.617761	0.731634	-1.76648	0	0

PAPS	1	-0.617761	0.731634	-1.76648	0	0
SER	4	0.338101	0.367644	-0.93047	0.747747	0
ASER	1	-0.240118	0.594881	-1.36502	0	0
RTHIO	2	0.0943776	0.462405	-1.03926	0.179266	0
OTHIO	2	0.0943776	0.462405	-1.03926	0.179266	0
GLUGSALm	1	1.18818	0.117381	0.153333	0	0
P5Cm	2	2.90204	0.00185368	1.07412	1.30218	1
PHP	1	-1.20513	0.885924	-2.39088	0	0
GLYm	1	1.41038	0.0792133	0.389541	0	0
GLY	5	-0.813339	0.791988	-1.49866	1.20218	0
GLX	2	-0.780731	0.78252	-1.69796	0.205782	0
BASP	2	-0.377234	0.647	-1.39424	0.164709	0
ASPSA	1	-0.158047	0.56279	-1.27778	0	0
HSER	2	-0.242505	0.595805	-1.29283	0.102093	0
PHSER	2	0.100287	0.460058	-1.03481	0.262809	0
THR	3	-0.663144	0.746381	-1.51842	0.721836	0
LLCT	2	-0.409669	0.658976	-1.41866	0.0973273	0
OBUT	3	-1.15053	0.875037	-1.81819	0.748671	0
THRm	2	-1.10127	0.864609	-1.93923	0.729927	0

NH3m	2	-1.10127	0.864609	-1.93923	0.729927	0
OBUTm	3	-0.928094	0.823321	-1.68138	0.68254	0
PRPP	7	1.17707	0.119583	-0.636136	0.404744	0
PRBATP	2	0.119585	0.452406	-1.02028	0.808436	0
PRBAMP	1	-0.453569	0.674931	-1.59193	0	0
PRFP	2	0.399268	0.344848	-0.80976	1.10616	0
PRLP	2	1.1197	0.131422	-0.267485	0.339264	0
DIMGP	2	0.962027	0.168018	-0.386164	0.171426	0
IMACP	2	0.911912	0.180907	-0.423886	0.224772	0
HISOLP	2	-0.038647	0.515414	-1.13939	0.787097	0
HISOL	2	-0.70896	0.760825	-1.64394	0.0735496	0
HIS	2	-0.378734	0.647557	-1.39537	0.277976	0
AICAR	3	0.115744	0.453928	-1.03934	0.504402	0
HTRNA	1	-0.083768	0.53338	-1.19881	0	0
HHTRNA	1	-0.083768	0.53338	-1.19881	0	0
OMVALm	1	-0.334583	0.63103	-1.46544	0	0
VAL	1	-0.663836	0.746602	-1.81546	0	0
OICAP	1	-0.128515	0.551129	-1.24638	0	0
ABUTm	2	-0.322023	0.626282	-1.35269	0.264474	0

ACLACm	2	-0.322023	0.626282	-1.35269	0.264474	0
DHVALm	2	-0.521148	0.698868	-1.50257	0.0525046	0
DHMVAm	2	-0.521148	0.698868	-1.50257	0.0525046	0
OIVALm	2	-0.501893	0.692129	-1.48808	0.0320079	0
IPPMALm	1	-0.377166	0.646975	-1.51071	0	0
CBHCAP	1	-1.25012	0.894372	-2.43871	0	0
IPPMAL	2	-0.972811	0.834676	-1.84254	0.843099	0
PPMAL	1	-1.25012	0.894372	-2.43871	0	0
HACNm	1	-0.158816	0.563093	-1.27859	0	0
HICITm	2	-0.138976	0.555265	-1.2149	0.0900709	0
OXAm	1	-0.0389908	0.515551	-1.15121	0	0
MICIT	1	-0.492412	0.688786	-1.63322	0	0
AKA	1	-0.147187	0.558508	-1.26623	0	0
AMA	3	0.237472	0.406145	-0.964466	0.264462	0
AMASA	2	0.574398	0.282849	-0.677938	0.134511	0
SACP	2	0.354201	0.361594	-0.843683	0.368911	0
LYS	2	-0.555707	0.710795	-1.52858	0.599685	0
LTRNA	1	-0.79287	0.786073	-1.95263	0	0
LLTRNA	1	-0.79287	0.786073	-1.95263	0	0

LYSm	1	-0.79287	0.786073	-1.95263	0	0
LTRNAm	1	-0.79287	0.786073	-1.95263	0	0
LLTRNAm	1	-0.79287	0.786073	-1.95263	0	0
ADN	4	0.177093	0.429718	-1.01628	0.608663	0
MTHPTGLU	1	-0.94271	0.827085	-2.11191	0	0
THPTGLU	1	-0.94271	0.827085	-2.11191	0	0
OAHSER	1	-0.240118	0.594881	-1.36502	0	0
OSLHSER	1	-1.47866	0.930384	-2.68165	0	0
3DDAH7P	2	-0.494377	0.68948	-1.48242	0.297876	0
DQT	1	-0.377166	0.646975	-1.51071	0	0
QT	1	-0.377166	0.646975	-1.51071	0	0
3PSME	1	1.2229	0.110683	0.190241	0	0
PHPYR	1	-0.492412	0.688786	-1.63322	0	0
PHE	1	-0.492412	0.688786	-1.63322	0	0
4HPP	2	-0.126042	0.55015	-1.20517	0.605361	0
TYR	2	-0.126042	0.55015	-1.20517	0.605361	0
AN	1	-1.05518	0.854328	-2.23147	0	0
NPRAN	1	-1.07354	0.858486	-2.25099	0	0
CPAD5P	1	-1.07354	0.858486	-2.25099	0	0

IGP	2	0.0939779	0.462563	-1.03956	1.71323	0
TRP	2	0.156007	0.438014	-0.992866	1.6472	0
FKYN	1	-0.985699	0.837859	-2.15761	0	0
AM6SA	1	-0.363608	0.641925	-1.4963	0	0
AMUCO	1	-0.363608	0.641925	-1.4963	0	0
GLUP	1	-1.20513	0.885924	-2.39088	0	0
PROm	1	2.92053	0.0017472	1.9949	0	1
GABALm	2	0.6828	0.247367	-0.596342	1.27273	0
GABAm	2	0.6828	0.247367	-0.596342	1.27273	0
LACALm	2	0.6828	0.247367	-0.596342	1.27273	0
GC	2	-1.7795	0.962421	-2.44975	0.283979	0
OGT	1	-1.20513	0.885924	-2.39088	0	0
cAMP	3	-1.38426	0.91686	-1.96196	0.529628	0
GMP	5	0.20474	0.418888	-1.01312	0.855143	0
DGMP	1	-0.377166	0.646975	-1.51071	0	0
DGDP	2	0.514329	0.303511	-0.723152	0.626304	0
DATP	1	0.78028	0.217613	-0.280289	0	0
DADP	3	1.55992	0.0593889	-0.151054	1.08537	0
AIR	1	0.685528	0.246505	-0.381015	0	0

CAIR	2	0.505946	0.306447	-0.729462	0.492779	0
SAICAR	2	0.0284027	0.488671	-1.08892	0.0155654	0
PRFICA	1	-0.377166	0.646975	-1.51071	0	0
IMP	7	0.545922	0.29256	-0.890701	0.771625	0
ASUC	2	0.659487	0.254792	-0.61389	0.687354	0
XMP	4	0.153264	0.439095	-1.02898	0.728422	0
cdAMP	1	-0.677618	0.750993	-1.83011	0	0
DAMP	3	0.533219	0.296941	-0.782558	1.54607	0
cIMP	1	-0.677618	0.750993	-1.83011	0	0
cGMP	1	-0.677618	0.750993	-1.83011	0	0
cCMP	1	-0.677618	0.750993	-1.83011	0	0
UGC	1	-0.416432	0.661453	-1.55245	0	0
CAASP	3	0.674016	0.25015	-0.695957	0.795144	0
DOROA	1	-0.768838	0.779005	-1.92708	0	0
OROA	2	-0.256761	0.601318	-1.30356	0.881783	0
OMP	2	0.348033	0.363908	-0.848326	0.237981	0
UMP	2	-0.203746	0.580724	-1.26366	0.349387	0
URA	3	-0.910617	0.818751	-1.67063	0.235131	0
CYTS	1	-0.424073	0.664244	-1.56058	0	0

URI	2	-0.720154	0.764285	-1.65237	0.200329	0
CYTD	2	-0.720154	0.764285	-1.65237	0.200329	0
DU	2	-0.720154	0.764285	-1.65237	0.200329	0
DT	1	-0.377166	0.646975	-1.51071	0	0
DC	2	-0.720154	0.764285	-1.65237	0.200329	0
DTMP	2	-0.750871	0.773635	-1.67549	0.233027	0
DTDP	2	0.0664534	0.473508	-1.06027	1.10307	0
OTHIOm	1	0.18557	0.426391	-0.912495	0	0
RTHIOm	1	0.18557	0.426391	-0.912495	0	0
DUTP	2	0.522382	0.300702	-0.717091	0.617732	0
DUMP	2	-0.294942	0.615981	-1.3323	0.252309	0
DCMP	1	-0.377166	0.646975	-1.51071	0	0
DCDP	2	0.514329	0.303511	-0.723152	0.626304	0
CDP	3	0.850347	0.197566	-0.587499	0.501332	0
PURI5P	2	-0.817526	0.793186	-1.72566	0.303981	0
AD	2	0.187025	0.425821	-0.969519	0.0768009	0
INS	2	0.341778	0.366259	-0.853034	0.930096	0
DA	1	-0.377166	0.646975	-1.51071	0	0
HYXN	1	1.05865	0.144881	0.0156269	0	0

DG	1	-0.377166	0.646975	-1.51071	0	0
GN	2	0.877494	0.190109	-0.449793	0.658203	0
GSN	3	0.384942	0.35014	-0.87376	0.658657	0
XAN	1	1.05865	0.144881	0.0156269	0	0
XTSINE	1	-0.377166	0.646975	-1.51071	0	0
ITP	2	1.94858	0.0256729	0.356427	0.900453	0
IDP	2	1.94858	0.0256729	0.356427	0.900453	0
DGTP	1	0.78028	0.217613	-0.280289	0	0
DUDP	2	0.514329	0.303511	-0.723152	0.626304	0
DCTP	1	0.78028	0.217613	-0.280289	0	0
DTTP	1	0.78028	0.217613	-0.280289	0	0
ACOA	1	-0.338487	0.632502	-1.46959	0	0
HACOA	1	-0.551415	0.709326	-1.69595	0	0
OACOA	2	-0.627695	0.734898	-1.58277	0.160056	0
AACCOA	2	1.77697	0.0377868	0.227254	2.3997	1
AACCOAm	1	-0.338487	0.632502	-1.46959	0	0
ACACPm	1	-0.083768	0.53338	-1.19881	0	0
MALACPm	1	-0.083768	0.53338	-1.19881	0	0
ACPm	1	-0.083768	0.53338	-1.19881	0	0

MALCOA	3	-0.755714	0.77509	-1.57535	0.770377	0
MALACP	2	-1.77721	0.962233	-2.44802	0.0205485	0
ACACP	2	-1.79652	0.963794	-2.46256	0.0411085	0
3OACPm	1	-0.083768	0.53338	-1.19881	0	0
C100ACP	1	-1.24522	0.893474	-2.43349	0	0
C120ACP	4	0.18563	0.426368	-1.01173	1.45523	0
C140ACP	4	0.18563	0.426368	-1.01173	1.45523	0
C141ACP	4	0.18563	0.426368	-1.01173	1.45523	0
C160ACP	4	0.18563	0.426368	-1.01173	1.45523	0
C161ACP	4	0.18563	0.426368	-1.01173	1.45523	0
C180ACP	4	0.18563	0.426368	-1.01173	1.45523	0
C181ACP	4	0.18563	0.426368	-1.01173	1.45523	0
C182ACP	4	0.18563	0.426368	-1.01173	1.45523	0
3HPACP	1	-1.2999	0.903183	-2.49162	0	0
2HDACP	1	-1.2999	0.903183	-2.49162	0	0
AACP	1	-1.2999	0.903183	-2.49162	0	0
23DAACP	1	-1.2999	0.903183	-2.49162	0	0
C150ACP	3	0.436622	0.331193	-0.841973	1.7331	0
C162ACP	4	0.18563	0.426368	-1.01173	1.45523	0

C170ACP	4	0.18563	0.426368	-1.01173	1.45523	0
C183ACP	4	0.18563	0.426368	-1.01173	1.45523	0
C200ACP	4	0.18563	0.426368	-1.01173	1.45523	0
AGL3P	1	-0.386859	0.65057	-1.52102	0	0
PA	2	-0.725247	0.76585	-1.6562	0.191178	0
CTPm	1	0.78028	0.217613	-0.280289	0	0
CDPDGm	1	-0.926452	0.822894	-2.09463	0	0
CDPDG	1	-0.166475	0.566109	-1.28674	0	0
PS	1	1.67838	0.046637	0.67443	0	0
CMPm	1	-0.926452	0.822894	-2.09463	0	0
PE	2	0.334881	0.368857	-0.858225	2.1675	0
PMME	1	-0.995109	0.840158	-2.16762	0	0
PDME	1	-0.995109	0.840158	-2.16762	0	0
PC	2	-1.55298	0.939786	-2.27925	0.157871	0
PCHO	1	-0.781564	0.782765	-1.94061	0	0
CDPCHO	2	-1.40219	0.919571	-2.16574	0.318392	0
DAGLY	5	0.287322	0.386933	-0.973737	1.29899	0
CDPETN	1	-1.20513	0.885924	-2.39088	0	0
MYOI	1	-0.166475	0.566109	-1.28674	0	0

PINS	3	-0.942549	0.827044	-1.69027	0.35283	0
PINSP	1	-0.781564	0.782765	-1.94061	0	0
PINS4P	1	-0.768838	0.779005	-1.92708	0	0
D45PI	2	-0.199599	0.579103	-1.26054	0.942632	0
TPI	1	0.485181	0.313774	-0.593994	0	0
GL3Pm	1	-0.926452	0.822894	-2.09463	0	0
PGPm	1	-0.926452	0.822894	-2.09463	0	0
CDPm	1	0.78028	0.217613	-0.280289	0	0
PSPH	1	-0.787945	0.784636	-1.94739	0	0
C260COA	1	-0.787945	0.784636	-1.94739	0	0
CER2	1	-0.787945	0.784636	-1.94739	0	0
CER3	1	-0.690191	0.754963	-1.84347	0	0
IPC	1	-0.690191	0.754963	-1.84347	0	0
MIPC	1	-0.690191	0.754963	-1.84347	0	0
MIP2C	1	-0.690191	0.754963	-1.84347	0	0
H3MCOA	2	2.63603	0.00419412	0.873882	1.48523	1
MVL	2	1.14789	0.125508	-0.246264	0.098894	0
PMVL	1	0.746506	0.227681	-0.316193	0	0
IPPP	1	-1.20513	0.885924	-2.39088	0	0

DMPP	1	-1.20513	0.885924	-2.39088	0	0
IGST	1	0.344954	0.365065	-0.743062	0	0
DMZYMST	3	-0.863703	0.806124	-1.64177	0.834034	0
IMZYMST	2	-1.30307	0.903724	-2.09113	0.423909	0
IIMZYMST	1	-0.166475	0.566109	-1.28674	0	0
MZYMST	3	-1.15834	0.876637	-1.823	0.55275	0
IZYMST	2	-1.30307	0.903724	-2.09113	0.423909	0
IIZYMST	1	-0.166475	0.566109	-1.28674	0	0
ZYMST	1	-0.166475	0.566109	-1.28674	0	0
FEST	1	-0.377166	0.646975	-1.51071	0	0
EPST	1	-0.377166	0.646975	-1.51071	0	0
ERTEOL	1	0.344954	0.365065	-0.743062	0	0
ERGOST	1	0.344954	0.365065	-0.743062	0	0
TAGLY	2	1.41366	0.078731	-0.0462139	1.48589	0
MAGLY	2	1.41366	0.078731	-0.0462139	1.48589	0
PHACAL	2	0.6828	0.247367	-0.596342	1.27273	0
PHAC	3	0.0263601	0.489485	-1.09432	1.24654	0
PHACCOA	2	-0.480786	0.684666	-1.47219	0.874091	0
IPN	1	0.240489	0.404976	-0.854114	0	0

PENG	1	0.240489	0.404976	-0.854114	0	0
NOR	1	0.0319577	0.487253	-1.07579	0	0
AVF	1	0.0111957	0.495534	-1.09786	0	0
VHA	1	0.0111957	0.495534	-1.09786	0	0
VERB	1	-0.663836	0.746602	-1.81546	0	0
HNO3	1	-0.768506	0.778907	-1.92672	0	0
UREAC	1	1.06543	0.14334	0.0228424	0	0
HNO3e	1	-0.768506	0.778907	-1.92672	0	0

#Results for Down-regulated only genes

#Feature	Number of neighbors	Z-score	P-value	Average Z	StdDev Z	Significance count
DGLCe	2	-0.466256	0.679484	-1.46125	0.775313	0
GLCe	16	1.89087	0.0293206	-0.605853	0.97856	1
bDGLCe	3	1.03285	0.150838	-0.475248	0.459306	0
DGLC	1	-0.519166	0.698178	-1.66166	0	0
GLC	17	5.25692	2.35423e-007	0.251813	1.54649	3
bDGLC	5	1.13222	0.12877	-0.570789	0.739544	0
ATP	43	-0.912139	0.819152	-1.26027	0.674088	0
ADP	28	-0.795077	0.786716	-1.27201	0.622143	0

G6P	8	-1.07192	0.858123	-1.51546	0.443002	0
bDG6P	3	-0.233194	0.592195	-1.25396	0.370515	0
H2O	49	2.88731	0.00194274	-0.669469	1.36083	3
PI	29	0.913934	0.180376	-0.929627	1.21976	1
F6P	10	0.237102	0.406289	-1.03093	0.646623	0
FDP	2	-0.248258	0.598033	-1.29716	0.626565	0
S7P	4	0.396399	0.345906	-0.899399	0.208581	0
S17P	2	-0.248258	0.598033	-1.29716	0.626565	0
T3P2	4	-0.638457	0.738412	-1.45095	0.263858	0
T3P1	5	0.341626	0.366316	-0.947838	0.556397	0
E4P	4	-0.0192385	0.507675	-1.12092	0.46157	0
NAD	38	-0.0295596	0.511791	-1.11641	1.18008	1
13PDG	3	0.225334	0.41086	-0.971932	0.664589	0
NADH	38	-0.0295596	0.511791	-1.11641	1.18008	1
3PG	2	-0.292082	0.614888	-1.33015	0.336803	0
2PG	2	-0.511848	0.695621	-1.49557	0.102863	0
23PDG	1	-0.431344	0.666891	-1.56831	0	0
PEP	2	-0.0472417	0.51884	-1.14585	0.39171	0
PYR	9	-1.32504	0.907421	-1.58253	0.460872	0

CO2	24	-1.4513	0.926651	-1.4281	0.650711	0
OA	4	-0.459743	0.67715	-1.3557	0.334313	0
ATPm	9	-1.02383	0.847042	-1.47534	0.501213	0
PYRm	4	-1.28925	0.901345	-1.79781	0.680609	0
CO2m	5	-0.360477	0.640755	-1.28268	0.525763	0
ADPm	5	-0.4662	0.679464	-1.3331	0.564375	0
PIm	7	-0.847195	0.801557	-1.45259	0.589459	0
OAm	4	-0.820061	0.793909	-1.54774	0.402036	0
GTP	3	-1.18433	0.881859	-1.83899	0.249486	0
GDP	1	-0.492412	0.688786	-1.63322	0	0
NADP	48	-0.284448	0.611967	-1.15529	0.812938	0
D6PGL	1	-0.317008	0.624381	-1.44676	0	0
NADPH	48	-0.284448	0.611967	-1.15529	0.812938	0
D6PGC	6	0.975492	0.164658	-0.68599	0.96147	0
RL5P	4	-1.11633	0.86786	-1.70565	0.81319	0
XUL5P	3	0.461827	0.322103	-0.82647	0.122426	0
R5P	7	-0.0136328	0.505439	-1.11639	0.310131	0
ACCOAm	3	-0.324585	0.627253	-1.31018	0.940074	0
H2Om	5	0.165918	0.434111	-1.03164	0.685883	0

CITm	1	-0.0415106	0.516556	-1.15389	0	0
COAm	3	-0.324585	0.627253	-1.31018	0.940074	0
ACOm	1	-0.0415106	0.516556	-1.15389	0	0
ICITm	2	-0.57073	0.715909	-1.53989	0.545885	0
NADm	11	0.043222	0.482762	-1.09711	0.734327	0
AKGm	3	-1.08298	0.860591	-1.77665	0.191607	0
NADHm	11	0.043222	0.482762	-1.09711	0.734327	0
ICIT	1	-0.767721	0.778674	-1.92589	0	0
AKG	7	-1.01302	0.844475	-1.51948	0.544371	0
NADPm	7	-0.646785	0.741114	-1.37176	0.758798	0
NADPHm	7	-0.646785	0.741114	-1.37176	0.758798	0
ICITg	1	-0.767721	0.778674	-1.92589	0	0
NADPg	1	-0.767721	0.778674	-1.92589	0	0
AKGg	1	-0.767721	0.778674	-1.92589	0	0
CO2g	1	-0.767721	0.778674	-1.92589	0	0
NADPHg	1	-0.767721	0.778674	-1.92589	0	0
DHLIPOm	1	-0.187125	0.574219	-1.30869	0	0
GDPm	1	0.275088	0.391624	-0.817333	0	0
GTPm	1	0.275088	0.391624	-0.817333	0	0

FADH2m	4	0.146419	0.441795	-1.03263	0.660643	0
FADm	4	0.146419	0.441795	-1.03263	0.660643	0
FUM	2	-0.826969	0.795873	-1.73277	1.29795	0
SUCC	3	-0.10314	0.541074	-1.17397	0.49914	0
MALm	2	-0.942967	0.827151	-1.82008	0.159949	0
MAL	1	-0.561793	0.712871	-1.70698	0	0
MALg	1	-0.618492	0.731874	-1.76725	0	0
NADg	1	-0.618492	0.731874	-1.76725	0	0
OAg	1	-0.618492	0.731874	-1.76725	0	0
NADHg	1	-0.618492	0.731874	-1.76725	0	0
Hm	4	-0.057391	0.522883	-1.14126	0.86827	0
CIT	1	-0.0519756	0.520726	-1.16502	0	0
COA	20	-0.348841	0.636396	-1.19458	0.741513	0
ACCOA	12	-0.513445	0.69618	-1.26939	0.534969	0
OXAL	2	-0.300779	0.618208	-1.3367	1.49084	0
AC	10	-0.0392806	0.515667	-1.12427	0.945532	0
FOR	6	-0.764278	0.777649	-1.44369	0.915279	0
FORm	1	-0.0343065	0.513684	-1.14623	0	0
METHOL	2	0.678221	0.248816	-0.599788	1.28824	0

FALD	5	0.540038	0.294586	-0.853212	0.749579	0
ADHLIPOm	1	-0.187125	0.574219	-1.30869	0	0
ACAL	9	-0.223419	0.588395	-1.19048	0.884749	0
RGT	4	-1.00023	0.8414	-1.64377	0.6948	0
FGT	1	-1.40346	0.91976	-2.60171	0	0
H+	19	1.31041	0.0950289	-0.789721	0.908972	0
MTHGXL	3	0.715935	0.237016	-0.670174	1.33344	0
LACAL	3	0.524451	0.299983	-0.787951	1.41132	0
LAC	2	-0.664566	0.746836	-1.61052	0.141155	0
LGT	2	-0.429859	0.666351	-1.43386	0.391	0
LLAC	2	-1.16669	0.878331	-1.98848	0.312858	0
PROP	1	-1.07354	0.858486	-2.25099	0	0
AMP	16	-0.227139	0.589842	-1.17182	0.864372	0
PPI	24	-1.08277	0.860545	-1.34763	0.731584	0
PROPCOA	1	-1.07354	0.858486	-2.25099	0	0
GLU	13	-0.971539	0.83436	-1.39897	0.565846	0
GABA	3	-0.304664	0.619689	-1.29792	0.839144	0
SUCCSAL	4	0.102662	0.459116	-1.05595	0.470961	0
METTHF	2	0.0366234	0.485393	-1.08273	0.60526	0

METTHFm	1	0.428033	0.334313	-0.654745	0	0
THFm	1	0.428033	0.334313	-0.654745	0	0
AHTD	1	1.97584	0.0240866	0.990647	0	0
DHP	2	1.68934	0.0455775	0.161292	1.17288	0
AHHMP	1	0.415506	0.338886	-0.668062	0	0
GLAL	1	0.415506	0.338886	-0.668062	0	0
CHOR	1	-0.872974	0.808661	-2.03778	0	0
GLN	4	-0.36204	0.641339	-1.30363	0.617065	0
PABA	1	0.415506	0.338886	-0.668062	0	0
AHHMD	1	0.415506	0.338886	-0.668062	0	0
DHPT	1	0.415506	0.338886	-0.668062	0	0
DHF	1	-0.377166	0.646975	-1.51071	0	0
THF	2	0.32955	0.37087	-0.862238	0.29344	0
THFG	1	0.0376589	0.48498	-1.06973	0	0
CTP	3	-0.487946	0.687206	-1.41066	0.429186	0
CYS	4	-0.0666819	0.526583	-1.14621	0.309188	0
CMP	4	0.223272	0.411662	-0.991671	0.814493	0
ASP	6	-0.886891	0.812431	-1.49709	0.242957	0
PAP	2	-1.03119	0.848774	-1.88649	0.335694	0

ACP	6	0.146442	0.441786	-1.04706	1.01566	0
ALA	4	-1.07504	0.858822	-1.68364	0.905897	0
ETH	2	0.678221	0.248816	-0.599788	1.28824	0
ETHm	2	0.678221	0.248816	-0.599788	1.28824	0
ACALm	5	0.627446	0.265183	-0.811526	0.954459	0
ACm	4	-0.312568	0.622696	-1.27726	1.01639	0
AMPm	5	-0.786722	0.784278	-1.48597	0.516451	0
PPIm	7	-0.992355	0.839488	-1.51114	0.435214	0
GLYN	8	-0.0372188	0.514845	-1.12498	0.954372	0
GL	8	0.46618	0.320543	-0.935006	1.16213	0
GLYAL	6	0.232967	0.407893	-1.00937	1.27421	0
O2	26	2.09488	0.0180909	-0.671671	1.51712	2
H2O2	6	3.63447	0.000139274	0.472048	2.66189	2
GL3P	3	0.233897	0.407533	-0.966665	0.892322	0
TAR	2	-1.13389	0.871579	-1.96379	0.412677	0
OXGLY	2	-1.13389	0.871579	-1.96379	0.412677	0
E	5	0.143132	0.443093	-1.0425	1.11506	0
EOL	5	0.143132	0.443093	-1.0425	1.11506	0
LXUL	4	-1.1161	0.867809	-1.70552	0.82396	0

XOL	6	0.168547	0.433077	-1.03743	1.00532	0
XUL	7	0.222437	0.411987	-1.02117	0.918732	0
AOL	6	0.168547	0.433077	-1.03743	1.00532	0
XYL	3	0.833612	0.20225	-0.597793	1.11427	0
ARAB	1	0.155956	0.438034	-0.943976	0	0
ARABLAC	1	0.155956	0.438034	-0.943976	0	0
LAOL	1	-1.20513	0.885924	-2.39088	0	0
R1P	2	-0.0867804	0.534577	-1.17562	0.224878	0
RL	2	-1.54375	0.938676	-2.2723	0.815342	0
O2e	6	4.30619	2.69653e-005	0.764594	2.38787	2
GLCN15LACe	2	0.595142	0.275874	-0.662323	0.460371	0
H2O2e	6	4.30619	2.69653e-005	0.764594	2.38787	2
H2Oe	14	4.11982	6.17289e-005	0.0654875	1.88875	3
GLCNT	5	1.13586	0.128008	-0.569055	1.02615	0
GLAC	17	5.4899	6.45169e-008	0.312217	1.97751	4
GALOL	5	0.143132	0.443093	-1.0425	1.11506	0
GAL1P	1	-0.216908	0.58586	-1.34035	0	0
UTP	3	-0.60772	0.728313	-1.48433	0.938628	0
UDPGAL	1	0.299079	0.38244	-0.79183	0	0

UDPG	6	0.559821	0.287801	-0.867023	1.03435	0
G1P	3	1.26754	0.102481	-0.330892	1.06065	0
MELI	4	6.40289	2.43299e-010	2.30191	1.10996	2
GALN14LAC	2	2.23377	0.012749	0.571097	4.63741	1
GALNT	1	-0.598549	0.725263	-1.74605	0	0
2D3DGALT	1	-0.598549	0.725263	-1.74605	0	0
SOR	7	1.15296	0.124464	-0.645863	0.979181	0
SOT	4	0.0584536	0.476694	-1.07952	1.04992	0
MAN6P	3	-0.538191	0.704777	-1.44156	0.0977246	0
MAN	2	0.394074	0.346763	-0.81367	1.00773	0
FRU	5	-0.211658	0.583813	-1.21171	0.859371	0
MNT6P	1	1.59411	0.0554558	0.584851	0	0
MNT	3	-0.595921	0.724386	-1.47707	0.839774	0
F26P	1	-0.431344	0.666891	-1.56831	0	0
GDPMAN	1	-1.20513	0.885924	-2.39088	0	0
IDOL	2	1.40738	0.0796576	-0.0509413	0.830004	0
UDP	12	-0.263729	0.604006	-1.19238	0.859204	0
TRE6P	3	-0.514801	0.696654	-1.42717	0.288226	0
TRE	2	-0.374562	0.646007	-1.39223	0.381034	0

MLT	4	0.355543	0.361091	-0.921174	0.871718	0
MLTe	4	0.355543	0.361091	-0.921174	0.871718	0
LACT	4	2.83995	0.00225601	0.402954	1.19903	1
LACTe	4	2.83995	0.00225601	0.402954	1.19903	1
GLACe	5	2.96259	0.00152531	0.302144	1.06257	1
13GLUCAN	1	-0.617761	0.731634	-1.76648	0	0
GA6P	1	-0.391774	0.652387	-1.52624	0	0
NAGA6P	1	-0.727475	0.766532	-1.88311	0	0
NAGA1P	2	-1.26971	0.897906	-2.06603	0.258687	0
UDPNAG	8	-0.739873	0.770311	-1.39015	0.716905	0
CHIT	11	1.01158	0.155869	-0.785263	0.989658	0
NAG	4	2.19384	0.0141235	0.0585915	0.924384	0
GLCN	1	-0.391774	0.652387	-1.52624	0	0
13GLUCANe	8	-0.130996	0.552111	-1.16037	0.525682	0
GLYCOGEN	2	2.62449	0.00433893	0.865197	0.122281	0
STARe	1	0.198553	0.421306	-0.898693	0	0
GLYCOGENe	1	0.198553	0.421306	-0.898693	0	0
AMYLSe	1	1.76667	0.0386415	0.768295	0	0
AMYLPe	1	1.76667	0.0386415	0.768295	0	0

CELLUe	4	-0.535587	0.703878	-1.39612	0.57698	0
CELLOBe	4	-0.535587	0.703878	-1.39612	0.57698	0
CELLOTe	2	-0.466256	0.679484	-1.46125	0.775313	0
MANNANe	5	0.819566	0.206232	-0.719901	1.50383	0
MANe	6	1.13444	0.128306	-0.616767	1.36859	0
ARABINe	2	1.4998	0.0668334	0.0186241	0.928384	0
LARABe	2	1.4998	0.0668334	0.0186241	0.928384	0
XYLANe	6	2.19379	0.0141253	-0.155398	1.11521	1
XYLe	6	2.19379	0.0141253	-0.155398	1.11521	1
H+_PO_mit	1	-0.0343065	0.513684	-1.14623	0	0
H+_PO	1	-0.0343065	0.513684	-1.14623	0	0
FERIm	1	-1.4494	0.926388	-2.65055	0	0
FEROm	1	-1.4494	0.926388	-2.65055	0	0
Ca	1	-0.0343065	0.513684	-1.14623	0	0
Cam	1	-0.0343065	0.513684	-1.14623	0	0
LLACm	3	0.256627	0.398733	-0.952684	0.957852	0
LACm	1	-1.4494	0.926388	-2.65055	0	0
GLUm	4	-0.718049	0.763637	-1.49337	0.267897	0
ASPm	2	-0.431113	0.666807	-1.4348	0.177873	0

ALAm	1	-0.690191	0.754963	-1.84347	0	0
ASN	3	-0.749049	0.773086	-1.57125	0.338865	0
SAM	8	-0.961261	0.831789	-1.4737	0.587118	0
HCYS	4	-0.745374	0.771977	-1.50794	0.65108	0
SAH	4	-0.728268	0.766775	-1.49882	0.0951088	0
MET	2	-1.70129	0.955556	-2.39088	0.000687845	0
TRNAm	2	-0.408306	0.658475	-1.41763	0.153595	0
ASPTRNAm	1	-0.187441	0.574343	-1.30902	0	0
TRNA	2	-0.32392	0.627001	-1.35411	0.0637662	0
ASPTRNA	2	-0.32392	0.627001	-1.35411	0.0637662	0
NH3	17	0.218288	0.413602	-1.05454	0.89336	0
NAGLUm	1	-1.07162	0.858054	-2.24895	0	0
NAGLUPm	1	-1.07162	0.858054	-2.24895	0	0
NAGLUSm	1	-1.07162	0.858054	-2.24895	0	0
CAP	1	4.54239	9.00259e-006	3.71902	0	1
ORN	2	2.94195	0.00163076	1.10415	3.69798	1
CITR	1	4.54239	9.00259e-006	3.71902	0	1
GLUGSAL	1	-0.360236	0.640665	-1.49271	0	0
ARG	1	-0.617761	0.731634	-1.76648	0	0

PTRSC	1	-0.377166	0.646975	-1.51071	0	0
DSAM	1	-0.768506	0.778907	-1.92672	0	0
SPRMD	1	0.357133	0.360496	-0.730115	0	0
SPRM	1	0.357133	0.360496	-0.730115	0	0
GBAD	3	0.79717	0.212676	-0.620207	1.02559	0
GBAT	3	0.79717	0.212676	-0.620207	1.02559	0
UREA	1	-0.56495	0.713946	-1.71033	0	0
ATRNA	1	-0.617761	0.731634	-1.76648	0	0
ALTRNA	1	-0.617761	0.731634	-1.76648	0	0
SLF	1	-0.28048	0.610445	-1.40793	0	0
APS	1	-0.28048	0.610445	-1.40793	0	0
PAPS	1	-0.953948	0.829945	-2.12386	0	0
SER	7	-0.860427	0.805223	-1.45793	0.481646	0
ASER	4	-0.0964403	0.538415	-1.16207	0.296732	0
H2S	5	-1.10853	0.866184	-1.63944	0.635923	0
RTHIO	2	-1.10966	0.866428	-1.94556	0.252159	0
OTHIO	2	-1.10966	0.866428	-1.94556	0.252159	0
H2SO3	2	-1.37418	0.915308	-2.14466	0.0294182	0
GLUGSALm	2	-0.231728	0.591626	-1.28472	0.0338969	0

P5Cm	2	-0.231728	0.591626	-1.28472	0.0338969	0
GLYm	1	0.428033	0.334313	-0.654745	0	0
GLY	6	0.0726374	0.471047	-1.0792	1.11343	0
ASPSA	1	-0.551415	0.709326	-1.69595	0	0
HSER	1	-0.551415	0.709326	-1.69595	0	0
THR	5	0.304441	0.380396	-0.965572	1.02558	0
LLCT	3	-0.167606	0.566553	-1.21362	0.340779	0
OBUT	1	-0.332069	0.630082	-1.46277	0	0
THRm	1	-0.594944	0.724059	-1.74222	0	0
NH3m	1	-0.594944	0.724059	-1.74222	0	0
OBUTm	1	-0.594944	0.724059	-1.74222	0	0
PRPP	2	-0.865796	0.806699	-1.76199	0.355364	0
PRBATP	1	-0.30955	0.621549	-1.43883	0	0
PRBAMP	1	-0.30955	0.621549	-1.43883	0	0
PRFP	1	-0.30955	0.621549	-1.43883	0	0
HISOL	1	-0.30955	0.621549	-1.43883	0	0
HIS	1	-0.30955	0.621549	-1.43883	0	0
AICAR	1	-1.4494	0.926388	-2.65055	0	0
VAL	2	0.120175	0.452172	-1.01984	0.107285	0

OICAP	1	-0.515297	0.696827	-1.65755	0	0
IPPMAL	1	-0.515297	0.696827	-1.65755	0	0
HCITm	1	-1.20513	0.885924	-2.39088	0	0
HACNm	1	-1.20513	0.885924	-2.39088	0	0
AMA	1	0.155956	0.438034	-0.943976	0	0
LYS	1	-0.391774	0.652387	-1.52624	0	0
LTRNA	1	-0.391774	0.652387	-1.52624	0	0
LLTRNA	1	-0.391774	0.652387	-1.52624	0	0
LYSm	1	-0.391774	0.652387	-1.52624	0	0
LTRNAm	1	-0.391774	0.652387	-1.52624	0	0
LLTRNAm	1	-0.391774	0.652387	-1.52624	0	0
ADN	3	0.596535	0.275409	-0.743614	0.814174	0
OAHSER	1	-1.20513	0.885924	-2.39088	0	0
METH	1	-1.20513	0.885924	-2.39088	0	0
CALH	1	-0.453569	0.674931	-1.59193	0	0
DPTH	1	-0.453569	0.674931	-1.59193	0	0
DQT	1	0.357133	0.360496	-0.730115	0	0
DHSK	1	0.357133	0.360496	-0.730115	0	0
PHEN	2	-1.46674	0.928776	-2.21433	0.249679	0

PHPYR	1	-0.195439	0.577476	-1.31753	0	0
4HPP	4	-0.595564	0.724267	-1.42809	0.722819	0
TYR	2	-0.217772	0.586196	-1.27421	0.404975	0
AN	3	-0.917604	0.820587	-1.67493	1.11947	0
NPRAN	1	-0.84992	0.802315	-2.01327	0	0
TRP	1	0.63164	0.263811	-0.438301	0	0
FKYN	2	0.69892	0.242301	-0.584208	0.206343	0
KYN	4	-0.726063	0.7661	-1.49764	1.07833	0
HKYN	3	-1.04532	0.852063	-1.75349	1.16253	0
HAN	2	-0.525381	0.700341	-1.50576	1.52798	0
CMUSA	1	0.286517	0.387241	-0.805183	0	0
AM6SA	1	0.286517	0.387241	-0.805183	0	0
HOMOGEN	2	0.526623	0.299228	-0.713898	0.0836553	0
MACAC	2	0.714507	0.237457	-0.572476	0.116346	0
FUACAC	2	0.608069	0.271571	-0.652593	0.229649	0
ACTAC	1	0.277302	0.390774	-0.81498	0	0
TRPm	1	-0.391774	0.652387	-1.52624	0	0
TRPTRNAm	1	-0.391774	0.652387	-1.52624	0	0
PAD	3	1.7852	0.0371147	-0.0124936	0.643446	0

PAC	3	1.7852	0.0371147	-0.0124936	0.643446	0
IAD	3	1.7852	0.0371147	-0.0124936	0.643446	0
IAC	3	1.7852	0.0371147	-0.0124936	0.643446	0
ASPERMD	2	0.458461	0.323311	-0.765205	0.0496249	0
APRUT	1	0.291116	0.385481	-0.800295	0	0
APROA	1	0.291116	0.385481	-0.800295	0	0
GABAL	1	0.291116	0.385481	-0.800295	0	0
ASPRM	2	0.458461	0.323311	-0.765205	0.0496249	0
GLUP	1	-0.360236	0.640665	-1.49271	0	0
P5C	1	-0.551415	0.709326	-1.69595	0	0
PRO	1	-0.551415	0.709326	-1.69595	0	0
PHC	1	-0.551415	0.709326	-1.69595	0	0
HPRO	1	-0.551415	0.709326	-1.69595	0	0
GABALm	3	0.256627	0.398733	-0.952684	0.957852	0
GABAm	3	0.256627	0.398733	-0.952684	0.957852	0
LACALm	3	0.256627	0.398733	-0.952684	0.957852	0
APROP	1	-0.724153	0.765514	-1.87958	0	0
TCOA	1	-1.05169	0.85353	-2.22777	0	0
GLP	1	-1.05169	0.85353	-2.22777	0	0

TGLP	1	-1.05169	0.85353	-2.22777	0	0
PEPD	1	-0.128632	0.551176	-1.24651	0	0
APEP	1	-0.128632	0.551176	-1.24651	0	0
OGT	1	0.00387987	0.498452	-1.10564	0	0
GMP	1	1.20572	0.113963	0.171974	0	0
DGMP	1	1.20572	0.113963	0.171974	0	0
DATP	1	-0.618492	0.731874	-1.76725	0	0
DADP	1	0.275088	0.391624	-0.817333	0	0
PRAM	1	-1.2658	0.897207	-2.45537	0	0
GAR	1	-1.2658	0.897207	-2.45537	0	0
FGAR	1	-0.377166	0.646975	-1.51071	0	0
FGAM	2	-1.15946	0.876867	-1.98304	0.667974	0
AIR	1	-1.2658	0.897207	-2.45537	0	0
SAICAR	1	-1.4494	0.926388	-2.65055	0	0
IMP	2	0.518292	0.302127	-0.720169	1.26168	0
ASUC	1	-1.4494	0.926388	-2.65055	0	0
XMP	1	1.20572	0.113963	0.171974	0	0
DAMP	2	1.04637	0.147696	-0.32268	0.699546	0
ATN	1	-1.02037	0.846223	-2.19446	0	0

ATT	2	-1.11876	0.868378	-1.9524	0.342331	0
UGC	1	-0.56495	0.713946	-1.71033	0	0
UMP	4	0.00372773	0.498513	-1.10868	0.856783	0
URA	3	-0.248068	0.597959	-1.26311	0.247056	0
CYTS	1	0.0631217	0.474835	-1.04266	0	0
URI	2	0.504402	0.30699	-0.730625	1.27647	0
CYTD	2	0.504402	0.30699	-0.730625	1.27647	0
DU	2	0.913999	0.180359	-0.422315	0.840451	0
DR1P	1	0.0876363	0.465083	-1.0166	0	0
DT	2	0.913999	0.180359	-0.422315	0.840451	0
THY	1	0.0876363	0.465083	-1.0166	0	0
DC	1	1.20572	0.113963	0.171974	0	0
DTMP	2	0.585782	0.279011	-0.669369	1.18984	0
DUTP	1	-0.618492	0.731874	-1.76725	0	0
DUMP	3	0.28727	0.386953	-0.933836	0.95796	0
DCMP	2	0.617627	0.268411	-0.645399	1.15594	0
DCDP	1	-0.332069	0.630082	-1.46277	0	0
CDP	1	-0.332069	0.630082	-1.46277	0	0
PURI5P	1	-0.143224	0.556943	-1.26202	0	0

AD	3	-0.370434	0.644471	-1.33838	0.300722	0
INS	3	0.596535	0.275409	-0.743614	0.814174	0
DA	3	0.596535	0.275409	-0.743614	0.814174	0
DIN	2	-0.121046	0.548173	-1.20141	0.261353	0
HYXN	2	-0.121046	0.548173	-1.20141	0.261353	0
DG	3	0.200776	0.420437	-0.987036	1.14451	0
GN	1	0.0876363	0.465083	-1.0166	0	0
GSN	3	0.200776	0.420437	-0.987036	1.14451	0
XAN	1	0.0876363	0.465083	-1.0166	0	0
XTSINE	2	0.913999	0.180359	-0.422315	0.840451	0
ITPm	1	0.275088	0.391624	-0.817333	0	0
IDPm	1	0.275088	0.391624	-0.817333	0	0
DGTP	2	-1.10476	0.865369	-1.94187	0.246941	0
DUDP	1	-0.332069	0.630082	-1.46277	0	0
DCTP	1	-0.618492	0.731874	-1.76725	0	0
LCCA	4	1.46709	0.0711756	-0.328746	0.706237	0
ACOA	6	1.01427	0.155226	-0.669101	0.760933	0
HACOA	2	0.0266183	0.489382	-1.09026	0.104949	0
OACOA	4	-0.205196	0.581291	-1.22003	0.170366	0

AACCOA	1	0.695094	0.243498	-0.370846	0	0
AACCOAm	1	0.695094	0.243498	-0.370846	0	0
MALCOA	4	-0.0439307	0.51752	-1.13408	0.290858	0
MALACP	2	0.021096	0.491585	-1.09442	0.424432	0
ACACP	2	-0.793159	0.786157	-1.70732	0.717974	0
C120ACP	5	-0.132473	0.552695	-1.17394	1.23478	0
C140ACP	5	-0.132473	0.552695	-1.17394	1.23478	0
C141ACP	5	-0.132473	0.552695	-1.17394	1.23478	0
C160ACP	5	-0.132473	0.552695	-1.17394	1.23478	0
C161ACP	5	-0.132473	0.552695	-1.17394	1.23478	0
C180ACP	5	-0.132473	0.552695	-1.17394	1.23478	0
C181ACP	5	-0.132473	0.552695	-1.17394	1.23478	0
C182ACP	5	-0.132473	0.552695	-1.17394	1.23478	0
3HPACP	2	-0.793159	0.786157	-1.70732	0.717974	0
2HDACP	2	-0.793159	0.786157	-1.70732	0.717974	0
AACP	2	-0.793159	0.786157	-1.70732	0.717974	0
23DAACP	2	-0.793159	0.786157	-1.70732	0.717974	0
C150ACP	3	0.152162	0.43953	-1.01694	1.71828	0
C162ACP	5	-0.132473	0.552695	-1.17394	1.23478	0

C170ACP	5	-0.132473	0.552695	-1.17394	1.23478	0
C183ACP	5	-0.132473	0.552695	-1.17394	1.23478	0
C200ACP	5	-0.132473	0.552695	-1.17394	1.23478	0
AGL3P	2	0.696154	0.243166	-0.58629	1.25851	0
AT3P2	2	0.696154	0.243166	-0.58629	1.25851	0
PA	3	0.368141	0.356384	-0.884094	0.136011	0
PAm	1	0.165045	0.434454	-0.934314	0	0
CTPm	1	0.165045	0.434454	-0.934314	0	0
CDPDGm	2	0.272375	0.392667	-0.905275	0.0410677	0
CDPDG	1	0.165045	0.434454	-0.934314	0	0
PS	2	0.739984	0.229655	-0.553298	0.680566	0
CMPm	2	-0.0786596	0.531348	-1.1695	0.414743	0
PSm	1	0.438069	0.330668	-0.644076	0	0
PE	4	0.294148	0.384322	-0.953896	0.607004	0
PEm	1	0.438069	0.330668	-0.644076	0	0
PMME	1	-0.241298	0.595338	-1.36628	0	0
PC	1	-0.21913	0.586725	-1.34271	0	0
CHO	1	-0.551415	0.709326	-1.69595	0	0
PCHO	1	-0.551415	0.709326	-1.69595	0	0

DAGLY	6	0.311785	0.377602	-0.975048	1.09377	0
PETHM	2	0.127405	0.44931	-1.0144	0.729739	0
CDPETN	1	-0.395686	0.653832	-1.5304	0	0
MI1P	2	-1.6332	0.948786	-2.33962	0.116076	0
MYOI	1	-1.07971	0.859864	-2.25755	0	0
PINS	4	0.785115	0.216193	-0.692223	0.420127	0
PINSP	3	0.59698	0.27526	-0.74334	0.499082	0
PINS4P	2	0.693408	0.244027	-0.588357	0.0699837	0
D45PI	1	0.0265872	0.489394	-1.0815	0	0
TPI	1	0.0265872	0.489394	-1.0815	0	0
PGPm	1	-0.884146	0.811691	-2.04966	0	0
PGm	2	-0.468505	0.680288	-1.46295	0.829734	0
CLm	1	0.219679	0.41306	-0.876235	0	0
DGPP	2	0.333872	0.369238	-0.858985	0.182249	0
LPC	1	-0.21913	0.586725	-1.34271	0	0
LPE	1	-0.21913	0.586725	-1.34271	0	0
CDPm	1	-0.332069	0.630082	-1.46277	0	0
PALCOA	2	-1.24474	0.893387	-2.04723	0.28816	0
DHSPH	3	-1.15386	0.875722	-1.82025	0.442814	0

SPH	4	0.120432	0.45207	-1.04648	0.22775	0
PSPH	2	0.15475	0.438509	-0.993813	0.0424443	0
CER2	1	-0.377166	0.646975	-1.51071	0	0
CER3	1	-0.377166	0.646975	-1.51071	0	0
IPC	1	-1.20513	0.885924	-2.39088	0	0
MIPC	1	-1.20513	0.885924	-2.39088	0	0
DHSP	3	0.562189	0.286994	-0.76474	0.23989	0
PHSP	1	0.137308	0.445394	-0.9638	0	0
C16A	1	0.575113	0.282607	-0.498392	0	0
H3MCOA	1	-0.0664624	0.526495	-1.18042	0	0
MVL	1	-0.0664624	0.526495	-1.18042	0	0
PMVL	1	-1.13948	0.872749	-2.32109	0	0
PPMVL	2	-1.09308	0.862821	-1.93307	0.548736	0
IPPP	2	-1.13944	0.872741	-1.96797	0.598086	0
DMPP	1	-1.20513	0.885924	-2.39088	0	0
GPP	1	-1.20513	0.885924	-2.39088	0	0
FPP	1	-1.20513	0.885924	-2.39088	0	0
S23E	1	0.128042	0.449058	-0.973651	0	0
LNST	4	0.387438	0.349216	-0.904175	0.588766	0

IGST	4	0.226138	0.410547	-0.990144	0.626207	0
DMZYMST	2	-0.436761	0.668858	-1.43905	0.171861	0
IMZYMST	2	-0.565086	0.713992	-1.53564	0.0352584	0
IIMZYMST	1	-0.377166	0.646975	-1.51071	0	0
MZYMST	1	-0.424073	0.664244	-1.56058	0	0
IZYMST	2	-0.565086	0.713992	-1.53564	0.0352584	0
IIZYMST	1	-0.377166	0.646975	-1.51071	0	0
ZYMST	1	-0.377166	0.646975	-1.51071	0	0
FEST	1	-0.377166	0.646975	-1.51071	0	0
EPST	2	-0.431475	0.666939	-1.43507	0.226452	0
ERTROL	3	-0.451898	0.674329	-1.38848	0.17931	0
ERTEOL	1	-0.174533	0.569277	-1.2953	0	0
TAGLY	3	0.152162	0.43953	-1.01694	1.71828	0
MAGLY	2	-1.08111	0.860177	-1.92406	0.983703	0
PHACAL	4	0.125287	0.450148	-1.04389	0.803076	0
PHAC	3	0.256627	0.398733	-0.952684	0.957852	0
LLDACV	2	-0.236043	0.5933	-1.28797	0.486478	0
IPN	1	-0.491223	0.688366	-1.63196	0	0
NOR	3	-0.150087	0.559652	-1.20285	0.351753	0

AVN	5	0.0307915	0.487718	-1.09608	0.534458	0
HAVN	4	0.39051	0.34808	-0.902538	0.655903	0
AVF	1	0.685528	0.246505	-0.381015	0	0
VHA	1	0.013231	0.494722	-1.0957	0	0
VERB	2	-0.521148	0.698868	-1.50257	0.0525046	0
VERA	2	-0.521148	0.698868	-1.50257	0.0525046	0
DMST	2	-0.521148	0.698868	-1.50257	0.0525046	0
DHDMST	2	-0.521148	0.698868	-1.50257	0.0525046	0
ST	1	-0.39188	0.652427	-1.52635	0	0
DHST	1	-0.39188	0.652427	-1.52635	0	0
OMST	3	1.48844	0.0683174	-0.195022	1.50996	0
DHOMST	3	1.48844	0.0683174	-0.195022	1.50996	0
AFB1	2	2.10032	0.0178505	0.470644	1.37888	0
AFG1	2	2.10032	0.0178505	0.470644	1.37888	0
AFB2	2	2.10032	0.0178505	0.470644	1.37888	0
AFG2	2	2.10032	0.0178505	0.470644	1.37888	0
HNO3	2	0.80411	0.210667	-0.50503	1.1055	0
HNO2	2	0.80411	0.210667	-0.50503	1.1055	0
NH4OH	1	-0.166475	0.566109	-1.28674	0	0

ACNL	1	-0.724153	0.765514	-1.87958	0	0
INAC	1	-0.724153	0.765514	-1.87958	0	0
NH3e	2	0.849099	0.197913	-0.471166	1.94073	0
HNO3e	1	-0.166475	0.566109	-1.28674	0	0
FRUe	1	0.948843	0.17135	-0.101099	0	0
SORe	1	0.948843	0.17135	-0.101099	0	0
