# Supplementary material for: Systems Analysis Unfolds the Relationship between the Phosphoketolase Pathway and Growth in Aspergillus nidulans
Source: PLoS One. 2008 Dec 4;3(12):e3847. doi: 10.1371/journal.pone.0003847 (PMC2585806; doi:10.1371/journal.pone.0003847)
Supplement: Table S6 — Results of reporter feature algorithm for the examination of phosphoketolase over-expression on glycerol. (0.35 MB PDF) [file pone.0003847.s006.pdf]

#Total number of ORFs used = 576

#Total number of significantly changed ORFs = 0

#Mean_all = -0.5325 #Std_all= 1.14592

#kmax, imax = 100, 10000

#Feature	Number of neighbors	Z-score	P-value	Average Z	StdDev Z	Significance count
DGLCe	4	-1.42533	0.92297	-1.34639	0.719473	0
GLCe	19	0.977436	0.164177	-0.275767	1.34175	2
bDGLCe	3	0.979543	0.163656	0.113925	2.39426	1
DGLC	2	-1.36044	0.913155	-1.63041	0.143498	0
GLC	20	-0.19723	0.578176	-0.582795	1.19725	1
bDGLC	7	-0.245012	0.596777	-0.638078	1.57391	1
ATP	89	0.630183	0.264287	-0.455887	1.13854	4
ADP	59	-0.0311608	0.512429	-0.537049	1.01749	2
G6P	10	-2.09391	0.981866	-1.28943	0.679253	0
bDG6P	5	-1.40568	0.920091	-1.25057	0.72318	0
H2O	73	0.995016	0.159864	-0.399008	1.41998	6
PI	58	2.02115	0.0216321	-0.228455	1.30625	4
F6P	14	-1.74882	0.959839	-1.06692	0.800814	0
FDP	3	-0.742269	0.771038	-1.02159	0.525949	0

S7P	6	-1.06979	0.857642	-1.03137	0.354152	0
S17P	3	-0.742269	0.771038	-1.02159	0.525949	0
T3P2	7	-0.666998	0.747613	-0.820402	0.676594	0
T3P1	11	0.766842	0.221588	-0.267865	1.62811	1
E4P	7	0.709755	0.238928	-0.225561	1.12458	0
NAD	72	-1.07083	0.857878	-0.676974	1.12244	2
13PDG	6	-0.113322	0.545112	-0.585073	1.12825	0
NADH	72	-1.07083	0.857878	-0.676974	1.12244	2
3PG	5	1.41804	0.0780898	0.192548	1.19748	1
2PG	3	-0.529431	0.701747	-0.881221	0.520031	0
23PDG	2	-0.0657214	0.5262	-0.585037	0.120475	0
PEP	6	2.15992	0.0153893	0.475657	1.60449	1
PYR	17	-1.49078	0.931991	-0.945964	0.954312	0
CO2	41	1.19364	0.116309	-0.31895	1.19354	3
OA	12	0.3554	0.361145	-0.414949	1.22727	1
ATPm	22	-0.888213	0.812787	-0.749033	0.708943	0
PYRm	9	-0.340188	0.633143	-0.661907	0.914549	0
CO2m	14	-0.598876	0.725372	-0.715379	0.875161	0
ADPm	14	-1.25542	0.895337	-0.916086	0.729376	0

PIm	15	-1.28612	0.900799	-0.912168	0.579852	0
OAm	10	-0.489844	0.687878	-0.709393	0.879523	0
GTP	10	-0.25187	0.599429	-0.623341	1.43676	1
GDP	7	0.718066	0.236358	-0.22197	1.35763	1
NADP	64	0.439375	0.330195	-0.4695	1.23601	3
D6PGL	2	-0.666681	0.747512	-1.07026	0.303823	0
NADPH	64	0.439375	0.330195	-0.4695	1.23601	3
D6PGC	7	0.473057	0.318086	-0.327829	0.882287	0
RL5P	5	-1.18183	0.881364	-1.13616	0.423815	0
XUL5P	6	1.24732	0.106139	0.0498248	1.91196	1
R5P	15	-0.290272	0.614196	-0.618041	0.893191	0
ACTP	2	2.56142	0.00521225	1.53614	3.08705	1
ACCOAm	11	-0.661141	0.745739	-0.76024	0.696257	0
H2Om	19	-1.19084	0.883142	-0.844907	1.56754	1
CITm	5	-0.711719	0.761681	-0.895904	0.992671	0
COAm	13	-1.05341	0.853924	-0.866458	0.767565	0
ACOm	3	-0.276876	0.609062	-0.714665	1.04047	0
ICITm	5	-0.74982	0.773318	-0.915376	0.799755	0
NADm	22	-0.257775	0.60171	-0.595229	1.63909	2

AKGm	9	-0.559439	0.712069	-0.745469	0.654957	0
NADHm	22	-0.257775	0.60171	-0.595229	1.63909	2
ICIT	2	-0.847735	0.801707	-1.21644	0.301861	0
AKG	19	1.59399	0.0554687	-0.113929	1.27738	1
NADPm	17	-0.598576	0.725272	-0.698405	1.59213	1
NADPHm	17	-0.598576	0.725272	-0.698405	1.59213	1
ICITg	2	-0.767698	0.778667	-1.15182	0.210471	0
NADPg	1	-0.412954	0.66018	-1.00299	0	0
AKGg	1	-0.412954	0.66018	-1.00299	0	0
CO2g	1	-0.412954	0.66018	-1.00299	0	0
NADPHg	1	-0.412954	0.66018	-1.00299	0	0
LIPOm	2	-0.35786	0.639776	-0.820912	1.25091	0
SUCDLIP	2	-1.81657	0.965358	-1.99868	0.414706	0
SUCCOAm	2	-1.13782	0.872402	-1.45066	1.18973	0
DHLIPOm	2	-0.908399	0.818166	-1.26542	1.45169	0
GDPm	2	0.520837	0.30124	-0.111444	0.7042	0
GTPm	2	0.520837	0.30124	-0.111444	0.7042	0
SUCCm	3	-0.591626	0.72295	-0.922238	0.270935	0
Qm	9	-0.0237816	0.509487	-0.541315	0.761675	0

FUMm	3	-1.51869	0.935579	-1.53362	0.788011	0
QH2m	9	-0.0237816	0.509487	-0.541315	0.761675	0
FADH2m	7	-0.478026	0.683684	-0.738754	0.684666	0
FADm	7	-0.478026	0.683684	-0.738754	0.684666	0
FUM	5	0.378987	0.352349	-0.338478	1.27959	0
SUCC	6	-0.402155	0.656215	-0.719847	0.68732	0
MALm	4	-1.35159	0.911746	-1.30426	1.36264	0
MAL	4	-1.33653	0.909312	-1.29566	1.36096	0
MALg	2	-1.73343	0.95849	-1.93156	0.611471	0
NADg	1	-1.60557	0.945815	-2.36393	0	0
OAg	1	-1.60557	0.945815	-2.36393	0	0
NADHg	1	-1.60557	0.945815	-2.36393	0	0
SUCCg	1	-0.67379	0.749778	-1.30064	0	0
GLXg	2	-1.07497	0.858806	-1.39991	0.140388	0
ACCOAg	2	-1.19078	0.883129	-1.49342	0.00815996	0
H2Og	1	-0.847772	0.801718	-1.49918	0	0
COAg	2	-1.19078	0.883129	-1.49342	0.00815996	0
Hm	11	0.304524	0.380364	-0.427274	1.94525	1
CIT	1	0.154805	0.438488	-0.3551	0	0

COA	37	-2.33082	0.990119	-0.971057	0.794524	0
ACCOA	24	-1.38213	0.916535	-0.855209	0.849492	0
CAR	2	-0.987826	0.838381	-1.32955	0.256752	0
ACAR	2	-0.987826	0.838381	-1.32955	0.256752	0
ACARm	2	-0.9733	0.834798	-1.31782	0.240166	0
CARm	2	-0.9733	0.834798	-1.31782	0.240166	0
ACARg	2	-0.9733	0.834798	-1.31782	0.240166	0
CARg	2	-0.9733	0.834798	-1.31782	0.240166	0
OXAL	3	0.631206	0.263953	-0.115798	0.58641	0
AC	17	0.203844	0.419238	-0.475759	1.42564	1
FOR	7	0.714894	0.237337	-0.22334	0.629559	0
FORm	2	-0.662996	0.746333	-1.06728	1.44735	0
METHOL	4	-1.81692	0.965385	-1.57009	0.818631	0
FALD	8	-1.7361	0.958727	-1.23398	0.663832	0
ADHLIPOm	2	0.550306	0.291055	-0.0876506	0.213927	0
ACAL	13	-0.836012	0.798426	-0.797494	1.80882	1
RGT	7	-1.10768	0.865999	-1.0108	0.569354	0
FGT	2	-0.866278	0.806831	-1.23141	0.385075	0
H+	25	2.59304	0.00475662	0.0611572	1.59907	3

HCIT	1	1.20401	0.114293	0.842193	0	0
MTHGXL	3	-0.520369	0.698597	-0.875245	0.860177	0
LACAL	5	-1.16239	0.877461	-1.12622	0.747573	0
LAC	4	-1.03205	0.848975	-1.12171	0.554444	0
LGT	2	-0.258556	0.602011	-0.740733	0.2417	0
LLAC	3	-1.96523	0.975306	-1.82811	0.470484	0
PROP	2	-0.382162	0.648829	-0.840534	0.240852	0
AMP	38	1.24357	0.10683	-0.301424	1.27916	3
PPI	45	-0.282576	0.611249	-0.580622	1.20557	2
PROPCOA	3	-1.07522	0.858862	-1.24116	0.714502	0
2MCIT	1	-1.32382	0.907218	-2.04242	0	0
GLU	34	3.02223	0.00125458	0.060957	1.36958	3
GABA	3	-0.644196	0.740276	-0.956907	1.09847	0
SUCCSAL	5	-0.201959	0.580026	-0.635381	0.413597	0
METTHF	6	0.411873	0.340216	-0.340009	0.500094	0
METHF	2	-0.492963	0.688981	-0.929995	1.6415	0
METTHFm	6	-0.300037	0.617926	-0.672198	0.794413	0
MTHFm	2	0.0218124	0.491299	-0.514361	0.263641	0
METHFm	2	-0.492963	0.688981	-0.929995	1.6415	0

FTHFm	1	-1.36614	0.914052	-2.09071	0	0
FTHF	2	-0.735664	0.769032	-1.12595	1.36437	0
THFm	3	-0.828411	0.796281	-1.07839	0.928945	0
AHTD	2	1.5848	0.0565059	0.747609	0.32259	0
DHP	2	0.853015	0.196825	0.15676	1.15818	0
AHHMP	1	-0.114307	0.545503	-0.662195	0	0
GLAL	1	-0.114307	0.545503	-0.662195	0	0
CHOR	3	1.55777	0.0596436	0.495259	0.90421	0
GLN	13	3.42164	0.000311222	0.553125	1.45636	2
PABA	1	-0.114307	0.545503	-0.662195	0	0
AHHMD	1	-0.114307	0.545503	-0.662195	0	0
DHPT	1	-0.114307	0.545503	-0.662195	0	0
DHF	1	0.382292	0.351122	-0.0955047	0	0
THF	8	0.177323	0.429628	-0.460562	0.40049	0
MTHF	1	0.168558	0.433072	-0.339406	0	0
THFG	2	0.0342621	0.486334	-0.504309	0.536739	0
OIVAL	2	0.0296613	0.488169	-0.508024	0.722382	0
AKP	1	-0.426827	0.665247	-1.01883	0	0
PANT	1	-0.998878	0.841073	-1.67162	0	0

AKPm	1	0.250678	0.401032	-0.245695	0	0
PANTm	1	0.250678	0.401032	-0.245695	0	0
bALA	1	-0.998878	0.841073	-1.67162	0	0
PNTO	2	-1.14483	0.873861	-1.45632	0.304473	0
4PPNTO	1	-0.621545	0.73288	-1.24103	0	0
CTP	7	-0.98867	0.838588	-0.959384	0.72679	0
CYS	7	-1.21271	0.88738	-1.05618	0.886969	0
CMP	8	-0.186046	0.573796	-0.607437	1.32921	1
ASP	18	2.95762	0.0015501	0.265243	1.2443	2
PAP	3	-0.227412	0.589948	-0.682045	1.06386	0
ACP	10	-0.563465	0.713441	-0.736015	0.771073	0
ALA	6	-1.12002	0.868647	-1.05481	0.522174	0
CHCOA	1	0.240087	0.405131	-0.257781	0	0
AONA	1	0.240087	0.405131	-0.257781	0	0
DTB	1	-0.538398	0.704849	-1.14614	0	0
BT	1	-0.538398	0.704849	-1.14614	0	0
ETH	4	-1.81692	0.965385	-1.57009	0.818631	0
ETHm	4	-1.81692	0.965385	-1.57009	0.818631	0
ACALm	9	-0.596154	0.724464	-0.759462	2.16225	1

ACm	9	0.293278	0.384655	-0.420475	1.99479	1
AMPm	9	0.444072	0.328495	-0.363003	0.632091	0
PPIm	11	0.021495	0.491425	-0.524864	0.606975	0
ACTPm	1	-0.100758	0.540129	-0.646734	0	0
GLYN	11	-0.857076	0.804299	-0.827799	0.668521	0
GL	14	-1.42234	0.922536	-0.967114	0.833863	0
GLYAL	9	-1.342	0.910203	-1.04373	0.961567	0
O2	34	1.70996	0.0436365	-0.19667	1.27594	3
H2O2	10	0.839106	0.200705	-0.22884	1.55619	1
GL3P	7	-0.311965	0.622466	-0.667006	0.725247	0
TAR	2	-0.175766	0.569761	-0.673888	0.303208	0
OXGLY	2	-0.175766	0.569761	-0.673888	0.303208	0
G	1	2.34257	0.00957582	2.14145	0	1
E	6	-0.488279	0.687324	-0.760034	0.7062	0
EOL	6	-0.488279	0.687324	-0.760034	0.7062	0
LXUL	6	-0.974273	0.83504	-0.986806	0.824706	0
XOL	6	-0.45236	0.674495	-0.743274	0.849494	0
XUL	7	-0.0711986	0.52838	-0.56298	0.910443	0
AOL	6	-0.45236	0.674495	-0.743274	0.849494	0

XYL	3	-0.136602	0.554327	-0.622157	0.36705	0
ARAB	2	-0.668463	0.748081	-1.0717	0.305859	0
ARABLAC	2	-0.668463	0.748081	-1.0717	0.305859	0
LAOL	3	-0.875156	0.809256	-1.10922	0.173276	0
RIB	3	-0.0296228	0.511816	-0.551605	0.502694	0
R1P	2	-0.828746	0.796376	-1.20111	1.15723	0
RL	2	-1.1389	0.872627	-1.45153	0.314613	0
O2e	7	0.173646	0.431072	-0.457193	1.75675	1
GLCN15LACe	3	-0.685782	0.753575	-0.984333	0.908861	0
H2O2e	7	0.173646	0.431072	-0.457193	1.75675	1
H2Oe	19	-0.641278	0.739329	-0.700655	1.26883	1
GLCNTe	1	-0.0540465	0.521551	-0.593429	0	0
GLCN15LAC	1	-0.0540465	0.521551	-0.593429	0	0
GLCNT	6	0.6163	0.268848	-0.244621	0.934338	0
GLAC	18	1.68266	0.0462208	-0.078571	1.22006	2
GALOL	5	-0.239263	0.594549	-0.654446	0.734699	0
GAL1P	2	0.871223	0.191816	0.17146	0.88634	0
UTP	7	-0.426765	0.665225	-0.716606	0.703256	0
UDPGAL	4	-0.165419	0.565693	-0.626627	0.841614	0

UDPG	10	-0.737359	0.769548	-0.798896	0.938112	0
G1P	4	-0.476957	0.683304	-0.804602	0.907766	0
MELI	4	0.626596	0.265462	-0.174167	0.968972	0
GALN14LAC	3	1.02039	0.153772	0.140861	1.54378	1
GALNT	2	-0.86549	0.806615	-1.23078	1.2087	0
2D3DGALT	2	-0.86549	0.806615	-1.23078	1.2087	0
SOR	8	0.738625	0.230067	-0.233681	1.41682	1
SOT	4	-0.431497	0.666946	-0.778631	1.05499	0
MAN6P	5	-1.64506	0.950021	-1.3729	0.336646	0
MAN	4	0.297774	0.382938	-0.362015	2.04845	1
FRU	8	-0.148533	0.559039	-0.592274	1.52523	1
MNT6P	1	1.64997	0.0494744	1.3511	0	0
MNT	4	-0.508344	0.694394	-0.822533	1.04431	0
F26P	1	-0.596939	0.724726	-1.21295	0	0
MAN1P	1	-1.28624	0.900821	-1.99954	0	0
GDPMAN	2	-0.404629	0.657125	-0.858674	1.61343	0
IDOL	3	0.0707343	0.471805	-0.485421	0.60009	0
UDP	17	-0.897555	0.815288	-0.781362	0.849222	0
TRE6P	3	-0.96722	0.833283	-1.16994	0.787922	0

TRE	3	-1.19226	0.883421	-1.31835	0.549941	0
MLT	5	-0.964388	0.832574	-1.02503	0.822427	0
MLTe	5	-0.964388	0.832574	-1.02503	0.822427	0
LACT	4	0.333028	0.369557	-0.341876	1.28452	0
LACTe	4	0.333028	0.369557	-0.341876	1.28452	0
GLACe	5	1.56326	0.0589952	0.266768	1.75776	1
13GLUCAN	2	0.231158	0.408596	-0.345334	1.02883	0
GA6P	4	-0.994753	0.840072	-1.10041	0.5875	0
NAGA6P	2	0.273923	0.392072	-0.310805	0.0828462	0
NAGA1P	2	0.212711	0.415776	-0.360228	0.0129516	0
UDPNAG	9	0.242892	0.404044	-0.439678	0.720613	0
CHIT	13	0.234286	0.407381	-0.457972	1.03815	1
NAG	5	0.122577	0.451221	-0.469521	1.4821	1
GLCN	3	-1.2905	0.901561	-1.38313	0.195255	0
13GLUCANe	12	0.129476	0.44849	-0.489538	1.27407	1
GLYCOGEN	2	-1.1841	0.881812	-1.48802	0.871487	0
STARe	2	-0.331424	0.629838	-0.799567	0.358207	0
GLYCOGENe	2	-0.331424	0.629838	-0.799567	0.358207	0
AMYLSe	1	-0.925923	0.822757	-1.58836	0	0

AMYLPe	1	-0.925923	0.822757	-1.58836	0	0
CELLUe	8	-1.98441	0.976395	-1.33435	0.6525	0
CELLOBe	8	-1.98441	0.976395	-1.33435	0.6525	0
CELLOTe	4	-1.42533	0.92297	-1.34639	0.719473	0
MANNANe	6	-0.91657	0.820316	-0.959881	0.78176	0
MANe	7	0.220731	0.412651	-0.436849	1.55699	1
PECTATEe	1	-0.312436	0.622645	-0.888288	0	0
GALUNTe	1	-0.312436	0.622645	-0.888288	0	0
ARABINe	5	-0.335563	0.6314	-0.703662	0.250564	0
LARABe	5	-0.335563	0.6314	-0.703662	0.250564	0
XYLANe	6	-0.744208	0.771625	-0.879454	0.504926	0
XYLe	6	-0.744208	0.771625	-0.879454	0.504926	0
H+_PO_mit	8	-0.846572	0.801383	-0.874424	0.658059	0
H+_PO	8	-0.846572	0.801383	-0.874424	0.658059	0
FERIm	5	-1.7016	0.955584	-1.4018	0.291574	0
FEROm	5	-1.7016	0.955584	-1.4018	0.291574	0
O2m	1	-0.697592	0.757284	-1.32781	0	0
K	1	-0.0501187	0.519986	-0.588947	0	0
Km	1	-0.0501187	0.519986	-0.588947	0	0

Ca	1	0.638786	0.261481	0.197191	0	0
Cam	1	0.638786	0.261481	0.197191	0	0
LLACm	6	0.384372	0.350351	-0.352842	2.54622	1
LACm	1	-0.685359	0.753441	-1.31385	0	0
GLUm	10	-0.246114	0.597203	-0.62126	0.690688	0
ASPm	6	0.554221	0.289714	-0.273588	0.505072	0
ALAm	1	-0.324552	0.62724	-0.902115	0	0
ASN	5	1.27868	0.100504	0.121327	2.04722	1
SAM	12	-0.258658	0.60205	-0.617681	1.34664	1
HCYS	8	0.154444	0.43863	-0.469809	0.85746	0
SAH	8	-1.22148	0.889047	-1.02596	0.936214	0
MET	4	-0.995317	0.840209	-1.10073	0.983959	0
TRNAm	4	0.0234241	0.490656	-0.518745	0.652376	0
ASPTRNAm	3	0.19318	0.423409	-0.40467	0.748541	0
TRNA	3	0.0506474	0.479803	-0.498668	0.730762	0
ASPTRNA	3	0.0506474	0.479803	-0.498668	0.730762	0
NH3	29	0.0649155	0.474121	-0.518564	1.48222	2
NAGLUm	2	-0.290117	0.614137	-0.766216	0.17658	0
NAGLUPm	1	-0.31488	0.623573	-0.891077	0	0

NAGLUSm	2	-0.66334	0.746444	-1.06756	0.249584	0
NAORNm	2	-0.508696	0.694517	-0.942699	0.426164	0
ORNm	1	-0.0960447	0.538257	-0.641355	0	0
CAP	5	3.17254	0.000755554	1.08922	1.56798	1
ORN	5	5.62128	3.03804e-008	2.34069	2.44372	3
CITR	2	3.99741	3.20191e-005	2.69557	1.44736	2
GLUGSAL	3	1.48027	0.0694003	0.444148	2.83657	1
ARGSUCC	2	2.33651	0.00973224	1.35455	0.449132	1
ARG	3	4.2356	3.70442e-005	2.26125	2.03587	1
PTRSC	2	0.684942	0.24669	0.0210556	1.24031	0
DSAM	2	0.157687	0.437352	-0.404655	0.638267	0
SPRMD	3	-1.31122	0.905109	-1.3968	0.966938	0
5MTA	1	-0.284122	0.611842	-0.855978	0	0
SPRM	2	-1.42752	0.923284	-1.68456	1.17179	0
GBAD	3	2.18864	0.0143114	0.911308	3.22715	1
GBAT	3	2.18864	0.0143114	0.911308	3.22715	1
UREA	3	1.84755	0.0323335	0.686365	3.40851	1
ATRNA	1	1.46095	0.0720149	1.13539	0	0
ALTRNA	1	1.46095	0.0720149	1.13539	0	0

DAPRP	1	-0.253717	0.600143	-0.821281	0	0
SLF	1	-0.583591	0.720252	-1.19772	0	0
APS	2	-0.00236378	0.500943	-0.533881	0.938803	0
PAPS	2	0.239523	0.40535	-0.33858	0.662605	0
SER	11	-0.643682	0.740109	-0.75422	0.896183	0
ASER	5	-0.0185332	0.507393	-0.541638	0.450123	0
H2S	5	0.433186	0.33244	-0.310779	0.217909	0
RTHIO	4	-1.25628	0.895492	-1.24981	0.752614	0
OTHIO	4	-1.25628	0.895492	-1.24981	0.752614	0
H2SO3	2	0.044878	0.482102	-0.495738	0.440349	0
GLUGSALm	3	-0.709715	0.761059	-1.00012	1.06464	0
P5Cm	4	0.767226	0.221474	-0.0938282	2.01024	1
PHP	1	0.956507	0.169408	0.559756	0	0
GLYm	2	-0.0498677	0.519886	-0.572236	0.434396	0
GLY	11	-0.486831	0.686811	-0.700137	0.673826	0
GLX	2	-1.0583	0.855042	-1.38646	0.0932604	0
BASP	2	2.12651	0.0167304	1.18499	0.577242	0
ASPSA	2	0.902852	0.183302	0.196998	0.819989	0
HSER	3	0.215526	0.414679	-0.389933	0.853172	0

PHSER	2	2.0397	0.0206901	1.1149	0.926648	1
THR	8	-0.973778	0.834917	-0.925842	1.2174	1
LLCT	5	0.177188	0.42968	-0.441611	0.561067	0
OBUT	4	-1.1834	0.881675	-1.20818	1.14106	0
THRm	3	-0.246853	0.597489	-0.694865	1.21301	0
NH3m	3	-0.246853	0.597489	-0.694865	1.21301	0
OBUTm	4	0.26925	0.393868	-0.37831	1.17548	0
PRPP	9	0.458138	0.323427	-0.357643	1.22274	0
PRBATP	3	0.132018	0.447485	-0.445005	2.27664	0
PRBAMP	2	-1.15405	0.87576	-1.46376	2.03444	0
PRFP	3	-0.288446	0.613497	-0.722296	1.92842	0
PRLP	2	1.94956	0.025614	1.04212	0.398077	0
DIMGP	2	1.95109	0.0255234	1.04335	0.39634	0
IMACP	2	2.07675	0.0189124	1.14481	0.539829	0
HISOLP	2	0.851455	0.197258	0.1555	1.93893	0
HISOL	3	-1.28729	0.901004	-1.38102	1.44569	0
HIS	4	-1.1034	0.865074	-1.16248	1.30606	0
AICAR	4	1.28285	0.0997717	0.200738	0.74997	0
HTRNA	1	-0.774768	0.780762	-1.41588	0	0

HHTRNA	1	-0.774768	0.780762	-1.41588	0	0
MHIS	1	0.197389	0.421761	-0.306505	0	0
OICAPm	1	0.468418	0.319743	0.00277732	0	0
LEUm	1	0.468418	0.319743	0.00277732	0	0
OMVALm	2	0.794825	0.213358	0.109776	0.151319	0
ILEm	1	0.468418	0.319743	0.00277732	0	0
OMVAL	1	0.468418	0.319743	0.00277732	0	0
ILE	1	0.468418	0.319743	0.00277732	0	0
VAL	4	0.827388	0.204009	-0.0594589	2.26282	1
OICAP	3	0.614568	0.26942	-0.126771	0.261333	0
LEU	1	0.468418	0.319743	0.00277732	0	0
ABUTm	2	0.860534	0.194748	0.16283	0.577742	0
ACLACm	2	0.860534	0.194748	0.16283	0.577742	0
DHVALm	2	0.640954	0.260776	-0.0144604	0.327016	0
DHMVAm	2	0.640954	0.260776	-0.0144604	0.327016	0
OIVALm	2	1.06763	0.142844	0.330041	0.160182	0
IPPMALm	1	0.85446	0.196425	0.443307	0	0
CBHCAP	1	1.6953	0.0450095	1.40282	0	0
IPPMAL	3	1.32221	0.0930486	0.33991	0.950286	0

PPMAL	1	1.6953	0.0450095	1.40282	0	0
HCITm	1	-0.0211248	0.508427	-0.555861	0	0
HACNm	2	0.704277	0.24063	0.0366666	0.83796	0
HICITm	2	0.989684	0.161164	0.267107	0.512069	0
OXAm	1	0.382751	0.350952	-0.0949809	0	0
MICIT	1	-0.941536	0.826785	-1.60618	0	0
AKA	1	-0.271697	0.607073	-0.8418	0	0
AMA	4	-0.947128	0.828213	-1.0732	1.80742	0
AMASA	2	1.55628	0.0598206	0.724583	0.910942	0
SACP	2	0.683827	0.247042	0.0201549	0.085269	0
LYS	3	0.838325	0.200924	0.0207938	0.45222	0
LTRNA	2	0.722352	0.235039	0.0512604	0.635166	0
LLTRNA	2	0.722352	0.235039	0.0512604	0.635166	0
LYSm	2	0.722352	0.235039	0.0512604	0.635166	0
LTRNAm	2	0.722352	0.235039	0.0512604	0.635166	0
LLTRNAm	2	0.722352	0.235039	0.0512604	0.635166	0
ADN	7	1.51697	0.0646372	0.123205	1.07666	1
MTHPTGLU	1	0.168558	0.433072	-0.339406	0	0
THPTGLU	1	0.168558	0.433072	-0.339406	0	0

OAHSER	2	-0.368395	0.643711	-0.829419	0.590038	0
METH	1	0.104769	0.45828	-0.412198	0	0
OSLHSER	1	0.847295	0.198415	0.43513	0	0
CALH	1	-0.19328	0.57663	-0.752314	0	0
DPTH	1	-0.19328	0.57663	-0.752314	0	0
3DDAH7P	3	1.57085	0.0581088	0.503882	1.53243	0
DQT	3	-0.125911	0.550099	-0.615106	0.839328	0
DHSK	2	-0.690007	0.754905	-1.08909	0.246934	0
QT	1	0.757677	0.224322	0.332863	0	0
SME	1	-0.641414	0.739373	-1.2637	0	0
SME5P	1	-0.641414	0.739373	-1.2637	0	0
3PSME	2	0.751097	0.226297	0.0744698	1.89246	0
PHEN	2	0.559037	0.288068	-0.080601	0.444882	0
PHPYR	2	0.34078	0.366635	-0.256824	0.442651	0
PHE	1	-0.0333623	0.513307	-0.569826	0	0
4HPP	6	2.47178	0.00672209	0.621175	1.58374	1
TYR	4	0.832592	0.202537	-0.056486	0.565023	0
AN	4	-0.281157	0.610705	-0.692746	0.783839	0
NPRAN	2	-0.748365	0.77288	-1.13621	0.134935	0

CPAD5P	1	-0.446081	0.672231	-1.0408	0	0
IGP	2	0.441494	0.329428	-0.175507	1.2237	0
TRP	3	0.786538	0.215776	-0.0133589	0.610848	0
FKYN	3	0.350986	0.362799	-0.300599	0.121427	0
KYN	4	-0.560222	0.712336	-0.85217	0.46974	0
HKYN	3	-0.829198	0.796504	-1.07891	0.150025	0
HAN	2	-0.584419	0.720531	-1.00384	0.105818	0
CMUSA	1	0.569637	0.284462	0.118282	0	0
AM6SA	2	-0.453996	0.675084	-0.898533	1.43799	0
AMUCO	1	-1.21246	0.887333	-1.91535	0	0
HOMOGEN	2	3.9986	3.18595e-005	2.69653	1.44601	2
MACAC	2	3.02611	0.00123861	1.91134	0.335581	2
FUACAC	2	2.03503	0.0209238	1.11113	1.46724	1
ACTAC	1	0.530513	0.297878	0.0736361	0	0
TRPm	1	-0.288497	0.613517	-0.860971	0	0
TRPTRNAm	1	-0.288497	0.613517	-0.860971	0	0
PAD	3	3.1011	0.000964021	1.51306	2.7331	1
PAC	3	3.1011	0.000964021	1.51306	2.7331	1
IAD	3	3.1011	0.000964021	1.51306	2.7331	1

IAC	3	3.1011	0.000964021	1.51306	2.7331	1
ASPERMD	2	-1.33725	0.90943	-1.61168	1.27486	0
APRUT	1	-0.156394	0.562139	-0.710223	0	0
APROA	1	-0.156394	0.562139	-0.710223	0	0
GABAL	1	-0.156394	0.562139	-0.710223	0	0
ASPRM	2	-1.33725	0.90943	-1.61168	1.27486	0
GLUP	2	-0.466403	0.679536	-0.90855	0.330896	0
P5C	1	-0.0889399	0.535435	-0.633248	0	0
PRO	1	-0.0889399	0.535435	-0.633248	0	0
PHC	1	-0.0889399	0.535435	-0.633248	0	0
HPRO	1	-0.0889399	0.535435	-0.633248	0	0
PROm	1	2.76634	0.00283446	2.62504	0	1
GABALm	5	0.824174	0.20492	-0.110957	2.76861	1
GABAm	5	0.824174	0.20492	-0.110957	2.76861	1
LACALm	5	0.824174	0.20492	-0.110957	2.76861	1
APROP	1	-1.14713	0.874336	-1.84079	0	0
TCOA	1	-0.161862	0.564293	-0.716462	0	0
GLP	1	-0.161862	0.564293	-0.716462	0	0
TGLP	1	-0.161862	0.564293	-0.716462	0	0

PEPD	1	-0.968109	0.833505	-1.6365	0	0
APEP	1	-0.968109	0.833505	-1.6365	0	0
GC	2	-0.545932	0.707444	-0.972763	0.362464	0
OGT	2	-0.836572	0.798583	-1.20743	1.15374	0
cAMP	3	-0.13391	0.553263	-0.620381	0.539374	0
GMP	6	1.63166	0.0513753	0.229163	1.164	1
DGMP	2	2.23597	0.0126769	1.27337	1.46992	1
DGDP	2	-0.793603	0.786287	-1.17273	0.95899	0
DATP	2	-1.42887	0.923479	-1.68565	0.233612	0
DADP	4	-0.176389	0.570006	-0.632894	0.921108	0
PRAM	1	1.10345	0.134915	0.727444	0	0
GAR	1	1.10345	0.134915	0.727444	0	0
FGAR	1	0.645604	0.259268	0.204972	0	0
FGAM	2	1.23628	0.108178	0.466208	0.369444	0
AIR	2	2.24102	0.0125124	1.27745	0.777822	1
CAIR	2	2.57371	0.00503075	1.54606	0.397943	1
SAICAR	3	1.26433	0.103055	0.30174	0.835731	0
PRFICA	1	0.324725	0.372695	-0.161197	0	0
IMP	9	0.599546	0.274404	-0.303748	1.27125	1

ASUC	3	0.653946	0.256573	-0.100802	0.147315	0
XMP	5	1.36513	0.0861056	0.165509	1.50236	1
cdAMP	1	-0.621545	0.73288	-1.24103	0	0
DAMP	5	1.47942	0.0695135	0.22392	1.33887	1
cIMP	1	-0.621545	0.73288	-1.24103	0	0
cGMP	1	-0.621545	0.73288	-1.24103	0	0
cCMP	1	-0.621545	0.73288	-1.24103	0	0
ATN	1	-1.30701	0.904396	-2.02324	0	0
ATT	2	-1.54055	0.938287	-1.77583	0.349898	0
UGC	2	-1.18705	0.882396	-1.49041	0.0537489	0
CAASP	3	1.5671	0.0585454	0.501411	0.752075	0
DOROA	1	1.59336	0.0555392	1.2865	0	0
OROA	2	0.952735	0.170362	0.237274	1.48383	0
OMP	2	-0.204659	0.581081	-0.697217	0.162263	0
UMP	6	0.429719	0.3337	-0.331682	1.46726	1
URA	6	0.0320496	0.487216	-0.517241	0.683654	0
CYTS	2	-0.839441	0.799389	-1.20975	0.881003	0
URI	4	1.48589	0.0686542	0.316728	1.40233	1
CYTD	4	1.48589	0.0686542	0.316728	1.40233	1

DU	4	1.68496	0.045998	0.430454	1.29157	1
DR1P	1	0.130512	0.448081	-0.382822	0	0
DT	3	1.90053	0.0286816	0.721304	1.41232	1
THY	1	0.130512	0.448081	-0.382822	0	0
DC	3	1.87057	0.0307022	0.701546	1.43571	1
DTMP	4	1.26992	0.102057	0.193347	1.64097	1
DTDP	2	-1.52633	0.936536	-1.76434	0.12233	0
OTHIOm	1	-1.44178	0.925318	-2.17704	0	0
RTHIOm	1	-1.44178	0.925318	-2.17704	0	0
DUTP	3	-1.52449	0.936306	-1.53745	0.305263	0
DUMP	5	1.00209	0.158151	-0.0200324	1.47207	1
DCMP	3	1.43171	0.0761132	0.412123	1.81812	1
DCDP	3	-1.04103	0.851069	-1.21861	0.682748	0
CDP	4	-0.848147	0.801822	-1.01665	0.688412	0
PURI5P	3	0.64122	0.26069	-0.109194	0.276527	0
AD	5	-1.001	0.841587	-1.04375	0.635502	0
INS	5	1.67982	0.0464962	0.326336	1.14061	1
DA	4	1.69657	0.0448894	0.437083	1.28565	1
DIN	2	0.164441	0.434692	-0.399202	0.0231648	0

HYXN	3	0.329044	0.371061	-0.315069	0.146639	0
DG	4	0.927164	0.176921	-0.00245884	1.8507	1
GN	3	-0.0178853	0.507135	-0.543865	0.497529	0
GSN	6	0.701763	0.241414	-0.204742	1.50012	1
XAN	2	0.330885	0.370366	-0.264813	0.166889	0
XTSINE	3	1.90053	0.0286816	0.721304	1.41232	1
ITP	2	-0.841894	0.800076	-1.21173	0.903849	0
IDP	2	-0.841894	0.800076	-1.21173	0.903849	0
ITPm	1	0.80468	0.210502	0.386501	0	0
IDPm	1	0.80468	0.210502	0.386501	0	0
DGTP	3	-1.99593	0.977029	-1.84835	0.326649	0
DUDP	3	-1.04103	0.851069	-1.21861	0.682748	0
DCTP	2	-1.42887	0.923479	-1.68565	0.233612	0
DTTP	1	-1.15594	0.876147	-1.85084	0	0
LCCA	4	-0.548955	0.708482	-0.845733	1.01647	0
ACOA	7	-0.806886	0.790134	-0.880842	0.8072	0
HACOA	3	-1.13169	0.871117	-1.2784	0.820683	0
OACOA	6	-1.22335	0.889401	-1.10303	0.68262	0
AACCOA	3	-0.616725	0.731292	-0.93879	0.641537	0

AACCOAm	2	-0.282261	0.611128	-0.759873	0.7944	0
ACACPm	2	-1.28045	0.899806	-1.56582	0.641459	0
MALACPm	2	-1.28045	0.899806	-1.56582	0.641459	0
C100ACPm	1	-0.508686	0.694514	-1.11224	0	0
ACPm	2	-1.28045	0.899806	-1.56582	0.641459	0
C120ACPm	1	-0.508686	0.694514	-1.11224	0	0
C140ACPm	1	-0.508686	0.694514	-1.11224	0	0
C141ACPm	1	-0.508686	0.694514	-1.11224	0	0
C160ACPm	1	-0.508686	0.694514	-1.11224	0	0
C161ACPm	1	-0.508686	0.694514	-1.11224	0	0
C180ACPm	1	-0.508686	0.694514	-1.11224	0	0
C181ACPm	1	-0.508686	0.694514	-1.11224	0	0
C182ACPm	1	-0.508686	0.694514	-1.11224	0	0
C150ACPm	1	-0.508686	0.694514	-1.11224	0	0
C162ACPm	1	-0.508686	0.694514	-1.11224	0	0
C170ACPm	1	-0.508686	0.694514	-1.11224	0	0
C183ACPm	1	-0.508686	0.694514	-1.11224	0	0
C200ACPm	1	-0.508686	0.694514	-1.11224	0	0
MALCOA	7	0.569209	0.284607	-0.286285	0.978075	0

MALACP	4	-0.105438	0.541986	-0.592361	0.890812	0
ACACP	4	-0.956777	0.83066	-1.07871	0.793381	0
3OACPm	1	-1.30364	0.903822	-2.0194	0	0
C100ACP	1	-0.637933	0.738241	-1.25973	0	0
C120ACP	9	-0.0326997	0.513043	-0.544714	0.72444	0
C140ACP	9	-0.0326997	0.513043	-0.544714	0.72444	0
C141ACP	9	-0.0326997	0.513043	-0.544714	0.72444	0
C160ACP	9	-0.0326997	0.513043	-0.544714	0.72444	0
C161ACP	9	-0.0326997	0.513043	-0.544714	0.72444	0
C180ACP	9	-0.0326997	0.513043	-0.544714	0.72444	0
C181ACP	9	-0.0326997	0.513043	-0.544714	0.72444	0
C182ACP	9	-0.0326997	0.513043	-0.544714	0.72444	0
3HPACP	3	-0.737401	0.769561	-1.01837	0.960383	0
2HDACP	3	-0.737401	0.769561	-1.01837	0.960383	0
AACP	3	-0.737401	0.769561	-1.01837	0.960383	0
23DAACP	3	-0.737401	0.769561	-1.01837	0.960383	0
C150ACP	6	0.359643	0.359557	-0.364381	0.797485	0
C162ACP	9	-0.0326997	0.513043	-0.544714	0.72444	0
C170ACP	9	-0.0326997	0.513043	-0.544714	0.72444	0

C183ACP	9	-0.0326997	0.513043	-0.544714	0.72444	0
C200ACP	9	-0.0326997	0.513043	-0.544714	0.72444	0
C140	1	-0.773943	0.780518	-1.41493	0	0
C160	1	-0.773943	0.780518	-1.41493	0	0
C180	1	-0.773943	0.780518	-1.41493	0	0
AGL3P	3	-0.0866777	0.534536	-0.589232	0.692739	0
AT3P2	2	-0.174556	0.569286	-0.672911	0.957999	0
PA	5	-0.388424	0.651149	-0.730678	0.875696	0
PAm	1	0.515977	0.302935	0.0570492	0	0
CTPm	2	-0.45197	0.674355	-0.896897	1.34908	0
CDPDGm	3	0.898799	0.18438	0.0606752	0.872724	0
CDPDG	2	-0.183905	0.572956	-0.680459	1.04299	0
PS	3	0.040082	0.484014	-0.505636	1.49262	0
CMPm	3	0.207646	0.417753	-0.39513	1.17893	0
PSm	1	0.0912982	0.463628	-0.42757	0	0
PE	6	-0.189004	0.574955	-0.620388	0.963024	0
PEm	1	0.0912982	0.463628	-0.42757	0	0
PMME	2	-0.346111	0.63537	-0.811426	0.447449	0
PDME	1	-0.522341	0.699284	-1.12782	0	0

PC	3	-0.627762	0.73492	-0.946069	0.180701	0
CHO	1	0.757677	0.224322	0.332863	0	0
PCHO	2	0.398166	0.345254	-0.21049	0.768416	0
CDPCHO	2	-0.282592	0.611255	-0.76014	0.00890704	0
DAGLY	11	-0.48043	0.684539	-0.69793	0.878645	0
PETHM	2	-0.811873	0.791568	-1.18749	0.697853	0
CDPETN	2	-0.856713	0.804198	-1.22369	0.646653	0
MI1P	2	-0.70497	0.759585	-1.10117	0.81992	0
MYOI	2	-1.26018	0.896198	-1.54946	0.185951	0
PINS	7	-0.891906	0.813778	-0.917576	0.840164	0
PINSP	4	0.00895827	0.496426	-0.52701	0.961941	0
PINS4P	3	-1.14784	0.874482	-1.28905	0.451039	0
D45PI	3	-1.32247	0.906994	-1.40422	0.682432	0
TPI	2	-1.01047	0.843865	-1.34784	0.955172	0
GL3Pm	1	1.28552	0.0993054	0.935206	0	0
PGPm	2	0.543591	0.293362	-0.0930729	1.45421	0
PGm	2	-0.537297	0.704469	-0.965791	0.219997	0
CLm	1	-0.244032	0.596397	-0.81023	0	0
DGPP	2	-0.0327995	0.513083	-0.558455	0.464514	0

LPC	1	-0.361213	0.64103	-0.94395	0	0
LPE	1	-0.361213	0.64103	-0.94395	0	0
CDPm	2	-1.29876	0.902987	-1.5806	0.382175	0
PALCOA	2	-0.780733	0.78252	-1.16234	1.01858	0
DHSPH	3	-0.478094	0.683708	-0.847365	0.903542	0
SPH	4	-0.230062	0.590978	-0.663556	0.726284	0
PSPH	3	-0.498356	0.690883	-0.860728	0.747864	0
C260COA	1	0.267465	0.394556	-0.226539	0	0
CER2	2	-0.217361	0.586037	-0.707472	0.680142	0
CER3	2	-0.892269	0.813876	-1.2524	0.0905017	0
IPC	2	0.0184198	0.492652	-0.5171	1.13037	0
MIPC	2	0.0184198	0.492652	-0.5171	1.13037	0
MIP2C	1	-0.687591	0.754145	-1.31639	0	0
DHSP	3	-0.436912	0.668913	-0.820207	0.809531	0
PHSP	1	-1.01097	0.843985	-1.68542	0	0
C16A	1	-0.142204	0.55654	-0.694029	0	0
H3MCOA	3	-0.92473	0.822447	-1.14192	0.321538	0
MVL	3	-0.476985	0.683314	-0.846634	0.477399	0
PMVL	2	-0.566211	0.714375	-0.989137	0.817916	0

PPMVL	2	-1.21276	0.887389	-1.51116	0.0796585	0
IPPP	3	-0.517212	0.697496	-0.873163	0.503754	0
DMPP	2	-0.0623638	0.524863	-0.582326	0.00421095	0
GPP	1	-0.0417075	0.516634	-0.579349	0	0
FPP	1	-0.0417075	0.516634	-0.579349	0	0
S23E	1	-1.07071	0.857849	-1.75358	0	0
LNST	4	-0.606191	0.727806	-0.878431	0.690556	0
IGST	5	-0.508178	0.694336	-0.79188	0.638052	0
DMZYMST	5	-0.536383	0.704153	-0.806295	1.36573	0
IMZYMST	4	-0.163637	0.564992	-0.62561	1.44607	0
IIMZYMST	2	-0.527902	0.701216	-0.958206	0.407266	0
MZYMST	4	-0.415687	0.66118	-0.7696	1.48026	0
IZYMST	4	-0.163637	0.564992	-0.62561	1.44607	0
IIZYMST	2	-0.527902	0.701216	-0.958206	0.407266	0
ZYMST	2	-0.873971	0.808933	-1.23762	0.01211	0
FEST	2	-0.787454	0.784492	-1.16777	0.0866817	0
EPST	3	-0.267652	0.605517	-0.708582	0.345671	0
ERTROL	3	-0.296839	0.616705	-0.727831	0.378915	0
ERTEOL	2	-0.326877	0.62812	-0.795896	0.520892	0

ERGOST	1	0.0912982	0.463628	-0.42757	0	0
TAGLY	5	0.678686	0.248668	-0.185311	0.744637	0
MAGLY	4	0.597493	0.275089	-0.190793	0.859717	0
PHACAL	6	0.962451	0.167912	-0.0831016	2.47726	1
PHAC	6	0.219718	0.413045	-0.429672	2.59647	1
PHACCOA	2	-1.88551	0.970319	-2.05435	0.0439914	0
LLDACV	2	-1.7367	0.95878	-1.9342	1.13146	0
IPN	2	-1.33492	0.909048	-1.6098	0.672685	0
PENG	1	-1.36153	0.913327	-2.08546	0	0
NOR	4	2.54076	0.00553055	0.919354	1.46536	1
AVN	5	2.01189	0.0221159	0.496045	1.36775	1
HAVN	4	1.0556	0.145576	0.0709119	0.565162	0
AVF	2	0.280835	0.389419	-0.305224	0.907482	0
VHA	2	1.80293	0.0356993	0.923732	2.64549	1
VERB	3	3.13265	0.000866173	1.53387	1.95328	1
VERA	2	3.69471	0.00011007	2.45117	1.60687	1
DMST	2	3.69471	0.00011007	2.45117	1.60687	1
DHDMST	2	3.69471	0.00011007	2.45117	1.60687	1
ST	1	-1.43707	0.92465	-2.17165	0	0

DHST	1	-1.43707	0.92465	-2.17165	0	0
OMST	3	-0.91346	0.8195	-1.13448	0.911478	0
DHOMST	3	-0.91346	0.8195	-1.13448	0.911478	0
AFB1	2	-0.103944	0.541393	-0.615898	0.219101	0
AFG1	2	-0.103944	0.541393	-0.615898	0.219101	0
AFB2	2	-0.103944	0.541393	-0.615898	0.219101	0
AFG2	2	-0.103944	0.541393	-0.615898	0.219101	0
HNO3	3	-0.643183	0.739947	-0.95624	0.947354	0
HNO2	2	1.06355	0.143766	0.326747	0.357953	0
NH4OH	1	0.974123	0.164998	0.579859	0	0
UREAC	1	-0.431253	0.666858	-1.02388	0	0
ACNL	1	-1.14713	0.874336	-1.84079	0	0
INAC	1	-1.14713	0.874336	-1.84079	0	0
NH3e	2	0.64517	0.259408	-0.0110566	0.0411292	0
HNO3e	2	-1.16323	0.877632	-1.47118	0.451657	0
FRUe	1	2.83321	0.00230414	2.70134	0	1
SORe	1	2.83321	0.00230414	2.70134	0	1

#Results for Up-regulated only genes

#Feature	Number of neighbors	Z-score	P-value	Average Z	StdDev Z	Significance count
DGLCe	1	-1.57953	0.942893	-2.33422	0	0
GLCe	11	0.120424	0.452074	-0.490752	1.30359	1
bDGLCe	1	2.83321	0.00230414	2.70134	0	1
DGLC	2	-1.36044	0.913155	-1.63041	0.143498	0
GLC	15	-0.386321	0.650371	-0.646409	1.2489	1
bDGLC	6	0.0554499	0.47789	-0.506322	1.68131	1
ATP	66	1.41301	0.0788265	-0.333186	0.984317	1
ADP	46	0.231712	0.408381	-0.49327	0.89672	0
G6P	5	-1.07303	0.858371	-1.08056	0.796701	0
bDG6P	3	-0.609441	0.728884	-0.933987	0.762916	0
H2O	30	-0.228755	0.59047	-0.580168	1.09963	1
PI	41	2.53732	0.00558531	-0.0786904	1.24922	3
F6P	8	-1.87453	0.969571	-1.28993	0.32427	0
FDP	2	-0.722759	0.765086	-1.11553	0.707309	0
S7P	3	-1.04849	0.852794	-1.22354	0.352419	0
S17P	2	-0.722759	0.765086	-1.11553	0.707309	0
T3P2	5	-0.283115	0.611456	-0.676858	0.70802	0
T3P1	7	0.132292	0.447377	-0.47506	1.90529	1

E4P	5	1.08238	0.139541	0.0210054	1.26165	0
NAD	38	-1.23082	0.890804	-0.760966	0.993279	1
13PDG	4	-0.986893	0.838153	-1.09592	0.702207	0
NADH	38	-1.23082	0.890804	-0.760966	0.993279	1
3PG	3	0.463669	0.321442	-0.226287	0.682634	0
2PG	2	-0.668726	0.748165	-1.07191	0.568064	0
23PDG	1	-0.121344	0.548291	-0.670226	0	0
PEP	4	1.61498	0.0531578	0.390473	1.35286	0
PYR	11	-1.6937	0.954839	-1.11627	1.01134	0
CO2	21	-0.093996	0.537444	-0.555808	0.968469	1
OA	6	-0.109629	0.543648	-0.58335	0.698915	0
ATPm	15	-0.394388	0.653353	-0.648792	0.769878	0
PYRm	6	-0.452922	0.674698	-0.743536	0.943374	0
CO2m	8	-0.397042	0.654332	-0.692722	0.921897	0
ADPm	8	-0.770243	0.779422	-0.843572	0.856517	0
PIm	8	-0.703624	0.759167	-0.816644	0.601122	0
OAm	6	-0.481591	0.684952	-0.756913	0.792045	0
GTP	6	0.034921	0.486071	-0.515901	0.812433	0
GDP	6	-0.327308	0.628283	-0.684922	0.641439	0

NADP	38	-0.907968	0.818053	-0.701007	0.786541	0
D6PGL	1	-0.283634	0.611655	-0.855421	0	0
NADPH	38	-0.907968	0.818053	-0.701007	0.786541	0
D6PGC	2	0.733357	0.23167	0.0601466	1.29481	0
RL5P	3	-0.932813	0.824542	-1.14725	0.226623	0
XUL5P	4	1.79922	0.0359922	0.495725	2.29019	1
R5P	11	0.541675	0.294021	-0.345503	0.825307	0
ACTP	1	3.72501	9.76524e-005	3.71902	0	1
ACCOAm	6	0.447946	0.327096	-0.323177	0.486879	0
H2Om	7	-0.727909	0.766665	-0.846719	0.968295	0
CITm	4	-0.135044	0.553712	-0.609275	0.875302	0
COAm	8	-0.180134	0.571476	-0.605047	0.802455	0
ACOm	3	-0.276876	0.609062	-0.714665	1.04047	0
ICITm	4	-0.445703	0.672094	-0.786747	0.861689	0
NADm	10	-0.563011	0.713286	-0.735851	1.40854	1
AKGm	6	-0.0188506	0.50752	-0.540992	0.568607	0
NADHm	10	-0.563011	0.713286	-0.735851	1.40854	1
ICIT	1	-0.412954	0.66018	-1.00299	0	0
AKG	15	1.57872	0.0572	-0.0660245	1.38934	1

NADPm	10	-0.638792	0.738521	-0.763254	0.729392	0
NADPHm	10	-0.638792	0.738521	-0.763254	0.729392	0
ICITg	1	-0.412954	0.66018	-1.00299	0	0
NADPg	1	-0.412954	0.66018	-1.00299	0	0
AKGg	1	-0.412954	0.66018	-1.00299	0	0
CO2g	1	-0.412954	0.66018	-1.00299	0	0
NADPHg	1	-0.412954	0.66018	-1.00299	0	0
LIPOm	1	0.521734	0.300928	0.0636184	0	0
SUCDLIP	1	-1.54246	0.93852	-2.29193	0	0
SUCCOAm	2	-1.13782	0.872402	-1.45066	1.18973	0
DHLIPOm	2	-0.908399	0.818166	-1.26542	1.45169	0
GDPm	2	0.520837	0.30124	-0.111444	0.7042	0
GTPm	2	0.520837	0.30124	-0.111444	0.7042	0
SUCCm	2	-0.386487	0.650432	-0.844026	0.331827	0
Qm	6	0.0914318	0.463575	-0.489532	0.935973	0
FUMm	1	-0.479264	0.684125	-1.07866	0	0
QH2m	6	0.0914318	0.463575	-0.489532	0.935973	0
FADH2m	3	0.191026	0.424253	-0.406091	0.900267	0
FADm	3	0.191026	0.424253	-0.406091	0.900267	0

FUM	3	1.14924	0.125229	0.225837	0.7046	0
SUCC	4	-0.250218	0.598791	-0.675071	0.879574	0
MALm	2	-1.46666	0.928766	-1.71617	0.281684	0
MAL	1	-1.21246	0.887333	-1.91535	0	0
MALg	1	-1.60557	0.945815	-2.36393	0	0
NADg	1	-1.60557	0.945815	-2.36393	0	0
OAg	1	-1.60557	0.945815	-2.36393	0	0
NADHg	1	-1.60557	0.945815	-2.36393	0	0
Hm	5	-0.698739	0.757642	-0.88927	0.917839	0
CIT	1	0.154805	0.438488	-0.3551	0	0
COA	20	-0.932281	0.824404	-0.770858	0.671283	0
ACCOA	12	-0.831843	0.797251	-0.806919	0.723924	0
CAR	1	-0.858218	0.804614	-1.5111	0	0
ACAR	1	-0.858218	0.804614	-1.5111	0	0
OXAL	1	0.10353	0.458771	-0.413612	0	0
AC	8	-0.468176	0.680171	-0.721475	0.519302	0
FOR	1	0.921233	0.178465	0.519503	0	0
METHOL	2	-1.61596	0.946948	-1.83671	0.470946	0
FALD	3	-1.52165	0.935951	-1.53557	0.618827	0

ADHLIPOm	2	0.550306	0.291055	-0.0876506	0.213927	0
ACAL	4	-1.81317	0.965097	-1.56795	0.543783	0
RGT	3	-0.513314	0.696134	-0.870593	0.571841	0
FGT	1	-0.851732	0.802819	-1.5037	0	0
H+	10	-0.425411	0.664731	-0.686094	0.630258	0
HCIT	1	1.20401	0.114293	0.842193	0	0
MTHGXL	1	0.282437	0.388804	-0.209454	0	0
LACAL	1	0.282437	0.388804	-0.209454	0	0
LLAC	2	-1.81928	0.965566	-2.00088	0.513443	0
PROP	1	-0.419832	0.662696	-1.01084	0	0
AMP	26	2.42102	0.00773849	0.0110403	1.05957	2
PPI	29	1.01831	0.154266	-0.315928	1.06747	1
PROPCOA	1	-0.419832	0.662696	-1.01084	0	0
GLU	24	2.57632	0.00499288	0.069472	1.1918	1
SUCCSAL	2	-0.288136	0.613379	-0.764616	0.453211	0
METTHF	5	0.587075	0.278577	-0.232131	0.474695	0
METHF	1	0.668168	0.252013	0.23072	0	0
METTHFm	4	0.466251	0.320518	-0.265769	0.382799	0
MTHFm	2	0.0218124	0.491299	-0.514361	0.263641	0

METHFm	1	0.668168	0.252013	0.23072	0	0
FTHF	1	0.324725	0.372695	-0.161197	0	0
THFm	1	0.233698	0.40761	-0.265072	0	0
AHTD	2	1.5848	0.0565059	0.747609	0.32259	0
DHP	2	0.853015	0.196825	0.15676	1.15818	0
AHHMP	1	-0.114307	0.545503	-0.662195	0	0
GLAL	1	-0.114307	0.545503	-0.662195	0	0
CHOR	3	1.55777	0.0596436	0.495259	0.90421	0
GLN	9	2.36304	0.00906279	0.368369	0.820822	0
PABA	1	-0.114307	0.545503	-0.662195	0	0
AHHMD	1	-0.114307	0.545503	-0.662195	0	0
DHPT	1	-0.114307	0.545503	-0.662195	0	0
DHF	1	0.382292	0.351122	-0.0955047	0	0
THF	6	0.454308	0.324804	-0.320209	0.360544	0
MTHF	1	0.168558	0.433072	-0.339406	0	0
THFG	1	0.35664	0.360681	-0.124777	0	0
OIVAL	2	0.0296613	0.488169	-0.508024	0.722382	0
AKP	1	-0.426827	0.665247	-1.01883	0	0
AKPm	1	0.250678	0.401032	-0.245695	0	0

PANTm	1	0.250678	0.401032	-0.245695	0	0
PNTO	1	-0.621545	0.73288	-1.24103	0	0
4PPNTO	1	-0.621545	0.73288	-1.24103	0	0
CTP	5	-0.333986	0.630805	-0.702856	0.707963	0
CYS	5	-0.262863	0.603672	-0.666508	0.517696	0
CMP	8	-0.186046	0.573796	-0.607437	1.32921	1
ASP	14	2.79179	0.00262088	0.321156	1.01298	1
PAP	3	-0.227412	0.589948	-0.682045	1.06386	0
ACP	5	-0.738457	0.769882	-0.909568	0.570941	0
ALA	3	-0.709887	0.761113	-1.00023	0.796053	0
CHCOA	1	0.240087	0.405131	-0.257781	0	0
AONA	1	0.240087	0.405131	-0.257781	0	0
DTB	1	-0.538398	0.704849	-1.14614	0	0
BT	1	-0.538398	0.704849	-1.14614	0	0
ETH	2	-1.61596	0.946948	-1.83671	0.470946	0
ETHm	2	-1.61596	0.946948	-1.83671	0.470946	0
ACALm	3	-1.92585	0.972939	-1.80214	0.33835	0
ACm	2	-1.0403	0.8509	-1.37192	0.510641	0
AMPm	8	0.51369	0.303734	-0.3246	0.664414	0

PPIm	9	0.294036	0.384365	-0.420186	0.605151	0
GLYN	4	-0.067728	0.526999	-0.570819	0.469609	0
GL	8	-0.704792	0.75953	-0.817116	0.753514	0
GLYAL	4	-0.834015	0.797864	-1.00858	1.00258	0
O2	13	-0.186064	0.573803	-0.591317	0.641936	0
H2O2	4	0.748205	0.227168	-0.104695	0.937558	0
GL3P	6	0.0499377	0.480086	-0.508894	0.648988	0
TAR	1	0.0633285	0.474752	-0.459488	0	0
OXGLY	1	0.0633285	0.474752	-0.459488	0	0
E	4	0.307527	0.379221	-0.356444	0.357316	0
EOL	4	0.307527	0.379221	-0.356444	0.357316	0
LXUL	3	-0.903707	0.816925	-1.12805	1.23562	0
XOL	5	-0.517645	0.697647	-0.796718	0.938418	0
XUL	6	-0.097024	0.538646	-0.577468	0.996456	0
AOL	5	-0.517645	0.697647	-0.796718	0.938418	0
XYL	3	-0.136602	0.554327	-0.622157	0.36705	0
ARAB	1	-0.283634	0.611655	-0.855421	0	0
ARABLAC	1	-0.283634	0.611655	-0.855421	0	0
LAOL	1	-0.644334	0.740321	-1.26703	0	0

RIB	3	-0.0296228	0.511816	-0.551605	0.502694	0
R1P	1	0.130512	0.448081	-0.382822	0	0
RL	1	-0.611062	0.729421	-1.22906	0	0
O2e	1	-0.0705266	0.528113	-0.612235	0	0
H2O2e	1	-0.0705266	0.528113	-0.612235	0	0
H2Oe	8	-1.5806	0.943015	-1.17112	0.806898	0
GLCNT	1	1.32102	0.0932478	0.975714	0	0
GLAC	12	1.92808	0.0269224	0.104274	1.28601	2
GALOL	4	0.307527	0.379221	-0.356444	0.357316	0
GAL1P	2	0.871223	0.191816	0.17146	0.88634	0
UTP	5	-0.376858	0.646861	-0.724767	0.633924	0
UDPGAL	4	-0.165419	0.565693	-0.626627	0.841614	0
UDPG	7	-0.51285	0.695972	-0.753801	0.94332	0
G1P	2	-0.162941	0.564717	-0.663532	0.294518	0
MELI	3	0.687663	0.245832	-0.0785656	1.16341	0
GALN14LAC	1	2.13086	0.0165505	1.89986	0	1
GALNT	1	0.136404	0.445751	-0.376098	0	0
2D3DGALT	1	0.136404	0.445751	-0.376098	0	0
SOR	5	0.621459	0.267149	-0.214559	1.85199	1

SOT	2	-0.652194	0.742862	-1.05856	1.73912	0
MAN6P	4	-1.44736	0.926102	-1.35897	0.387058	0
MAN	3	0.796968	0.212735	-0.00648105	2.35283	1
FRU	6	0.128461	0.448892	-0.472254	1.75983	1
MNT	3	-0.615567	0.73091	-0.938027	1.24734	0
F26P	1	-0.596939	0.724726	-1.21295	0	0
GDPMAN	1	0.713274	0.237838	0.282192	0	0
IDOL	2	-0.367268	0.64329	-0.828508	0.118174	0
UDP	14	-0.40747	0.658169	-0.656865	0.848223	0
TRE6P	2	-1.05877	0.855147	-1.38683	0.979481	0
TRE	3	-1.19226	0.883421	-1.31835	0.549941	0
MLT	4	-0.790931	0.785508	-0.983969	0.943719	0
MLTe	4	-0.790931	0.785508	-0.983969	0.943719	0
LACT	2	-0.881023	0.810847	-1.24332	0.264722	0
LACTe	2	-0.881023	0.810847	-1.24332	0.264722	0
GLACe	3	0.915317	0.180013	0.0715687	2.28513	1
13GLUCAN	2	0.231158	0.408596	-0.345334	1.02883	0
GA6P	3	-0.695897	0.756753	-0.991003	0.667772	0
NAGA6P	2	0.273923	0.392072	-0.310805	0.0828462	0

NAGA1P	2	0.212711	0.415776	-0.360228	0.0129516	0
UDPNAG	8	0.47854	0.316133	-0.338808	0.699151	0
CHIT	8	0.387106	0.349339	-0.375766	0.707655	0
NAG	1	-0.100758	0.540129	-0.646734	0	0
GLCN	2	-1.02602	0.84756	-1.36039	0.270455	0
13GLUCANe	6	-0.552176	0.709586	-0.789849	0.915651	0
GLYCOGEN	1	-0.297976	0.617139	-0.871788	0	0
STARe	1	-0.0127258	0.505077	-0.546276	0	0
GLYCOGENe	1	-0.0127258	0.505077	-0.546276	0	0
CELLUe	4	-1.66906	0.952447	-1.48562	0.85924	0
CELLOBe	4	-1.66906	0.952447	-1.48562	0.85924	0
CELLOTe	1	-1.57953	0.942893	-2.33422	0	0
MANNANe	6	-0.91657	0.820316	-0.959881	0.78176	0
MANe	7	0.220731	0.412651	-0.436849	1.55699	1
PECTATEe	1	-0.312436	0.622645	-0.888288	0	0
GALUNTe	1	-0.312436	0.622645	-0.888288	0	0
XYLANe	3	-1.13375	0.871551	-1.27976	0.323599	0
XYLe	3	-1.13375	0.871551	-1.27976	0.323599	0
H+_PO_mit	2	-0.429146	0.666091	-0.878469	1.52121	0

H+_PO	2	-0.429146	0.666091	-0.878469	1.52121	0
FERIm	2	-1.41741	0.921819	-1.6764	0.161421	0
FEROm	2	-1.41741	0.921819	-1.6764	0.161421	0
Ca	1	0.638786	0.261481	0.197191	0	0
Cam	1	0.638786	0.261481	0.197191	0	0
LLACm	2	-1.38178	0.91648	-1.64763	0.120728	0
GLUm	8	0.1821	0.427752	-0.458631	0.455736	0
ASPm	5	0.527227	0.299018	-0.262717	0.563902	0
ALAm	1	-0.324552	0.62724	-0.902115	0	0
ASN	3	0.554635	0.289572	-0.166296	1.46774	0
SAM	7	-0.904832	0.817223	-0.923161	0.71645	0
HCYS	6	0.82328	0.205174	-0.14804	0.337373	0
SAH	5	-0.573692	0.716912	-0.825362	0.947521	0
MET	2	-0.290274	0.614197	-0.766343	0.60378	0
TRNAm	4	0.0234241	0.490656	-0.518745	0.652376	0
ASPTRNAm	3	0.19318	0.423409	-0.40467	0.748541	0
TRNA	2	0.0417032	0.483368	-0.498301	1.03345	0
ASPTRNA	2	0.0417032	0.483368	-0.498301	1.03345	0
NH3	16	-1.49228	0.932187	-0.959094	0.793885	0

NAGLUm	2	-0.290117	0.614137	-0.766216	0.17658	0
NAGLUPm	1	-0.31488	0.623573	-0.891077	0	0
NAGLUSm	2	-0.66334	0.746444	-1.06756	0.249584	0
NAORNm	2	-0.508696	0.694517	-0.942699	0.426164	0
ORNm	1	-0.0960447	0.538257	-0.641355	0	0
CAP	4	1.68726	0.0457763	0.431769	0.629665	0
ORN	3	4.38856	1.85048e-005	2.36212	3.15474	2
CITR	1	1.9313	0.026723	1.67213	0	1
GLUGSAL	3	1.48027	0.0694003	0.444148	2.83657	1
ARGSUCC	2	2.33651	0.00973224	1.35455	0.449132	1
ARG	3	4.2356	3.70442e-005	2.26125	2.03587	1
PTRSC	1	-0.284122	0.611842	-0.855978	0	0
DSAM	2	0.157687	0.437352	-0.404655	0.638267	0
SPRMD	2	-0.379804	0.647954	-0.83863	0.0245342	0
5MTA	1	-0.284122	0.611842	-0.855978	0	0
SPRM	1	-0.284122	0.611842	-0.855978	0	0
GBAD	2	-0.50378	0.692792	-0.93873	0.541458	0
GBAT	2	-0.50378	0.692792	-0.93873	0.541458	0
UREA	3	1.84755	0.0323335	0.686365	3.40851	1

ATRNA	1	1.46095	0.0720149	1.13539	0	0
ALTRNA	1	1.46095	0.0720149	1.13539	0	0
DAPRP	1	-0.253717	0.600143	-0.821281	0	0
SLF	1	-0.583591	0.720252	-1.19772	0	0
APS	2	-0.00236378	0.500943	-0.533881	0.938803	0
PAPS	2	0.239523	0.40535	-0.33858	0.662605	0
SER	10	-1.01314	0.844504	-0.89862	0.798456	0
ASER	5	-0.0185332	0.507393	-0.541638	0.450123	0
H2S	4	0.431845	0.332927	-0.285424	0.242954	0
RTHIO	3	-0.951532	0.829333	-1.15959	0.894878	0
OTHIO	3	-0.951532	0.829333	-1.15959	0.894878	0
H2SO3	2	0.044878	0.482102	-0.495738	0.440349	0
GLUGSALm	2	0.172905	0.431363	-0.392368	0.225297	0
P5Cm	3	1.73697	0.0411967	0.613433	1.74937	1
PHP	1	0.956507	0.169408	0.559756	0	0
GLYm	1	0.233698	0.40761	-0.265072	0	0
GLY	7	0.309832	0.378344	-0.398352	0.59691	0
GLX	1	-0.806777	0.790102	-1.4524	0	0
BASP	2	2.12651	0.0167304	1.18499	0.577242	0

ASPSA	2	0.902852	0.183302	0.196998	0.819989	0
HSER	3	0.215526	0.414679	-0.389933	0.853172	0
PHSER	2	2.0397	0.0206901	1.1149	0.926648	1
THR	6	-0.535927	0.703996	-0.782267	1.37065	1
LLCT	4	0.545684	0.292642	-0.22039	0.305729	0
OBUT	3	-0.965403	0.832829	-1.16874	1.39417	0
THRm	3	-0.246853	0.597489	-0.694865	1.21301	0
NH3m	3	-0.246853	0.597489	-0.694865	1.21301	0
OBUTm	4	0.26925	0.393868	-0.37831	1.17548	0
PRPP	7	1.08781	0.13834	-0.0622181	1.23561	0
PRBATP	3	0.132018	0.447485	-0.445005	2.27664	0
PRBAMP	2	-1.15405	0.87576	-1.46376	2.03444	0
PRFP	3	-0.288446	0.613497	-0.722296	1.92842	0
PRLP	2	1.94956	0.025614	1.04212	0.398077	0
DIMGP	2	1.95109	0.0255234	1.04335	0.39634	0
IMACP	2	2.07675	0.0189124	1.14481	0.539829	0
HISOLP	2	0.851455	0.197258	0.1555	1.93893	0
HISOL	3	-1.28729	0.901004	-1.38102	1.44569	0
HIS	4	-1.1034	0.865074	-1.16248	1.30606	0

AICAR	4	1.28285	0.0997717	0.200738	0.74997	0
HTRNA	1	-0.774768	0.780762	-1.41588	0	0
HHTRNA	1	-0.774768	0.780762	-1.41588	0	0
MHIS	1	0.197389	0.421761	-0.306505	0	0
OICAPm	1	0.468418	0.319743	0.00277732	0	0
LEUm	1	0.468418	0.319743	0.00277732	0	0
OMVALm	2	0.794825	0.213358	0.109776	0.151319	0
ILEm	1	0.468418	0.319743	0.00277732	0	0
OMVAL	1	0.468418	0.319743	0.00277732	0	0
ILE	1	0.468418	0.319743	0.00277732	0	0
VAL	1	0.468418	0.319743	0.00277732	0	0
OICAP	3	0.614568	0.26942	-0.126771	0.261333	0
LEU	1	0.468418	0.319743	0.00277732	0	0
ABUTm	2	0.860534	0.194748	0.16283	0.577742	0
ACLACm	2	0.860534	0.194748	0.16283	0.577742	0
DHVALm	2	0.640954	0.260776	-0.0144604	0.327016	0
DHMVAm	2	0.640954	0.260776	-0.0144604	0.327016	0
OIVALm	2	1.06763	0.142844	0.330041	0.160182	0
IPPMALm	1	0.85446	0.196425	0.443307	0	0

CBHCAP	1	1.6953	0.0450095	1.40282	0	0
IPPMAL	3	1.32221	0.0930486	0.33991	0.950286	0
PPMAL	1	1.6953	0.0450095	1.40282	0	0
HACNm	1	1.01736	0.154492	0.629194	0	0
HICITm	2	0.989684	0.161164	0.267107	0.512069	0
OXAm	1	0.382751	0.350952	-0.0949809	0	0
MICIT	1	-0.941536	0.826785	-1.60618	0	0
AKA	1	-0.271697	0.607073	-0.8418	0	0
AMA	2	0.985165	0.162272	0.263458	1.56307	0
AMASA	2	1.55628	0.0598206	0.724583	0.910942	0
SACP	2	0.683827	0.247042	0.0201549	0.085269	0
LYS	3	0.838325	0.200924	0.0207938	0.45222	0
LTRNA	2	0.722352	0.235039	0.0512604	0.635166	0
LLTRNA	2	0.722352	0.235039	0.0512604	0.635166	0
LYSm	2	0.722352	0.235039	0.0512604	0.635166	0
LTRNAm	2	0.722352	0.235039	0.0512604	0.635166	0
LLTRNAm	2	0.722352	0.235039	0.0512604	0.635166	0
ADN	6	1.59703	0.0551294	0.213003	1.15035	1
MTHPTGLU	1	0.168558	0.433072	-0.339406	0	0

THPTGLU	1	0.168558	0.433072	-0.339406	0	0
OAHSER	1	-0.626464	0.734495	-1.24664	0	0
OSLHSER	1	0.847295	0.198415	0.43513	0	0
CALH	1	-0.19328	0.57663	-0.752314	0	0
DPTH	1	-0.19328	0.57663	-0.752314	0	0
3DDAH7P	2	2.37754	0.00871431	1.38767	0.100898	0
DQT	1	0.757677	0.224322	0.332863	0	0
QT	1	0.757677	0.224322	0.332863	0	0
3PSME	1	1.7039	0.0441998	1.41264	0	0
PHEN	2	0.559037	0.288068	-0.080601	0.444882	0
PHPYR	1	-0.0333623	0.513307	-0.569826	0	0
PHE	1	-0.0333623	0.513307	-0.569826	0	0
4HPP	4	1.0785	0.140406	0.0839932	0.544517	0
TYR	3	0.858348	0.19535	0.0339983	0.655555	0
AN	1	0.876377	0.190412	0.468317	0	0
NPRAN	1	-0.446081	0.672231	-1.0408	0	0
CPAD5P	1	-0.446081	0.672231	-1.0408	0	0
IGP	1	-0.446081	0.672231	-1.0408	0	0
KYN	1	-0.611062	0.729421	-1.22906	0	0

HKYN	1	-0.611062	0.729421	-1.22906	0	0
TRPm	1	-0.288497	0.613517	-0.860971	0	0
TRPTRNAm	1	-0.288497	0.613517	-0.860971	0	0
PAD	1	-0.0211248	0.508427	-0.555861	0	0
PAC	1	-0.0211248	0.508427	-0.555861	0	0
IAD	1	-0.0211248	0.508427	-0.555861	0	0
IAC	1	-0.0211248	0.508427	-0.555861	0	0
ASPERMD	1	-0.156394	0.562139	-0.710223	0	0
APRUT	1	-0.156394	0.562139	-0.710223	0	0
APROA	1	-0.156394	0.562139	-0.710223	0	0
GABAL	1	-0.156394	0.562139	-0.710223	0	0
ASPRM	1	-0.156394	0.562139	-0.710223	0	0
GLUP	2	-0.466403	0.679536	-0.90855	0.330896	0
P5C	1	-0.0889399	0.535435	-0.633248	0	0
PRO	1	-0.0889399	0.535435	-0.633248	0	0
PHC	1	-0.0889399	0.535435	-0.633248	0	0
HPRO	1	-0.0889399	0.535435	-0.633248	0	0
PROm	1	2.76634	0.00283446	2.62504	0	1
GABALm	1	-1.05267	0.853753	-1.733	0	0

GABAm	1	-1.05267	0.853753	-1.733	0	0
LACALm	1	-1.05267	0.853753	-1.733	0	0
APROP	1	-1.14713	0.874336	-1.84079	0	0
TCOA	1	-0.161862	0.564293	-0.716462	0	0
GLP	1	-0.161862	0.564293	-0.716462	0	0
TGLP	1	-0.161862	0.564293	-0.716462	0	0
PEPD	1	-0.968109	0.833505	-1.6365	0	0
APEP	1	-0.968109	0.833505	-1.6365	0	0
GC	2	-0.545932	0.707444	-0.972763	0.362464	0
OGT	1	0.122808	0.45113	-0.391613	0	0
cAMP	2	-0.27381	0.607885	-0.753049	0.690104	0
GMP	6	1.63166	0.0513753	0.229163	1.164	1
DGMP	2	2.23597	0.0126769	1.27337	1.46992	1
DGDP	2	-0.793603	0.786287	-1.17273	0.95899	0
DATP	1	-1.15594	0.876147	-1.85084	0	0
DADP	4	-0.176389	0.570006	-0.632894	0.921108	0
PRAM	1	1.10345	0.134915	0.727444	0	0
GAR	1	1.10345	0.134915	0.727444	0	0
FGAR	1	0.645604	0.259268	0.204972	0	0

FGAM	2	1.23628	0.108178	0.466208	0.369444	0
AIR	2	2.24102	0.0125124	1.27745	0.777822	1
CAIR	2	2.57371	0.00503075	1.54606	0.397943	1
SAICAR	3	1.26433	0.103055	0.30174	0.835731	0
PRFICA	1	0.324725	0.372695	-0.161197	0	0
IMP	8	1.0177	0.154409	-0.120876	1.22595	1
ASUC	3	0.653946	0.256573	-0.100802	0.147315	0
XMP	5	1.36513	0.0861056	0.165509	1.50236	1
cdAMP	1	-0.621545	0.73288	-1.24103	0	0
DAMP	5	1.47942	0.0695135	0.22392	1.33887	1
cIMP	1	-0.621545	0.73288	-1.24103	0	0
cGMP	1	-0.621545	0.73288	-1.24103	0	0
cCMP	1	-0.621545	0.73288	-1.24103	0	0
ATN	1	-1.30701	0.904396	-2.02324	0	0
ATT	2	-1.54055	0.938287	-1.77583	0.349898	0
UGC	2	-1.18705	0.882396	-1.49041	0.0537489	0
CAASP	3	1.5671	0.0585454	0.501411	0.752075	0
DOROA	1	1.59336	0.0555392	1.2865	0	0
OROA	2	0.952735	0.170362	0.237274	1.48383	0

OMP	2	-0.204659	0.581081	-0.697217	0.162263	0
UMP	6	0.429719	0.3337	-0.331682	1.46726	1
URA	6	0.0320496	0.487216	-0.517241	0.683654	0
CYTS	2	-0.839441	0.799389	-1.20975	0.881003	0
URI	4	1.48589	0.0686542	0.316728	1.40233	1
CYTD	4	1.48589	0.0686542	0.316728	1.40233	1
DU	4	1.68496	0.045998	0.430454	1.29157	1
DR1P	1	0.130512	0.448081	-0.382822	0	0
DT	3	1.90053	0.0286816	0.721304	1.41232	1
THY	1	0.130512	0.448081	-0.382822	0	0
DC	3	1.87057	0.0307022	0.701546	1.43571	1
DTMP	4	1.26992	0.102057	0.193347	1.64097	1
DTDP	2	-1.52633	0.936536	-1.76434	0.12233	0
OTHIOm	1	-1.44178	0.925318	-2.17704	0	0
RTHIOm	1	-1.44178	0.925318	-2.17704	0	0
DUTP	1	-1.15594	0.876147	-1.85084	0	0
DUMP	4	1.43073	0.076254	0.285216	1.50603	1
DCMP	3	1.43171	0.0761132	0.412123	1.81812	1
DCDP	3	-1.04103	0.851069	-1.21861	0.682748	0

CDP	4	-0.848147	0.801822	-1.01665	0.688412	0
PURI5P	3	0.64122	0.26069	-0.109194	0.276527	0
AD	2	-0.260613	0.602805	-0.742394	0.508513	0
INS	4	1.82738	0.0338212	0.511815	1.22691	1
DA	3	1.90053	0.0286816	0.721304	1.41232	1
DIN	1	0.130512	0.448081	-0.382822	0	0
HYXN	2	0.330885	0.370366	-0.264813	0.166889	0
DG	3	1.90053	0.0286816	0.721304	1.41232	1
GN	3	-0.0178853	0.507135	-0.543865	0.497529	0
GSN	5	1.41121	0.0790911	0.189059	1.28446	1
XAN	2	0.330885	0.370366	-0.264813	0.166889	0
XTSINE	3	1.90053	0.0286816	0.721304	1.41232	1
ITP	2	-0.841894	0.800076	-1.21173	0.903849	0
IDP	2	-0.841894	0.800076	-1.21173	0.903849	0
ITPm	1	0.80468	0.210502	0.386501	0	0
IDPm	1	0.80468	0.210502	0.386501	0	0
DGTP	1	-1.15594	0.876147	-1.85084	0	0
DUDP	3	-1.04103	0.851069	-1.21861	0.682748	0
DCTP	1	-1.15594	0.876147	-1.85084	0	0

DTTP	1	-1.15594	0.876147	-1.85084	0	0
LCCA	2	-0.173086	0.568708	-0.671724	0.875959	0
ACOA	4	-0.308172	0.621024	-0.708179	0.67575	0
HACOA	1	-1.30615	0.904249	-2.02226	0	0
OACOA	3	-0.968086	0.833499	-1.17051	0.918016	0
AACCOA	1	0.292345	0.385011	-0.198147	0	0
AACCOAm	1	0.292345	0.385011	-0.198147	0	0
ACACPm	1	-1.30364	0.903822	-2.0194	0	0
MALACPm	1	-1.30364	0.903822	-2.0194	0	0
ACPm	1	-1.30364	0.903822	-2.0194	0	0
MALCOA	2	-0.882101	0.811139	-1.24419	0.0657237	0
MALACP	1	-0.665042	0.746988	-1.29066	0	0
ACACP	1	-0.692152	0.755579	-1.3216	0	0
3OACPm	1	-1.30364	0.903822	-2.0194	0	0
C120ACP	4	-0.0809104	0.532243	-0.578349	0.526886	0
C140ACP	4	-0.0809104	0.532243	-0.578349	0.526886	0
C141ACP	4	-0.0809104	0.532243	-0.578349	0.526886	0
C160ACP	4	-0.0809104	0.532243	-0.578349	0.526886	0
C161ACP	4	-0.0809104	0.532243	-0.578349	0.526886	0

C180ACP	4	-0.0809104	0.532243	-0.578349	0.526886	0
C181ACP	4	-0.0809104	0.532243	-0.578349	0.526886	0
C182ACP	4	-0.0809104	0.532243	-0.578349	0.526886	0
3HPACP	1	-0.692152	0.755579	-1.3216	0	0
2HDACP	1	-0.692152	0.755579	-1.3216	0	0
AACP	1	-0.692152	0.755579	-1.3216	0	0
23DAACP	1	-0.692152	0.755579	-1.3216	0	0
C150ACP	2	0.323715	0.373077	-0.270602	0.151626	0
C162ACP	4	-0.0809104	0.532243	-0.578349	0.526886	0
C170ACP	4	-0.0809104	0.532243	-0.578349	0.526886	0
C183ACP	4	-0.0809104	0.532243	-0.578349	0.526886	0
C200ACP	4	-0.0809104	0.532243	-0.578349	0.526886	0
AGL3P	2	-0.438593	0.669522	-0.886097	0.656508	0
AT3P2	1	-0.717319	0.763411	-1.35032	0	0
PA	4	-0.279183	0.609948	-0.691618	1.00612	0
PAm	1	0.515977	0.302935	0.0570492	0	0
CTPm	2	-0.45197	0.674355	-0.896897	1.34908	0
CDPDGm	2	1.27333	0.10145	0.496128	0.620951	0
CDPDG	2	-0.183905	0.572956	-0.680459	1.04299	0

PS	1	-1.4869	0.931479	-2.22852	0	0
CMPm	2	0.426541	0.334857	-0.18758	1.58786	0
PSm	1	0.0912982	0.463628	-0.42757	0	0
PE	2	-1.19581	0.884114	-1.49748	1.03385	0
PEm	1	0.0912982	0.463628	-0.42757	0	0
PC	1	-0.205656	0.58147	-0.766438	0	0
CHO	1	0.757677	0.224322	0.332863	0	0
PCHO	2	0.398166	0.345254	-0.21049	0.768416	0
CDPCHO	2	-0.282592	0.611255	-0.76014	0.00890704	0
DAGLY	7	-0.88593	0.812172	-0.914994	0.837756	0
PETHM	1	-0.142204	0.55654	-0.694029	0	0
CDPETN	1	-0.205656	0.58147	-0.766438	0	0
MI1P	1	0.00907351	0.49638	-0.5214	0	0
MYOI	1	-0.776602	0.781303	-1.41797	0	0
PINS	5	-0.306895	0.620538	-0.689011	0.902972	0
PINSP	3	0.581138	0.280574	-0.148818	0.727888	0
PINS4P	3	-1.14784	0.874482	-1.28905	0.451039	0
D45PI	3	-1.32247	0.906994	-1.40422	0.682432	0
TPI	2	-1.01047	0.843865	-1.34784	0.955172	0

GL3Pm	1	1.28552	0.0993054	0.935206	0	0
PGPm	2	0.543591	0.293362	-0.0930729	1.45421	0
PGm	1	-0.516673	0.697308	-1.12135	0	0
DGPP	1	0.264437	0.395722	-0.229995	0	0
CDPm	2	-1.29876	0.902987	-1.5806	0.382175	0
PALCOA	2	-0.780733	0.78252	-1.16234	1.01858	0
DHSPH	3	-0.478094	0.683708	-0.847365	0.903542	0
SPH	4	-0.230062	0.590978	-0.663556	0.726284	0
PSPH	3	-0.498356	0.690883	-0.860728	0.747864	0
C260COA	1	0.267465	0.394556	-0.226539	0	0
CER2	2	-0.217361	0.586037	-0.707472	0.680142	0
CER3	1	-0.575432	0.717501	-1.1884	0	0
IPC	1	0.713274	0.237838	0.282192	0	0
MIPC	1	0.713274	0.237838	0.282192	0	0
DHSP	3	-0.436912	0.668913	-0.820207	0.809531	0
PHSP	1	-1.01097	0.843985	-1.68542	0	0
C16A	1	-0.142204	0.55654	-0.694029	0	0
H3MCOA	1	-0.210765	0.583465	-0.772268	0	0
MVL	2	-0.0737573	0.529398	-0.591525	0.255609	0

PMVL	1	0.106009	0.457787	-0.410783	0	0
IPPP	2	-0.0623638	0.524863	-0.582326	0.00421095	0
DMPP	2	-0.0623638	0.524863	-0.582326	0.00421095	0
GPP	1	-0.0417075	0.516634	-0.579349	0	0
FPP	1	-0.0417075	0.516634	-0.579349	0	0
LNST	1	0.202452	0.419782	-0.300728	0	0
IGST	2	0.207855	0.417671	-0.364149	0.0896911	0
DMZYMST	1	0.0912982	0.463628	-0.42757	0	0
IMZYMST	1	-0.121344	0.548291	-0.670226	0	0
IIMZYMST	2	-0.527902	0.701216	-0.958206	0.407266	0
MZYMST	1	-0.626067	0.734365	-1.24619	0	0
IZYMST	1	-0.121344	0.548291	-0.670226	0	0
IIZYMST	2	-0.527902	0.701216	-0.958206	0.407266	0
ZYMST	2	-0.873971	0.808933	-1.23762	0.01211	0
FEST	2	-0.787454	0.784492	-1.16777	0.0866817	0
EPST	2	-0.358874	0.640155	-0.821731	0.402692	0
ERTROL	2	-0.394634	0.653443	-0.850604	0.443524	0
ERTEOL	2	-0.326877	0.62812	-0.795896	0.520892	0
ERGOST	1	0.0912982	0.463628	-0.42757	0	0

TAGLY	2	0.323715	0.373077	-0.270602	0.151626	0
MAGLY	1	0.134897	0.446347	-0.377818	0	0
PHACAL	1	-1.05267	0.853753	-1.733	0	0
PHAC	1	-1.05267	0.853753	-1.733	0	0
AVF	1	-0.363808	0.641999	-0.946911	0	0
VHA	1	-0.363808	0.641999	-0.946911	0	0
ST	1	-1.43707	0.92465	-2.17165	0	0
DHST	1	-1.43707	0.92465	-2.17165	0	0
OMST	3	-0.91346	0.8195	-1.13448	0.911478	0
DHOMST	3	-0.91346	0.8195	-1.13448	0.911478	0
AFB1	2	-0.103944	0.541393	-0.615898	0.219101	0
AFG1	2	-0.103944	0.541393	-0.615898	0.219101	0
AFB2	2	-0.103944	0.541393	-0.615898	0.219101	0
AFG2	2	-0.103944	0.541393	-0.615898	0.219101	0
HNO3	1	0.530513	0.297878	0.0736361	0	0
HNO2	1	0.530513	0.297878	0.0736361	0	0
UREAC	1	-0.431253	0.666858	-1.02388	0	0
ACNL	1	-1.14713	0.874336	-1.84079	0	0
INAC	1	-1.14713	0.874336	-1.84079	0	0

FRUe	1	2.83321	0.00230414	2.70134	0	1
SORe	1	2.83321	0.00230414	2.70134	0	1

#Results for Down-regulated only genes

#Feature	Number of neighbors	Z-score	P-value	Average Z	StdDev Z	Significance count
DGLCe	3	-0.735483	0.768977	-1.01711	0.354846	0
GLCe	8	1.36583	0.0859959	0.0198388	1.4245	1
bDGLCe	2	-0.802334	0.78882	-1.17978	1.19283	0
GLC	5	0.274358	0.391905	-0.391951	1.13528	0
bDGLC	1	-0.785935	0.784047	-1.42862	0	0
ATP	21	-0.901282	0.816281	-0.757383	1.52677	3
ADP	12	-0.339325	0.632817	-0.644313	1.44936	2
G6P	5	-1.89043	0.970649	-1.4983	0.542508	0
bDG6P	2	-1.47814	0.930315	-1.72544	0.419765	0
H2O	43	1.48773	0.0684109	-0.272617	1.60681	5
PI	16	-0.053179	0.521205	-0.547522	1.44261	1
F6P	6	-0.508713	0.694523	-0.769569	1.15521	0
FDP	1	-0.264587	0.604336	-0.833686	0	0
S7P	3	-0.465731	0.679296	-0.839212	0.280325	0

S17P	1	-0.264587	0.604336	-0.833686	0	0
T3P2	2	-0.801688	0.788633	-1.17926	0.617184	0
T3P1	3	0.649666	0.257954	-0.103624	1.30922	0
E4P	2	-0.383948	0.649491	-0.841976	0.396381	0
NAD	31	0.00706232	0.497183	-0.530914	1.30232	1
13PDG	2	1.19962	0.115143	0.436615	1.32436	0
NADH	31	0.00706232	0.497183	-0.530914	1.30232	1
3PG	2	1.67545	0.0469231	0.820799	1.86768	1
2PG	1	0.02796	0.488847	-0.499848	0	0
23PDG	1	0.02796	0.488847	-0.499848	0	0
PEP	1	2.70562	0.00340881	2.55575	0	1
PYR	6	-0.217609	0.586133	-0.633735	0.829373	0
CO2	18	2.23868	0.0125885	0.0713679	1.37591	2
OA	6	0.61217	0.270213	-0.246548	1.66045	1
ATPm	4	-0.927788	0.823241	-1.06215	0.691146	0
PYRm	3	0.050678	0.479791	-0.498648	1.02992	0
CO2m	4	0.086261	0.465629	-0.482848	1.01966	0
ADPm	3	-1.00188	0.841799	-1.19279	0.783658	0
PIm	4	-1.10393	0.865189	-1.16278	0.724557	0

OAm	4	-0.185526	0.573592	-0.638114	1.1242	0
GTP	4	-0.441772	0.670673	-0.784502	2.2439	1
GDP	1	2.70562	0.00340881	2.55575	0	1
NADP	24	2.12083	0.0169682	-0.0369299	1.6885	3
D6PGL	1	-0.660161	0.745425	-1.28509	0	0
NADPH	24	2.12083	0.0169682	-0.0369299	1.6885	3
D6PGC	5	0.0961668	0.461694	-0.483019	0.801964	0
RL5P	2	-0.727718	0.766607	-1.11954	0.784117	0
XUL5P	2	-0.383948	0.649491	-0.841976	0.396381	0
R5P	4	-1.46232	0.928173	-1.36752	0.656715	0
ACTP	1	-0.100758	0.540129	-0.646734	0	0
ACCOAm	5	-1.4725	0.929557	-1.28471	0.53408	0
H2Om	8	-0.631566	0.736165	-0.787518	2.33251	1
CITm	1	-1.32382	0.907218	-2.04242	0	0
COAm	5	-1.4725	0.929557	-1.28471	0.53408	0
NADm	10	0.391002	0.347898	-0.390877	2.03339	1
AKGm	2	-0.600337	0.725859	-1.01669	0.974042	0
NADHm	10	0.391002	0.347898	-0.390877	2.03339	1
AKG	3	0.935987	0.17464	0.0851999	0.480443	0

NADPm	6	0.0232384	0.49073	-0.521352	2.65964	1
NADPHm	6	0.0232384	0.49073	-0.521352	2.65964	1
ICITg	1	-0.67379	0.749778	-1.30064	0	0
LIPOm	1	-1.02852	0.848147	-1.70544	0	0
SUCDLIP	1	-1.02852	0.848147	-1.70544	0	0
FUMm	1	-1.67532	0.953065	-2.44354	0	0
FADH2m	2	-0.244899	0.596733	-0.729706	0.535473	0
FADm	2	-0.244899	0.596733	-0.729706	0.535473	0
FUM	2	-0.808733	0.790666	-1.18495	1.77991	0
SUCC	2	-0.343601	0.634427	-0.8094	0.130512	0
MALm	2	-0.446338	0.672324	-0.89235	2.19371	0
MAL	3	-0.844635	0.800843	-1.08909	1.58818	0
MALg	1	-0.847772	0.801718	-1.49918	0	0
SUCCg	1	-0.67379	0.749778	-1.30064	0	0
GLXg	2	-1.07497	0.858806	-1.39991	0.140388	0
ACCOAg	2	-1.19078	0.883129	-1.49342	0.00815996	0
H2Og	1	-0.847772	0.801718	-1.49918	0	0
COAg	2	-1.19078	0.883129	-1.49342	0.00815996	0
Hm	6	1.04994	0.146873	-0.0422771	2.55004	1

COA	15	-2.41413	0.992114	-1.24533	0.915341	0
ACCOA	11	-0.941781	0.826848	-0.857006	1.02429	0
CAR	1	-0.540026	0.705411	-1.148	0	0
ACAR	1	-0.540026	0.705411	-1.148	0	0
ACARm	2	-0.9733	0.834798	-1.31782	0.240166	0
CARm	2	-0.9733	0.834798	-1.31782	0.240166	0
ACARg	2	-0.9733	0.834798	-1.31782	0.240166	0
CARg	2	-0.9733	0.834798	-1.31782	0.240166	0
OXAL	2	0.69987	0.242004	0.0331086	0.744792	0
AC	9	0.7213	0.235363	-0.257344	1.9274	1
FOR	6	0.396576	0.34584	-0.347147	0.588945	0
FORm	2	-0.662996	0.746333	-1.06728	1.44735	0
METHOL	2	-0.955527	0.830344	-1.30347	1.22651	0
FALD	5	-1.01914	0.845932	-1.05302	0.685967	0
ACAL	9	0.202511	0.419759	-0.455069	2.09001	1
RGT	4	-1.02197	0.846604	-1.11596	0.629224	0
FGT	1	-0.374511	0.645988	-0.959124	0	0
H+	15	3.696	0.00010951	0.559325	1.86101	3
MTHGXL	2	-0.837454	0.798831	-1.20814	0.902714	0

LACAL	4	-1.44114	0.925227	-1.35542	0.628457	0
LAC	4	-1.03205	0.848975	-1.12171	0.554444	0
LGT	2	-0.258556	0.602011	-0.740733	0.2417	0
LLAC	1	-0.833223	0.79764	-1.48258	0	0
PROP	1	-0.121344	0.548291	-0.670226	0	0
AMP	11	-1.17885	0.880771	-0.938748	1.55652	1
PPI	15	-1.70789	0.956172	-1.03674	1.36787	1
PROPCOA	2	-1.02098	0.846368	-1.35632	0.970287	0
2MCIT	1	-1.32382	0.907218	-2.04242	0	0
GLU	10	1.58402	0.0565951	0.0405211	1.80202	2
GABA	3	-0.644196	0.740276	-0.956907	1.09847	0
SUCCSAL	3	-0.0260132	0.510377	-0.549225	0.459988	0
METTHF	1	-0.304648	0.619683	-0.879401	0	0
METHF	1	-1.36614	0.914052	-2.09071	0	0
METTHFm	2	-1.18042	0.881084	-1.48506	0.856525	0
METHFm	1	-1.36614	0.914052	-2.09071	0	0
FTHFm	1	-1.36614	0.914052	-2.09071	0	0
FTHF	1	-1.36614	0.914052	-2.09071	0	0
THFm	2	-1.18042	0.881084	-1.48506	0.856525	0

GLN	4	2.62736	0.00430251	0.968825	2.52081	2
THF	2	-0.43305	0.667511	-0.881621	0.00314602	0
THFG	1	-0.308539	0.621164	-0.883841	0	0
PANT	1	-0.998878	0.841073	-1.67162	0	0
bALA	1	-0.998878	0.841073	-1.67162	0	0
PNTO	1	-0.998878	0.841073	-1.67162	0	0
CTP	2	-1.32366	0.907191	-1.6007	0.113475	0
CYS	2	-1.85582	0.96826	-2.03038	0.995441	0
ASP	4	1.05321	0.146123	0.0695461	2.06432	1
ACP	3	0.232836	0.407945	-0.378518	1.25241	0
ALA	3	-0.87542	0.809327	-1.1094	0.197551	0
ETH	2	-0.955527	0.830344	-1.30347	1.22651	0
ETHm	2	-0.955527	0.830344	-1.30347	1.22651	0
ACALm	6	0.630225	0.264274	-0.238123	2.54092	1
ACm	7	0.887802	0.187324	-0.148634	2.20775	1
AMPm	1	-0.121344	0.548291	-0.670226	0	0
PPIm	2	-0.574602	0.71722	-0.995912	0.46059	0
ACTPm	1	-0.100758	0.540129	-0.646734	0	0
GLYN	7	-1.02399	0.84708	-0.974645	0.751942	0

GL	6	-1.36068	0.913193	-1.16711	0.963824	0
GLYAL	5	-1.05598	0.854511	-1.07184	1.04553	0
O2	21	2.32272	0.0100971	0.047635	1.50834	3
H2O2	6	0.472747	0.318197	-0.311604	1.95221	1
GL3P	1	-0.949858	0.828908	-1.61568	0	0
TAR	1	-0.312436	0.622645	-0.888288	0	0
OXGLY	1	-0.312436	0.622645	-0.888288	0	0
G	1	2.34257	0.00957582	2.14145	0	1
E	2	-1.28218	0.90011	-1.56721	0.394909	0
EOL	2	-1.28218	0.90011	-1.56721	0.394909	0
LXUL	3	-0.475359	0.682735	-0.845562	0.337269	0
XOL	1	0.0488137	0.480534	-0.476051	0	0
XUL	1	0.0488137	0.480534	-0.476051	0	0
AOL	1	0.0488137	0.480534	-0.476051	0	0
ARAB	1	-0.662684	0.746233	-1.28797	0	0
ARABLAC	1	-0.662684	0.746233	-1.28797	0	0
LAOL	2	-0.617215	0.731453	-1.03032	0.150643	0
R1P	1	-1.30364	0.903822	-2.0194	0	0
RL	1	-1.00096	0.841577	-1.67399	0	0

O2e	6	0.216117	0.414448	-0.431352	1.92296	1
GLCN15LACe	3	-0.685782	0.753575	-0.984333	0.908861	0
H2O2e	6	0.216117	0.414448	-0.431352	1.92296	1
H2Oe	11	0.50399	0.307134	-0.358497	1.4616	1
GLCNTe	1	-0.0540465	0.521551	-0.593429	0	0
GLCN15LAC	1	-0.0540465	0.521551	-0.593429	0	0
GLCNT	5	0.0850747	0.466101	-0.488688	0.802789	0
GLAC	6	0.188452	0.425261	-0.444261	1.0872	0
GALOL	1	-1.15209	0.875358	-1.84646	0	0
UTP	2	-0.203407	0.580592	-0.696206	1.16568	0
UDPG	3	-0.564149	0.713674	-0.904117	1.12559	0
G1P	2	-0.512378	0.695807	-0.945671	1.51848	0
MELI	1	0.0620293	0.47527	-0.46097	0	0
GALN14LAC	2	-0.255959	0.601009	-0.738637	0.354174	0
GALNT	1	-1.36153	0.913327	-2.08546	0	0
2D3DGALT	1	-1.36153	0.913327	-2.08546	0	0
SOR	3	0.404131	0.343058	-0.265551	0.404465	0
SOT	2	0.041206	0.483566	-0.498703	0.0320341	0
MAN6P	1	-0.785935	0.784047	-1.42862	0	0

MAN	1	-0.785935	0.784047	-1.42862	0	0
FRU	2	-0.520631	0.698688	-0.952335	0.673567	0
MNT6P	1	1.64997	0.0494744	1.3511	0	0
MNT	1	0.0488137	0.480534	-0.476051	0	0
MAN1P	1	-1.28624	0.900821	-1.99954	0	0
GDPMAN	1	-1.28624	0.900821	-1.99954	0	0
IDOL	1	0.641907	0.260467	0.200753	0	0
UDP	3	-1.25898	0.895981	-1.36235	0.691355	0
TRE6P	1	-0.179114	0.571076	-0.736149	0	0
MLT	1	-0.576216	0.717765	-1.1893	0	0
MLTe	1	-0.576216	0.717765	-1.1893	0	0
LACT	2	1.3519	0.0882029	0.559567	1.27652	0
LACTe	2	1.3519	0.0882029	0.559567	1.27652	0
GLACe	2	1.3519	0.0882029	0.559567	1.27652	0
GA6P	1	-0.785935	0.784047	-1.42862	0	0
UDPNAG	1	-0.626464	0.734495	-1.24664	0	0
CHIT	5	-0.112188	0.544663	-0.589502	1.52373	1
NAG	4	0.18714	0.425775	-0.425218	1.70755	1
GLCN	1	-0.785935	0.784047	-1.42862	0	0

13GLUCANe	6	0.735015	0.231165	-0.189226	1.58629	1
GLYCOGEN	1	-1.37801	0.915899	-2.10426	0	0
STARe	1	-0.456651	0.676039	-1.05286	0	0
GLYCOGENe	1	-0.456651	0.676039	-1.05286	0	0
AMYLSe	1	-0.925923	0.822757	-1.58836	0	0
AMYLPe	1	-0.925923	0.822757	-1.58836	0	0
CELLUe	4	-1.13945	0.872742	-1.18307	0.440582	0
CELLOBe	4	-1.13945	0.872742	-1.18307	0.440582	0
CELLOTe	3	-0.735483	0.768977	-1.01711	0.354846	0
ARABINe	5	-0.335563	0.6314	-0.703662	0.250564	0
LARABe	5	-0.335563	0.6314	-0.703662	0.250564	0
XYLANe	3	0.0802496	0.468019	-0.479146	0.227859	0
XYLe	3	0.0802496	0.468019	-0.479146	0.227859	0
FERIm	1	-0.685359	0.753441	-1.31385	0	0
FEROm	1	-0.685359	0.753441	-1.31385	0	0
LLACm	4	1.44707	0.0739384	0.294553	3.0206	1
LACm	1	-0.685359	0.753441	-1.31385	0	0
GLUm	2	-0.916268	0.820237	-1.27178	1.33479	0
ASPm	1	0.178607	0.429123	-0.327939	0	0

ASN	2	1.34348	0.0895588	0.552763	3.44028	1
SAM	5	0.669495	0.25159	-0.190009	1.95577	1
HCYS	2	-1.11857	0.868338	-1.43512	1.44662	0
SAH	3	-1.25587	0.895418	-1.36029	1.00194	0
MET	2	-1.11857	0.868338	-1.43512	1.44662	0
TRNA	1	0.0283507	0.488691	-0.499402	0	0
ASPTRNA	1	0.0283507	0.488691	-0.499402	0	0
NH3	13	1.75247	0.0398469	0.0236264	1.93939	2
CAP	1	3.72501	9.76524e-005	3.71902	0	1
ORN	2	3.51808	0.000217344	2.30855	1.9947	1
CITR	1	3.72501	9.76524e-005	3.71902	0	1
PTRSC	1	1.25299	0.105104	0.898089	0	0
SPRMD	1	-1.73632	0.958747	-2.51315	0	0
SPRM	1	-1.73632	0.958747	-2.51315	0	0
GBAD	1	4.50701	1.06465e-005	4.61138	0	1
GBAT	1	4.50701	1.06465e-005	4.61138	0	1
H2S	1	0.104769	0.45828	-0.412198	0	0
RTHIO	1	-0.866422	0.806871	-1.52047	0	0
OTHIO	1	-0.866422	0.806871	-1.52047	0	0

GLUGSALm	1	-1.47559	0.929973	-2.21561	0	0
P5Cm	1	-1.47559	0.929973	-2.21561	0	0
GLYm	1	-0.304648	0.619683	-0.879401	0	0
GLY	4	-1.21855	0.888493	-1.22826	0.465271	0
GLX	1	-0.6912	0.75528	-1.32051	0	0
THR	2	-1.02128	0.846439	-1.35656	0.697348	0
LLCT	1	-0.696442	0.756924	-1.32649	0	0
OBUT	1	-0.696442	0.756924	-1.32649	0	0
PRPP	2	-1.06471	0.856496	-1.39163	0.226282	0
VAL	3	0.685179	0.246616	-0.0802042	2.77091	1
HCITm	1	-0.0211248	0.508427	-0.555861	0	0
HACNm	1	-0.0211248	0.508427	-0.555861	0	0
AMA	2	-2.32582	0.989986	-2.40986	0.458774	0
ADN	1	0.101804	0.459456	-0.415582	0	0
OAHSER	1	0.104769	0.45828	-0.412198	0	0
METH	1	0.104769	0.45828	-0.412198	0	0
DQT	1	-0.335389	0.631334	-0.914481	0	0
DHSK	1	-0.335389	0.631334	-0.914481	0	0
PHPYR	1	0.515214	0.303202	0.056178	0	0

4HPP	2	2.75884	0.00290035	1.69554	2.86163	1
TYR	1	0.178607	0.429123	-0.327939	0	0
AN	3	-0.830492	0.79687	-1.07977	0.151308	0
NPRAN	1	-0.613305	0.730163	-1.23162	0	0
TRP	2	0.206888	0.418049	-0.36493	0.0682531	0
FKYN	3	0.350986	0.362799	-0.300599	0.121427	0
KYN	3	-0.29488	0.615957	-0.726538	0.486091	0
HKYN	2	-0.584419	0.720531	-1.00384	0.105818	0
HAN	2	-0.584419	0.720531	-1.00384	0.105818	0
CMUSA	1	0.569637	0.284462	0.118282	0	0
AM6SA	2	-0.453996	0.675084	-0.898533	1.43799	0
AMUCO	1	-1.21246	0.887333	-1.91535	0	0
HOMOGEN	2	3.9986	3.18595e-005	2.69653	1.44601	2
MACAC	2	3.02611	0.00123861	1.91134	0.335581	2
FUACAC	2	2.03503	0.0209238	1.11113	1.46724	1
ACTAC	1	0.530513	0.297878	0.0736361	0	0
PAD	2	3.81404	6.83559e-005	2.54752	2.91874	1
PAC	2	3.81404	6.83559e-005	2.54752	2.91874	1
IAD	2	3.81404	6.83559e-005	2.54752	2.91874	1

IAC	2	3.81404	6.83559e-005	2.54752	2.91874	1
ASPERMD	1	-1.73632	0.958747	-2.51315	0	0
ASPRM	1	-1.73632	0.958747	-2.51315	0	0
GABALm	4	1.44707	0.0739384	0.294553	3.0206	1
GABAm	4	1.44707	0.0739384	0.294553	3.0206	1
LACALm	4	1.44707	0.0739384	0.294553	3.0206	1
OGT	1	-1.30701	0.904396	-2.02324	0	0
cAMP	1	0.154853	0.438469	-0.355045	0	0
DATP	1	-0.866422	0.806871	-1.52047	0	0
IMP	1	-1.08222	0.860424	-1.76673	0	0
DUTP	2	-1.05123	0.853424	-1.38075	0.197594	0
DUMP	1	-0.621545	0.73288	-1.24103	0	0
AD	3	-1.08051	0.860042	-1.24465	0.726002	0
INS	1	0.101804	0.459456	-0.415582	0	0
DA	1	0.101804	0.459456	-0.415582	0	0
DIN	1	0.101804	0.459456	-0.415582	0	0
HYXN	1	0.101804	0.459456	-0.415582	0	0
DG	1	-1.4389	0.924911	-2.17375	0	0
GSN	1	-1.4389	0.924911	-2.17375	0	0

DGTP	2	-1.62883	0.948326	-1.84711	0.461941	0
DCTP	1	-0.866422	0.806871	-1.52047	0	0
LCCA	2	-0.604117	0.727117	-1.01974	1.48702	0
ACOA	3	-0.877944	0.810013	-1.11106	1.06331	0
HACOA	2	-0.46383	0.678615	-0.906474	0.719071	0
OACOA	3	-0.763442	0.7774	-1.03555	0.555439	0
AACCOA	2	-0.96251	0.832103	-1.30911	0.0176608	0
AACCOAm	1	-0.692152	0.755579	-1.3216	0	0
MALCOA	4	1.32594	0.0924298	0.225353	0.972303	0
MALACP	1	0.989996	0.161088	0.597972	0	0
ACACP	2	-0.414647	0.6608	-0.866763	1.30643	0
C120ACP	3	0.61202	0.270262	-0.128451	1.0418	0
C140ACP	3	0.61202	0.270262	-0.128451	1.0418	0
C141ACP	3	0.61202	0.270262	-0.128451	1.0418	0
C160ACP	3	0.61202	0.270262	-0.128451	1.0418	0
C161ACP	3	0.61202	0.270262	-0.128451	1.0418	0
C180ACP	3	0.61202	0.270262	-0.128451	1.0418	0
C181ACP	3	0.61202	0.270262	-0.128451	1.0418	0
C182ACP	3	0.61202	0.270262	-0.128451	1.0418	0

3HPACP	2	-0.414647	0.6608	-0.866763	1.30643	0
2HDACP	2	-0.414647	0.6608	-0.866763	1.30643	0
AACP	2	-0.414647	0.6608	-0.866763	1.30643	0
23DAACP	2	-0.414647	0.6608	-0.866763	1.30643	0
C150ACP	3	0.61202	0.270262	-0.128451	1.0418	0
C162ACP	3	0.61202	0.270262	-0.128451	1.0418	0
C170ACP	3	0.61202	0.270262	-0.128451	1.0418	0
C183ACP	3	0.61202	0.270262	-0.128451	1.0418	0
C200ACP	3	0.61202	0.270262	-0.128451	1.0418	0
AGL3P	1	0.469925	0.319204	0.00449693	0	0
AT3P2	1	0.469925	0.319204	0.00449693	0	0
PA	1	-0.311234	0.622188	-0.886916	0	0
CDPDGm	1	-0.244032	0.596397	-0.81023	0	0
PS	2	1.09954	0.135767	0.355805	0.0579161	0
CMPm	1	-0.244032	0.596397	-0.81023	0	0
PE	3	0.916264	0.179764	0.0721929	0.492935	0
PMME	2	-0.346111	0.63537	-0.811426	0.447449	0
PDME	1	-0.522341	0.699284	-1.12782	0	0
PC	1	-0.522341	0.699284	-1.12782	0	0

DAGLY	4	0.374704	0.35394	-0.318067	0.931335	0
PETHM	1	-1.00705	0.843045	-1.68094	0	0
CDPETN	1	-1.00705	0.843045	-1.68094	0	0
MI1P	1	-1.00705	0.843045	-1.68094	0	0
MYOI	1	-1.00705	0.843045	-1.68094	0	0
PINS	2	-1.18529	0.882049	-1.48899	0.244087	0
PINSP	1	-0.990087	0.838934	-1.66159	0	0
PGm	1	-0.244032	0.596397	-0.81023	0	0
CLm	1	-0.244032	0.596397	-0.81023	0	0
DGPP	1	-0.311234	0.622188	-0.886916	0	0
CER3	1	-0.687591	0.754145	-1.31639	0	0
IPC	1	-0.687591	0.754145	-1.31639	0	0
MIPC	1	-0.687591	0.754145	-1.31639	0	0
MIP2C	1	-0.687591	0.754145	-1.31639	0	0
H3MCOA	2	-0.984342	0.837526	-1.32674	0.0425869	0
MVL	1	-0.723046	0.765174	-1.35685	0	0
PMVL	1	-0.907631	0.817963	-1.56749	0	0
PPMVL	2	-1.21276	0.887389	-1.51116	0.0796585	0
IPPP	1	-0.808912	0.790717	-1.45484	0	0

S23E	1	-1.07071	0.857849	-1.75358	0	0
LNST	3	-0.817196	0.793092	-1.071	0.702002	0
IGST	3	-0.826348	0.795697	-1.07703	0.710827	0
DMZYMST	4	-0.645655	0.740749	-0.900976	1.55794	0
IMZYMST	3	-0.119287	0.547476	-0.610738	1.77069	0
MZYMST	3	-0.119287	0.547476	-0.610738	1.77069	0
IZYMST	3	-0.119287	0.547476	-0.610738	1.77069	0
EPST	1	0.0433514	0.482711	-0.482284	0	0
ERTROL	1	0.0433514	0.482711	-0.482284	0	0
TAGLY	3	0.61202	0.270262	-0.128451	1.0418	0
MAGLY	3	0.61202	0.270262	-0.128451	1.0418	0
PHACAL	5	1.52435	0.0637113	0.246878	2.61809	1
PHAC	5	0.71059	0.238669	-0.169007	2.8138	1
PHACCOA	2	-1.88551	0.970319	-2.05435	0.0439914	0
LLDACV	2	-1.7367	0.95878	-1.9342	1.13146	0
IPN	2	-1.33492	0.909048	-1.6098	0.672685	0
PENG	1	-1.36153	0.913327	-2.08546	0	0
NOR	4	2.54076	0.00553055	0.919354	1.46536	1
AVN	5	2.01189	0.0221159	0.496045	1.36775	1

HAVN	4	1.0556	0.145576	0.0709119	0.565162	0
AVF	1	0.760832	0.223379	0.336463	0	0
VHA	1	2.91474	0.00177994	2.79438	0	1
VERB	3	3.13265	0.000866173	1.53387	1.95328	1
VERA	2	3.69471	0.00011007	2.45117	1.60687	1
DMST	2	3.69471	0.00011007	2.45117	1.60687	1
DHDMST	2	3.69471	0.00011007	2.45117	1.60687	1
HNO3	2	-1.16323	0.877632	-1.47118	0.451657	0
HNO2	1	0.974123	0.164998	0.579859	0	0
NH4OH	1	0.974123	0.164998	0.579859	0	0
NH3e	2	0.64517	0.259408	-0.0110566	0.0411292	0
HNO3e	2	-1.16323	0.877632	-1.47118	0.451657	0
