# Supplementary material for: Systems Analysis Unfolds the Relationship between the Phosphoketolase Pathway and Growth in Aspergillus nidulans
Source: PLoS One. 2008 Dec 4;3(12):e3847. doi: 10.1371/journal.pone.0003847 (PMC2585806; doi:10.1371/journal.pone.0003847)
Supplement: Table S7 — Results of reporter feature algorithm for the examination of phosphoketolase over-expression on ethanol (0.35 MB PDF) [file pone.0003847.s007.pdf]

#Total number of ORFs used = 576

#Total number of significantly changed ORFs = 0

#Mean_all = -0.823378 #Std_all= 0.878508

#kmax, imax = 100, 10000

#Feature	Number of neighbors		Z-score	P-value	Average Z	StdDev Z	Significance count
DGLCe	4	-0.549556	0.708688	-1.06534	0.355792	0	
GLCe	19	0.0608106	0.475755	-0.811054	0.686934	0	
bDGLCe	3	0.00571144	0.497721	-0.820328	0.39097	0	
DGLC	2	2.14474	0.0159867	0.51416	1.43461	0	
GLC	20	-0.929184	0.823603	-1.00599	0.612719	0	
bDGLC	7	-0.805561	0.789752	-1.09134	0.635906	0	
ATP	89	-0.448894	0.673246	-0.865104	0.738775	1	
ADP	59	0.313455	0.376967	-0.787518	0.774529	1	
G6P	10	0.265242	0.395411	-0.749489	0.902279	0	
bDG6P	5	-0.606673	0.727966	-1.06223	0.620707	0	
H2O	73	-0.158448	0.562948	-0.839625	1.07524	2	
PI	58	2.13395	0.0164234	-0.577338	1.286	4	
F6P	14	-0.184917	0.573353	-0.866782	0.836351	0	
FDP	3	-1.04762	0.852593	-1.35632	0.990513	0	

S7P	6	-0.713036	0.762088	-1.07961	0.80854	0
S17P	3	-1.04762	0.852593	-1.35632	0.990513	0
T3P2	7	0.430375	0.333461	-0.680073	0.734643	0
T3P1	11	1.26233	0.103415	-0.488416	1.61965	1
E4P	7	-0.33854	0.632522	-0.935936	0.767939	0
NAD	72	-0.241687	0.595489	-0.848348	0.71633	0
13PDG	6	-0.959886	0.831444	-1.16835	0.93944	0
NADH	72	-0.241687	0.595489	-0.848348	0.71633	0
3PG	5	0.2044	0.419021	-0.742754	0.79449	0
2PG	3	1.06832	0.142688	-0.279608	0.882289	0
23PDG	2	0.40362	0.343246	-0.571523	1.02254	0
PEP	6	-0.219454	0.586852	-0.902168	0.974059	0
PYR	17	-0.586557	0.721249	-0.948418	0.701741	0
CO2	41	0.58375	0.279694	-0.743262	0.675281	0
OA	12	0.474639	0.317522	-0.702762	1.28464	1
ATPm	22	-0.596247	0.724495	-0.935073	0.670164	0
PYRm	9	-0.275959	0.60871	-0.904252	0.600503	0
CO2m	14	-2.21919	0.986763	-1.345	0.944513	0
ADPm	14	0.350468	0.362994	-0.740922	0.649579	0

PIm	15	0.747525	0.227373	-0.653558	0.631406	0
OAm	10	-0.157812	0.562697	-0.867214	0.583591	0
GTP	10	0.305676	0.379926	-0.738237	0.542084	0
GDP	7	-0.442216	0.670834	-0.970434	0.470851	0
NADP	64	0.120739	0.451949	-0.810098	0.765093	0
D6PGL	2	-0.661235	0.745769	-1.23552	0.908206	0
NADPH	64	0.120739	0.451949	-0.810098	0.765093	0
D6PGC	7	-0.862733	0.805858	-1.11036	0.451442	0
RL5P	5	1.40044	0.0806914	-0.271638	0.920729	0
XUL5P	6	2.62656	0.00431269	0.12095	1.98751	1
R5P	15	0.468296	0.319786	-0.716968	0.636561	0
ACTP	2	3.69839	0.000108486	1.48294	3.16229	1
ACCOAm	11	-0.771965	0.779932	-1.0281	1.06935	0
H2Om	19	-0.1787	0.570913	-0.859367	0.66694	0
CITm	5	0.582575	0.28009	-0.593791	0.675366	0
COAm	13	-0.63143	0.73612	-0.977365	1.04395	0
ACOm	3	0.195732	0.42241	-0.723634	0.78128	0
ICITm	5	0.306728	0.379525	-0.702447	0.747166	0
NADm	22	-2.64244	0.995884	-1.31857	0.756831	0

AKGm	9	0.61873	0.268047	-0.641779	0.528098	0
NADHm	22	-2.64244	0.995884	-1.31857	0.756831	0
ICIT	2	0.244622	0.403375	-0.670667	1.00442	0
AKG	19	2.09739	0.0179793	-0.400249	0.915905	0
NADPm	17	-0.916667	0.820341	-1.01882	0.663142	0
NADPHm	17	-0.916667	0.820341	-1.01882	0.663142	0
ICITg	2	-0.00788078	0.503144	-0.828116	1.22709	0
NADPg	1	0.977407	0.164184	0.0395674	0	0
AKGg	1	0.977407	0.164184	0.0395674	0	0
CO2g	1	0.977407	0.164184	0.0395674	0	0
NADPHg	1	0.977407	0.164184	0.0395674	0	0
LIPOm	2	0.106125	0.457742	-0.757027	0.0277124	0
SUCDLIP	2	-0.515925	0.697047	-1.14491	0.57626	0
SUCCOAm	2	0.200262	0.420638	-0.698327	1.20782	0
DHLIPOm	2	-2.55428	0.99468	-2.41593	1.22124	0
GDPm	2	-0.298622	0.617386	-1.00941	1.64776	0
GTPm	2	-0.298622	0.617386	-1.00941	1.64776	0
SUCCm	3	-0.72135	0.764653	-1.1903	1.1657	0
Qm	9	-0.255082	0.60067	-0.898128	0.517883	0

FUMm	3	-1.51956	0.935689	-1.59648	0.462176	0
QH2m	9	-0.255082	0.60067	-0.898128	0.517883	0
FADH2m	7	-0.458096	0.676558	-0.975719	0.957574	0
FADm	7	-0.458096	0.676558	-0.975719	0.957574	0
FUM	5	-0.214316	0.58485	-0.907685	0.745445	0
SUCC	6	-0.488843	0.687523	-0.999011	0.934081	0
MALm	4	-0.527655	0.701131	-1.0557	0.736805	0
MAL	4	-0.647399	0.741313	-1.10845	0.692918	0
MALg	2	-0.594896	0.724043	-1.19415	0.147776	0
NADg	1	-0.301969	0.618662	-1.08966	0	0
OAg	1	-0.301969	0.618662	-1.08966	0	0
NADHg	1	-0.301969	0.618662	-1.08966	0	0
SUCCg	1	-0.988707	0.838597	-1.6958	0	0
GLXg	2	-1.08093	0.860137	-1.49722	0.280831	0
ACCOAg	2	-0.370015	0.644314	-1.05393	0.346084	0
H2Og	1	-0.538744	0.704968	-1.29864	0	0
COAg	2	-0.370015	0.644314	-1.05393	0.346084	0
Hm	11	-1.21384	0.887596	-1.14533	0.70225	0
CIT	1	4.2161	4.04067e-005	2.89816	0	1

COA	37	0.665307	0.252927	-0.727252	0.920999	1
ACCOA	24	1.23729	0.107989	-0.601331	1.07881	1
CAR	2	1.83816	0.0330192	0.322992	1.27564	0
ACAR	2	1.83816	0.0330192	0.322992	1.27564	0
ACARm	2	1.65359	0.0491053	0.207901	1.43841	0
CARm	2	1.65359	0.0491053	0.207901	1.43841	0
ACARg	2	1.65359	0.0491053	0.207901	1.43841	0
CARg	2	1.65359	0.0491053	0.207901	1.43841	0
OXAL	3	-0.00538795	0.502149	-0.825976	0.727302	0
AC	17	-2.80694	0.997499	-1.42198	0.514878	0
FOR	7	-0.724792	0.76571	-1.06446	0.583043	0
FORm	2	0.861454	0.194494	-0.286039	0.591197	0
METHOL	4	-1.95048	0.97444	-1.68248	0.598495	0
FALD	8	-1.89058	0.97066	-1.41166	0.654208	0
ADHLIPOm	2	-1.93223	0.973335	-2.02805	1.76979	0
ACAL	13	-2.07655	0.981078	-1.32994	0.781106	0
RGT	7	-1.23509	0.891601	-1.23427	0.292601	0
FGT	2	-1.19346	0.883656	-1.56739	0.197256	0
H+	25	-1.01628	0.845252	-1.00198	0.763549	0

HCIT	1	0.760461	0.22349	-0.151918	0	0
MTHGXL	3	-0.280896	0.610605	-0.966171	0.688424	0
LACAL	5	-0.765543	0.778026	-1.12481	0.692905	0
LAC	4	-1.06985	0.857656	-1.29454	0.54315	0
LGT	2	-0.646468	0.741012	-1.22631	0.287319	0
LLAC	3	-0.475768	0.68288	-1.06533	0.312385	0
PROP	2	-1.25733	0.895684	-1.60722	1.16949	0
AMP	38	-0.613809	0.730329	-0.910807	0.81166	1
PPI	45	-1.12823	0.870389	-0.971051	0.620406	0
PROPCOA	3	-0.429244	0.666127	-1.04166	1.28196	0
2MCIT	1	1.03393	0.150584	0.089458	0	0
GLU	34	0.932148	0.17563	-0.682884	0.80264	0
GABA	3	-1.44561	0.925857	-1.55885	0.736376	0
SUCCSAL	5	-1.35107	0.911664	-1.35545	1.03844	0
METTHF	6	-1.29654	0.902604	-1.28937	0.676422	0
METHF	2	0.895033	0.185385	-0.265101	0.561586	0
METTHFm	6	-0.0817611	0.532582	-0.852669	0.582419	0
MTHFm	2	-0.571582	0.716198	-1.17961	0.524262	0
METHFm	2	0.895033	0.185385	-0.265101	0.561586	0

FTHFm	1	1.08213	0.139597	0.132	0	0
FTHF	2	0.211685	0.416176	-0.691205	1.16419	0
THFm	3	0.245726	0.402947	-0.698194	0.74147	0
AHTD	2	-0.21928	0.586784	-0.959934	0.0347406	0
DHP	2	0.597751	0.275003	-0.450472	0.685748	0
AHHMP	1	0.971581	0.165629	0.0344253	0	0
GLAL	1	0.971581	0.165629	0.0344253	0	0
CHOR	3	0.00683743	0.497272	-0.819755	0.738889	0
GLN	13	-0.0498219	0.519868	-0.835464	0.668273	0
PABA	1	0.971581	0.165629	0.0344253	0	0
AHHMD	1	0.971581	0.165629	0.0344253	0	0
DHPT	1	0.971581	0.165629	0.0344253	0	0
DHF	1	-1.72408	0.957653	-2.34486	0	0
THF	8	-0.783477	0.783326	-1.06712	0.48782	0
MTHF	1	0.646785	0.258886	-0.252252	0	0
THFG	2	-0.316506	0.624191	-1.02056	0.0992404	0
OIVAL	2	-0.615952	0.731037	-1.20728	0.868273	0
AKP	1	-1.13083	0.870937	-1.82124	0	0
PANT	1	-0.944489	0.82754	-1.65677	0	0

AKPm	1	-0.208233	0.582476	-1.00692	0	0
PANTm	1	-0.208233	0.582476	-1.00692	0	0
bALA	1	-0.944489	0.82754	-1.65677	0	0
PNTO	2	-0.282537	0.611234	-0.999379	0.929691	0
4PPNTO	1	0.545117	0.292836	-0.341988	0	0
CTP	7	-0.478313	0.683786	-0.982446	0.671786	0
CYS	7	0.0177338	0.492926	-0.817383	0.926217	0
CMP	8	2.51804	0.00590043	-0.0396438	0.916241	1
ASP	18	-1.2882	0.901162	-1.0903	0.426731	0
PAP	3	-1.31674	0.906037	-1.49327	1.75437	0
ACP	10	1.04435	0.148161	-0.532682	0.476588	0
ALA	6	0.580606	0.280753	-0.614553	0.425436	0
CHCOA	1	0.116229	0.453736	-0.720541	0	0
AONA	1	0.116229	0.453736	-0.720541	0	0
DTB	1	0.917382	0.179471	-0.0134134	0	0
BT	1	0.917382	0.179471	-0.0134134	0	0
ETH	4	-1.95048	0.97444	-1.68248	0.598495	0
ETHm	4	-1.95048	0.97444	-1.68248	0.598495	0
ACALm	9	-2.42392	0.992323	-1.5344	0.545406	0

ACm	9	-2.01998	0.978307	-1.41589	0.623076	0
AMPm	9	-1.87992	0.969941	-1.3748	0.633879	0
PPIm	11	-0.796339	0.787082	-1.03457	0.695863	0
ACTPm	1	0.079303	0.468396	-0.753133	0	0
GLYN	11	-0.419536	0.662588	-0.934602	0.73933	0
GL	14	-1.29235	0.901882	-1.12712	0.773078	0
GLYAL	9	-1.01209	0.844252	-1.12021	0.759642	0
O2	34	0.793852	0.213641	-0.703722	1.05642	1
H2O2	10	0.805364	0.210305	-0.599186	1.53365	1
GL3P	7	0.125187	0.450188	-0.781626	0.613217	0
TAR	2	-0.112033	0.544601	-0.89306	0.470099	0
OXGLY	2	-0.112033	0.544601	-0.89306	0.470099	0
G	1	0.0970923	0.461327	-0.737431	0	0
E	6	1.01962	0.153955	-0.456732	0.500564	0
EOL	6	1.01962	0.153955	-0.456732	0.500564	0
LXUL	6	-0.169473	0.567288	-0.8842	0.501627	0
XOL	6	-0.63156	0.736163	-1.05032	0.511005	0
XUL	7	-0.626353	0.734458	-1.03171	0.469073	0
AOL	6	-0.63156	0.736163	-1.05032	0.511005	0

XYL	3	-0.529201	0.701667	-1.09252	0.794569	0
ARAB	2	0.995315	0.159791	-0.202569	0.0911173	0
ARABLAC	2	0.995315	0.159791	-0.202569	0.0911173	0
LAOL	3	0.123697	0.450778	-0.76029	0.752915	0
RIB	3	-0.0630089	0.52512	-0.855297	0.409038	0
R1P	2	1.56401	0.0589082	0.152039	1.15405	0
RL	2	1.34529	0.0892658	0.0156587	0.0845979	0
O2e	7	0.98218	0.163005	-0.496455	1.84332	1
GLCN15LACe	3	-0.193333	0.576651	-0.921613	0.270917	0
H2O2e	7	0.98218	0.163005	-0.496455	1.84332	1
H2Oe	19	0.469973	0.319187	-0.728521	1.17695	1
GLCNTe	1	0.0699478	0.472118	-0.76139	0	0
GLCN15LAC	1	0.0699478	0.472118	-0.76139	0	0
GLCNT	6	-0.311524	0.622299	-0.935266	0.336891	0
GLAC	18	0.346	0.364672	-0.751609	0.591013	0
GALOL	5	0.768768	0.221015	-0.52045	0.531747	0
GAL1P	2	-0.928298	0.823374	-1.40205	0.461997	0
UTP	7	-0.699934	0.758016	-1.05619	0.519972	0
UDPGAL	4	-0.496704	0.690301	-1.04206	0.584435	0

UDPG	10	-1.01978	0.846085	-1.10708	0.683823	0
G1P	4	0.494119	0.310611	-0.605584	1.07916	0
MELI	4	-1.04425	0.851816	-1.28327	0.408723	0
GALN14LAC	3	0.233627	0.407637	-0.704351	0.676174	0
GALNT	2	0.389993	0.348271	-0.58002	0.539406	0
2D3DGALT	2	0.389993	0.348271	-0.58002	0.539406	0
SOR	8	-1.10145	0.86465	-1.16608	0.422066	0
SOT	4	-0.829266	0.796523	-1.18856	0.375504	0
MAN6P	5	-0.197301	0.578204	-0.900983	0.753967	0
MAN	4	0.439658	0.330092	-0.629575	0.311772	0
FRU	8	-0.0749565	0.529875	-0.846617	0.324643	0
MNT6P	1	1.94119	0.0261179	0.890234	0	0
MNT	4	-0.545732	0.707375	-1.06366	0.151974	0
F26P	1	0.0733327	0.470771	-0.758403	0	0
MAN1P	1	0.971581	0.165629	0.0344253	0	0
GDPMAN	2	1.2885	0.0987851	-0.0197499	0.0766153	0
IDOL	3	-1.07896	0.859698	-1.37228	0.310126	0
UDP	17	-1.02939	0.848352	-1.04287	0.829076	0
TRE6P	3	-0.386678	0.650503	-1.02	0.889034	0

TRE	3	1.97716	0.0240118	0.182863	1.16547	0
MLT	5	-0.881822	0.811064	-1.17061	0.659126	0
MLTe	5	-0.881822	0.811064	-1.17061	0.659126	0
LACT	4	1.02078	0.15368	-0.373582	0.35311	0
LACTe	4	1.02078	0.15368	-0.373582	0.35311	0
GLACe	5	1.099	0.135884	-0.390372	0.308099	0
13GLUCAN	2	0.62386	0.26636	-0.434191	0.379803	0
GA6P	4	-0.707935	0.760507	-1.13511	0.942095	0
NAGA6P	2	-0.65432	0.743547	-1.2312	1.76561	0
NAGA1P	2	0.541996	0.293911	-0.485238	0.710656	0
UDPNAG	9	-1.13899	0.872647	-1.15744	0.92104	0
CHIT	13	-1.87787	0.969801	-1.28147	0.982879	0
NAG	5	-1.58091	0.943051	-1.44599	1.07454	0
GLCN	3	0.267876	0.394397	-0.686923	0.355064	0
13GLUCANe	12	-0.738632	0.769935	-1.0109	0.573135	0
GLYCOGEN	2	-0.958456	0.831084	-1.42085	0.805296	0
STARe	2	1.40538	0.0799542	0.0531276	0.625022	0
GLYCOGENe	2	1.40538	0.0799542	0.0531276	0.625022	0
AMYLSe	1	0.18293	0.427427	-0.661668	0	0

AMYLPe	1	0.18293	0.427427	-0.661668	0	0
CELLUe	8	-0.614097	0.730424	-1.0144	0.443864	0
CELLOBe	8	-0.614097	0.730424	-1.0144	0.443864	0
CELLOTe	4	-0.549556	0.708688	-1.06534	0.355792	0
MANNANe	6	-0.662438	0.746155	-1.06142	0.558317	0
MANe	7	-0.456382	0.675942	-0.975148	0.558446	0
PECTATEe	1	0.638448	0.261591	-0.259611	0	0
GALUNTe	1	0.638448	0.261591	-0.259611	0	0
ARABINe	5	0.237822	0.40601	-0.729589	0.549646	0
LARABe	5	0.237822	0.40601	-0.729589	0.549646	0
XYLANe	6	-0.692372	0.755648	-1.07218	1.02836	0
XYLe	6	-0.692372	0.755648	-1.07218	1.02836	0
H+_PO_mit	8	0.782396	0.216991	-0.579799	0.412048	0
H+_PO	8	0.782396	0.216991	-0.579799	0.412048	0
FERIm	5	1.08287	0.139433	-0.396726	0.737956	0
FEROm	5	1.08287	0.139433	-0.396726	0.737956	0
O2m	1	0.296281	0.383508	-0.56162	0	0
K	1	-0.467598	0.679964	-1.23585	0	0
Km	1	-0.467598	0.679964	-1.23585	0	0

Ca	1	0.29738	0.383088	-0.56065	0	0
Cam	1	0.29738	0.383088	-0.56065	0	0
LLACm	6	-1.10205	0.864781	-1.21946	0.679025	0
LACm	1	-0.764038	0.777578	-1.4975	0	0
GLUm	10	0.336015	0.36843	-0.729794	0.581329	0
ASPm	6	-0.553323	0.709979	-1.02219	0.414039	0
ALAm	1	0.71884	0.23612	-0.188654	0	0
ASN	5	-1.00634	0.842875	-1.21966	0.672353	0
SAM	12	0.893335	0.185839	-0.596426	0.620616	0
HCYS	8	-0.541437	0.705897	-0.991792	0.690521	0
SAH	8	0.444136	0.328472	-0.685069	0.707505	0
MET	4	0.639634	0.261205	-0.541482	0.599277	0
TRNAm	4	-0.472462	0.681701	-1.03138	0.402558	0
ASPTRNAm	3	-0.533261	0.703074	-1.09459	0.46809	0
TRNA	3	-0.656934	0.744388	-1.15752	0.518406	0
ASPTRNA	3	-0.656934	0.744388	-1.15752	0.518406	0
NH3	29	2.38088	0.00863561	-0.434815	1.49152	2
NAGLUm	2	-0.172242	0.568376	-0.930604	0.523193	0
NAGLUPm	1	-0.540911	0.705716	-1.30056	0	0

NAGLUSm	2	0.224483	0.411191	-0.683225	0.87304	0
NAORNm	2	0.817782	0.206741	-0.313271	0.349847	0
ORNm	1	0.29738	0.383088	-0.56065	0	0
CAP	5	2.18063	0.0146056	0.0356769	2.48078	1
ORN	5	3.54942	0.000193042	0.574841	2.39381	1
CITR	2	4.08148	7.28988e-005	1.72182	3.87971	1
GLUGSAL	3	1.07673	0.1408	-0.275328	0.457136	0
ARGSUCC	2	0.764036	0.222423	-0.346784	0.954263	0
ARG	3	1.59388	0.0554815	-0.0121732	0.763944	0
PTRSC	2	-1.2362	0.891808	-1.59404	0.697531	0
DSAM	2	-0.0921336	0.536704	-0.880652	0.311348	0
SPRMD	3	-0.34775	0.635986	-1.00019	0.165733	0
5MTA	1	-0.314602	0.623468	-1.10081	0	0
SPRM	2	-0.211136	0.58361	-0.954857	0.206407	0
GBAD	3	2.14655	0.0159147	0.269056	3.76423	1
GBAT	3	2.14655	0.0159147	0.269056	3.76423	1
UREA	3	1.06432	0.143591	-0.281643	1.27753	0
ATRNA	1	-0.0724922	0.528895	-0.887113	0	0
ALTRNA	1	-0.0724922	0.528895	-0.887113	0	0

DAPRP	1	-0.303327	0.61918	-1.09086	0	0
SLF	1	0.414208	0.339361	-0.457533	0	0
APS	2	0.356218	0.360839	-0.601081	0.203008	0
PAPS	2	-1.5461	0.938959	-1.78727	1.47452	0
SER	11	0.314881	0.376426	-0.739767	0.776622	0
ASER	5	-1.31651	0.905999	-1.34184	0.512449	0
H2S	5	-1.18677	0.882341	-1.29073	0.48609	0
RTHIO	4	-0.876233	0.809548	-1.20925	1.11236	0
OTHIO	4	-0.876233	0.809548	-1.20925	1.11236	0
H2SO3	2	-2.01173	0.977876	-2.07762	1.06391	0
GLUGSALm	3	-0.373611	0.645653	-1.01335	0.862197	0
P5Cm	4	-0.177138	0.5703	-0.901286	0.738797	0
PHP	1	0.828363	0.203733	-0.0919849	0	0
GLYm	2	-0.46522	0.679113	-1.11329	0.256361	0
GLY	11	-0.738878	0.770009	-1.01932	0.643169	0
GLX	2	-0.343958	0.634561	-1.03768	0.355903	0
BASP	2	0.464543	0.32113	-0.533535	0.31365	0
ASPSA	2	0.468508	0.319711	-0.531062	0.310153	0
HSER	3	0.7931	0.21386	-0.419657	0.418707	0

PHSER	2	0.697289	0.242811	-0.388405	0.621593	0
THR	8	-0.0649673	0.5259	-0.843508	0.879697	0
LLCT	5	0.251816	0.400592	-0.724077	1.14235	0
OBUT	4	1.83002	0.0336234	-0.0170932	0.834254	0
THRm	3	0.807082	0.20981	-0.412543	0.800167	0
NH3m	3	0.807082	0.20981	-0.412543	0.800167	0
OBUTm	4	0.630597	0.264152	-0.545463	0.705349	0
PRPP	9	-0.360249	0.64067	-0.928981	0.537934	0
PRBATP	3	-0.3655	0.642631	-1.00922	1.07858	0
PRBAMP	2	0.650733	0.257609	-0.417435	0.474749	0
PRFP	3	0.258966	0.397831	-0.691457	0.581341	0
PRLP	2	-0.218208	0.586367	-0.959266	0.396312	0
DIMGP	2	0.561598	0.287195	-0.473015	0.291351	0
IMACP	2	1.22335	0.110598	-0.0603757	0.292209	0
HISOLP	2	1.01371	0.155362	-0.191102	0.477084	0
HISOL	3	0.724745	0.234304	-0.45444	0.341762	0
HIS	4	-0.129766	0.551624	-0.880418	0.643325	0
AICAR	4	-1.06135	0.855735	-1.2908	0.408109	0
HTRNA	1	-0.9087	0.818246	-1.62518	0	0

HHTRNA	1	-0.9087	0.818246	-1.62518	0	0
MHIS	1	-0.270203	0.606498	-1.06162	0	0
OICAPm	1	0.260368	0.39729	-0.593319	0	0
LEUm	1	0.260368	0.39729	-0.593319	0	0
OMVALm	2	0.738206	0.230195	-0.362891	0.325874	0
ILEm	1	0.260368	0.39729	-0.593319	0	0
OMVAL	1	0.260368	0.39729	-0.593319	0	0
ILE	1	0.260368	0.39729	-0.593319	0	0
VAL	4	-0.610404	0.729203	-1.09215	0.556474	0
OICAP	3	0.98328	0.162735	-0.322882	0.396279	0
LEU	1	0.260368	0.39729	-0.593319	0	0
ABUTm	2	-0.24436	0.596524	-0.975573	0.0443345	0
ACLACm	2	-0.24436	0.596524	-0.975573	0.0443345	0
DHVALm	2	0.406555	0.342167	-0.569693	0.618336	0
DHMVAm	2	0.406555	0.342167	-0.569693	0.618336	0
OIVALm	2	0.485882	0.313525	-0.520228	0.548382	0
IPPMALm	1	-0.0961484	0.538299	-0.907993	0	0
CBHCAP	1	-1.26711	0.897443	-1.94153	0	0
IPPMAL	3	0.100121	0.460124	-0.772286	1.06185	0

PPMAL	1	-1.26711	0.897443	-1.94153	0	0
HCITm	1	-0.375044	0.646186	-1.15416	0	0
HACNm	2	-0.848425	0.801899	-1.35224	0.280132	0
HICITm	2	-2.62453	0.995662	-2.45974	1.28611	0
OXAm	1	-2.88456	0.99804	-3.36915	0	0
MICIT	1	-0.880541	0.810717	-1.60033	0	0
AKA	1	-0.288227	0.613414	-1.07753	0	0
AMA	4	-0.0705615	0.528127	-0.854337	0.185171	0
AMASA	2	-0.0923921	0.536807	-0.880813	0.0554912	0
SACP	2	0.890384	0.18663	-0.268	0.811158	0
LYS	3	-0.0546701	0.521799	-0.851053	1.03487	0
LTRNA	2	-0.972116	0.834503	-1.42937	0.367732	0
LLTRNA	2	-0.972116	0.834503	-1.42937	0.367732	0
LYSm	2	-0.972116	0.834503	-1.42937	0.367732	0
LTRNAm	2	-0.972116	0.834503	-1.42937	0.367732	0
LLTRNAm	2	-0.972116	0.834503	-1.42937	0.367732	0
ADN	7	2.99096	0.00139051	0.171982	1.70425	2
MTHPTGLU	1	0.646785	0.258886	-0.252252	0	0
THPTGLU	1	0.646785	0.258886	-0.252252	0	0

OAHSER	2	0.40865	0.341398	-0.568387	0.0122476	0
METH	1	0.278803	0.390198	-0.577047	0	0
OSLHSER	1	0.925717	0.177296	-0.00605609	0	0
CALH	1	-0.182828	0.572533	-0.984499	0	0
DPTH	1	-0.182828	0.572533	-0.984499	0	0
3DDAH7P	3	-0.00827117	0.5033	-0.827443	1.03943	0
DQT	3	-0.286342	0.612692	-0.968942	0.342717	0
DHSK	2	-0.538879	0.705015	-1.15922	0.132931	0
QT	1	0.265961	0.395135	-0.588382	0	0
SME	1	-0.487278	0.686969	-1.25322	0	0
SME5P	1	-0.487278	0.686969	-1.25322	0	0
3PSME	2	-0.840886	0.799794	-1.34754	0.133389	0
PHEN	2	-0.487766	0.687142	-1.12735	0.159783	0
PHPYR	2	1.59669	0.0551674	0.17242	1.98758	0
PHE	1	2.72023	0.0032618	1.57785	0	0
4HPP	6	0.687112	0.246006	-0.576265	1.19794	0
TYR	4	0.555022	0.28944	-0.578755	1.44683	0
AN	4	0.979982	0.163548	-0.391552	0.380145	0
NPRAN	2	0.493275	0.310909	-0.515618	0.206546	0

CPAD5P	1	0.513869	0.303672	-0.369569	0	0
IGP	2	1.02328	0.153088	-0.185133	0.260832	0
TRP	3	0.598512	0.274749	-0.518675	0.523237	0
FKYN	3	-0.425945	0.664926	-1.03998	0.528185	0
KYN	4	0.0776843	0.46904	-0.789032	0.589579	0
HKYN	3	0.575265	0.282556	-0.530505	0.346972	0
HAN	2	0.597303	0.275152	-0.450751	0.450127	0
CMUSA	1	0.268647	0.394101	-0.586011	0	0
AM6SA	2	0.258968	0.39783	-0.661721	0.10707	0
AMUCO	1	0.0970923	0.461327	-0.737431	0	0
HOMOGEN	2	0.0878708	0.46499	-0.76841	1.22496	0
MACAC	2	-0.227804	0.590101	-0.96525	0.946584	0
FUACAC	2	0.414274	0.339337	-0.56488	0.380375	0
ACTAC	1	-0.0121417	0.504844	-0.833845	0	0
TRPm	1	-0.0211091	0.508421	-0.841761	0	0
TRPTRNAm	1	-0.0211091	0.508421	-0.841761	0	0
PAD	3	1.94645	0.0258003	0.167236	3.82348	1
PAC	3	1.94645	0.0258003	0.167236	3.82348	1
IAD	3	1.94645	0.0258003	0.167236	3.82348	1

IAC	3	1.94645	0.0258003	0.167236	3.82348	1
ASPERMD	2	0.0849591	0.466147	-0.770225	0.0547008	0
APRUT	1	0.103761	0.45868	-0.731546	0	0
APROA	1	0.103761	0.45868	-0.731546	0	0
GABAL	1	0.103761	0.45868	-0.731546	0	0
ASPRM	2	0.0849591	0.466147	-0.770225	0.0547008	0
GLUP	2	0.546418	0.292389	-0.482481	0.448558	0
P5C	1	0.782502	0.21696	-0.132463	0	0
PRO	1	0.782502	0.21696	-0.132463	0	0
PHC	1	0.782502	0.21696	-0.132463	0	0
HPRO	1	0.782502	0.21696	-0.132463	0	0
PROm	1	0.292342	0.385013	-0.565097	0	0
GABALm	5	-1.50462	0.933789	-1.41593	0.535555	0
GABAm	5	-1.50462	0.933789	-1.41593	0.535555	0
LACALm	5	-1.50462	0.933789	-1.41593	0.535555	0
APROP	1	0.26721	0.394654	-0.587279	0	0
TCOA	1	-0.101349	0.540363	-0.912583	0	0
GLP	1	-0.101349	0.540363	-0.912583	0	0
TGLP	1	-0.101349	0.540363	-0.912583	0	0

PEPD	1	0.145084	0.442322	-0.695072	0	0
APEP	1	0.145084	0.442322	-0.695072	0	0
GC	2	-0.364664	0.642319	-1.05059	0.19269	0
OGT	2	-0.394307	0.653323	-1.06907	0.115011	0
cAMP	3	0.767106	0.221509	-0.432885	0.241678	0
GMP	6	1.76885	0.0384594	-0.187388	1.27024	1
DGMP	2	3.00513	0.00132734	1.05066	1.47237	1
DGDP	2	-0.452942	0.674705	-1.10564	0.0899395	0
DATP	2	-0.0241823	0.509646	-0.838281	0.468036	0
DADP	4	-1.03868	0.850523	-1.28081	0.62276	0
PRAM	1	-0.248017	0.597939	-1.04204	0	0
GAR	1	-0.248017	0.597939	-1.04204	0	0
FGAR	1	0.432334	0.332749	-0.441535	0	0
FGAM	2	0.130567	0.448059	-0.741786	0.42462	0
AIR	2	0.27302	0.392419	-0.652959	0.55024	0
CAIR	2	0.214177	0.415204	-0.689651	0.60213	0
SAICAR	3	-1.05823	0.855026	-1.36173	0.213381	0
PRFICA	1	-0.783199	0.783245	-1.51441	0	0
IMP	9	1.24988	0.105671	-0.456618	1.07142	1

ASUC	3	-0.996743	0.840555	-1.33044	0.267562	0
XMP	5	1.44112	0.0747752	-0.255612	1.45058	1
cdAMP	1	0.72884	0.23305	-0.179827	0	0
DAMP	5	1.58713	0.0562412	-0.198098	1.53982	1
cIMP	1	0.72884	0.23305	-0.179827	0	0
cGMP	1	0.72884	0.23305	-0.179827	0	0
cCMP	1	0.72884	0.23305	-0.179827	0	0
ATN	1	-0.520147	0.698519	-1.28223	0	0
ATT	2	-1.11503	0.867582	-1.51848	0.334114	0
UGC	2	-0.717141	0.763357	-1.27038	0.68499	0
CAASP	3	-0.538252	0.704798	-1.09713	0.204446	0
DOROA	1	-0.685197	0.75339	-1.42791	0	0
OROA	2	-0.427817	0.665608	-1.08997	0.47792	0
OMP	2	0.714724	0.23739	-0.377533	0.529616	0
UMP	6	2.3901	0.00842195	0.035945	1.07134	1
URA	6	-0.0539103	0.521497	-0.842657	0.55359	0
CYTS	2	-0.874254	0.80901	-1.36835	0.720358	0
URI	4	2.32323	0.0100834	0.200176	1.29887	1
CYTD	4	2.32323	0.0100834	0.200176	1.29887	1

DU	4	2.31751	0.010238	0.197654	1.30109	1
DR1P	1	0.180294	0.428461	-0.663994	0	0
DT	3	2.55933	0.00524366	0.479107	1.43665	1
THY	1	0.180294	0.428461	-0.663994	0	0
DC	3	2.57066	0.00507525	0.484871	1.42979	1
DTMP	4	1.12608	0.130066	-0.327193	1.87799	1
DTDP	2	-0.471535	0.681371	-1.11723	0.0735439	0
OTHIOm	1	0.414	0.339437	-0.457717	0	0
RTHIOm	1	0.414	0.339437	-0.457717	0	0
DUTP	3	-0.348279	0.636185	-1.00046	0.434091	0
DUMP	5	1.13768	0.127628	-0.375138	1.65982	1
DCMP	3	2.79297	0.0026113	0.597997	1.30332	1
DCDP	3	-0.0320282	0.512775	-0.839532	0.465272	0
CDP	4	0.245409	0.40307	-0.715146	0.454099	0
PURI5P	3	0.574386	0.282853	-0.530952	0.256285	0
AD	5	1.60945	0.0537585	-0.189306	1.80721	1
INS	5	4.04668	8.46714e-005	0.770714	1.66984	2
DA	4	4.38437	1.88651e-005	1.10815	1.7201	2
DIN	2	3.18953	0.000712529	1.16564	2.58749	1

HYXN	3	2.66203	0.00388354	0.531365	2.13412	1
DG	4	2.58579	0.00485775	0.315839	1.21762	1
GN	3	-0.168134	0.566761	-0.908791	0.36247	0
GSN	6	1.99299	0.0231311	-0.106811	1.17216	1
XAN	2	0.196637	0.422056	-0.700588	0.0517509	0
XTSINE	3	2.55933	0.00524366	0.479107	1.43665	1
ITP	2	-0.208691	0.582655	-0.953332	0.305329	0
IDP	2	-0.208691	0.582655	-0.953332	0.305329	0
ITPm	1	-1.53111	0.937129	-2.17455	0	0
IDPm	1	-1.53111	0.937129	-2.17455	0	0
DGTP	3	0.405598	0.342519	-0.616842	0.506591	0
DUDP	3	-0.0320282	0.512775	-0.839532	0.465272	0
DCTP	2	-0.0241823	0.509646	-0.838281	0.468036	0
DTTP	1	-0.392124	0.652517	-1.16923	0	0
LCCA	4	-1.18949	0.882877	-1.34725	0.579739	0
ACOA	7	-1.16316	0.877617	-1.21033	0.542693	0
HACOA	3	0.600438	0.274107	-0.517695	0.340342	0
OACOA	6	0.140585	0.444099	-0.772737	0.491078	0
AACCOA	3	0.786032	0.215924	-0.423254	0.798221	0

AACCOAm	2	1.28532	0.0993397	-0.021734	0.55408	0
ACACPm	2	-2.22014	0.986795	-2.20758	0.880129	0
MALACPm	2	-2.22014	0.986795	-2.20758	0.880129	0
C100ACPm	1	-0.863437	0.806051	-1.58523	0	0
ACPm	2	-2.22014	0.986795	-2.20758	0.880129	0
C120ACPm	1	-0.863437	0.806051	-1.58523	0	0
C140ACPm	1	-0.863437	0.806051	-1.58523	0	0
C141ACPm	1	-0.863437	0.806051	-1.58523	0	0
C160ACPm	1	-0.863437	0.806051	-1.58523	0	0
C161ACPm	1	-0.863437	0.806051	-1.58523	0	0
C180ACPm	1	-0.863437	0.806051	-1.58523	0	0
C181ACPm	1	-0.863437	0.806051	-1.58523	0	0
C182ACPm	1	-0.863437	0.806051	-1.58523	0	0
C150ACPm	1	-0.863437	0.806051	-1.58523	0	0
C162ACPm	1	-0.863437	0.806051	-1.58523	0	0
C170ACPm	1	-0.863437	0.806051	-1.58523	0	0
C183ACPm	1	-0.863437	0.806051	-1.58523	0	0
C200ACPm	1	-0.863437	0.806051	-1.58523	0	0
MALCOA	7	1.04606	0.147766	-0.475198	0.55509	0

MALACP	4	-0.22849	0.590367	-0.923908	0.278209	0
ACACP	4	0.917372	0.179474	-0.419133	0.620742	0
3OACPm	1	-2.27363	0.988506	-2.82992	0	0
C100ACP	1	-0.559543	0.712104	-1.317	0	0
C120ACP	9	-0.0354387	0.514135	-0.833691	0.47343	0
C140ACP	9	-0.0354387	0.514135	-0.833691	0.47343	0
C141ACP	9	-0.0354387	0.514135	-0.833691	0.47343	0
C160ACP	9	-0.0354387	0.514135	-0.833691	0.47343	0
C161ACP	9	-0.0354387	0.514135	-0.833691	0.47343	0
C180ACP	9	-0.0354387	0.514135	-0.833691	0.47343	0
C181ACP	9	-0.0354387	0.514135	-0.833691	0.47343	0
C182ACP	9	-0.0354387	0.514135	-0.833691	0.47343	0
3HPACP	3	1.38229	0.0834415	-0.119843	0.201331	0
2HDACP	3	1.38229	0.0834415	-0.119843	0.201331	0
AACP	3	1.38229	0.0834415	-0.119843	0.201331	0
23DAACP	3	1.38229	0.0834415	-0.119843	0.201331	0
C150ACP	6	-0.820626	0.79407	-1.11828	0.141428	0
C162ACP	9	-0.0354387	0.514135	-0.833691	0.47343	0
C170ACP	9	-0.0354387	0.514135	-0.833691	0.47343	0

C183ACP	9	-0.0354387	0.514135	-0.833691	0.47343	0
C200ACP	9	-0.0354387	0.514135	-0.833691	0.47343	0
C140	1	0.592086	0.276896	-0.300531	0	0
C160	1	0.592086	0.276896	-0.300531	0	0
C180	1	0.592086	0.276896	-0.300531	0	0
AGL3P	3	0.358147	0.360117	-0.640988	0.87206	0
AT3P2	2	0.15328	0.439089	-0.727624	1.21488	0
PA	5	-0.610584	0.729262	-1.06377	0.823584	0
PAm	1	0.646785	0.258886	-0.252252	0	0
CTPm	2	0.180352	0.428438	-0.710742	0.648403	0
CDPDGm	3	0.164843	0.434534	-0.739352	0.455278	0
CDPDG	2	1.15743	0.123548	-0.101482	0.213221	0
PS	3	0.425326	0.3353	-0.606803	0.824198	0
CMPm	3	0.128767	0.448771	-0.75771	0.425985	0
PSm	1	-1.38756	0.917364	-2.04784	0	0
PE	6	0.655136	0.25619	-0.58776	0.534521	0
PEm	1	-1.38756	0.917364	-2.04784	0	0
PMME	2	1.16306	0.122402	-0.0979705	0.966244	0
PDME	1	1.59567	0.0552815	0.585267	0	0

PC	3	1.39529	0.0814635	-0.113225	0.604935	0
CHO	1	-0.768355	0.778862	-1.50131	0	0
PCHO	2	-1.36553	0.913957	-1.67468	0.245189	0
CDPCHO	2	-0.536738	0.704276	-1.15789	0.976049	0
DAGLY	11	-1.30738	0.904458	-1.17014	0.476751	0
PETHM	2	-0.209384	0.582926	-0.953764	0.0616006	0
CDPETN	2	0.215284	0.414773	-0.688961	0.312888	0
MI1P	2	-0.587718	0.721639	-1.18968	1.71327	0
MYOI	2	-0.565671	0.714191	-1.17593	1.73272	0
PINS	7	0.290184	0.385838	-0.726723	0.546939	0
PINSP	4	0.0186055	0.492578	-0.815057	0.402619	0
PINS4P	3	0.0562893	0.477556	-0.794591	0.731163	0
D45PI	3	0.0216371	0.491369	-0.812224	0.767924	0
TPI	2	-0.614072	0.730416	-1.20611	0.498541	0
GL3Pm	1	-0.375044	0.646186	-1.15416	0	0
PGPm	2	-0.743995	0.77156	-1.28712	0.188042	0
PGm	2	-0.469352	0.680591	-1.11587	0.430233	0
CLm	1	0.013008	0.494811	-0.811647	0	0
DGPP	2	-1.0274	0.847884	-1.46384	1.00509	0

LPC	1	0.414555	0.339234	-0.457227	0	0
LPE	1	0.414555	0.339234	-0.457227	0	0
CDPm	2	0.136192	0.445835	-0.738279	0.60946	0
PALCOA	2	1.41239	0.0789179	0.0574976	0.276673	0
DHSPH	3	0.712181	0.238176	-0.460834	0.918845	0
SPH	4	-0.250319	0.59883	-0.933524	0.9463	0
PSPH	3	0.759676	0.223724	-0.436666	0.535095	0
C260COA	1	-0.24523	0.596861	-1.03958	0	0
CER2	2	-0.222632	0.588089	-0.962025	0.109677	0
CER3	2	0.292745	0.384858	-0.640659	0.344802	0
IPC	2	0.942686	0.172921	-0.235386	0.22834	0
MIPC	2	0.942686	0.172921	-0.235386	0.22834	0
MIP2C	1	0.482963	0.314561	-0.396847	0	0
DHSP	3	-0.335368	0.631326	-0.993889	0.97401	0
PHSP	1	0.911997	0.180885	-0.0181665	0	0
C16A	1	-0.197356	0.578225	-0.997322	0	0
H3MCOA	3	-0.623713	0.733592	-1.14062	0.515921	0
MVL	3	-0.0444394	0.517723	-0.845847	0.671621	0
PMVL	2	0.243082	0.403971	-0.671627	0.46618	0

PPMVL	2	-0.127926	0.550896	-0.90297	0.139012	0
IPPP	3	-0.5822	0.719784	-1.11949	0.495832	0
DMPP	2	-0.727603	0.766572	-1.2769	0.58569	0
GPP	1	-0.983323	0.837276	-1.69105	0	0
FPP	1	-0.983323	0.837276	-1.69105	0	0
S23E	1	-0.0208988	0.508337	-0.841575	0	0
LNST	4	-0.527979	0.701243	-1.05584	1.15109	0
IGST	5	0.900541	0.183916	-0.468545	1.40456	0
DMZYMST	5	0.682385	0.247498	-0.554477	1.09059	0
IMZYMST	4	-0.12156	0.548376	-0.876803	0.826504	0
IIMZYMST	2	1.79585	0.0362595	0.296605	0.0107743	0
MZYMST	4	-0.130207	0.551799	-0.880612	0.81925	0
IZYMST	4	-0.12156	0.548376	-0.876803	0.826504	0
IIZYMST	2	1.79585	0.0362595	0.296605	0.0107743	0
ZYMST	2	0.950212	0.171002	-0.230694	0.734938	0
FEST	2	0.0436654	0.482586	-0.795974	0.0644892	0
EPST	3	-0.545367	0.707249	-1.10075	0.272754	0
ERTROL	3	0.563485	0.286552	-0.536499	1.21171	0
ERTEOL	2	2.90141	0.00185745	0.985982	0.190645	0

ERGOST	1	2.2024	0.0138187	1.12079	0	0
TAGLY	5	-0.648074	0.741532	-1.07854	0.114698	0
MAGLY	4	-0.660768	0.745619	-1.11433	0.0948681	0
PHACAL	6	-1.56379	0.941066	-1.38545	0.484801	0
PHAC	6	-1.1507	0.875071	-1.23694	0.64937	0
PHACCOA	2	0.524163	0.300083	-0.496358	0.218312	0
LLDACV	2	0.191815	0.423944	-0.703595	0.0925525	0
IPN	2	0.286684	0.387177	-0.644439	0.00889328	0
PENG	1	0.195324	0.42257	-0.650728	0	0
NOR	4	0.907049	0.18219	-0.42368	1.00626	0
AVN	5	-0.024982	0.509965	-0.833107	0.703574	0
HAVN	4	0.373351	0.354444	-0.658785	0.439827	0
AVF	2	0.713233	0.237851	-0.378463	0.385042	0
VHA	2	-0.130303	0.551837	-0.904452	0.35882	0
VERB	3	-1.64853	0.950378	-1.6621	0.879909	0
VERA	2	-1.19625	0.8842	-1.56912	1.22336	0
DMST	2	-1.19625	0.8842	-1.56912	1.22336	0
DHDMST	2	-1.19625	0.8842	-1.56912	1.22336	0
ST	1	1.15648	0.123743	0.197623	0	0

DHST	1	1.15648	0.123743	0.197623	0	0
OMST	3	0.879383	0.189597	-0.375751	1.38508	0
DHOMST	3	0.879383	0.189597	-0.375751	1.38508	0
AFB1	2	0.257817	0.398274	-0.662439	1.82859	0
AFG1	2	0.257817	0.398274	-0.662439	1.82859	0
AFB2	2	0.257817	0.398274	-0.662439	1.82859	0
AFG2	2	0.257817	0.398274	-0.662439	1.82859	0
HNO3	3	0.24748	0.402268	-0.697301	0.656966	0
HNO2	2	0.279792	0.389819	-0.648737	0.898713	0
NH4OH	1	-0.522405	0.699306	-1.28422	0	0
UREAC	1	1.37126	0.0851468	0.387197	0	0
ACNL	1	0.26721	0.394654	-0.587279	0	0
INAC	1	0.26721	0.394654	-0.587279	0	0
NH3e	2	0.725375	0.234111	-0.370892	1.01183	0
HNO3e	2	-0.346602	0.635555	-1.03933	0.401648	0
FRUe	1	0.414208	0.339361	-0.457533	0	0
SORe	1	0.414208	0.339361	-0.457533	0	0

#Results for Up-regulated only genes

#Feature	Number of neighbors	Z-score	P-value	Average Z	StdDev Z	Significance count
DGLCe	2	-0.529382	0.70173	-1.1533	0.0977921	0
GLCe	11	-0.30389	0.619394	-0.903922	0.68231	0
GLC	13	-1.64238	0.949744	-1.22402	0.639899	0
bDGLC	4	-1.65939	0.951481	-1.55425	0.376225	0
ATP	46	-0.148953	0.559205	-0.842629	0.813273	1
ADP	31	0.399837	0.344638	-0.760232	0.908264	1
G6P	5	0.454343	0.324791	-0.644302	1.06838	0
bDG6P	2	-1.13517	0.871848	-1.53104	0.615376	0
H2O	35	-0.184551	0.573209	-0.850742	1.00087	1
PI	30	1.89447	0.0290811	-0.519399	1.33104	3
F6P	7	-0.310994	0.622097	-0.926769	0.666328	0
FDP	2	-1.68647	0.954147	-1.8748	0.591025	0
S7P	4	-0.586794	0.721329	-1.08175	0.841761	0
S17P	2	-1.68647	0.954147	-1.8748	0.591025	0
T3P2	4	-0.302543	0.618881	-0.956529	0.46396	0
T3P1	7	2.01587	0.0219066	-0.152486	1.78029	1
E4P	5	0.498837	0.308947	-0.626776	0.677448	0
NAD	39	0.554326	0.289678	-0.745367	0.626468	0

13PDG	2	-0.755933	0.775155	-1.29457	0.000300437	0
NADH	39	0.554326	0.289678	-0.745367	0.626468	0
3PG	3	0.226467	0.410419	-0.707994	0.601831	0
2PG	1	-0.534123	0.703372	-1.29457	0	0
23PDG	1	-0.534123	0.703372	-1.29457	0	0
PEP	2	0.47764	0.316453	-0.525368	1.24825	0
PYR	11	-0.23306	0.592143	-0.885132	0.740271	0
CO2	17	-0.122108	0.548593	-0.84936	0.738494	0
OA	4	2.29575	0.0108452	0.188068	1.81815	1
ATPm	8	-0.936635	0.825527	-1.11478	0.597033	0
PYRm	2	-0.642671	0.739781	-1.22394	0.386866	0
CO2m	4	-3.35075	0.999597	-2.29932	1.05023	0
ADPm	2	-0.0457987	0.518265	-0.85176	0.139479	0
PIm	2	0.63338	0.263243	-0.428255	0.738405	0
OAm	2	0.194057	0.423065	-0.702197	0.350993	0
GTP	3	0.296254	0.383518	-0.672483	0.340162	0
GDP	4	0.13253	0.447283	-0.764871	0.333591	0
NADP	28	-0.0546552	0.521793	-0.832408	0.629453	0
D6PGL	2	-0.661235	0.745769	-1.23552	0.908206	0

NADPH	28	-0.0546552	0.521793	-0.832408	0.629453	0
D6PGC	3	-1.02804	0.848034	-1.34636	0.482566	0
RL5P	3	2.27246	0.0115293	0.33313	0.553121	0
XUL5P	5	3.38835	0.000351573	0.511397	1.94792	1
R5P	8	1.33741	0.0905438	-0.40707	0.642584	0
ACTP	2	3.69839	0.000108486	1.48294	3.16229	1
ACCOAm	6	-1.59514	0.94466	-1.39672	1.335	0
H2Om	4	-1.2326	0.891137	-1.36624	0.576714	0
COAm	6	-1.59514	0.94466	-1.39672	1.335	0
NADm	10	-1.72913	0.958107	-1.30447	0.886714	0
AKGm	1	0.418204	0.337899	-0.454007	0	0
NADHm	10	-1.72913	0.958107	-1.30447	0.886714	0
AKG	8	2.93646	0.00165993	0.0905712	0.893446	0
NADPm	6	-0.278991	0.609874	-0.923571	0.591893	0
NADPHm	6	-0.278991	0.609874	-0.923571	0.591893	0
DHLIPOm	1	-2.78297	0.997307	-3.27948	0	0
Qm	3	-0.0537858	0.521447	-0.850603	0.555766	0
QH2m	3	-0.0537858	0.521447	-0.850603	0.555766	0
FADH2m	5	-0.279185	0.609949	-0.933237	1.03065	0

FADm	5	-0.279185	0.609949	-0.933237	1.03065	0
FUM	3	0.31742	0.375462	-0.661712	0.91584	0
SUCC	5	0.233221	0.407795	-0.731401	0.74399	0
MAL	1	0.092486	0.463156	-0.741497	0	0
MALg	1	-0.301969	0.618662	-1.08966	0	0
NADg	1	-0.301969	0.618662	-1.08966	0	0
OAg	1	-0.301969	0.618662	-1.08966	0	0
NADHg	1	-0.301969	0.618662	-1.08966	0	0
Hm	4	-0.646022	0.740867	-1.10784	0.610402	0
CIT	1	4.2161	4.04067e-005	2.89816	0	1
COA	21	0.0400661	0.48402	-0.815637	1.12268	1
ACCOA	17	0.748279	0.227146	-0.663725	1.25835	1
OXAL	1	0.863307	0.193984	-0.0611421	0	0
AC	12	-2.09088	0.981731	-1.35433	0.597974	0
FOR	1	0.863307	0.193984	-0.0611421	0	0
METHOL	2	-1.03463	0.84958	-1.46835	0.057194	0
FALD	4	-0.484509	0.685988	-1.03669	0.543746	0
ADHLIPOm	1	-2.78297	0.997307	-3.27948	0	0
ACAL	9	-1.05135	0.853452	-1.13173	0.800216	0

RGT	3	-0.924174	0.822302	-1.29351	0.234145	0
FGT	1	-0.685197	0.75339	-1.42791	0	0
H+	13	-0.407245	0.658086	-0.922668	0.575028	0
HCIT	1	0.760461	0.22349	-0.151918	0	0
MTHGXL	3	-0.280896	0.610605	-0.966171	0.688424	0
LACAL	4	-0.512542	0.695864	-1.04904	0.775808	0
LAC	3	-0.83884	0.79922	-1.25009	0.656247	0
LGT	2	-0.646468	0.741012	-1.22631	0.287319	0
LLAC	2	-0.148144	0.558885	-0.915577	0.246187	0
PROP	2	-1.25733	0.895684	-1.60722	1.16949	0
AMP	20	-0.328213	0.628625	-0.887846	0.924447	1
PPI	24	-0.592729	0.723319	-0.929675	0.518646	0
PROPCOA	2	-1.25733	0.895684	-1.60722	1.16949	0
GLU	18	1.62611	0.0519629	-0.486299	0.843148	0
GABA	1	-0.540911	0.705716	-1.30056	0	0
SUCCSAL	3	0.274521	0.391842	-0.683541	0.680462	0
METTHF	3	-1.20002	0.884935	-1.43388	0.849938	0
METHF	1	0.182325	0.427664	-0.662202	0	0
METTHFm	2	-0.248867	0.598268	-0.978384	0.447149	0

METHFm	1	0.182325	0.427664	-0.662202	0	0
FTHF	1	-0.783199	0.783245	-1.51441	0	0
THFm	1	-0.534123	0.703372	-1.29457	0	0
AHTD	1	-0.127165	0.550595	-0.935369	0	0
DHP	1	-0.127165	0.550595	-0.935369	0	0
CHOR	3	0.00683743	0.497272	-0.819755	0.738889	0
GLN	8	0.347475	0.364117	-0.715151	0.619102	0
DHF	1	-1.72408	0.957653	-2.34486	0	0
THF	3	-0.844804	0.80089	-1.25312	0.284286	0
THFG	1	-0.144179	0.55732	-0.950386	0	0
CTP	3	-0.413047	0.660214	-1.03342	0.760557	0
CYS	6	-0.356339	0.639207	-0.951377	0.937355	0
CMP	3	2.56594	0.00514484	0.482469	1.43264	1
ASP	11	-1.0591	0.855223	-1.10427	0.518598	0
PAP	1	-1.49561	0.932623	-2.14321	0	0
ACP	4	0.805233	0.210343	-0.468532	0.354925	0
ALA	2	0.232606	0.408034	-0.678159	0.128524	0
ETH	2	-1.03463	0.84958	-1.46835	0.057194	0
ETHm	2	-1.03463	0.84958	-1.46835	0.057194	0

ACALm	5	-1.31482	0.905715	-1.34117	0.477014	0
ACm	7	-1.57538	0.942415	-1.3475	0.689817	0
AMPm	6	-1.05477	0.854234	-1.20246	0.676935	0
PPIm	7	-0.582953	0.720038	-1.01727	0.788629	0
ACTPm	1	0.079303	0.468396	-0.753133	0	0
GLYN	6	0.173819	0.431004	-0.76079	0.428508	0
GL	7	-1.01116	0.844029	-1.15975	0.541312	0
GLYAL	5	-1.02044	0.846241	-1.22522	0.275082	0
O2	20	0.149745	0.440483	-0.793883	0.67638	0
H2O2	4	-0.13584	0.554026	-0.883093	0.471466	0
GL3P	4	-0.512526	0.695859	-1.04903	0.610662	0
TAR	2	-0.112033	0.544601	-0.89306	0.470099	0
OXGLY	2	-0.112033	0.544601	-0.89306	0.470099	0
G	1	0.0970923	0.461327	-0.737431	0	0
E	3	0.104091	0.458548	-0.770266	0.562826	0
EOL	3	0.104091	0.458548	-0.770266	0.562826	0
LXUL	5	-0.122983	0.54894	-0.871709	0.559792	0
XOL	3	0.142093	0.443503	-0.750929	0.511834	0
XUL	4	0.0682012	0.472813	-0.793209	0.42638	0

AOL	3	0.142093	0.443503	-0.750929	0.511834	0
XYL	1	0.734186	0.231418	-0.175109	0	0
LAOL	3	0.123697	0.450778	-0.76029	0.752915	0
RIB	1	-0.568827	0.715263	-1.3252	0	0
R1P	1	2.02937	0.0212101	0.968073	0	0
RL	2	1.34529	0.0892658	0.0156587	0.0845979	0
H2Oe	8	-0.482983	0.685446	-0.9736	0.670596	0
GLCNT	2	-0.412926	0.660169	-1.08068	0.205505	0
GLAC	10	0.342297	0.366064	-0.728046	0.654406	0
GALOL	3	0.104091	0.458548	-0.770266	0.562826	0
GAL1P	1	-0.285774	0.612474	-1.07536	0	0
UTP	5	-0.812623	0.791783	-1.14336	0.563517	0
UDPGAL	3	-0.411155	0.65952	-1.03245	0.715397	0
UDPG	6	-0.994825	0.840089	-1.18091	0.669772	0
G1P	4	0.494119	0.310611	-0.605584	1.07916	0
MELI	3	-1.14198	0.873268	-1.40434	0.403275	0
GALN14LAC	1	1.01809	0.154317	0.0754785	0	0
GALNT	1	0.707569	0.239607	-0.198602	0	0
2D3DGALT	1	0.707569	0.239607	-0.198602	0	0

SOR	4	-0.867693	0.807219	-1.20549	0.362256	0
SOT	3	-0.876396	0.809593	-1.2692	0.415322	0
MAN6P	2	0.195982	0.422312	-0.700997	0.558486	0
MAN	1	-0.309047	0.621357	-1.09591	0	0
FRU	3	-0.461084	0.677631	-1.05786	0.119933	0
MNT	2	-0.345819	0.635261	-1.03884	0.163084	0
F26P	1	0.0733327	0.470771	-0.758403	0	0
IDOL	1	-0.216666	0.585766	-1.01437	0	0
UDP	9	0.369709	0.3558	-0.714834	0.572436	0
TRE6P	2	0.507168	0.306018	-0.506955	0.0384998	0
TRE	1	0.389058	0.348617	-0.479732	0	0
MLT	3	-1.42827	0.923394	-1.55003	0.424125	0
MLTe	3	-1.42827	0.923394	-1.55003	0.424125	0
LACT	3	1.07271	0.1417	-0.277375	0.362624	0
LACTe	3	1.07271	0.1417	-0.277375	0.362624	0
GLACe	3	1.07271	0.1417	-0.277375	0.362624	0
GA6P	2	-1.54693	0.93906	-1.78779	0.978477	0
NAGA6P	1	-1.87682	0.969729	-2.47968	0	0
NAGA1P	1	-0.186508	0.573977	-0.987748	0	0

UDPNAG	6	0.66153	0.254136	-0.585461	0.371495	0
CHIT	7	-0.178323	0.570765	-0.882622	0.742381	0
NAG	2	-1.60927	0.946221	-1.82667	0.561715	0
GLCN	1	-0.309047	0.621357	-1.09591	0	0
13GLUCANe	7	0.106722	0.457505	-0.787771	0.571636	0
GLYCOGEN	2	-0.958456	0.831084	-1.42085	0.805296	0
CELLUe	4	-0.0380438	0.515174	-0.840012	0.435875	0
CELLOBe	4	-0.0380438	0.515174	-0.840012	0.435875	0
CELLOTe	2	-0.529382	0.70173	-1.1533	0.0977921	0
MANNANe	3	-0.567325	0.714753	-1.11192	0.78748	0
MANe	3	-0.567325	0.714753	-1.11192	0.78748	0
PECTATEe	1	0.638448	0.261591	-0.259611	0	0
GALUNTe	1	0.638448	0.261591	-0.259611	0	0
ARABINe	4	0.0775065	0.46911	-0.78911	0.615789	0
LARABe	4	0.0775065	0.46911	-0.78911	0.615789	0
XYLANe	6	-0.692372	0.755648	-1.07218	1.02836	0
XYLe	6	-0.692372	0.755648	-1.07218	1.02836	0
FERIm	1	-0.764038	0.777578	-1.4975	0	0
FEROm	1	-0.764038	0.777578	-1.4975	0	0

LLACm	3	-0.851217	0.802676	-1.25638	0.653063	0
LACm	1	-0.764038	0.777578	-1.4975	0	0
GLUm	4	0.663014	0.253661	-0.531183	0.279538	0
ASPm	3	0.0173556	0.493076	-0.814402	0.535284	0
ASN	4	-1.10762	0.865988	-1.31118	0.739531	0
SAM	6	0.197664	0.421654	-0.752217	0.637296	0
HCYS	6	-0.657375	0.74453	-1.0596	0.728359	0
SAH	3	0.210654	0.416579	-0.716041	0.81337	0
MET	2	-0.234053	0.592528	-0.969146	0.554513	0
TRNAm	3	-0.236646	0.593534	-0.943654	0.443736	0
ASPTRNAm	2	-0.274874	0.608293	-0.9946	0.615004	0
TRNA	2	-0.274874	0.608293	-0.9946	0.615004	0
ASPTRNA	2	-0.274874	0.608293	-0.9946	0.615004	0
NH3	15	2.31074	0.0104236	-0.298566	1.32616	1
NAGLUm	1	0.29738	0.383088	-0.56065	0	0
NAORNm	1	0.29738	0.383088	-0.56065	0	0
ORNm	1	0.29738	0.383088	-0.56065	0	0
CAP	1	-0.540911	0.705716	-1.30056	0	0
ORN	3	0.618779	0.268031	-0.508362	1.38854	0

CITR	1	-0.224805	0.588934	-1.02155	0	0
GLUGSAL	2	0.710694	0.238637	-0.380046	0.593423	0
ARGSUCC	2	0.764036	0.222423	-0.346784	0.954263	0
ARG	3	1.59388	0.0554815	-0.0121732	0.763944	0
PTRSC	2	-1.2362	0.891808	-1.59404	0.697531	0
DSAM	1	-0.314602	0.623468	-1.10081	0	0
SPRMD	2	-0.211136	0.58361	-0.954857	0.206407	0
5MTA	1	-0.314602	0.623468	-1.10081	0	0
SPRM	2	-0.211136	0.58361	-0.954857	0.206407	0
GBAD	1	-0.314602	0.623468	-1.10081	0	0
GBAT	1	-0.314602	0.623468	-1.10081	0	0
UREA	3	1.06432	0.143591	-0.281643	1.27753	0
ATRNA	1	-0.0724922	0.528895	-0.887113	0	0
ALTRNA	1	-0.0724922	0.528895	-0.887113	0	0
SER	8	-0.395545	0.65378	-0.946388	0.787279	0
ASER	5	-1.31651	0.905999	-1.34184	0.512449	0
H2S	4	-1.04157	0.851194	-1.28208	0.560844	0
RTHIO	1	-0.248017	0.597939	-1.04204	0	0
OTHIO	1	-0.248017	0.597939	-1.04204	0	0

GLUGSALm	2	0.430059	0.333576	-0.555037	0.475881	0
P5Cm	3	0.520466	0.301369	-0.55839	0.336549	0
PHP	1	0.828363	0.203733	-0.0919849	0	0
GLYm	1	-0.534123	0.703372	-1.29457	0	0
GLY	5	-0.0897275	0.535748	-0.85861	0.899399	0
BASP	1	0.579375	0.281168	-0.311751	0	0
ASPSA	2	0.468508	0.319711	-0.531062	0.310153	0
HSER	3	0.7931	0.21386	-0.419657	0.418707	0
PHSER	2	0.697289	0.242811	-0.388405	0.621593	0
THR	6	0.599944	0.274272	-0.607601	0.903461	0
LLCT	5	0.251816	0.400592	-0.724077	1.14235	0
OBUT	4	1.83002	0.0336234	-0.0170932	0.834254	0
THRm	3	0.807082	0.20981	-0.412543	0.800167	0
NH3m	3	0.807082	0.20981	-0.412543	0.800167	0
OBUTm	3	0.807082	0.20981	-0.412543	0.800167	0
PRPP	5	0.190093	0.424618	-0.748389	0.339496	0
PRBATP	2	0.650733	0.257609	-0.417435	0.474749	0
PRBAMP	2	0.650733	0.257609	-0.417435	0.474749	0
PRFP	3	0.258966	0.397831	-0.691457	0.581341	0

PRLP	2	-0.218208	0.586367	-0.959266	0.396312	0
DIMGP	1	0.163258	0.435158	-0.679031	0	0
IMACP	1	1.09827	0.136043	0.146248	0	0
HISOLP	2	1.01371	0.155362	-0.191102	0.477084	0
HISOL	3	0.724745	0.234304	-0.45444	0.341762	0
HIS	3	0.00632198	0.497478	-0.820017	0.773893	0
AICAR	3	-0.788043	0.784664	-1.22424	0.472489	0
HTRNA	1	-0.9087	0.818246	-1.62518	0	0
HHTRNA	1	-0.9087	0.818246	-1.62518	0	0
VAL	2	-0.225171	0.589077	-0.963608	0.275162	0
CBHCAP	1	-1.26711	0.897443	-1.94153	0	0
IPPMAL	1	-1.26711	0.897443	-1.94153	0	0
PPMAL	1	-1.26711	0.897443	-1.94153	0	0
HACNm	1	-0.823888	0.794998	-1.55032	0	0
HICITm	2	-2.62453	0.995662	-2.45974	1.28611	0
OXAm	1	-2.88456	0.99804	-3.36915	0	0
MICIT	1	-0.880541	0.810717	-1.60033	0	0
AMA	2	-0.034229	0.513653	-0.844545	0.106782	0
AMASA	1	-0.10981	0.54372	-0.920051	0	0

SACP	1	1.27879	0.100486	0.305575	0	0
LYS	2	0.627562	0.265145	-0.431883	1.04292	0
LTRNA	1	-0.392248	0.652563	-1.16934	0	0
LLTRNA	1	-0.392248	0.652563	-1.16934	0	0
LYSm	1	-0.392248	0.652563	-1.16934	0	0
LTRNAm	1	-0.392248	0.652563	-1.16934	0	0
LLTRNAm	1	-0.392248	0.652563	-1.16934	0	0
ADN	4	4.00913	9.93931e-005	0.942848	1.96194	2
OAHSER	2	0.40865	0.341398	-0.568387	0.0122476	0
METH	1	0.278803	0.390198	-0.577047	0	0
OSLHSER	1	0.925717	0.177296	-0.00605609	0	0
CALH	1	-0.182828	0.572533	-0.984499	0	0
DPTH	1	-0.182828	0.572533	-0.984499	0	0
3DDAH7P	1	1.33736	0.0905526	0.357275	0	0
DQT	1	-0.274288	0.608068	-1.06523	0	0
DHSK	1	-0.274288	0.608068	-1.06523	0	0
3PSME	1	-0.701002	0.758349	-1.44186	0	0
PHEN	2	-0.487766	0.687142	-1.12735	0.159783	0
PHPYR	2	1.59669	0.0551674	0.17242	1.98758	0

PHE	1	2.72023	0.0032618	1.57785	0	0
4HPP	4	1.266	0.102756	-0.265554	1.41398	0
TYR	2	1.38461	0.0830859	0.0401767	2.1746	0
AN	3	0.678613	0.248691	-0.477915	0.414745	0
NPRAN	1	0.18293	0.427427	-0.661668	0	0
IGP	1	0.931789	0.175723	-0.000696652	0	0
TRP	3	0.598512	0.274749	-0.518675	0.523237	0
FKYN	2	0.0730281	0.470892	-0.777665	0.380921	0
KYN	1	0.0612816	0.475567	-0.769039	0	0
HKYN	1	0.0612816	0.475567	-0.769039	0	0
HAN	1	0.0612816	0.475567	-0.769039	0	0
CMUSA	1	0.268647	0.394101	-0.586011	0	0
AM6SA	2	0.258968	0.39783	-0.661721	0.10707	0
AMUCO	1	0.0970923	0.461327	-0.737431	0	0
HOMOGEN	2	0.0878708	0.46499	-0.76841	1.22496	0
MACAC	2	-0.227804	0.590101	-0.96525	0.946584	0
FUACAC	2	0.414274	0.339337	-0.56488	0.380375	0
ACTAC	1	-0.0121417	0.504844	-0.833845	0	0
TRPm	1	-0.0211091	0.508421	-0.841761	0	0

TRPTRNAm	1	-0.0211091	0.508421	-0.841761	0	0
ASPERMD	2	0.0849591	0.466147	-0.770225	0.0547008	0
APRUT	1	0.103761	0.45868	-0.731546	0	0
APROA	1	0.103761	0.45868	-0.731546	0	0
GABAL	1	0.103761	0.45868	-0.731546	0	0
ASPRM	2	0.0849591	0.466147	-0.770225	0.0547008	0
GLUP	2	0.546418	0.292389	-0.482481	0.448558	0
PROm	1	0.292342	0.385013	-0.565097	0	0
GABALm	3	-0.851217	0.802676	-1.25638	0.653063	0
GABAm	3	-0.851217	0.802676	-1.25638	0.653063	0
LACALm	3	-0.851217	0.802676	-1.25638	0.653063	0
APROP	1	0.26721	0.394654	-0.587279	0	0
TCOA	1	-0.101349	0.540363	-0.912583	0	0
GLP	1	-0.101349	0.540363	-0.912583	0	0
TGLP	1	-0.101349	0.540363	-0.912583	0	0
GC	1	-0.412075	0.659858	-1.18684	0	0
GMP	3	2.51139	0.00601282	0.454711	1.46608	1
DGMP	2	3.00513	0.00132734	1.05066	1.47237	1
DGDP	1	-0.248017	0.597939	-1.04204	0	0

DADP	1	-0.248017	0.597939	-1.04204	0	0
PRAM	1	-0.248017	0.597939	-1.04204	0	0
GAR	1	-0.248017	0.597939	-1.04204	0	0
FGAR	1	0.432334	0.332749	-0.441535	0	0
FGAM	2	0.130567	0.448059	-0.741786	0.42462	0
AIR	2	0.27302	0.392419	-0.652959	0.55024	0
CAIR	2	0.214177	0.415204	-0.689651	0.60213	0
SAICAR	2	-0.760391	0.776489	-1.29735	0.25728	0
PRFICA	1	-0.783199	0.783245	-1.51441	0	0
IMP	5	1.49506	0.0674492	-0.234365	1.41257	1
ASUC	2	-0.685119	0.753366	-1.25041	0.323657	0
XMP	3	2.51139	0.00601282	0.454711	1.46608	1
DAMP	2	3.00513	0.00132734	1.05066	1.47237	1
ATN	1	-0.520147	0.698519	-1.28223	0	0
ATT	2	-1.11503	0.867582	-1.51848	0.334114	0
UGC	1	-1.05548	0.854398	-1.75474	0	0
CAASP	1	-0.540911	0.705716	-1.30056	0	0
DOROA	1	-0.685197	0.75339	-1.42791	0	0
OROA	1	-0.685197	0.75339	-1.42791	0	0

UMP	4	2.16735	0.015104	0.131508	1.36418	1
URA	4	0.441969	0.329256	-0.628557	0.286173	0
URI	3	2.56594	0.00514484	0.482469	1.43264	1
CYTD	3	2.56594	0.00514484	0.482469	1.43264	1
DU	2	3.00513	0.00132734	1.05066	1.47237	1
DT	2	3.00513	0.00132734	1.05066	1.47237	1
DC	2	3.00513	0.00132734	1.05066	1.47237	1
DTMP	4	1.12608	0.130066	-0.327193	1.87799	1
DTDP	1	-0.274288	0.608068	-1.06523	0	0
DUMP	3	1.45826	0.072384	-0.0811829	2.21971	1
DCMP	2	3.00513	0.00132734	1.05066	1.47237	1
DCDP	1	-0.248017	0.597939	-1.04204	0	0
CDP	2	0.210389	0.416682	-0.692013	0.49501	0
PURI5P	3	0.574386	0.282853	-0.530952	0.256285	0
AD	2	2.65934	0.00391472	0.835038	3.05503	1
INS	3	4.95637	1.15734e-006	1.69886	1.53116	2
DA	3	4.95637	1.15734e-006	1.69886	1.53116	2
DIN	1	4.32613	2.46311e-005	2.99527	0	1
HYXN	2	3.13084	0.000871531	1.12905	2.63924	1

DG	2	3.00513	0.00132734	1.05066	1.47237	1
GN	2	-0.333552	0.630641	-1.03119	0.415789	0
GSN	3	2.12621	0.016743	0.258706	1.72206	1
XAN	1	0.0973756	0.461214	-0.737181	0	0
XTSINE	2	3.00513	0.00132734	1.05066	1.47237	1
DUDP	1	-0.248017	0.597939	-1.04204	0	0
LCCA	1	-0.716558	0.763177	-1.45559	0	0
ACOA	2	-0.993227	0.8397	-1.44253	0.0184708	0
HACOA	1	0.348181	0.363852	-0.515812	0	0
OACOA	2	-0.23966	0.594703	-0.972643	0.646057	0
AACCOA	2	0.633602	0.26317	-0.428117	1.12879	0
AACCOAm	1	1.35184	0.0882125	0.37006	0	0
ACACPm	1	-2.27363	0.988506	-2.82992	0	0
MALACPm	1	-2.27363	0.988506	-2.82992	0	0
ACPm	1	-2.27363	0.988506	-2.82992	0	0
MALCOA	5	0.818646	0.206494	-0.500803	0.589876	0
MALACP	2	0.121002	0.451845	-0.747751	0.11812	0
ACACP	2	1.01657	0.154678	-0.189314	0.228278	0
3OACPm	1	-2.27363	0.988506	-2.82992	0	0

C120ACP	4	-0.258845	0.602122	-0.937279	0.332056	0
C140ACP	4	-0.258845	0.602122	-0.937279	0.332056	0
C141ACP	4	-0.258845	0.602122	-0.937279	0.332056	0
C160ACP	4	-0.258845	0.602122	-0.937279	0.332056	0
C161ACP	4	-0.258845	0.602122	-0.937279	0.332056	0
C180ACP	4	-0.258845	0.602122	-0.937279	0.332056	0
C181ACP	4	-0.258845	0.602122	-0.937279	0.332056	0
C182ACP	4	-0.258845	0.602122	-0.937279	0.332056	0
3HPACP	2	1.01657	0.154678	-0.189314	0.228278	0
2HDACP	2	1.01657	0.154678	-0.189314	0.228278	0
AACP	2	1.01657	0.154678	-0.189314	0.228278	0
23DAACP	2	1.01657	0.154678	-0.189314	0.228278	0
C150ACP	3	-0.538582	0.704912	-1.0973	0.108435	0
C162ACP	4	-0.258845	0.602122	-0.937279	0.332056	0
C170ACP	4	-0.258845	0.602122	-0.937279	0.332056	0
C183ACP	4	-0.258845	0.602122	-0.937279	0.332056	0
C200ACP	4	-0.258845	0.602122	-0.937279	0.332056	0
AGL3P	1	-0.865075	0.806501	-1.58668	0	0
AT3P2	1	-0.865075	0.806501	-1.58668	0	0

PA	1	-0.960861	0.831689	-1.67122	0	0
PS	3	0.425326	0.3353	-0.606803	0.824198	0
PSm	1	-1.38756	0.917364	-2.04784	0	0
PE	4	0.576239	0.282227	-0.569409	0.677098	0
PEm	1	-1.38756	0.917364	-2.04784	0	0
PC	1	0.414555	0.339234	-0.457227	0	0
CHO	1	-0.768355	0.778862	-1.50131	0	0
PCHO	1	-0.768355	0.778862	-1.50131	0	0
DAGLY	5	-1.22134	0.889021	-1.30435	0.296387	0
PETHM	1	-0.0986553	0.539294	-0.910206	0	0
CDPETN	1	-0.0986553	0.539294	-0.910206	0	0
PINS	2	-0.0682537	0.527208	-0.865761	0.867633	0
PINSP	1	0.646785	0.258886	-0.252252	0	0
PINS4P	1	-0.743388	0.771377	-1.47927	0	0
D45PI	1	-0.833299	0.797662	-1.55863	0	0
TPI	1	-0.833299	0.797662	-1.55863	0	0
LPC	1	0.414555	0.339234	-0.457227	0	0
LPE	1	0.414555	0.339234	-0.457227	0	0
SPH	1	-1.29504	0.902347	-1.96618	0	0

PSPH	1	-0.24523	0.596861	-1.03958	0	0
C260COA	1	-0.24523	0.596861	-1.03958	0	0
CER2	1	-0.24523	0.596861	-1.03958	0	0
DHSP	1	-1.29504	0.902347	-1.96618	0	0
H3MCOA	2	-0.952785	0.829651	-1.41731	0.270143	0
MVL	2	-0.243699	0.596268	-0.975161	0.895442	0
PMVL	2	0.243082	0.403971	-0.671627	0.46618	0
PPMVL	2	-0.127926	0.550896	-0.90297	0.139012	0
IPPP	2	-0.0168595	0.506726	-0.833714	0.0410699	0
DMPP	1	-0.0448955	0.517905	-0.862755	0	0
LNST	3	-0.597464	0.724901	-1.12726	1.3989	0
IGST	4	0.585685	0.279044	-0.565248	1.60251	0
DMZYMST	2	1.48114	0.0692846	0.100368	1.44309	0
IMZYMST	1	-0.10981	0.54372	-0.920051	0	0
MZYMST	1	-0.10981	0.54372	-0.920051	0	0
IZYMST	1	-0.10981	0.54372	-0.920051	0	0
EPST	1	-0.285774	0.612474	-1.07536	0	0
ERTROL	1	-0.285774	0.612474	-1.07536	0	0
ERTEOL	1	2.2024	0.0138187	1.12079	0	0

ERGOST	1	2.2024	0.0138187	1.12079	0	0
TAGLY	3	-0.538582	0.704912	-1.0973	0.108435	0
MAGLY	3	-0.538582	0.704912	-1.0973	0.108435	0
PHACAL	4	-0.969962	0.833967	-1.25054	0.533352	0
PHAC	4	-0.464296	0.678782	-1.02778	0.702394	0
PHACCOA	1	0.545117	0.292836	-0.341988	0	0
LLDACV	2	0.191815	0.423944	-0.703595	0.0925525	0
IPN	1	0.209575	0.417	-0.63815	0	0
NOR	3	0.421191	0.336808	-0.608907	1.14586	0
AVN	4	-0.570078	0.715687	-1.07438	0.521464	0
HAVN	4	0.373351	0.354444	-0.658785	0.439827	0
AVF	2	0.713233	0.237851	-0.378463	0.385042	0
VHA	2	-0.130303	0.551837	-0.904452	0.35882	0
VERB	1	0.13488	0.446353	-0.704078	0	0
VERA	1	0.13488	0.446353	-0.704078	0	0
DMST	1	0.13488	0.446353	-0.704078	0	0
DHDMST	1	0.13488	0.446353	-0.704078	0	0
ST	1	1.15648	0.123743	0.197623	0	0
DHST	1	1.15648	0.123743	0.197623	0	0

OMST	1	1.15648	0.123743	0.197623	0	0
DHOMST	1	1.15648	0.123743	0.197623	0	0
HNO3	3	0.24748	0.402268	-0.697301	0.656966	0
HNO2	1	0.917566	0.179423	-0.0132504	0	0
UREAC	1	1.37126	0.0851468	0.387197	0	0
ACNL	1	0.26721	0.394654	-0.587279	0	0
INAC	1	0.26721	0.394654	-0.587279	0	0
HNO3e	2	-0.346602	0.635555	-1.03933	0.401648	0

#Results for Down-regulated only genes

#Feature	Number of neighbors	Z-score	P-value	Average Z	StdDev Z	Significance count
DGLCe	2	-0.247269	0.59765	-0.977387	0.582456	0
GLCe	8	0.449625	0.32649	-0.683361	0.718434	0
bDGLCe	3	0.00571144	0.497721	-0.820328	0.39097	0
DGLC	2	2.14474	0.0159867	0.51416	1.43461	0
GLC	7	0.667727	0.252154	-0.601092	0.275033	0
bDGLC	3	0.686049	0.246341	-0.474132	0.0287496	0
ATP	42	-0.588964	0.722057	-0.903162	0.660063	0
ADP	28	0.0337424	0.486541	-0.817727	0.608488	0

G6P	5	-0.0797394	0.531778	-0.854676	0.814023	0
bDG6P	3	0.144517	0.442546	-0.749695	0.463584	0
H2O	38	-0.0424505	0.51693	-0.829387	1.15285	1
PI	28	1.1074	0.134061	-0.639416	1.25727	1
F6P	7	0.0495525	0.48024	-0.806795	1.03109	0
FDP	1	0.570746	0.284086	-0.319367	0	0
S7P	2	-0.404335	0.657017	-1.07533	1.06909	0
S17P	1	0.570746	0.284086	-0.319367	0	0
T3P2	3	1.00572	0.157275	-0.311464	0.969302	0
T3P1	4	-0.574415	0.717156	-1.07629	1.29662	0
E4P	2	-1.4203	0.92224	-1.70884	0.173171	0
NAD	32	-1.07931	0.859775	-0.990977	0.806303	0
13PDG	4	-0.640119	0.738952	-1.10524	1.20623	0
NADH	32	-1.07931	0.859775	-0.990977	0.806303	0
3PG	2	0.0453985	0.481895	-0.794893	1.33843	0
2PG	2	1.68562	0.0459347	0.227871	0.107978	0
23PDG	1	1.10425	0.134743	0.151519	0	0
PEP	4	-0.606817	0.728014	-1.09057	0.95915	0
PYR	6	-0.670854	0.748843	-1.06444	0.67442	0

CO2	23	1.00077	0.15847	-0.639896	0.630581	0
OA	8	-1.04394	0.851744	-1.14818	0.70456	0
ATPm	11	0.0320329	0.487223	-0.814805	0.795378	0
PYRm	7	0.031167	0.487568	-0.812913	0.641919	0
CO2m	9	-0.241583	0.595448	-0.894168	0.587209	0
ADPm	9	0.54189	0.293947	-0.664321	0.800576	0
PIm	10	0.710317	0.238754	-0.625635	0.711889	0
OAm	8	-0.273701	0.607843	-0.908469	0.64075	0
GTP	7	0.170893	0.432154	-0.766418	0.631763	0
GDP	3	-0.8279	0.796136	-1.24452	0.548618	0
NADP	35	0.306859	0.379475	-0.777767	0.872335	0
NADPH	35	0.306859	0.379475	-0.777767	0.872335	0
D6PGC	4	-0.249966	0.598693	-0.933368	0.39356	0
RL5P	2	-0.570261	0.71575	-1.17879	0.18973	0
XUL5P	1	-1.14221	0.873316	-1.83129	0	0
R5P	7	-0.744845	0.771817	-1.07114	0.435162	0
ACCOAm	5	0.602973	0.273263	-0.585757	0.427318	0
H2Om	12	0.560158	0.287686	-0.681043	0.701105	0
CITm	5	0.582575	0.28009	-0.593791	0.675366	0

COAm	7	0.617157	0.268566	-0.61792	0.606539	0
ACOm	3	0.195732	0.42241	-0.723634	0.78128	0
ICITm	5	0.306728	0.379525	-0.702447	0.747166	0
NADm	12	-1.99635	0.977052	-1.33032	0.670731	0
AKGm	7	0.444038	0.328508	-0.675526	0.603534	0
NADHm	12	-1.99635	0.977052	-1.33032	0.670731	0
ICIT	2	0.244622	0.403375	-0.670667	1.00442	0
AKG	10	0.178602	0.429125	-0.773598	0.826892	0
NADPm	10	-0.704465	0.759429	-1.01933	0.738554	0
NADPHm	10	-0.704465	0.759429	-1.01933	0.738554	0
ICITg	2	-0.00788078	0.503144	-0.828116	1.22709	0
NADPg	1	0.977407	0.164184	0.0395674	0	0
AKGg	1	0.977407	0.164184	0.0395674	0	0
CO2g	1	0.977407	0.164184	0.0395674	0	0
NADPHg	1	0.977407	0.164184	0.0395674	0	0
LIPOm	2	0.106125	0.457742	-0.757027	0.0277124	0
SUCDLIP	2	-0.515925	0.697047	-1.14491	0.57626	0
SUCCOAm	2	0.200262	0.420638	-0.698327	1.20782	0
DHLIPOm	1	-0.826225	0.795662	-1.55239	0	0

GDPm	2	-0.298622	0.617386	-1.00941	1.64776	0
GTPm	2	-0.298622	0.617386	-1.00941	1.64776	0
SUCCm	3	-0.72135	0.764653	-1.1903	1.1657	0
Qm	6	-0.274314	0.608078	-0.92189	0.550946	0
FUMm	3	-1.51956	0.935689	-1.59648	0.462176	0
QH2m	6	-0.274314	0.608078	-0.92189	0.550946	0
FADH2m	1	-1.1785	0.880701	-1.86331	0	0
FADm	1	-1.1785	0.880701	-1.86331	0	0
FUM	2	-0.727192	0.766446	-1.27664	0.30242	0
SUCC	1	-1.71524	0.956849	-2.33706	0	0
MALm	4	-0.527655	0.701131	-1.0557	0.736805	0
MAL	3	-0.800864	0.788395	-1.23076	0.794	0
MALg	1	-0.538744	0.704968	-1.29864	0	0
SUCCg	1	-0.988707	0.838597	-1.6958	0	0
GLXg	2	-1.08093	0.860137	-1.49722	0.280831	0
ACCOAg	2	-0.370015	0.644314	-1.05393	0.346084	0
H2Og	1	-0.538744	0.704968	-1.29864	0	0
COAg	2	-0.370015	0.644314	-1.05393	0.346084	0
Hm	7	-1.03217	0.849004	-1.16675	0.79634	0

COA	15	0.842623	0.19972	-0.631962	0.588336	0
ACCOA	6	0.96968	0.166103	-0.474684	0.485785	0
CAR	2	1.83816	0.0330192	0.322992	1.27564	0
ACAR	2	1.83816	0.0330192	0.322992	1.27564	0
ACARm	2	1.65359	0.0491053	0.207901	1.43841	0
CARm	2	1.65359	0.0491053	0.207901	1.43841	0
ACARg	2	1.65359	0.0491053	0.207901	1.43841	0
CARg	2	1.65359	0.0491053	0.207901	1.43841	0
OXAL	2	-0.617735	0.731625	-1.20839	0.424829	0
AC	5	-1.93219	0.973332	-1.58435	0.174313	0
FOR	6	-1.13607	0.872036	-1.23168	0.415981	0
FORm	2	0.861454	0.194494	-0.286039	0.591197	0
METHOL	2	-1.72143	0.957413	-1.8966	0.942293	0
FALD	4	-2.18692	0.985626	-1.78664	0.572742	0
ADHLIPOm	1	0.0526897	0.47899	-0.776623	0	0
ACAL	4	-2.16262	0.984715	-1.77593	0.591478	0
RGT	4	-0.832165	0.797342	-1.18984	0.358525	0
FGT	1	-1.00125	0.841647	-1.70687	0	0
H+	12	-1.04181	0.851249	-1.08789	0.946474	0

LACAL	1	-0.685197	0.75339	-1.42791	0	0
LAC	1	-0.685197	0.75339	-1.42791	0	0
LLAC	1	-0.613746	0.730309	-1.36484	0	0
AMP	17	-0.705184	0.759652	-0.973719	0.693161	0
PPI	20	-1.17455	0.879912	-1.05423	0.729811	0
PROPCOA	1	1.03393	0.150584	0.089458	0	0
2MCIT	1	1.03393	0.150584	0.089458	0	0
GLU	15	-0.446707	0.672457	-0.924756	0.736457	0
GABA	2	-1.38688	0.91726	-1.68799	0.992183	0
SUCCSAL	2	-2.4699	0.993242	-2.36332	0.0371334	0
METTHF	3	-0.632059	0.736326	-1.14486	0.599022	0
METHF	1	1.08213	0.139597	0.132	0	0
METTHFm	4	0.0759155	0.469743	-0.789811	0.694911	0
MTHFm	2	-0.571582	0.716198	-1.17961	0.524262	0
METHFm	1	1.08213	0.139597	0.132	0	0
FTHFm	1	1.08213	0.139597	0.132	0	0
FTHF	1	1.08213	0.139597	0.132	0	0
THFm	2	0.678681	0.24867	-0.400008	0.752374	0
AHTD	1	-0.182828	0.572533	-0.984499	0	0

DHP	1	0.971581	0.165629	0.0344253	0	0
AHHMP	1	0.971581	0.165629	0.0344253	0	0
GLAL	1	0.971581	0.165629	0.0344253	0	0
GLN	5	-0.519674	0.698355	-1.02796	0.770546	0
PABA	1	0.971581	0.165629	0.0344253	0	0
AHHMD	1	0.971581	0.165629	0.0344253	0	0
DHPT	1	0.971581	0.165629	0.0344253	0	0
THF	5	-0.335744	0.631468	-0.955515	0.578376	0
MTHF	1	0.646785	0.258886	-0.252252	0	0
THFG	1	-0.303188	0.619127	-1.09073	0	0
OIVAL	1	-1.13083	0.870937	-1.82124	0	0
AKP	1	-1.13083	0.870937	-1.82124	0	0
PANT	1	-0.944489	0.82754	-1.65677	0	0
AKPm	1	-0.208233	0.582476	-1.00692	0	0
PANTm	1	-0.208233	0.582476	-1.00692	0	0
bALA	1	-0.944489	0.82754	-1.65677	0	0
PNTO	2	-0.282537	0.611234	-0.999379	0.929691	0
4PPNTO	1	0.545117	0.292836	-0.341988	0	0
CTP	4	-0.274595	0.608186	-0.944218	0.715832	0

CYS	1	0.917382	0.179471	-0.0134134	0	0
CMP	5	1.1941	0.116218	-0.352911	0.34025	0
ASP	7	-0.736479	0.769281	-1.06835	0.258436	0
PAP	2	-0.553436	0.710017	-1.1683	2.34989	0
ACP	5	1.00567	0.157288	-0.427137	0.494721	0
ALA	4	0.545955	0.292548	-0.582749	0.54047	0
CHCOA	1	0.116229	0.453736	-0.720541	0	0
AONA	1	0.116229	0.453736	-0.720541	0	0
DTB	1	0.917382	0.179471	-0.0134134	0	0
BT	1	0.917382	0.179471	-0.0134134	0	0
ETH	2	-1.72143	0.957413	-1.8966	0.942293	0
ETHm	2	-1.72143	0.957413	-1.8966	0.942293	0
ACALm	4	-2.16262	0.984715	-1.77593	0.591478	0
ACm	2	-1.33438	0.90896	-1.65526	0.321518	0
AMPm	3	-1.76133	0.960909	-1.7195	0.440763	0
PPIm	4	-0.548411	0.708295	-1.06484	0.606871	0
GLYN	5	-0.812168	0.791652	-1.14318	1.01848	0
GL	7	-0.815015	0.792468	-1.09449	0.999702	0
GLYAL	4	-0.376141	0.646594	-0.988951	1.18177	0

O2	14	1.05662	0.145343	-0.574919	1.46082	1
H2O2	6	1.14985	0.125103	-0.409914	1.99822	1
GL3P	3	0.78243	0.216981	-0.425087	0.484821	0
E	3	1.33639	0.0907104	-0.143198	0.121351	0
EOL	3	1.33639	0.0907104	-0.143198	0.121351	0
LXUL	1	-0.139953	0.555651	-0.946656	0	0
XOL	3	-1.03461	0.849575	-1.34971	0.3492	0
XUL	3	-1.03461	0.849575	-1.34971	0.3492	0
AOL	3	-1.03461	0.849575	-1.34971	0.3492	0
XYL	2	-1.16755	0.878505	-1.55123	0.0144327	0
ARAB	2	0.995315	0.159791	-0.202569	0.0911173	0
ARABLAC	2	0.995315	0.159791	-0.202569	0.0911173	0
RIB	2	0.325321	0.372469	-0.620347	0.0584371	0
R1P	1	0.180294	0.428461	-0.663994	0	0
O2e	7	0.98218	0.163005	-0.496455	1.84332	1
GLCN15LACe	3	-0.193333	0.576651	-0.921613	0.270917	0
H2O2e	7	0.98218	0.163005	-0.496455	1.84332	1
H2Oe	11	1.02913	0.151709	-0.550282	1.44762	1
GLCNTe	1	0.0699478	0.472118	-0.76139	0	0

GLCN15LAC	1	0.0699478	0.472118	-0.76139	0	0
GLCNT	4	-0.0892242	0.535548	-0.862558	0.392347	0
GLAC	8	0.135687	0.446034	-0.781062	0.543969	0
GALOL	2	1.08647	0.138635	-0.145727	0.171505	0
GAL1P	1	-1.02601	0.847557	-1.72873	0	0
UTP	2	-0.0241823	0.509646	-0.838281	0.468036	0
UDPGAL	1	-0.280698	0.610529	-1.07088	0	0
UDPG	4	-0.39291	0.652807	-0.996338	0.792421	0
MELI	1	-0.10981	0.54372	-0.920051	0	0
GALN14LAC	2	-0.434708	0.668113	-1.09427	0.0471386	0
GALNT	1	-0.1567	0.562259	-0.961438	0	0
2D3DGALT	1	-0.1567	0.562259	-0.961438	0	0
SOR	4	-0.688752	0.75451	-1.12666	0.529421	0
SOT	1	-0.139953	0.555651	-0.946656	0	0
MAN6P	3	-0.414796	0.660854	-1.03431	0.956202	0
MAN	3	0.686049	0.246341	-0.474132	0.0287496	0
FRU	5	0.262494	0.39647	-0.719871	0.351706	0
MNT6P	1	1.94119	0.0261179	0.890234	0	0
MNT	2	-0.425429	0.664738	-1.08848	0.200568	0

MAN1P	1	0.971581	0.165629	0.0344253	0	0
GDPMAN	2	1.2885	0.0987851	-0.0197499	0.0766153	0
IDOL	2	-1.16755	0.878505	-1.55123	0.0144327	0
UDP	8	-1.89135	0.970711	-1.4119	0.950073	0
TRE6P	1	-1.38557	0.917061	-2.04609	0	0
TRE	2	2.14474	0.0159867	0.51416	1.43461	0
MLT	2	0.355549	0.361089	-0.601498	0.54624	0
MLTe	2	0.355549	0.361089	-0.601498	0.54624	0
LACT	1	0.182325	0.427664	-0.662202	0	0
LACTe	1	0.182325	0.427664	-0.662202	0	0
GLACe	2	0.422312	0.336399	-0.559867	0.144723	0
13GLUCAN	2	0.62386	0.26636	-0.434191	0.379803	0
GA6P	2	0.546498	0.292362	-0.482431	0.0352109	0
NAGA6P	1	0.952147	0.170511	0.0172715	0	0
NAGA1P	1	0.952147	0.170511	0.0172715	0	0
UDPNAG	3	-2.90485	0.998163	-2.30139	0.322201	0
CHIT	6	-2.56896	0.9949	-1.7468	1.0838	0
NAG	3	-0.72508	0.765799	-1.1922	1.38203	0
GLCN	2	0.546498	0.292362	-0.482431	0.0352109	0

13GLUCANe	5	-1.26937	0.897846	-1.32327	0.451756	0
STARe	2	1.40538	0.0799542	0.0531276	0.625022	0
GLYCOGENe	2	1.40538	0.0799542	0.0531276	0.625022	0
AMYLSe	1	0.18293	0.427427	-0.661668	0	0
AMYLPe	1	0.18293	0.427427	-0.661668	0	0
CELLUe	4	-0.829799	0.796674	-1.1888	0.4343	0
CELLOBe	4	-0.829799	0.796674	-1.1888	0.4343	0
CELLOTe	2	-0.247269	0.59765	-0.977387	0.582456	0
MANNANe	3	-0.368821	0.643869	-1.01091	0.389253	0
MANe	4	-0.111945	0.544567	-0.872567	0.421389	0
ARABINe	1	0.37572	0.353562	-0.491504	0	0
LARABe	1	0.37572	0.353562	-0.491504	0	0
H+_PO_mit	5	1.10102	0.135444	-0.389577	0.359782	0
H+_PO	5	1.10102	0.135444	-0.389577	0.359782	0
FERIm	4	1.59294	0.055587	-0.121533	0.470353	0
FEROm	4	1.59294	0.055587	-0.121533	0.470353	0
O2m	1	0.296281	0.383508	-0.56162	0	0
Ca	1	0.29738	0.383088	-0.56065	0	0
Cam	1	0.29738	0.383088	-0.56065	0	0

LLACm	3	-0.706075	0.759929	-1.18253	0.849769	0
GLUm	5	-0.235372	0.59304	-0.915979	0.783793	0
ASPm	3	-0.799329	0.78795	-1.22998	0.111866	0
ALAm	1	0.71884	0.23612	-0.188654	0	0
ASN	1	-0.0345078	0.513764	-0.853587	0	0
SAM	5	1.2867	0.0990995	-0.316438	0.602511	0
HCYS	2	0.0558524	0.47773	-0.788375	0.758192	0
SAH	4	0.580111	0.28092	-0.567703	0.812615	0
MET	2	1.13765	0.127634	-0.113817	0.195777	0
TRNAm	1	-0.534123	0.703372	-1.29457	0	0
ASPTRNAm	1	-0.534123	0.703372	-1.29457	0	0
TRNA	1	-0.748024	0.772777	-1.48336	0	0
ASPTRNA	1	-0.748024	0.772777	-1.48336	0	0
NH3	14	1.03162	0.151125	-0.580796	1.68913	1
NAGLUm	1	-0.540911	0.705716	-1.30056	0	0
NAGLUPm	1	-0.540911	0.705716	-1.30056	0	0
NAGLUSm	2	0.224483	0.411191	-0.683225	0.87304	0
NAORNm	1	0.857925	0.195467	-0.0658918	0	0
CAP	4	2.70814	0.00338307	0.369735	2.73161	1

ORN	2	4.84778	2.01403e-006	2.19965	3.20395	1
CITR	1	5.99149	3.32509e-009	4.46518	0	1
GLUGSAL	1	0.857925	0.195467	-0.0658918	0	0
DSAM	1	0.184259	0.426905	-0.660495	0	0
SPRMD	1	-0.303327	0.61918	-1.09086	0	0
GBAD	2	2.8501	0.00218527	0.953989	5.05214	1
GBAT	2	2.8501	0.00218527	0.953989	5.05214	1
DAPRP	1	-0.303327	0.61918	-1.09086	0	0
SLF	1	0.414208	0.339361	-0.457533	0	0
APS	2	0.356218	0.360839	-0.601081	0.203008	0
PAPS	2	-1.5461	0.938959	-1.78727	1.47452	0
SER	3	1.24682	0.106232	-0.188779	0.469287	0
H2S	1	-0.56897	0.715312	-1.32532	0	0
RTHIO	3	-0.868127	0.807338	-1.26499	1.3555	0
OTHIO	3	-0.868127	0.807338	-1.26499	1.3555	0
H2SO3	2	-2.01173	0.977876	-2.07762	1.06391	0
GLUGSALm	1	-1.25402	0.895083	-1.92997	0	0
P5Cm	1	-1.25402	0.895083	-1.92997	0	0
GLYm	1	-0.123366	0.549091	-0.932016	0	0

GLY	6	-0.917881	0.820659	-1.15325	0.364482	0
GLX	2	-0.343958	0.634561	-1.03768	0.355903	0
BASP	1	0.0768271	0.469381	-0.755318	0	0
THR	2	-1.16755	0.878505	-1.55123	0.0144327	0
OBUTm	1	-0.137197	0.554563	-0.944224	0	0
PRPP	4	-0.752445	0.774108	-1.15472	0.704049	0
PRBATP	1	-1.55179	0.939644	-2.1928	0	0
DIMGP	1	0.630077	0.264322	-0.266999	0	0
IMACP	1	0.630077	0.264322	-0.266999	0	0
AICAR	1	-0.756097	0.775204	-1.49049	0	0
OMVALm	1	0.782502	0.21696	-0.132463	0	0
VAL	1	-1.16121	0.877222	-1.84806	0	0
OICAP	2	1.01922	0.15405	-0.187664	0.452074	0
ABUTm	2	-0.24436	0.596524	-0.975573	0.0443345	0
ACLACm	2	-0.24436	0.596524	-0.975573	0.0443345	0
DHVALm	2	0.406555	0.342167	-0.569693	0.618336	0
DHMVAm	2	0.406555	0.342167	-0.569693	0.618336	0
OIVALm	2	0.485882	0.313525	-0.520228	0.548382	0
IPPMALm	1	-0.0961484	0.538299	-0.907993	0	0

IPPMAL	2	1.01922	0.15405	-0.187664	0.452074	0
HCITm	1	-0.375044	0.646186	-1.15416	0	0
HACNm	1	-0.375044	0.646186	-1.15416	0	0
AKA	1	-0.288227	0.613414	-1.07753	0	0
AMA	2	-0.0656349	0.526166	-0.864129	0.301794	0
AMASA	1	-0.0208988	0.508337	-0.841575	0	0
SACP	1	-0.0208988	0.508337	-0.841575	0	0
LYS	1	-0.981451	0.836815	-1.68939	0	0
LTRNA	1	-0.981451	0.836815	-1.68939	0	0
LLTRNA	1	-0.981451	0.836815	-1.68939	0	0
LYSm	1	-0.981451	0.836815	-1.68939	0	0
LTRNAm	1	-0.981451	0.836815	-1.68939	0	0
LLTRNAm	1	-0.981451	0.836815	-1.68939	0	0
ADN	3	-0.0640746	0.525545	-0.855839	0.408087	0
MTHPTGLU	1	0.646785	0.258886	-0.252252	0	0
THPTGLU	1	0.646785	0.258886	-0.252252	0	0
3DDAH7P	2	-0.956774	0.830659	-1.4198	0.235585	0
DQT	2	-0.15652	0.562188	-0.9208	0.47011	0
DHSK	1	-0.487278	0.686969	-1.25322	0	0

QT	1	0.265961	0.395135	-0.588382	0	0
SME	1	-0.487278	0.686969	-1.25322	0	0
SME5P	1	-0.487278	0.686969	-1.25322	0	0
3PSME	1	-0.487278	0.686969	-1.25322	0	0
4HPP	2	-0.600567	0.725936	-1.19769	0.137007	0
TYR	2	-0.600567	0.725936	-1.19769	0.137007	0
AN	1	0.782502	0.21696	-0.132463	0	0
NPRAN	1	0.513869	0.303672	-0.369569	0	0
CPAD5P	1	0.513869	0.303672	-0.369569	0	0
IGP	1	0.513869	0.303672	-0.369569	0	0
FKYN	1	-0.840076	0.799567	-1.56461	0	0
KYN	3	0.0541173	0.478421	-0.795696	0.7219	0
HKYN	2	0.660671	0.254412	-0.411238	0.394247	0
HAN	1	0.782502	0.21696	-0.132463	0	0
PAD	3	1.94645	0.0258003	0.167236	3.82348	1
PAC	3	1.94645	0.0258003	0.167236	3.82348	1
IAD	3	1.94645	0.0258003	0.167236	3.82348	1
IAC	3	1.94645	0.0258003	0.167236	3.82348	1
P5C	1	0.782502	0.21696	-0.132463	0	0

PRO	1	0.782502	0.21696	-0.132463	0	0
PHC	1	0.782502	0.21696	-0.132463	0	0
HPRO	1	0.782502	0.21696	-0.132463	0	0
GABALm	2	-1.33438	0.90896	-1.65526	0.321518	0
GABAm	2	-1.33438	0.90896	-1.65526	0.321518	0
LACALm	2	-1.33438	0.90896	-1.65526	0.321518	0
PEPD	1	0.145084	0.442322	-0.695072	0	0
APEP	1	0.145084	0.442322	-0.695072	0	0
GC	1	-0.103335	0.541152	-0.914337	0	0
OGT	2	-0.394307	0.653323	-1.06907	0.115011	0
cAMP	3	0.767106	0.221509	-0.432885	0.241678	0
GMP	3	-0.0122897	0.504903	-0.829488	0.804677	0
DGDP	1	-0.392124	0.652517	-1.16923	0	0
DATP	2	-0.0241823	0.509646	-0.838281	0.468036	0
DADP	3	-1.05563	0.854432	-1.3604	0.737384	0
SAICAR	1	-0.756097	0.775204	-1.49049	0	0
IMP	4	0.201621	0.420106	-0.734435	0.464171	0
ASUC	1	-0.756097	0.775204	-1.49049	0	0
XMP	2	-0.79848	0.787704	-1.3211	0.577726	0

cdAMP	1	0.72884	0.23305	-0.179827	0	0
DAMP	3	-0.407516	0.658185	-1.0306	1.02917	0
cIMP	1	0.72884	0.23305	-0.179827	0	0
cGMP	1	0.72884	0.23305	-0.179827	0	0
cCMP	1	0.72884	0.23305	-0.179827	0	0
UGC	1	0.0420473	0.483231	-0.786016	0	0
CAASP	2	-0.276179	0.608795	-0.995414	0.146701	0
OROA	1	0.0805545	0.467898	-0.752028	0	0
OMP	2	0.714724	0.23739	-0.377533	0.529616	0
UMP	2	1.07131	0.142015	-0.155182	0.215163	0
URA	2	-0.717907	0.763593	-1.27086	0.858231	0
CYTS	2	-0.874254	0.80901	-1.36835	0.720358	0
URI	1	0.199885	0.420785	-0.646703	0	0
CYTD	1	0.199885	0.420785	-0.646703	0	0
DU	2	0.269188	0.393893	-0.655349	0.0122267	0
DR1P	1	0.180294	0.428461	-0.663994	0	0
DT	1	0.180294	0.428461	-0.663994	0	0
THY	1	0.180294	0.428461	-0.663994	0	0
DC	1	0.199885	0.420785	-0.646703	0	0

DTDP	1	-0.392124	0.652517	-1.16923	0	0
OTHIOm	1	0.414	0.339437	-0.457717	0	0
RTHIOm	1	0.414	0.339437	-0.457717	0	0
DUTP	3	-0.348279	0.636185	-1.00046	0.434091	0
DUMP	2	0.0114359	0.495438	-0.816071	0.719475	0
DCMP	1	0.584389	0.279479	-0.307325	0	0
DCDP	2	0.136192	0.445835	-0.738279	0.60946	0
CDP	2	0.136192	0.445835	-0.738279	0.60946	0
AD	3	-0.0962304	0.538331	-0.872202	0.341485	0
INS	2	0.323455	0.373175	-0.62151	0.0600819	0
DA	1	0.180294	0.428461	-0.663994	0	0
DIN	1	0.180294	0.428461	-0.663994	0	0
HYXN	1	0.180294	0.428461	-0.663994	0	0
DG	2	0.648257	0.258409	-0.418979	0.346504	0
GN	1	0.180294	0.428461	-0.663994	0	0
GSN	3	0.689593	0.245225	-0.472328	0.26186	0
XAN	1	0.180294	0.428461	-0.663994	0	0
XTSINE	1	0.180294	0.428461	-0.663994	0	0
ITP	2	-0.208691	0.582655	-0.953332	0.305329	0

IDP	2	-0.208691	0.582655	-0.953332	0.305329	0
ITPm	1	-1.53111	0.937129	-2.17455	0	0
IDPm	1	-1.53111	0.937129	-2.17455	0	0
DGTP	3	0.405598	0.342519	-0.616842	0.506591	0
DUDP	2	0.136192	0.445835	-0.738279	0.60946	0
DCTP	2	-0.0241823	0.509646	-0.838281	0.468036	0
DTTP	1	-0.392124	0.652517	-1.16923	0	0
LCCA	3	-0.958811	0.831173	-1.31113	0.7045	0
ACOA	5	-0.74686	0.772426	-1.11745	0.635568	0
HACOA	2	0.488433	0.312621	-0.518637	0.481311	0
OACOA	4	0.341572	0.366336	-0.672784	0.472055	0
AACCOA	1	0.464065	0.321301	-0.413528	0	0
AACCOAm	1	0.464065	0.321301	-0.413528	0	0
MALCOA	2	0.660757	0.254384	-0.411185	0.667428	0
MALACP	1	-0.0679764	0.527098	-0.883127	0	0
ACACP	1	0.954217	0.169987	0.0190991	0	0
C120ACP	4	0.485739	0.313576	-0.609276	0.572814	0
C140ACP	4	0.485739	0.313576	-0.609276	0.572814	0
C141ACP	4	0.485739	0.313576	-0.609276	0.572814	0

C160ACP	4	0.485739	0.313576	-0.609276	0.572814	0
C161ACP	4	0.485739	0.313576	-0.609276	0.572814	0
C180ACP	4	0.485739	0.313576	-0.609276	0.572814	0
C181ACP	4	0.485739	0.313576	-0.609276	0.572814	0
C182ACP	4	0.485739	0.313576	-0.609276	0.572814	0
3HPACP	1	0.954217	0.169987	0.0190991	0	0
2HDACP	1	0.954217	0.169987	0.0190991	0	0
AACP	1	0.954217	0.169987	0.0190991	0	0
23DAACP	1	0.954217	0.169987	0.0190991	0	0
C150ACP	2	-0.364374	0.642211	-1.05041	0.16269	0
C162ACP	4	0.485739	0.313576	-0.609276	0.572814	0
C170ACP	4	0.485739	0.313576	-0.609276	0.572814	0
C183ACP	4	0.485739	0.313576	-0.609276	0.572814	0
C200ACP	4	0.485739	0.313576	-0.609276	0.572814	0
AGL3P	2	1.05052	0.146738	-0.168143	0.423659	0
AT3P2	1	1.08148	0.139741	0.131429	0	0
PA	4	-0.20126	0.579752	-0.911912	0.866395	0
PAm	1	0.646785	0.258886	-0.252252	0	0
CTPm	2	0.180352	0.428438	-0.710742	0.648403	0

CDPDGm	3	0.164843	0.434534	-0.739352	0.455278	0
CDPDG	2	1.15743	0.123548	-0.101482	0.213221	0
CMPm	3	0.128767	0.448771	-0.75771	0.425985	0
PE	2	0.318721	0.374969	-0.624462	0.221673	0
PMME	2	1.16306	0.122402	-0.0979705	0.966244	0
PDME	1	1.59567	0.0552815	0.585267	0	0
PC	2	1.41444	0.0786167	0.0587758	0.744571	0
PCHO	1	-1.16121	0.877222	-1.84806	0	0
CDPCHO	2	-0.536738	0.704276	-1.15789	0.976049	0
DAGLY	6	-0.653766	0.743369	-1.0583	0.592693	0
PETHM	1	-0.197356	0.578225	-0.997322	0	0
CDPETN	1	0.402672	0.343595	-0.467716	0	0
MI1P	2	-0.587718	0.721639	-1.18968	1.71327	0
MYOI	2	-0.565671	0.714191	-1.17593	1.73272	0
PINS	5	0.386291	0.349641	-0.671107	0.496976	0
PINSP	3	-0.352602	0.637807	-1.00266	0.178847	0
PINS4P	2	0.594898	0.275956	-0.452251	0.604995	0
D45PI	2	0.616116	0.268909	-0.439021	0.586285	0
TPI	1	-0.0345078	0.513764	-0.853587	0	0

GL3Pm	1	-0.375044	0.646186	-1.15416	0	0
PGPm	2	-0.743995	0.77156	-1.28712	0.188042	0
PGm	2	-0.469352	0.680591	-1.11587	0.430233	0
CLm	1	0.013008	0.494811	-0.811647	0	0
DGPP	2	-1.0274	0.847884	-1.46384	1.00509	0
CDPm	2	0.136192	0.445835	-0.738279	0.60946	0
PALCOA	2	1.41239	0.0789179	0.0574976	0.276673	0
DHSPH	3	0.712181	0.238176	-0.460834	0.918845	0
SPH	3	0.459712	0.322862	-0.589305	0.795178	0
PSPH	2	1.10334	0.13494	-0.135209	0.165524	0
CER2	1	-0.0694989	0.527704	-0.884471	0	0
CER3	2	0.292745	0.384858	-0.640659	0.344802	0
IPC	2	0.942686	0.172921	-0.235386	0.22834	0
MIPC	2	0.942686	0.172921	-0.235386	0.22834	0
MIP2C	1	0.482963	0.314561	-0.396847	0	0
DHSP	2	0.505903	0.306463	-0.507744	0.692368	0
PHSP	1	0.911997	0.180885	-0.0181665	0	0
C16A	1	-0.197356	0.578225	-0.997322	0	0
H3MCOA	1	0.267277	0.394628	-0.58722	0	0

MVL	1	0.267277	0.394628	-0.58722	0	0
IPPP	1	-0.983323	0.837276	-1.69105	0	0
DMPP	1	-0.983323	0.837276	-1.69105	0	0
GPP	1	-0.983323	0.837276	-1.69105	0	0
FPP	1	-0.983323	0.837276	-1.69105	0	0
S23E	1	-0.0208988	0.508337	-0.841575	0	0
LNST	1	-0.0208988	0.508337	-0.841575	0	0
IGST	1	0.839974	0.200462	-0.0817367	0	0
DMZYMST	3	-0.329769	0.629213	-0.99104	0.789182	0
IMZYMST	3	-0.0769422	0.530665	-0.862387	1.01164	0
IIMZYMST	2	1.79585	0.0362595	0.296605	0.0107743	0
MZYMST	3	-0.0869231	0.534634	-0.867466	1.00286	0
IZYMST	3	-0.0769422	0.530665	-0.862387	1.01164	0
IIZYMST	2	1.79585	0.0362595	0.296605	0.0107743	0
ZYMST	2	0.950212	0.171002	-0.230694	0.734938	0
FEST	2	0.0436654	0.482586	-0.795974	0.0644892	0
EPST	2	-0.465462	0.6792	-1.11344	0.384478	0
ERTROL	2	0.89188	0.186229	-0.267067	1.58143	0
ERTEOL	1	1.89693	0.0289184	0.851175	0	0

TAGLY	2	-0.364374	0.642211	-1.05041	0.16269	0
MAGLY	1	-0.387836	0.650931	-1.16545	0	0
PHACAL	2	-1.33438	0.90896	-1.65526	0.321518	0
PHAC	2	-1.33438	0.90896	-1.65526	0.321518	0
PHACCOA	1	0.195324	0.42257	-0.650728	0	0
IPN	1	0.195324	0.42257	-0.650728	0	0
PENG	1	0.195324	0.42257	-0.650728	0	0
NOR	1	1.08213	0.139597	0.132	0	0
AVN	1	1.08213	0.139597	0.132	0	0
VERB	2	-2.11355	0.982723	-2.14111	0.414445	0
VERA	1	-1.82526	0.966019	-2.43417	0	0
DMST	1	-1.82526	0.966019	-2.43417	0	0
DHDMST	1	-1.82526	0.966019	-2.43417	0	0
OMST	2	0.257817	0.398274	-0.662439	1.82859	0
DHOMST	2	0.257817	0.398274	-0.662439	1.82859	0
AFB1	2	0.257817	0.398274	-0.662439	1.82859	0
AFG1	2	0.257817	0.398274	-0.662439	1.82859	0
AFB2	2	0.257817	0.398274	-0.662439	1.82859	0
AFG2	2	0.257817	0.398274	-0.662439	1.82859	0

HNO2	1	-0.522405	0.699306	-1.28422	0	0
NH4OH	1	-0.522405	0.699306	-1.28422	0	0
NH3e	2	0.725375	0.234111	-0.370892	1.01183	0
FRUe	1	0.414208	0.339361	-0.457533	0	0
SORe	1	0.414208	0.339361	-0.457533	0	0
