# Supplementary material for: Systems Analysis Unfolds the Relationship between the Phosphoketolase Pathway and Growth in Aspergillus nidulans
Source: PLoS One. 2008 Dec 4;3(12):e3847. doi: 10.1371/journal.pone.0003847 (PMC2585806; doi:10.1371/journal.pone.0003847)
Supplement: Table S8 — General functional (KOG/Interpro/PFAM) assignment and SwissProt gene names for 2805 genes from A. nidulans. (0.38 MB PDF) [file pone.0003847.s008.pdf]

Gene	Annotation
AN0006.3	
AN0013.3	
AN0034.3	Dak kinase
AN0038.3	Atm
AN0044.3	Rad5
AN0048.3	
AN0050.3	Amidohydrolase 2
AN0051.3	Iron/ascorbate family oxidoreductases
AN0052.3	short chain dehydrogenase
AN0064.3	
AN0069.3	WD40 repeat-containing protein
AN0085.3	
AN0087.3	Zn-finger-like, PHD finger
AN0091.3	Dot1
AN0092.3	Spb1
AN0095.3	Irs4
AN0096.3	Fungal Zn(2)-Cys(6) binuclear cluster domain
AN0097.3	Dnl4

AN0098.3	NirA
AN0103.3	Protein involved in cell cycle control in A. niger
AN0111.3	Syf1
AN0113.3	RhoGEF domain
AN0117.3	Svf1
AN0118.3	DyhC
AN0123.3	Ribosomal protein L23
AN0127.3	Atg18
AN0128.3	Lcmt2
AN0129.3	Dual specificity phosphatase, catalytic domain
AN0132.3	
AN0135.3	Hypothetical myosin assembly protein/sexual cycle protein in A. niger
AN0150.3	Acyl transferase domain
AN0158.3	Alcohol dehydrogenase
AN0166.3	Nuf2
AN0173.3	
AN0178.3	
AN0182.3	Ras
AN0185.3	

AN0204.3	Dbp7
AN0211.3	Ru2A
AN0214.3	Ank repeat
AN0221.3	Glycosyl hydrolases family 18
AN0224.3	Peptidase M19
AN0226.3	Ubiquitin-conjugating enzyme
AN0227.3	
AN0230.3	
AN0231.3	Tyrosinase
AN0232.3	HypB/UreG, nucleotide-binding
AN0234.3	
AN0235.3	Eukaryotic protein kinase domain
AN0248.3	Thioredoxin
AN0256.3	PalB
AN0261.3	Sec23
AN0267.3	Myosin regulatory light chain
AN0271.3	dUTPase
AN0279.3	Transcription factor, Myb superfamily
AN0280.3	Glycoside hydrolase, family 31

AN0282.3	RNA recognition motif. (a.k.a. RRM, RBD, or RNP domain)
AN0287.3	WD domain, G-beta repeat
AN0289.3	Cwc22
AN0290.3	CapzB
AN0293.3	Ppme1
AN0297.3	
AN0298.3	Yth1
AN0301.3	
AN0303.3	
AN0307.3	
AN0309.3	
AN0313.3	Arylacetamide deacetylase
AN0316.3	Tba1
AN0319.3	WD40 repeat protein
AN0323.3	
AN0327.3	Ruvb2
AN0331.3	
AN0335.3	
AN0338.3	Cytochrome P450

AN0340.3	Serine/threonine protein kinase
AN0348.3	
AN0355.3	
AN0356.3	
AN0362.3	
AN0364.3	
AN0365.3	
AN0366.3	
AN0370.3	Enoyl-CoA hydratase/isomerase family
AN0374.3	
AN0386.3	
AN0393.3	Glycosyl hydrolase family 76
AN0401.3	
AN0402.3	
AN0406.3	Vts1
AN0407.3	TPR Domain
AN0408.3	Plpl
AN0410.3	Pp1
AN0411.3	Sar1

AN0421.3	Mrd1
AN0422.3	AbaA
AN0430.3	
AN0432.3	Oxidoreductase FAD-binding domain
AN0436.3	
AN0438.3	
AN0442.3	
AN0443.3	Zinc-containing alcohol dehydrogenase
AN0457.3	
AN0458.3	Molecular chaperone (DnaJ superfamily)
AN0462.3	
AN0467.3	
AN0468.3	
AN0471.3	Sodium/calcium exchanger protein
AN0474.3	
AN0482.3	
AN0483.3	
AN0484.3	
AN0485.3	Peptidase

AN0490.3	PyrG
AN0494.3	glycoside hydrolase, family 7
AN0499.3	Chitin binding Peritrophin-A domain
AN0500.3	
AN0501.3	Sugar (ANd other) transporter
AN0506.3	
AN0528.3	
AN0536.3	
AN0539.3	
AN0540.3	
AN0546.3	
AN0548.3	
AN0551.3	Glycosyl hydrolase family 47
AN0553.3	
AN0554.3	AldH
AN0560.3	Exo84
AN0565.3	Pyr1
AN0576.3	Eukaryotic protein kinase domain
AN0578.3	

AN0580.3	
AN0582.3	Single-stranded DNA-binding replication protein A (RPA), medium (30 kD) subunit
AN0583.3	Dbp10
AN0585.3	hypothetical protein with predicted fungal transcriptional regulatory domain
AN0587.3	Molecular chaperones HSP70/HSC70
AN0589.3	Dbp4
AN0591.3	Phosphoadenosine phosphosulfate reductase family
AN0593.3	Predicted dehydrogenase
AN0597.3	
AN0600.3	
AN0602.3	
AN0606.3	Cytochrome P450
AN0607.3	hypothetical non-ribosomal peptide synthetase
AN0608.3	
AN0610.3	Aldo/keto reductase family
AN0617.3	Dihydrodipicolinate synthetase family
AN0623.3	Hypothetical long chain fatty alcohol oxidase
AN0626.3	GCN5-related N-acetyltransferase
AN0628.3	D-isomer specific 2-hydroxyacid dehydrogenase, NAD binding domain

AN0635.3	
AN0638.3	
AN0640.3	Sterol desaturase
AN0648.3	TrpG
AN0649.3	Acyl-CoA synthetase
AN0651.3	Gpa1
AN0656.3	
AN0660.3	Permeases for cytosine/purines, uracil, thiamine, allantoin
AN0663.3	
AN0667.3	Mpi
AN0670.3	GTP cyclohydrolase II
AN0674.3	Protein of unknown function DUF6
AN0676.3	Tbg
AN0680.3	
AN0681.3	Cys/Met metabolism pyridoxal-phosphate-dependent enzymes
AN0685.3	
AN0691.3	
AN0696.3	
AN0707.3	Xrn2

AN0708.3	Aro1
AN0717.3	Aminotransferase class I and II
AN0720.3	Dph5
AN0732.3	General substrate transporter
AN0736.3	
AN0741.3	PelA
AN0746.3	May be involved in regulation of ribosomal RNA transcription
AN0747.3	Insulinase-like
AN0756.3	Glycosyl hydrolases family 35
AN0759.3	
AN0761.3	O-methyltransferase, family 2
AN0768.3	Cell adhesin
AN0771.3	
AN0773.3	Ferric reductase-like transmembrane component
AN0779.3	
AN0787.3	Glycosyl hydrolase family 47
AN0791.3	
AN0792.3	
AN0795.3	

AN0798.3	
AN0800.3	
AN0801.3	AMP-binding enzyme
AN0802.3	
AN0806.3	WD domain, G-beta repeat
AN0815.3	
AN0817.3	Putative transcriptional repressor
AN0820.3	
AN0821.3	
AN0827.3	Gwt1
AN0833.3	
AN0841.3	
AN0845.3	
AN0857.3	
AN0858.3	ATPases associated with various cellular activities (AAA)
AN0867.3	
AN0868.3	Acyl-CoA thioesterase
AN0869.3	
AN0872.3	Cell cycle regulation

AN0878.3	Multicopper oxidase
AN0881.3	
AN0884.3	
AN0885.3	Transcription factor containing homeobox and Zn-finger
AN0888.3	
AN0889.3	GTP1/OBG subdomain
AN0890.3	Permease of the major facilitator superfamily
AN0891.3	Uay
AN0893.3	Adenylosuccinate synthetase
AN0896.3	
AN0901.3	Laccase
AN0902.3	Fungal transcriptional regulatory protein
AN0904.3	
AN0918.3	Sphingolipid fatty acid hydroxylase
AN0920.3	
AN0925.3	SacI homology domain
AN0928.3	Fungal transcriptional regulatory protein, N-terminal
AN0935.3	
AN0936.3	Threonyl-tRNA synthetase

AN0938.3	Sugar (ANd other) transporter
AN0941.3	Glycosyl hydrolases family 31
AN0942.3	
AN0943.3	
AN0944.3	Rok1
AN0948.3	ABC transporter
AN0950.3	
AN0959.3	
AN0963.3	
AN0964.3	
AN0966.3	Swc5
AN0969.3	N-acetyltransferase activity
AN0973.3	BrlA
AN0974.3	
AN0980.3	Glycosyl hydrolases family 35
AN0981.3	
AN0986.3	Predicted transcription factor
AN0990.3	Ysh1
AN0995.3	Stu1

AN10003.3	
AN10005.3	Short-chain dehydrogenase
AN10018.3	
AN10019.3	
AN1003.3	Isocitrate/isopropylmalate dehydrogenase
AN10034.3	Uncharacterized conserved protein
AN10040.3	
AN10045.3	
AN10057.3	
AN1006.3	NiA
AN10060.3	
AN10062.3	
AN10063.3	Helicase, C-terminal
AN10066.3	
AN10067.3	Pam17
AN1007.3	Nir
AN10075.3	Major facilitator superfamily
AN10078.3	ABC transporter
AN1008.3	Crna

AN10080.3	
AN10081.3	
AN10087.3	
AN1009.3	
AN10090.3	
AN10095.3	
AN10096.3	
AN10098.3	Zinc-binding oxidoreductase
AN10099.3	
AN10108.3	Y0679
AN10109.3	
AN10111.3	PtpA2
AN10113.3	
AN10117.3	
AN10119.3	Pan3
AN10123.3	
AN10124.3	Glycoside hydrolase, family 1
AN10126.3	Ketopantoate reductase ApbA/PanE
AN10129.3	

AN10136.3	Cation transporter
AN10140.3	Transcription coactivator
AN10148.3	
AN1015.3	Carbohydrate phosphorylases
AN10150.3	
AN10158.3	
AN10160.3	Uncharacterized conserved protein
AN10161.3	Ribosomal protein S6
AN10167.3	Flavin-containing monooxygenase
AN1017.3	Hog1
AN10171.3	Rmd11
AN10175.3	
AN10178.3	
AN10181.3	
AN10186.3	Guanylate kinase
AN10195.3	SyV
AN10197.3	
AN10201.3	RNA-binding protein musashi/mRNA cleavage and polyadenylation factor I complex
AN10208.3	Csn8

AN1021.3	
AN10213.3	Might be involved in signal transduction
AN10220.3	CcpR
AN10221.3	
AN10234.3	GCN5-related N-acetyltransferase
AN10235.3	RNA polymerase II, large subunit
AN10237.3	
AN10238.3	
AN10242.3	
AN10252.3	
AN10254.3	Y1985
AN10259.3	Cytochrome P450
AN10268.3	
AN10269.3	
AN10271.3	hypothetical gamma interferon inducible lysosomal thiol reductase
AN10282.3	
AN10283.3	Tah18
AN10293.3	
AN10296.3	

AN10297.3	NRPS
AN10298.3	Phosphoserine aminotransferase
AN10299.3	Pyridoxal-dependent decarboxylase
AN10303.3	
AN10305.3	
AN10311.3	
AN10314.3	
AN10315.3	LepA
AN10317.3	TBP-associated transcription factor Prodos
AN10318.3	
AN10321.3	Major facilitator superfamily
AN10325.3	
AN10326.3	
AN10327.3	
AN10332.3	
AN10338.3	
AN10351.3	Peptidase M18, aminopeptidase I
AN10354.3	Peptidase S26B, eukaryotic signal peptidase
AN10356.3	

AN10358.3	Zinc-containing alcohol dehydrogenase
AN10360.3	
AN10363.3	
AN10370.3	
AN10375.3	
AN10376.3	
AN10378.3	KapC
AN1038.3	
AN10380.3	Dcl2
AN10385.3	
AN10390.3	
AN10392.3	Histidine acid phosphatase
AN10393.3	SyI
AN10395.3	
AN10399.3	
AN10403.3	
AN10407.3	
AN10410.3	Major facilitator superfamily
AN10422.3	

AN10424.3	
AN10426.3	
AN10432.3	Fungal specific transcription factor
AN10438.3	
AN10448.3	
AN10455.3	Uroporphyrinogen III synthase HEM4
AN10467.3	
AN10470.3	DNA polymerase delta, regulatory subunit 55
AN10473.3	
AN10482.3	Glycoside hydrolase, family 3
AN10485.3	Cbk1
AN10488.3	
AN10489.3	Fkbp4
AN10494.3	
AN10496.3	
AN10498.3	
AN10504.3	Fungal specific transcription factor
AN10506.3	
AN10507.3	

AN10516.3	GrrA
AN10518.3	
AN1052.3	
AN10520.3	Predicted hydrolase
AN10522.3	
AN10540.3	Peptidase M49
AN10549.3	
AN10552.3	Acyl-CoA thioesterase
AN10557.3	Ded1
AN10577.3	Dihydrolipoamide dehydrogenase
AN10578.3	
AN10581.3	
AN10582.3	FAD dependent oxidoreductase
AN10589.3	
AN10599.3	Mandelate racemase
AN10600.3	Fungal specific transcription factor
AN10601.3	Glycosyl transferase, family 25
AN10602.3	Aldehyde dehydrogenase
AN10604.3	

AN10607.3	Splicing coactivator SRm160/300
AN10609.3	FOG: RCC1 domain
AN1061.3	Amino acid permease
AN10611.3	
AN10625.3	
AN10626.3	
AN10629.3	
AN10631.3	
AN10646.3	Fructosamine kinase
AN1065.3	Iws1
AN10650.3	
AN10655.3	
AN10659.3	Fungal specific transcription factor
AN1066.3	
AN10661.3	5' nucleotidase
AN10663.3	
AN1067.3	
AN10671.3	
AN10672.3	GH family 92

AN10683.3	purine nucleoside permease
AN10689.3	Serine/threonine dehydratase
AN1069.3	Chs7
AN10691.3	Dynamin
AN10695.3	Glutathione S-transferase
AN10698.3	
AN10707.3	SNF2-related
AN10715.3	
AN10718.3	
AN10725.3	
AN10726.3	
AN10729.3	
AN10732.3	Phox-like
AN10736.3	
AN10738.3	
AN10741.3	
AN10752.3	
AN1076.3	Cation efflux protein
AN10761.3	

AN10763.3	Histone acetyltransferase complex
AN10764.3	Enoyl-CoA hydratase/isomerase
AN10767.3	Permease for cytosine/purines, uracil, thiamine, allantoin
AN10773.3	
AN10787.3	
AN10788.3	Bro1
AN10789.3	
AN10790.3	
AN10797.3	
AN10804.3	
AN10811.3	Cytochrome P450
AN10812.3	
AN10815.3	
AN10825.3	
AN10830.3	
AN10834.3	
AN10837.3	Glutamine phosphoribosylpyrophosphate amidotransferase
AN1084.3	EftU
AN10842.3	

AN10843.3	
AN10845.3	Sugar transporter
AN10847.3	
AN1085.3	Sey1
AN10860.3	
AN10864.3	
AN10867.3	
AN10869.3	
AN1087.3	Cytochrome P450
AN10872.3	Cytochrome c heme-binding site
AN10873.3	Predicted cell surface protein homologous to bacterial outer membrane proteins
AN10876.3	Cation efflux protein
AN1088.3	
AN10885.3	
AN10886.3	
AN1089.3	
AN10891.3	
AN10898.3	
AN10905.3	Amino acid/polyamine transporter I

AN10907.3	
AN10908.3	Esterase/lipase/thioesterase
AN10910.3	
AN10911.3	
AN10915.3	Predicted histone tail methylase
AN10921.3	WD40 repeat-containing protein
AN10921.3	WD4 repeat-containing protein
AN10923.3	
AN10930.3	hypothetical extracellular FAD/FMN-containing dehydrogenase
AN10934.3	Pfa3
AN10935.3	
AN10937.3	Serine/threonine protein kinase
AN1094.3	FAD-dependent pyridine nucleotide-disulphide oxidoreductase
AN10944.3	Cef1
AN10949.3	ABC transporter
AN10954.3	WD40 repeat stress protein/actin interacting protein
AN10959.3	Nucleolar GTPase/ATPase p130
AN10964.3	Cytochrome P450
AN10967.3	Predicted Yippee-type zinc-binding protein

AN10973.3	Citrate synthase
AN10977.3	
AN10978.3	
AN10981.3	GTP cyclohydrolase II
AN10983.3	
AN10990.3	
AN10991.3	
AN10993.3	
AN11002.3	
AN11004.3	Atg3
AN11006.3	
AN11008.3	
AN1101.3	Amino acid/polyamine transporter II
AN11013.3	Stcl
AN11022.3	
AN11025.3	
AN11038.3	N-Acetylglucosamine kinase
AN11043.3	
AN11046.3	

AN11053.3	
AN11059.3	
AN11062.3	
AN11064.3	
AN11068.3	
AN11070.3	Peptidase M18, aminopeptidase I
AN11076.3	
AN11079.3	
AN11080.3	
AN11081.3	
AN11083.3	
AN11085.3	Glucose/ribitol dehydrogenase
AN11093.3	Fungal specific transcription factor
AN11094.3	Zinc-binding oxidoreductase
AN11095.3	
AN11099.3	
AN1110.3	
AN11101.3	Protein kinase activity
AN11110.3	Alanyl-tRNA synthetase

AN11116.3	Major facilitator superfamily
AN11117.3	Phospholipase/carboxyhydrolase
AN11120.3	Major facilitator superfamily
AN11121.3	
AN11140.3	
AN11142.3	Cytochrome P450
AN11143.3	
AN11146.3	Cytidine deaminase, homotetrameric
AN11149.3	
AN1115.3	Predicted inosine-uridine preferring nucleoside hydrolase
AN11151.3	
AN11153.3	Major facilitator superfamily
AN11154.3	Aminoacyl-tRNA synthetase, class I
AN11156.3	
AN11167.3	ABC transporter
AN1117.3	
AN11173.3	
AN11177.3	
AN11185.3	

AN11200.3	
AN11207.3	
AN11209.3	
AN11212.3	
AN11217.3	
AN11219.3	Major facilitator superfamily
AN11221.3	Proline dehydrogenase
AN11232.3	Glycoside hydrolase, family 18
AN11236.3	Cox3
AN11239.3	
AN1124.3	
AN11242.3	
AN11252.3	3-hydroxyanthranilate oxygenase HAAO
AN11271.3	
AN11274.3	
AN11280.3	
AN11281.3	
AN11286.3	
AN1129.3	Flavin-containing monooxygenase

AN1130.3	
AN11303.3	
AN1131.3	Hypothetical superoxide dismutase
AN11323.3	
AN1133.3	N-methyl-D-aspartate receptor glutamate-binding subunit
AN11334.3	
AN11337.3	
AN1134.3	QutA
AN1136.3	QutG
AN1137.3	DhqA
AN11374.3	
AN11378.3	
AN1138.3	QutD
AN11381.3	
AN11387.3	
AN11395.3	
AN1140.3	
AN11401.3	
AN11403.3	

AN11415.3

AN11432.3

AN11433.3

AN11436.3

AN11438.3

AN11440.3

AN11468.3

AN11477.3

AN11489.3

AN11498.3

AN1151.3

AN11510.3

AN11526.3

AN11531.3

AN11533.3

AN11541.3

AN11552.3

AN11563.3

AN11567.3

AN11576.3	
AN11581.3	
AN11583.3	
AN11584.3	
AN11597.3	
AN11601.3	
AN11608.3	
AN11620.3	
AN11624.3	
AN11627.3	
AN1163.3	Clpb1
AN11638.3	
AN11645.3	
AN11646.3	
AN1165.3	Tsc10
AN11657.3	
AN11672.3	
AN1169.3	
AN1181.3	Ammonium transporter

AN1182.3	Tbb1
AN1184.3	Amidohydrolase
AN1186.3	Permease for cytosine/purines, uracil, thiamine, allantoin
AN1187.3	
AN1189.3	Cation transporting ATPase, C-terminus
AN1194.3	KapS
AN1198.3	Glycine cleavage system T protein
AN1199.3	Transmembrane amino acid transporter protein
AN1201.3	Prolyl-tRNA synthetase
AN1202.3	
AN1208.3	Prp46
AN1212.3	Fungal transcriptional regulatory protein, N-terminal
AN1217.3	Homeobox transcription factor
AN1222.3	MetK
AN1227.3	Med16
AN1240.3	
AN1242.3	Non-ribosomal peptide synthetase
AN1243.3	Major facilitator superfamily
AN1244.3	

AN1246.3	Pgk
AN1259.3	Clf1
AN1262.3	
AN1266.3	Prp5
AN1273.3	Glycoside hydrolase, family 6
AN1277.3	
AN1286.3	Hir1
AN1290.3	Predicted K+/H+-antiporter
AN1291.3	Glutathione-dependent formaldehyde-activating, GFA
AN1298.3	Helix loop helix transcription factor EB
AN1302.3	
AN1303.3	Fungal specific transcription factor domain
AN1304.3	
AN1305.3	
AN1306.3	Actin regulatory proteins
AN1311.3	Monooxygenase
AN1312.3	
AN1322.3	
AN1327.3	

AN1338.3	
AN1356.3	von Willebrand factor
AN1360.3	Acetyltransferase (GNAT) family
AN1370.3	Cell cycle-associated protein Mob1-1
AN1377.3	Regulator of G protein
AN1378.3	
AN1383.3	Signal transduction
AN1389.3	
AN1392.3	Spc25
AN1399.3	Sgo1
AN1402.3	Serine O-acetyltransferase
AN1413.3	Cft1
AN1414.3	PhoG
AN1424.3	
AN1426.3	Serine carboxypeptidase
AN1432.3	Predicted RNA binding protein
AN1433.3	Carboxylesterases
AN1438.3	
AN1443.3	RNA polymerase II, large subunit

AN1444.3	NADH-ubiquinone oxidoreductase
AN1452.3	Nop12
AN1461.3	Os9
AN1463.3	
AN1465.3	
AN1467.3	Serine/threonine protein phosphatase
AN1472.3	C4-dicarboxylate transporter/malic acid transport protein
AN1477.3	
AN1479.3	DNA mismatch repair protein MutS
AN1482.3	Acetoacetyl-CoA synthase
AN1489.3	
AN1491.3	Csn1
AN1497.3	
AN1502.3	Glycosyl hydrolase family 20
AN1503.3	Dihydrodipicolinate synthetase family
AN1506.3	Major facilitator superfamily
AN1509.3	
AN1510.3	Endoplasmic Reticulum Oxidoreductin 1 (ERO1)
AN1519.3	Translation initiation factor 2C

AN1522.3	Predicted membrane protein
AN1523.3	AtpA
AN1527.3	Yfas1
AN1532.3	
AN1539.3	Csn4
AN1540.3	
AN1541.3	Aldehyde dehydrogenase
AN1542.3	
AN1543.3	Fumarate reductase, flavoprotein subunit
AN1547.3	Acetyl-CoA hydrolase
AN1549.3	
AN1555.3	ChsD
AN1561.3	
AN1565.3	Pfa4
AN1566.3	
AN1567.3	
AN1568.3	
AN1573.3	Eukaryotic aspartyl protease
AN1577.3	Sugar (ANd other) transporter

AN1578.3	
AN1580.3	Kinesin light chain
AN1583.3	Peptidase family M20/M25/M40
AN1584.3	
AN1586.3	Copper amine oxidase
AN1587.3	
AN1588.3	
AN1589.3	
AN1590.3	
AN1591.3	ATPases associated with various cellular activities (AAA)
AN1593.3	Hydroxymethylglutaryl-coenzyme A reductase
AN1596.3	Glucose/ribitol dehydrogenase
AN1597.3	
AN1599.3	Fungal Zn(2)-Cys(6) binuclear cluster domain
AN1604.3	hypothetical alpha-1,3-glucanase
AN1605.3	
AN1606.3	Annexin
AN1607.3	Glycosyltransferase family 28
AN1608.3	

AN1610.3	
AN1611.3	
AN1614.3	SAM-dependent methyltransferases
AN1616.3	Voltage-gated shaker-like K+ channe
AN1617.3	
AN1619.3	MmgE/PrpD family
AN1621.3	Acetyltransferase (GNAT) family
AN1622.3	
AN1628.3	ATPase, E1-E2 type
AN1631.3	Amino acid/polyamine transporter I
AN1632.3	Atg1
AN1634.3	Prp28
AN1637.3	Mocos
AN1646.3	
AN1647.3	
AN1649.3	
AN1650.3	short chain dehydrogenase
AN1653.3	Phosphatidate cytidylyltransferase
AN1656.3	

AN1658.3	Zinc finger, C3HC4 type (RING finger)
AN1659.3	Amino acid permease
AN1661.3	
AN1670.3	PHD finger protein AF10
AN1673.3	
AN1677.3	short chain dehydrogenase
AN1681.3	Major facilitator superfamily
AN1685.3	PhyB
AN1693.3	
AN1698.3	Spt5
AN1700.3	Proteasome/cyclosome, regulatory subunit
AN1703.3	Cytochrome P450
AN1705.3	
AN1714.3	
AN1717.3	
AN1718.3	
AN1719.3	
AN1720.3	Med8
AN1722.3	Cox10

AN1723.3	
AN1725.3	
AN1726.3	Dehydrogenase E1 component
AN1730.3	
AN1731.3	Proline dehydrogenase
AN1732.3	PutX
AN1733.3	Put2
AN1738.3	
AN1742.3	beta-mannosidase A
AN1743.3	ubiquinone biosynthesis methyltransferase
AN1744.3	Sugar isomerase (SIS)
AN1747.3	Thioesterase superfamily
AN1749.3	
AN1750.3	Mak5
AN1753.3	Fmp52
AN1763.3	short chain dehydrogenase
AN1767.3	
AN1770.3	Ribosomal protein L6
AN1777.3	Zn-finger-like, PHD finger

AN1778.3	PanB
AN1788.3	
AN1791.3	
AN1792.3	Short-chain dehydrogenase
AN1793.3	
AN1795.3	Cation efflux family
AN1796.3	Cell division control protein/predicted DNA repair exonuclease
AN1797.3	Sugar (ANd other) transporter
AN1800.3	Tcsb
AN1801.3	
AN1805.3	Prokaryotic-type carbonic anhydrases
AN1810.3	Oat
AN1811.3	Gpi10
AN1812.3	bZIP transcription factor
AN1814.3	
AN1816.3	
AN1818.3	XynC
AN1824.3	Fungal specific transcription factor
AN1827.3	G protein-coupled receptors

AN1832.3	Ang1
AN1834.3	
AN1837.3	Fungal hydrophobin
AN1840.3	Thiolase
AN1843.3	Protein involved in meiotic recombination/predicted coiled-coil protein
AN1844.3	PalF
AN1847.3	
AN1848.3	Fungal transcriptional regulatory protein, N-terminal
AN1850.3	Peptidase family M48
AN1852.3	Polysaccharide deacetylase
AN1859.3	Af9
AN1860.3	
AN1861.3	Syf2
AN1862.3	
AN1865.3	Sugar (ANd other) transporter
AN1866.3	
AN1869.3	
AN1870.3	Glycoside hydrolase, family 43
AN1881.3	Monooxygenase

AN1882.3	
AN1883.3	Arginosuccinate synthase
AN1887.3	
AN1892.3	Mitochondrial import inner membrane translocase subunit Tim7
AN1893.3	
AN1894.3	
AN1895.3	MaaI
AN1896.3	FaaA
AN1897.3	Hgd
AN1898.3	
AN1899.3	4-hydroxyphenylpyruvate dioxygenase
AN1900.3	
AN1901.3	Cytochrome P450
AN1901.3	Cytochrome P450
AN1907.3	Swf1
AN1915.3	
AN1917.3	Mitochondrial oxoglutarate/malate carrier proteins
AN1918.3	PpcK
AN1919.3	

AN1920.3	Na+/H+ antiporter
AN1925.3	
AN1926.3	
AN1937.3	WetA
AN1940.3	Nucleolar GTPase/ATPase
AN1941.3	
AN1947.3	
AN1948.3	Hypothetical protein
AN1949.3	Has1
AN1950.3	
AN1958.3	
AN1962.3	HMG-box transcription factor
AN1967.3	Cytochrome P450
AN1971.3	Ruvb1
AN1976.3	Eaf3
AN1980.3	Rab6 GTPase activator
AN1981.3	Protein prenyltransferase, alpha subunit
AN1987.3	
AN1999.3	Methyltransferases

AN2000.3	Ubiquitin and ubiquitin-like proteins
AN2001.3	Fungal transcriptional regulatory protein, N-terminal
AN2004.3	
AN2005.3	two-component signal transduction system
AN2013.3	Oxidoreductase, N-terminal
AN2017.3	alpha-glucosidase A
AN2019.3	
AN2020.3	Transcription factor
AN2021.3	Protein-protein-interactor
AN2023.3	
AN2025.3	
AN2029.3	
AN2030.3	
AN2031.3	
AN2032.3	Acyl transferase domain
AN2033.3	Monooxygenase
AN2034.3	
AN2035.3	Acyl transferase domain
AN2036.3	Fungal specific transcription factor domain

AN2037.3	
AN2038.3	
AN2042.3	FAD binding domain
AN2043.3	Amino acid/polyamine transporter I
AN2044.3	
AN2056.3	
AN2057.3	Ribosomal protein L7/L12
AN2058.3	
AN2059.3	Ubiquitin C-terminal hydrolase
AN2060.3	
AN2061.3	Zinc-containing alcohol dehydrogenase
AN2064.3	NRPS
AN2065.3	Pan2
AN2066.3	Hse1
AN2067.3	Ste20
AN2070.3	Transcription factor Engrailed, contains HOX domain
AN2071.3	Vps27
AN2076.3	Atg13
AN2093.3	Mon1

AN2094.3	Dop1
AN2099.3	Aox
AN2103.3	Uncharacterized conserved protein
AN2104.3	N-6 Adenine-specific DNA methylase
AN2112.3	
AN2116.3	
AN2118.3	
AN2123.3	Med7
AN2126.3	CapzA
AN2129.3	Csn5
AN2133.3	Phosphoribosyltransferase
AN2134.3	
AN2137.3	
AN2144.3	UreD urease accessory protein
AN2145.3	
AN2151.3	
AN2155.3	Nbp35
AN2157.3	Peptidase aspartic
AN2162.3	Predicted E3 ubiquitin ligase

AN2165.3	Methyltransferase
AN2166.3	
AN2168.3	Signal transduction
AN2172.3	Ssn8
AN2177.3	short chain dehydrogenase
AN2181.3	Transcription initiation factor IIA, gamma subunit
AN2185.3	DNA/RNA non-specific endonuclease
AN2187.3	
AN2204.3	
AN2205.3	
AN2210.3	ABC transporter
AN2227.3	Glycoside hydrolase, family 3
AN2228.3	
AN2229.3	Met2
AN2230.3	
AN2233.3	Csn6
AN2237.3	Serine carboxypeptidase
AN2240.3	Pbn1
AN2243.3	CarA

AN2245.3	Cwc2
AN2248.3	Gata
AN2249.3	
AN2264.3	Acyl-CoA dehydrogenase
AN2272.3	Adenosine kinase
AN2284.3	Hem1
AN2285.3	Ino80
AN2286.3	Adh3
AN2287.3	Amino acid transporters
AN2289.3	Ribosomal protein S2
AN2290.3	Ste12
AN2291.3	DDE superfamily endonuclease
AN2297.3	
AN2303.3	Smp3
AN2305.3	
AN2311.3	GHMP kinase
AN2314.3	GlgB
AN2315.3	AtpB
AN2319.3	

AN2325.3	Major facilitator superfamily
AN2326.3	Lipase, active site
AN2334.3	Fructose-bisphosphate aldolase class-II
AN2335.3	6-phosphogluconate dehydrogenase
AN2336.3	Phosphoesterase family
AN2338.3	
AN2346.3	Ank repeat
AN2348.3	
AN2351.3	Zinc-containing alcohol dehydrogenase superfamily
AN2358.3	Monocarboxylate transporter
AN2359.3	Glycoside hydrolase, family 3
AN2364.3	Ank repeat
AN2366.3	
AN2370.3	Uncharacterized conserved protein
AN2372.3	Sugar (ANd other) transporter
AN2374.3	
AN2375.3	
AN2376.3	
AN2383.3	

AN2386.3	
AN2387.3	FAD binding domain
AN2388.3	
AN2390.3	Isopenicillin N synthase
AN2398.3	
AN2407.3	Polyprenyl synthetase
AN2412.3	Kcc1
AN2414.3	NADH-ubiquinone oxidoreductase, chain 49kDa
AN2421.3	
AN2424.3	
AN2426.3	Core histone H2A/H2B/H3/H4
AN2427.3	Annexin
AN2435.3	ATP-citrate lyase
AN2436.3	ATP-citrate lyase
AN2441.3	NEDD8-activating complex, APP-BP1/UBA5 component
AN2445.3	
AN2446.3	
AN2453.3	Ppil3
AN2456.3	

AN2463.3	Beta galactosidase small chain, C terminal domain
AN2466.3	Sugar (ANd other) transporter
AN2471.3	
AN2473.3	
AN2479.3	Hypothetical N-acetyl transferase
AN2481.3	Nte1
AN2488.3	
AN2489.3	Ssn3
AN2490.3	
AN2493.3	Alkaline phosphatase
AN2494.3	Adenosine/AMP deaminase
AN2496.3	Efr3
AN2500.3	Nnt1
AN2513.3	Bud32
AN2523.3	ChsB
AN2529.3	Enoyl-CoA hydratase/isomerase
AN2530.3	Hsp30
AN2532.3	Copper amine oxidase
AN2533.3	

AN2538.3	
AN2545.3	AMP-binding enzyme
AN2547.3	Acyl transferase domain
AN2548.3	
AN2549.3	AMP-binding enzyme
AN2555.3	Serine carboxypeptidase
AN2556.3	
AN2557.3	
AN2561.3	
AN2567.3	ABC transporter
AN2574.3	FAD binding domain
AN2577.3	MaoC-like dehydratase
AN2583.3	Glyceraldehyde 3-phosphate dehydrogenase, C-terminal domain
AN2585.3	Sugar (ANd other) transporter
AN2586.3	Phospholipase D. Active site motif
AN2587.3	
AN2588.3	
AN2591.3	Major facilitator superfamily
AN2592.3	

AN2597.3	
AN2601.3	Sugar (ANd other) transporter
AN2612.3	Glycosyl hydrolase family 3 C terminal domain
AN2615.3	Fungal Zn(2)-Cys(6) binuclear cluster domain
AN2621.3	AcvS
AN2622.3	IpnS
AN2623.3	AaaA
AN2629.3	
AN2632.3	Glycosyl hydrolase family 62
AN2639.3	
AN2646.3	Molecular chaperones HSP70/HSC70
AN2647.3	
AN2648.3	FAD/FMN-containing dehydrogenase
AN2649.3	
AN2660.3	
AN2666.3	Zinc-containing alcohol dehydrogenase
AN2675.3	Major facilitator superfamily
AN2683.3	
AN2685.3	

AN2687.3	RhoC
AN2690.3	
AN2701.3	Splicing coactivator SRm160/300,
AN2701.3	Splicing coactivator SRm16/3,
AN2703.3	
AN2704.3	
AN2719.3	Lysozyme
AN2725.3	
AN2727.3	Cytochrome P450
AN2728.3	Dihydrodipicolinate synthetase family
AN2742.3	G-protein, gamma subunit
AN2746.3	Major facilitator superfamily
AN2750.3	
AN2760.3	Splicing coactivator SRm160/300,
AN2772.3	BimE
AN2777.3	Fumarylacetoacetate (FAA) hydrolase family
AN2778.3	Heme-binding domain in cytochrome b5 and oxidoreductases
AN2779.3	Amidohydrolase
AN2788.3	

AN2792.3	
AN2793.3	Isocitrate and isopropylmalate dehydrogenases
AN2797.3	Ank repeat
AN2804.3	
AN2810.3	
AN2815.3	Mannitol dehydrogenase
AN2821.3	Amino acid/polyamine transporter II
AN2826.3	Fungal Zn(2)-Cys(6) binuclear cluster domain
AN2828.3	Glycosyl hydrolase family 3 C terminal domain
AN2837.3	
AN2841.3	
AN2844.3	
AN2846.3	Glutathione peroxidase
AN2851.3	
AN2855.3	PacC
AN2856.3	
AN2858.3	Oxidoreductase, N-terminal
AN2859.3	Dihydrodipicolinate synthetase family
AN2866.3	Oxidoreductase, N-terminal

AN2867.3	Pgm
AN2870.3	Acetyltransferase (GNAT) family
AN2872.3	
AN2873.3	Lys1
AN2881.3	
AN2886.3	
AN2887.3	Atg11
AN2890.3	Esterase/lipase/thioesterase
AN2899.3	
AN2900.3	GatB
AN2901.3	ArgI
AN2912.3	
AN2913.3	
AN2920.3	
AN2921.3	
AN2922.3	
AN2923.3	
AN2924.3	AMP-binding enzyme
AN2926.3	Nsa2

AN2931.3	
AN2932.3	If4A
AN2933.3	
AN2934.3	Ctr copper transporter family
AN2936.3	Glycoside hydrolase, family 38
AN2937.3	
AN2941.3	
AN2943.3	
AN2947.3	Phosphoinositide-specific phospholipase C (PLC)
AN2949.3	Mitochondrial/chloroplast ribosomal protein L54/L37
AN2951.3	UDP-glucose 4-epimerase
AN2953.3	
AN2964.3	E3 binding
AN2974.3	
AN2977.3	Mitochondrial carrier proteins
AN2978.3	
AN2981.3	G6Pd
AN2993.3	
AN3002.3	

AN3003.3	Mov34 family
AN3004.3	Oxr1
AN3006.3	Esterase/lipase/thioesterase
AN3010.3	Trm10
AN3013.3	Glycoside hydrolase, family 5
AN3016.3	
AN3020.3	Peptidase S8 and S53, subtilisin, kexin, sedolisin
AN3021.3	
AN3022.3	Beta-tubulin folding cofactor C
AN3030.3	NADPH-dependent alcohol dehydrogenase (EC 1.1.1.2)
AN3031.3	
AN3033.3	Eukaryotic-type DNA primase, catalytic (small) subunit
AN3036.3	
AN3040.3	Sld2
AN3042.3	
AN3048.3	
AN3057.3	
AN3062.3	Myosin class II heavy chain
AN3064.3	Uncharacterized conserved protein, AMMECR1

AN3067.3	DpoE
AN3068.3	
AN3073.3	tRNA synthetase anti-codon binding domain
AN3076.3	
AN3081.3	Amino acid transporters
AN3085.3	HAD-superfamily hydrolase,
AN3086.3	beta-1,6-N-acetylglucosaminyltransferase
AN3087.3	
AN3095.3	HymA
AN3105.3	Sugar (ANd other) transporter
AN3118.3	Mus81
AN3121.3	
AN3123.3	Hir3
AN3129.3	Poly(ADP-ribose) polymerase, regulatory region
AN3130.3	
AN3131.3	AAA ATPase
AN3136.3	Ubiquitin-conjugating enzymes
AN3139.3	
AN3141.3	

AN3155.3	Receptor-activated Ca2+-permeable cation channels
AN3160.3	
AN3161.3	
AN3163.3	Prohibitins and stomatins of the PID superfamily
AN3175.3	Transferase family
AN3176.3	Spb4
AN3178.3	
AN3180.3	
AN3184.3	
AN3191.3	
AN3195.3	Major facilitator superfamily
AN3196.3	GH family 88
AN3197.3	
AN3198.3	
AN3201.3	Beta galactosidase small chain, C terminal domain
AN3203.3	
AN3204.3	Sugar transporter
AN3205.3	Aldehyde dehydrogenase family
AN3206.3	Glucose-methanol-choline oxidoreductase

AN3207.3	Transmembrane amino acid transporter protein
AN3211.3	
AN3214.3	
AN3216.3	
AN3217.3	Fungal transcriptional regulatory protein,
AN3218.3	
AN3220.3	Sugar (ANd other) transporter
AN3221.3	
AN3222.3	Isn1
AN3223.3	Phosphofructokinase
AN3224.3	Med17
AN3225.3	Cytochrome P450
AN3226.3	
AN3227.3	Monooxygenase
AN3228.3	UbiA prenyltransferase family
AN3229.3	Glucose-methanol-choline oxidoreductase
AN3230.3	Acyl transferase domain
AN3239.3	
AN3249.3	short chain dehydrogenase

AN3253.3	
AN3255.3	Glutathione S-transferase
AN3258.3	
AN3262.3	
AN3264.3	
AN3265.3	
AN3276.3	short chain dehydrogenase
AN3277.3	
AN3278.3	
AN3279.3	short chain dehydrogenase
AN3280.3	
AN3294.3	
AN3304.3	Amino acid/polyamine transporter I
AN3305.3	short chain dehydrogenase
AN3308.3	Alpha amylase
AN3312.3	short chain dehydrogenase
AN3328.3	
AN3329.3	ABC transporter
AN3330.3	

AN3331.3	Metal-dependent phosphohydrolas
AN3333.3	Oxidoreductase family
AN3334.3	
AN3344.3	
AN3345.3	
AN3346.3	
AN3347.3	Amino acid transporters
AN3348.3	
AN3349.3	Cytochrome P450
AN3356.3	Fungal transcriptional regulatory protein
AN3357.3	Sugar (ANd other) transporter
AN3361.3	Bacteriorhodopsin
AN3363.3	BimC
AN3366.3	RPEL repeat
AN3368.3	Glycoside hydrolase
AN3369.3	Fungal specific transcription factor domain
AN3370.3	Gpi14
AN3371.3	
AN3377.3	Peptidase

AN3378.3	Homocysteine S-methyltransferase
AN3380.3	Beta-ketoacyl synthase, C-terminal domain
AN3384.3	
AN3385.3	Fungal Zn(2)-Cys(6) binuclear cluster domain
AN3387.3	
AN3390.3	Pectinesterase
AN3392.3	Monooxygenase
AN3394.3	Cytochrome P450
AN3394.3	Cytochrome P450
AN3395.3	
AN3396.3	AMP-binding enzyme
AN3408.3	
AN3417.3	Acyl-CoA synthetase
AN3418.3	
AN3421.3	
AN3423.3	Spt6
AN3429.3	
AN3433.3	Fungal transcriptional regulatory protein
AN3434.3	Ras small GTPase, Rab type

AN3436.3	Zn-finger, GATA type
AN3437.3	ApsB
AN3439.3	
AN3452.3	Oxysterol-binding protein
AN3470.3	Atg4
AN3474.3	
AN3475.3	
AN3489.3	Predicted membrane protein
AN3491.3	
AN3493.3	Proteasome A-type and B-type
AN3496.3	AMP-binding enzyme
AN3498.3	Major facilitator superfamily
AN3499.3	Protein of unknown function UPF0075
AN3510.3	
AN3512.3	
AN3513.3	
AN3515.3	Sugar (ANd other) transporter
AN3517.3	RNAse3 domain
AN3518.3	beta-1,6-N-acetylglucosaminyltransferase

AN3519.3	
AN3520.3	
AN3522.3	Globin
AN3524.3	Oxidoreductase family
AN3526.3	
AN3529.3	
AN3530.3	
AN3535.3	
AN3543.3	Ank repeat
AN3545.3	short chain dehydrogenase
AN3548.3	
AN3551.3	
AN3552.3	
AN3554.3	
AN3564.3	short chain dehydrogenase
AN3565.3	Metallo-beta-lactamase superfamily
AN3566.3	Glycosyl hydrolase family 47
AN3569.3	Monooxygenase
AN3578.3	Zn-finger, C-x8-C-x5-C-x3-H type

AN3579.3	Predicted membrane protein
AN3580.3	Amine oxidase
AN3581.3	Pyridine nucleotide-disulphide oxidoreductase class-I
AN3584.3	Snx4
AN3585.3	
AN3586.3	Monooxygenase
AN3590.3	
AN3591.3	Aldehyde dehydrogenase
AN3595.3	
AN3600.3	SAM (and some other nucleotide) binding motif
AN3602.3	Ccr4
AN3606.3	
AN3613.3	XynA
AN3616.3	
AN3623.3	Csn7
AN3624.3	AtcS
AN3626.3	AIR carboxylase
AN3634.3	glycosyl transferase family
AN3637.3	

AN3638.3	Sterol desaturase
AN3643.3	
AN3648.3	Cg21
AN3666.3	Psf1
AN3667.3	HMG-box transcription factor
AN3670.3	
AN3672.3	Isopenicillin N synthase
AN3678.3	Vacuolar sorting protein 9 (VPS9) domain
AN3679.3	short chain dehydrogenase
AN3683.3	Fungal specific transcription factor
AN3690.3	Mitochondrial substrate carrier
AN3703.3	
AN3707.3	Peptidase M20
AN3708.3	
AN3714.3	
AN3717.3	Methyltransferases
AN3719.3	MAP kinase
AN3720.3	Sec24
AN3722.3	Major facilitator superfamily

AN3731.3	
AN3732.3	Coeffector of mDia Rho GTPase, regulates actin polymerization and cell adhesion turnover
AN3733.3	
AN3734.3	Atg9
AN3739.3	RNA recognition motif. (a.k.a. RRM, RBD, or RNP domain)
AN3740.3	
AN3741.3	Adh2
AN3744.3	
AN3747.3	Prenyltransferase/squalene oxidase
AN3753.3	
AN3763.3	Predicted transporter
AN3764.3	
AN3765.3	
AN3769.3	
AN3776.3	Permease of the major facilitator superfamily
AN3777.3	
AN3783.3	
AN3787.3	Png1
AN3791.3	

AN3799.3	ZIP Zinc transporter
AN3802.3	
AN3806.3	Histone deacetylase family
AN3811.3	Transcription-coupled repair protein
AN3813.3	Ctr copper transporter family
AN3816.3	
AN3818.3	
AN3820.3	
AN3831.3	Uncharacterized protein, induced by hypoxia
AN3832.3	Efg
AN3839.3	Nmt
AN3842.3	Ras GTPase
AN3848.3	
AN3853.3	Cym1
AN3855.3	2-nitropropane dioxygenase
AN3856.3	
AN3858.3	
AN3869.3	GHMP kinase
AN3871.3	Ank repeat

AN3872.3	
AN3873.3	
AN3881.3	
AN3883.3	
AN3888.3	Major facilitator superfamily
AN3895.3	L-carnitine dehydratase/bile acid-inducible protein F
AN3897.3	Phenylalanine ammonia-lyase
AN3905.3	Ptpa1
AN3917.3	Cytochrome P450
AN3918.3	Peptidase M28
AN3920.3	protoporphyrinogen oxidase
AN3926.3	WD40 repeat-containing protein
AN3927.3	Sphingomyelinase family protein
AN3941.3	MpiP
AN3943.3	Peptidase C15, pyroglutamyl peptidase I
AN3952.3	ABC transporter
AN3954.3	6Pgd
AN3957.3	Amidases
AN3961.3	SCO1/SenC

AN3970.3	
AN3973.3	Alkyl hydroperoxide reductase
AN3976.3	
AN3979.3	
AN3982.3	Calcineurin-like phosphoesterase
AN3983.3	
AN3987.3	
AN3995.3	Zinc-binding oxidoreductase
AN3996.3	
AN3998.3	
AN4000.3	PabP
AN4008.3	
AN4010.3	
AN4011.3	Adenylosuccinate lyase
AN4019.3	Major facilitator superfamily
AN4020.3	Uncharacterised protein family (UPF0136)
AN4023.3	
AN4025.3	Survival protein SurE
AN4035.3	AmdR

AN4051.3	
AN4052.3	Cellulase (glycosyl hydrolase family 5)
AN4055.3	Phosphoesterase
AN4058.3	Dihydroxy-acid dehydratase
AN4068.3	Hut1
AN4070.3	
AN4071.3	3-oxo-5-alpha-steroid 4-dehydrogenase, C-terminal
AN4072.3	
AN4074.3	
AN4076.3	Serine/threonine protein kinase
AN4077.3	
AN4081.3	Cysteine dioxygenase CDO1
AN4082.3	Glycosyl transferase, family 8
AN4089.3	
AN4094.3	Ergosterol biosynthesis ERG4/ERG24 family
AN4102.3	Glycosyl hydrolase family 3 C terminal domain
AN4111.3	Taurine catabolism dioxygenase TauD, TfdA family
AN4113.3	His Kinase A (phosphoacceptor) domain
AN4117.3	Cytochrome P450

AN4119.3	Major facilitator superfamily
AN4121.3	
AN4122.3	
AN4127.3	
AN4129.3	
AN4135.3	Fatty acid desaturase
AN4136.3	
AN4146.3	
AN4148.3	Sugar (ANd other) transporter
AN4151.3	
AN4159.3	GlnA
AN4165.3	Erv25
AN4166.3	CreC
AN4167.3	Gem1
AN4170.3	
AN4172.3	
AN4181.3	
AN4182.3	Cdc2
AN4195.3	Amidase

AN4196.3	
AN4197.3	
AN4198.3	
AN4199.3	
AN4201.3	AMP-binding enzyme
AN4208.3	Sterol O-acyltransferase
AN4210.3	Signaling protein SWIFT and related BRCT domain proteins
AN4211.3	
AN4212.3	
AN4216.3	
AN4223.3	
AN4224.3	
AN4227.3	short chain dehydrogenase
AN4228.3	
AN4233.3	Rrp3
AN4241.3	Major facilitator superfamily
AN4245.3	May be involved in signal transduction
AN4251.3	Ribosomal protein S2
AN4256.3	

AN4264.3	
AN4267.3	Putative ubiquitin fusion degradation protein
AN4273.3	C2 domain
AN4280.3	Mitochondrial carrier proteins
AN4289.3	retroviral pol related endonuclease
AN4292.3	
AN4294.3	Acyl transferase region
AN4299.3	
AN4305.3	Flavoprotein
AN4306.3	Putative transcription factor
AN4313.3	
AN4314.3	Splicing coactivator SRm160/300,
AN4316.3	Major facilitator superfamily
AN4317.3	Sec13
AN4328.3	Amino acid/polyamine transporter I
AN4332.3	Longevity-assurance protein (LAG1)
AN4343.3	
AN4348.3	
AN4351.3	PalA

AN4354.3	
AN4355.3	Hypothetical Delta 1-pyrroline-5-carboxylate reductase (EC 1.5.1.2)
AN4359.3	
AN4365.3	DNA mismatch repair protein
AN4372.3	Glycoside hydrolase, family 28
AN4375.3	
AN4376.3	Dhe4
AN4377.3	Med5
AN4378.3	
AN4381.3	
AN4384.3	Folylpolyglutamate synthetase
AN4392.3	
AN4394.3	
AN4400.3	
AN4405.3	Fatty acid desaturase
AN4407.3	Rad52
AN4408.3	
AN4409.3	Otc
AN4415.3	Cytochrome c and c1 heme-lyase

AN4418.3	Zn-finger, C2H2 type
AN4422.3	Eukaryotic aspartyl protease
AN4424.3	FMN-dependent alpha-hydroxy acid dehydrogenase
AN4430.3	Amino acid-binding ACT
AN4445.3	Swc4
AN4461.3	Spp2
AN4462.3	Biotin carboxylase C-terminal domain
AN4467.3	Peptidyl-prolyl cis-trans isomerase
AN4472.3	Vid21
AN4474.3	
AN4480.3	
AN4482.3	ABC transporter
AN4483.3	Protein kinase
AN4490.3	Tim50
AN4495.3	
AN4503.3	
AN4504.3	GH family 76
AN4518.3	WD domain, G-beta repeat
AN4527.3	Transcription factor, Myb superfamily

AN4528.3	ER lumen protein retaining receptor
AN4532.3	Intradiol ring-cleavage dioxygenase
AN4539.3	Yippee-type zinc-binding protein
AN4552.3	Ku80
AN4553.3	
AN4556.3	
AN4562.3	Basic-leucine zipper (bZIP) transcription factor
AN4566.3	Chs1
AN4569.3	Ubiquinone biosynthesis protein COQ7
AN4576.3	Monooxygenase
AN4577.3	Acetamidase/Formamidase
AN4583.3	PpiD
AN4585.3	CCR4-NOT transcriptional regulation complex
AN4586.3	Zinc finger, C2H2 type
AN4590.3	Major facilitator superfamily
AN4592.3	Fatty acid desaturase
AN4593.3	Ribosomal protein S2
AN4598.3	
AN4599.3	Grc3

AN4601.3	Atg26
AN4603.3	
AN4609.3	
AN4623.3	
AN4627.3	
AN4646.3	
AN4659.3	AMP-dependent synthetase and ligase
AN4661.3	
AN4666.3	Hsv2
AN4668.3	MpkC
AN4672.3	Predicted PRP38-like splicing factor
AN4691.3	Short-chain dehydrogenase/reductase SDR
AN4697.3	Pfa5
AN4699.3	SWI-SNF chromatin-remodeling complex protein
AN4701.3	
AN4702.3	
AN4707.3	Isy1
AN4709.3	Phosphoinositide 3-kinase, C2
AN4717.3	Protein kinase

AN4720.3	Zinc finger, C2H2 type
AN4722.3	
AN4729.3	
AN4741.3	Srb8
AN4744.3	Fungal transcriptional regulatory protein, N-terminal
AN4750.3	
AN4755.3	
AN4757.3	Protein involved in ubiquinone biosynthesis
AN4763.3	ErfB
AN4767.3	WASP-interacting protein VRP1/WIP
AN4769.3	Met3
AN4770.3	Met16
AN4772.3	Deoxyribose-phosphate aldolase
AN4776.3	Phospholipase/carboxyhydrolase
AN4783.3	Csn2
AN4788.3	Slu7
AN4791.3	Myosin class II heavy chain
AN4792.3	Proteins containing the FAD binding domain
AN4795.3	

AN4796.3	
AN4799.3	FOG: RRM domain
AN4806.3	
AN4809.3	
AN4812.3	
AN4819.3	FluG
AN4820.3	Aldehyde dehydrogenase
AN4829.3	Aldo/keto reductase family proteins
AN4830.3	phosphopantothenoylcysteine synthetase/decarboxylase
AN4833.3	
AN4837.3	Fungal specific transcription factor
AN4839.3	Serine/threonine protein kinase
AN4842.3	Putative cytochrome C oxidase assembly protein
AN4845.3	
AN4846.3	
AN4848.3	
AN4852.3	
AN4853.3	PalI
AN4857.3	ABC1 family

AN4859.3	Plasma membrane H+-transporting ATPase
AN4866.3	Nucleolar GTPase/ATPase p130
AN4871.3	Glycosyl hydrolases family 18
AN4874.3	Rny1
AN4875.3	Uncharacterized ACR, YagE family COG1723
AN4876.3	Cytochrome c heme-binding site
AN4884.3	ER to golgi transport protein
AN4888.3	Pdc
AN4889.3	
AN4891.3	Asf1
AN4892.3	Rna14
AN4903.3	Dbp8
AN4904.3	HCO3-transporter family
AN4906.3	Ferric reductase like transmembrane component
AN4910.3	
AN4912.3	
AN4913.3	Phk
AN4917.3	Dynactin
AN4920.3	Cation transporting ATPase, C-terminus

AN4921.3	
AN4923.3	Hydroxymethylglutaryl-coenzyme A synthase
AN4929.3	Ankyrin repeat
AN4941.3	
AN4946.3	
AN4952.3	Nst1
AN4958.3	
AN4961.3	Disintegrin metalloproteinases with thrombospondin repeats
AN4969.3	Ndc80
AN4974.3	
AN4976.3	Tbp
AN4983.3	Phosphoglycerate mutase
AN4987.3	KapR
AN4989.3	
AN4990.3	Integral membrane protein
AN5001.3	3'-5' exonuclease
AN5008.3	
AN5011.3	
AN5013.3	Rexo3

AN5015.3	
AN5017.3	Short-chain dehydrogenase
AN5019.3	Methionine synthase II
AN5021.3	Glycosyl transferases group 1
AN5022.3	Dynactin, subunit p25
AN5028.3	Animal haem peroxidase
AN5034.3	
AN5037.3	Pyridine nucleotide-disulphide oxidoreductase, class I
AN5038.3	
AN5049.3	Cation efflux family
AN5050.3	Sugar (ANd other) transporter
AN5051.3	FOG: Predicted E3 ubiquitin ligase
AN5053.3	
AN5060.3	
AN5067.3	Major facilitator superfamily
AN5068.3	
AN5069.3	
AN5076.3	LysM domain
AN5079.3	

AN5083.3	V-type ATPase 116kDa subunit family
AN5086.3	
AN5094.3	
AN5102.3	Spt16
AN5104.3	Sugar transporter
AN5109.3	Aldo/keto reductase
AN5114.3	Ribonuclease HII
AN5117.3	
AN5119.3	
AN5130.3	
AN5132.3	Mitochondrial substrate carrier
AN5140.3	Major facilitator superfamily
AN5145.3	
AN5146.3	FMN-dependent dehydrogenase
AN5149.3	
AN5155.3	Subunit of tRNA-specific adenosine-34 deaminase
AN5157.3	
AN5168.3	Ank repeat
AN5169.3	RNA polymerase II, large subunit

AN5170.3	Fungal Zn(2)-Cys(6) binuclear cluster domain
AN5174.3	Atg5
AN5175.3	Predicted hydrolase
AN5179.3	Dph2
AN5183.3	Alpha/beta hydrolase
AN5192.3	AMP-dependent synthetase and ligase
AN5203.3	Ribosomal protein S8
AN5204.3	
AN5206.3	Isocitrate/isopropylmalate dehydrogenase
AN5208.3	
AN5210.3	KpyK
AN5219.3	
AN5220.3	Fungal specific transcription factor
AN5223.3	
AN5226.3	GPR1/FUN34/yaaH
AN5228.3	hypothetical NADH:flavin oxidoreductase/NADH oxidase
AN5231.3	
AN5237.3	
AN5245.3	

AN5255.3	
AN5274.3	Fungal specific transcription factor domain
AN5276.3	
AN5281.3	P2Ox
AN5283.3	
AN5290.3	
AN5296.3	Tcsa
AN5303.3	
AN5305.3	
AN5311.3	
AN5312.3	
AN5314.3	
AN5318.3	
AN5320.3	
AN5324.3	Transcription elongation factor
AN5329.3	Major facilitator superfamily
AN5330.3	
AN5331.3	Short-chain dehydrogenase/reductase SDR
AN5332.3	

AN5333.3	
AN5335.3	
AN5338.3	
AN5342.3	
AN5348.3	
AN5350.3	
AN5351.3	Ubiquitin-conjugating enzymes
AN5353.3	
AN5355.3	HMMPfam indicates Zinc-containing alcohol dehydrogenase activity
AN5357.3	
AN5359.3	
AN5364.3	von Willebrand factor type A domain
AN5368.3	Short-chain dehydrogenase/reductase SDR
AN5373.3	short chain dehydrogenase
AN5379.3	short chain dehydrogenase
AN5383.3	Uncharacterized conserved protein
AN5389.3	
AN5390.3	Fungal transcriptional regulatory protein
AN5393.3	

AN5395.3	
AN5396.3	
AN5398.3	
AN5400.3	Flavin-containing monooxygenase
AN5402.3	Patatin
AN5406.3	DNA topoisomerase II
AN5407.3	NRPS
AN5408.3	
AN5410.3	
AN5411.3	HD domain
AN5412.3	
AN5413.3	
AN5414.3	
AN5421.3	Flavin-containing monooxygenase
AN5422.3	Beta-lactamase
AN5423.3	
AN5429.3	TAP-like protein
AN5434.3	
AN5435.3	Aldehyde dehydrogenase family

AN5437.3	
AN5440.3	Ccpr2
AN5442.3	Peptidase S10, serine carboxypeptidase
AN5444.3	Pyridoxal-phosphate dependent enzyme
AN5447.3	Pyridoxal-dependent decarboxylase
AN5449.3	Sulfatase
AN5452.3	Rse1
AN5453.3	
AN5455.3	Utp10
AN5458.3	
AN5461.3	
AN5464.3	
AN5466.3	
AN5467.3	
AN5468.3	
AN5478.3	
AN5480.3	
AN5487.3	
AN5490.3	

AN5491.3	Atg2
AN5492.3	Adenosine/AMP deaminase
AN5494.3	Eukaryotic protein kinase domain. Related to cell cycle checkpoints
AN5502.3	
AN5503.3	
AN5508.3	
AN5510.3	
AN5516.3	HORMA domain
AN5519.3	Clathrin adaptor complex small chain
AN5523.3	Tps1
AN5528.3	Atm1
AN5540.3	ATP binding protein
AN5550.3	FAD binding domain
AN5556.3	short chain dehydrogenase
AN5559.3	
AN5565.3	
AN5566.3	Guaa
AN5586.3	Mpg1
AN5587.3	Nucleic acid-binding OB-fold

AN5596.3	Permease of the major facilitator superfamily
AN5601.3	Saccharopine dehydrogenase
AN5602.3	
AN5610.3	Aminoadipate-semialdehyde dehydrogenase
AN5611.3	Prokaryotic-type carbonic anhydrases
AN5612.3	
AN5613.3	Xdh
AN5618.3	EF hand, invovled in cell cycle control
AN5621.3	Nucleolar GTPase/ATPase p130
AN5624.3	Hypothetical protein
AN5626.3	AcsA
AN5633.3	
AN5634.3	AceA
AN5635.3	TreB
AN5653.3	short chain dehydrogenase
AN5655.3	5'-nucleotidase, C-terminal domain
AN5656.3	Major facilitator superfamily
AN5657.3	hypothetical PHD zinc finger
AN5660.3	Aldehyde dehydrogenase

AN5663.3	
AN5664.3	
AN5665.3	Cytochrome P450
AN5666.3	Protein kinase
AN5672.3	Mandelate racemase
AN5673.3	Fungal specific transcription factor
AN5674.3	Eukaryotic protein kinase domain. Involved in signal transduction
AN5678.3	Amino acid permease
AN5680.3	3'-5' exonuclease
AN5687.3	
AN5689.3	
AN5690.3	Copper amine oxidase
AN5695.3	Glutathione-dependent formaldehyde-activating, GFA
AN5714.3	
AN5725.3	Glycosyl transferases group 1
AN5726.3	Zn-finger, C2H2 type
AN5731.3	AroC
AN5733.3	
AN5740.3	RhoA

AN5741.3	Ribosomal protein L24
AN5743.3	Atcl
AN5752.3	Med18
AN5758.3	Rt106
AN5762.3	
AN5763.3	Major facilitator superfamily
AN5764.3	Dystonin, GAS (Growth-arrest-specific protein), and related proteins
AN5777.3	Esterase/lipase/thioesterase
AN5780.3	
AN5785.3	Pno1
AN5786.3	
AN5788.3	Protein of unknown function (DUF396)
AN5791.3	
AN5795.3	Set1
AN5798.3	Csn3
AN5806.3	Transcriptional coactivator p15
AN5810.3	Peptidase M24
AN5812.3	LkhA4
AN5815.3	Eukaryotic protein kinase domain. Involved in signal transduction

AN5818.3	
AN5819.3	Ras GTPase
AN5821.3	
AN5823.3	
AN5824.3	Akr1
AN5829.3	
AN5831.3	
AN5833.3	
AN5836.3	StuA
AN5844.3	
AN5845.3	
AN5846.3	putative FAD/FMN-containing dehydrogenase
AN5847.3	
AN5849.3	Putatively involved in growth development in niger
AN5852.3	Enoyl-CoA hydratase/isomerase family
AN5860.3	Sugar (ANd other) transporter
AN5863.3	DDE superfamily endonuclease
AN5876.3	
AN5878.3	Hypothetical acetyl-CoA acetyltransferase

AN5885.3	
AN5886.3	Leu2
AN5890.3	Amino acid/polyamine transporter I
AN5893.3	FlbA
AN5901.3	Psf2
AN5902.3	Alg10
AN5903.3	
AN5906.3	
AN5907.3	Ribose/Galactose Isomerase
AN5908.3	Triosephosphate isomerase
AN5917.3	Sugar (ANd other) transporter
AN5919.3	Atg15
AN5924.3	Fungal transcriptional regulatory protein, N-terminal
AN5931.3	Dbp2
AN5935.3	
AN5936.3	Trypsin Inhibitor like cysteine rich domain
AN5938.3	
AN5939.3	5'-nucleotidase, C-terminal domain
AN5942.3	

AN5945.3	
AN5946.3	
AN5953.3	HesB-like domain
AN5963.3	
AN5966.3	Zn-finger, C2H2 type
AN5968.3	Amino acid/polyamine transporter I
AN5984.3	Oxidoreductase family
AN5985.3	
AN6001.3	Glyoxylase
AN6002.3	Monooxygenase
AN6005.3	
AN6010.3	DnaK
AN6013.3	Cytosine deaminase FCY1 and related enzymes
AN6015.3	
AN6016.3	
AN6018.3	
AN6019.3	
AN6031.3	2-nitropropane dioxygenase
AN6034.3	Uncharacterized conserved protein

AN6035.3	Mandelate racemase/muconate lactonizing enzyme
AN6046.3	Sorbin and SH3 domain-containing protein
AN6048.3	Aspartate/other aminotransferase
AN6050.3	
AN6052.3	
AN6058.3	
AN6061.3	Universal stress protein family
AN6062.3	
AN6065.3	Chitinase
AN6066.3	Isochorismatase hydrolase
AN6069.3	ATP-dependent DNA ligase
AN6072.3	Rmt2
AN6075.3	Phenylalanine and histidine ammonia-lyases
AN6077.3	NADH dehydrogenase (ubiquinone), 24 kDa subunit
AN6078.3	Ada
AN6084.3	
AN6087.3	Mitochondrial substrate carrier
AN6089.3	Ch60
AN6091.3	Fungal specific transcription factor domain

AN6095.3	Sugar (ANd other) transporter
AN6097.3	
AN6100.3	
AN6101.3	Cytochrome P450
AN6108.3	
AN6113.3	
AN6116.3	
AN6118.3	Amino acid permease
AN6125.3	Nde1
AN6127.3	ATP synthase subunit C
AN6128.3	
AN6135.3	
AN6138.3	BimA
AN6146.3	Ribosomal protein L14
AN6147.3	Set9
AN6149.3	Mrh4
AN6156.3	DDE superfamily endonuclease
AN6157.3	PyrF
AN6158.3	

AN6159.3	Sterol O-acyltransferase/Diacylglycerol O-acyltransferase
AN6160.3	Pfs2
AN6167.3	NADH:flavin oxidoreductase/NADH oxidase
AN6172.3	
AN6178.3	RNA polymerase II transcription elongation factor
AN6180.3	
AN6185.3	
AN6193.3	Peptidase S16, lon protease
AN6195.3	CreA
AN6197.3	NudF
AN6201.3	
AN6208.3	
AN6210.3	Exo70
AN6214.3	Hat1
AN6218.3	
AN6227.3	
AN6233.3	DnaJ domain
AN6241.3	Serine/threonine protein kinase
AN6246.3	Cytochrome c

AN6249.3	
AN6253.3	Phenylalanyl-tRNA synthetase
AN6254.3	Mitochondrial carrier protein
AN6257.3	Sec31
AN6259.3	
AN6272.3	Ferredoxin/adrenodoxin reductase
AN6274.3	short chain dehydrogenase
AN6281.3	
AN6283.3	Dph1
AN6288.3	Mob1/phocein family
AN6289.3	
AN6299.3	
AN6301.3	
AN6306.3	Tim10/DDP family zinc finger
AN6316.3	DNA mismatch repair protein
AN6317.3	Chitin synthase/hyaluronan synthase
AN6323.3	Microtubule-associated protein
AN6327.3	von Willebrand factor
AN6337.3	

AN6339.3	Protein kinase
AN6340.3	KlpA
AN6348.3	Mitochondrial import inner membrane translocase, subunit TIM23
AN6351.3	Snx41
AN6359.3	SconB
AN6360.3	Atg17
AN6362.3	Involved in cell cycle control
AN6364.3	SudA
AN6365.3	
AN6369.3	ABC transporter
AN6372.3	
AN6373.3	
AN6374.3	Dbp9
AN6375.3	
AN6380.3	
AN6382.3	
AN6386.3	Oxidoreductase FAD-binding domain
AN6387.3	Vesicular amine transporter
AN6391.3	Pp2A1

AN6399.3	Peptidase C1-like family
AN6400.3	Ferric reductase like transmembrane component
AN6401.3	
AN6403.3	3-dehydroquinate synthase
AN6404.3	
AN6405.3	Glycosyl hydrolases family 25
AN6412.3	
AN6413.3	
AN6418.3	
AN6419.3	
AN6422.3	
AN6423.3	
AN6424.3	
AN6426.3	
AN6430.3	Fungal specific transcription factor domain
AN6436.3	ABC transporter
AN6438.3	Dipeptidyl peptidase IV (DPP IV) N-terminal region
AN6442.3	Transmembrane amino acid transporter protein
AN6443.3	ABC transporter

AN6460.3	
AN6468.3	
AN6470.3	Glycosyl hydrolases family 25
AN6471.3	
AN6472.3	GH family 76
AN6473.3	
AN6477.3	General substrate transporter
AN6480.3	
AN6481.3	
AN6489.3	Rt10
AN6496.3	
AN6506.3	Sterol desaturase
AN6512.3	ABC transporter
AN6514.3	Dom3Z
AN6521.3	Lys4
AN6523.3	SepA
AN6524.3	
AN6525.3	Fdh
AN6526.3	Syl

AN6535.3	
AN6537.3	
AN6542.3	Actg
AN6543.3	Fes1
AN6549.3	Med6
AN6550.3	Glutamine amidotransferase class-I
AN6552.3	
AN6554.3	Histone
AN6567.3	Epl1
AN6569.3	Kae1
AN6571.3	
AN6572.3	ABC (ATP binding cassette) 1 protein
AN6587.3	Translational repressor
AN6589.3	
AN6593.3	
AN6610.3	Dolichyl pyrophosphate phosphatase and related acid phosphatases
AN6614.3	ATPase, E1-E2 type
AN6615.3	Sec16
AN6616.3	Possibly related to mitotic and DNA damage checkpoint protein hus

AN6620.3	
AN6621.3	
AN6622.3	
AN6624.3	
AN6626.3	Mpv17 / PMP22 family
AN6633.3	
AN6635.3	Lac1
AN6636.3	Aldehyde dehydrogenase family
AN6640.3	Peptidase family M20/M25/M40
AN6644.3	adenosylmethionine-8-amino-7-oxononanoate transaminase
AN6645.3	Aminotransferase
AN6650.3	PrpC
AN6653.3	MasY
AN6657.3	
AN6658.3	
AN6663.3	Ank repeat
AN6670.3	
AN6675.3	Zn-finger-like, PHD finger
AN6678.3	Uncharacterized conserved protein

AN6686.3	
AN6687.3	Pob3
AN6693.3	
AN6694.3	Ctf18
AN6702.3	Bst1
AN6703.3	Sugar (ANd other) transporter
AN6703.3	Major facilitator superfamily
AN6704.3	
AN6712.3	Phospholipase D. Active site motif
AN6713.3	
AN6718.3	ATPases associated with various cellular activities (AAA)
AN6723.3	Amidohydrolase 2
AN6727.3	
AN6730.3	UapC
AN6736.3	
AN6740.3	
AN6741.3	Ddi1
AN6743.3	Peptidase S15
AN6750.3	Flavoprotein monooxygenase

AN6753.3	NADH:flavin oxidoreductase/NADH oxidase
AN6755.3	Acyl-CoA dehydrogenase
AN6761.3	Acyl-CoA dehydrogenase, central region
AN6763.3	
AN6767.3	Enoyl-CoA hydratase/isomerase family
AN6771.3	carboxylesterase
AN6772.3	
AN6775.3	Peptidase C45
AN6776.3	
AN6780.3	
AN6786.3	Glycosyl hydrolase family 45
AN6787.3	Cytochrome P450
AN6788.3	Fungal specific transcription factor domain
AN6789.3	
AN6791.3	Polyketide synthase
AN6793.3	
AN6796.3	
AN6798.3	CoA-transferase
AN6804.3	Sugar (ANd other) transporter

AN6806.3	
AN6809.3	
AN6810.3	DJ-1/PfpI family
AN6821.3	
AN6833.3	
AN6834.3	Sugar (ANd other) transporter
AN6836.3	Bacterial transferase hexapeptide (four repeats)
AN6838.3	Tbb2
AN6839.3	
AN6845.3	Iron transporter
AN6847.3	Sulfatase
AN6856.3	
AN6860.3	
AN6861.3	
AN6862.3	
AN6865.3	Bcp1
AN6876.3	
AN6878.3	
AN6879.3	Mch1

AN6880.3	Inositol polyphosphate kinase
AN6881.3	
AN6884.3	
AN6885.3	Calcium-responsive transcription coactivator
AN6886.3	PalH
AN6888.3	Aspartyl protease
AN6890.3	
AN6894.3	Ppil2
AN6897.3	
AN6900.3	TpiS
AN6908.3	Signal transduction protein with FHA domain
AN6909.3	
AN6910.3	
AN6913.3	Peptidase C19, ubiquitin carboxyl-terminal hydrolase 2
AN6918.3	short chain dehydrogenase
AN6923.3	Sugar (ANd other) transporter
AN6924.3	
AN6928.3	
AN6930.3	Aminotransferases class-III pyridoxal-phosphate

AN6932.3	UapA
AN6933.3	Malic enzyme
AN6940.3	CorA-like Mg2+ transporter protein
AN6941.3	
AN6943.3	
AN6944.3	
AN6945.3	O-methyltransferase
AN6946.3	
AN6948.3	Glycosyl hydrolases family 16
AN6950.3	alpha/beta hydrolase fold
AN6952.3	O-methyltransferase
AN6954.3	
AN6960.3	Ank repeat
AN6963.3	
AN6964.3	
AN6969.3	
AN6973.3	Sterol desaturase
AN6982.3	Tyrosine specific protein phosphatase
AN6983.3	

AN6985.3	Ribulose kinase and related carbohydrate kinases
AN6986.3	Rhodopsin-like GPCR superfamily
AN6996.3	Protein kinase
AN7006.3	
AN7007.3	Tof1
AN7009.3	Arp6
AN7010.3	Phenazine biosynthesis-like protein
AN7026.3	Heavy metal transport/detoxification protein
AN7027.3	Permease of the major facilitator superfamily
AN7032.3	ChsA
AN7033.3	
AN7035.3	Peptidase M28
AN7037.3	Vacuolar sorting protein VPS36
AN7038.3	
AN7040.3	endoribonuclease
AN7046.3	
AN7049.3	Mcd4
AN7053.3	
AN7058.3	

AN7074.3	short chain dehydrogenase
AN7075.3	FAD binding domain
AN7081.3	
AN7090.3	
AN7091.3	
AN7092.3	
AN7093.3	
AN7101.3	beta-1,6-N-acetylglucosaminyltransferase, contains WSC domain
AN7109.3	Ank repeat
AN7110.3	
AN7128.3	short chain dehydrogenase
AN7130.3	
AN7133.3	
AN7138.3	
AN7139.3	
AN7140.3	
AN7142.3	Phosphoesterase family
AN7145.3	Slt11
AN7148.3	

AN7149.3	
AN7150.3	Amino acid transporters
AN7152.3	Glycoside hydrolase, family 27
AN7156.3	Oxidoreductase family
AN7165.3	Protein required for meiotic chromosome segregation
AN7169.3	Globin
AN7170.3	Helix-loop-helix DNA-binding domain
AN7171.3	
AN7173.3	Sodium/calcium exchanger protein
AN7175.3	SAM-dependent methyltransferases
AN7177.3	
AN7183.3	
AN7184.3	Cwc26
AN7187.3	FAD dependent oxidoreductase
AN7188.3	OPT oligopeptide transporter protein
AN7190.3	
AN7191.3	
AN7200.3	
AN7201.3	Related to subtilisin

AN7204.3	Fatty acid desaturase
AN7206.3	Ras small GTPase, Ras type
AN7208.3	
AN7211.3	
AN7217.3	
AN7222.3	FAD binding domain
AN7223.3	
AN7225.3	Major facilitator superfamily
AN7228.3	FAD-dependent pyridine nucleotide-disulphide oxidoreductase
AN7229.3	Sulfatase
AN7231.3	
AN7232.3	
AN7233.3	alpha/beta hydrolase fold
AN7243.3	
AN7251.3	Monooxygenase
AN7259.3	Dcn1
AN7263.3	
AN7264.3	
AN7265.3	

AN7266.3	
AN7267.3	GMC oxidoreductase
AN7268.3	short chain dehydrogenase
AN7269.3	
AN7270.3	
AN7271.3	
AN7275.3	
AN7278.3	Pyridoxal-dependent decarboxylase conserved domain
AN7280.3	
AN7283.3	
AN7295.3	Major facilitator superfamily
AN7298.3	
AN7301.3	Alg8
AN7305.3	Esf2
AN7307.3	
AN7309.3	Rad18
AN7316.3	Metallo-beta-lactamase superfamily
AN7317.3	GPR1/FUN34/yaaH family
AN7323.3	

AN7327.3	
AN7334.3	Phospholipase D/Transphosphatidylase
AN7336.3	
AN7341.3	
AN7343.3	Fungal Zn(2)-Cys(6) binuclear cluster domain
AN7347.3	
AN7352.3	
AN7353.3	Oxidoreductase family
AN7357.3	
AN7362.3	
AN7364.3	Protein of unknown function (DUF455)
AN7382.3	Flavoprotein monooxygenase
AN7386.3	
AN7388.3	
AN7389.3	Multicopper oxidase
AN7392.3	Amino acid/polyamine transporter I
AN7399.3	Cytochrome P450
AN7399.3	Cytochrome P450
AN7400.3	Cytochrome P450

AN7406.3	
AN7407.3	Carboxylesterases
AN7414.3	
AN7424.3	Dbp3
AN7426.3	Oligosaccharyltransferase, gamma subunit
AN7428.3	Atg7
AN7430.3	His5
AN7436.3	Thioredoxin domain 2
AN7437.3	At221
AN7438.3	Med14
AN7441.3	Arp4
AN7455.3	Jhd1
AN7463.3	Ammonium Transporter Family
AN7466.3	Major facilitator superfamily
AN7469.3	Riboflavin kinase / FAD synthetase
AN7470.3	
AN7479.3	cytosolic asparaginyl-tRNA synthetase
AN7482.3	
AN7484.3	

AN7485.3	MirC
AN7488.3	Arginase/agmatinase/formiminoglutamase
AN7498.3	DohH
AN7500.3	EF hand
AN7505.3	Glycosyl hydrolases family 31
AN7506.3	Iml1
AN7519.3	
AN7524.3	Lipolytic enzyme
AN7526.3	Bfr2
AN7528.3	
AN7532.3	
AN7534.3	
AN7539.3	
AN7548.3	Endoplasmic reticulum protein EP58
AN7549.3	ATPases associated with various cellular activities (AAA)
AN7551.3	
AN7553.3	Transcription factor with Helix-loop-helix DNA-binding domain
AN7560.3	PalC
AN7569.3	Tpc1

AN7570.3	Tba2
AN7580.3	
AN7591.3	At222
AN7594.3	
AN7596.3	
AN7600.3	Sir
AN7607.3	
AN7617.3	
AN7621.3	Predicted oxidoreductase
AN7624.3	Glycoside hydrolase, family 27
AN7629.3	
AN7635.3	
AN7636.3	Zinc-containing alcohol dehydrogenase superfamily
AN7659.3	Dbp5
AN7661.3	Eukaryotic transcription factor with helix-loop-helix DNA-binding domain
AN7662.3	Ferric reductase like transmembrane component
AN7663.3	D-isomer specific 2-hydroxyacid dehydrogenase, NAD-binding
AN7666.3	Predicted sugar kinase
AN7667.3	Major facilitator superfamily

AN7668.3	GAF domain
AN7672.3	Anp1
AN7674.3	Nop16
AN7686.3	FHA domain
AN7691.3	Phosphoesterase family
AN7692.3	
AN7701.3	Ribosomal protein S16
AN7708.3	Aldo/keto reductase family proteins
AN7710.3	Haloacid dehalogenase-like hydrolase
AN7713.3	
AN7714.3	
AN7717.3	
AN7720.3	Cytochrome c heme-binding site
AN7725.3	Pdx1
AN7727.3	
AN7734.3	Helix loop helix transcription factor EB
AN7735.3	
AN7753.3	Ku70
AN7755.3	Cwc24

AN7757.3	ApsA
AN7762.3	RNA 3'-terminal phosphate cyclase
AN7772.3	Cytochrome P450
AN7774.3	
AN7776.3	Fungal Zn(2)-Cys(6) binuclear cluster domain
AN7778.3	
AN7779.3	Major facilitator superfamily
AN7781.3	
AN7792.3	Lysophospholipase catalytic domain
AN7796.3	Major facilitator superfamily
AN7800.3	MirA
AN7804.3	StcW
AN7805.3	StcV
AN7806.3	StcU
AN7807.3	StcT
AN7808.3	StcS
AN7810.3	StcQ
AN7811.3	StcO
AN7814.3	StcK

AN7815.3	StcJ
AN7816.3	StcI
AN7818.3	StcF
AN7820.3	AflR
AN7821.3	StcE
AN7822.3	
AN7823.3	StcC
AN7824.3	StcB
AN7825.3	StcA
AN7834.3	
AN7836.3	
AN7838.3	Acyl transferase domain
AN7839.3	ABC transporter
AN7856.3	
AN7863.3	
AN7864.3	
AN7870.3	WSC domain
AN7873.3	Acyl transferase domain
AN7877.3	

AN7878.3	Aminotransferase class IV
AN7890.3	Glucose-methanol-choline oxidoreductase
AN7892.3	Hsp20/alpha crystallin family
AN7893.3	2OG-Fe(II) oxygenase superfamily
AN7894.3	YCII-related domain
AN7895.3	
AN7914.3	alcohol dehydrogenase
AN7917.3	FAD dependent oxidoreductase
AN7936.3	Major facilitator superfamily
AN7939.3	
AN7941.3	
AN7943.3	Arylacetamide deacetylase
AN7952.3	Hydantoinase/oxoprolinase
AN7953.3	2OG-Fe(II) oxygenase superfamily
AN7960.3	
AN7968.3	
AN7987.3	
AN7989.3	Phosphatidylserine decarboxylase
AN7990.3	

AN7996.3	
AN7999.3	short chain dehydrogenase
AN8004.3	Cytochrome P450
AN8006.3	Fungal hydrophobin
AN8007.3	Glycoside hydrolase, family 43
AN8010.3	Glycogen synthase
AN8016.3	Fal1
AN8018.3	
AN8019.3	Ank repeat
AN8036.3	Aldo/keto reductase family proteins
AN8041.3	G3P
AN8043.3	
AN8049.3	LbsA
AN8057.3	CysK
AN8060.3	
AN8061.3	Cwc27
AN8068.3	Cellulase (glycosyl hydrolase family 5)
AN8072.3	
AN8074.3	MORN motif, unknown function

AN8081.3	
AN8084.3	Sugar (ANd other) transporter
AN8085.3	Ank repeat
AN8089.3	
AN8095.3	Major facilitator superfamily
AN8100.3	Sodium/hydrogen exchanger family
AN8106.3	
AN8116.3	
AN8118.3	Cytochrome c oxidase, subunit Va
AN8122.3	Major facilitator superfamily
AN8123.3	
AN8124.3	
AN8130.3	Alanine dehydrogenase/PNT, N-terminal
AN8138.3	Glycoside hydrolase, family 36 (melibiase)
AN8139.3	
AN8145.3	
AN8146.3	
AN8148.3	
AN8149.3	Extracellular protein with glycoside transferase motif

AN8152.3	FAD binding domain
AN8153.3	ATPases associated with various cellular activities (AAA)
AN8157.3	
AN8161.3	
AN8163.3	short chain dehydrogenase
AN8166.3	
AN8167.3	
AN8175.3	beta-1,6-N-acetylglucosaminyltransferase
AN8180.3	Prp45
AN8186.3	
AN8188.3	
AN8190.3	Protein kinase
AN8193.3	
AN8202.3	
AN8205.3	
AN8209.3	Wa
AN8218.3	
AN8222.3	
AN8227.3	

AN8233.3	Sfh5
AN8234.3	
AN8235.3	Ribosomal protein L35
AN8237.3	
AN8241.3	Glycosyl hydrolases family 18
AN8242.3	
AN8256.3	Ribosomal protein L27
AN8262.3	
AN8265.3	
AN8270.3	Myb, DNA-binding
AN8274.3	Mitochondrial carrier proteins
AN8275.3	CisY
AN8277.3	CysD
AN8282.3	WD domain, G-beta repeat
AN8290.3	
AN8291.3	Vesicle coat complex AP-3, delta subunit
AN8304.3	Ank repeat
AN8308.3	
AN8311.3	

AN8314.3	FG-GAP repeat
AN8323.3	
AN8326.3	
AN8327.3	Glycoside hydrolase, family 28 (Polygalacturonase)
AN8333.3	
AN8337.3	ZIP Zinc transporter
AN8344.3	ABC transporter
AN8347.3	Sugar (ANd other) transporter
AN8349.3	
AN8351.3	Flavin-containing monooxygenase
AN8356.3	Zinc-containing alcohol dehydrogenase
AN8361.3	HPr serine phosphorylation site
AN8362.3	
AN8366.3	
AN8368.3	
AN8370.3	
AN8375.3	
AN8376.3	
AN8379.3	

AN8384.3	
AN8390.3	
AN8392.3	
AN8399.3	Cation transporting ATPase, C-terminus
AN8400.3	Sugar transporter superfamily
AN8406.3	Zinc-containing alcohol dehydrogenase
AN8407.3	
AN8414.3	Fungal Zn(2)-Cys(6) binuclear cluster domain
AN8415.3	Acyl-CoA dehydrogenase,
AN8420.3	Heme-binding domain in cytochrome b5 and oxidoreductases
AN8429.3	
AN8430.3	Kelch motif
AN8431.3	Fungal transcriptional regulatory protein, N-terminal
AN8432.3	
AN8433.3	AMP-binding enzyme
AN8434.3	Ank repeat
AN8435.3	Common central domain of tyrosinase
AN8438.3	
AN8439.3	

AN8445.3	PA domain
AN8451.3	
AN8453.3	Pectate lyase
AN8458.3	
AN8478.3	
AN8479.3	
AN8483.3	
AN8485.3	Dre2
AN8488.3	SNARE protein
AN8489.3	ABC transporter
AN8490.3	
AN8498.3	
AN8502.3	Major facilitator superfamily
AN8503.3	
AN8505.3	WD domain, G-beta repeat
AN8509.3	
AN8515.3	
AN8522.3	
AN8523.3	

AN8532.3	
AN8536.3	
AN8537.3	Flavin-containing monooxygenase
AN8540.3	MirB
AN8541.3	
AN8542.3	
AN8544.3	
AN8547.3	GMC oxidoreductase
AN8548.3	
AN8553.3	Catalase
AN8557.3	
AN8559.3	Transketolase, C-terminal domain
AN8561.3	short chain dehydrogenase
AN8562.3	Ank repeat
AN8565.3	Homoserine acetyltransferase
AN8567.3	
AN8568.3	
AN8580.3	
AN8587.3	FMN-dependent alpha-hydroxy acid dehydrogenase

AN8590.3	Fungal Zn(2)-Cys(6) binuclear cluster domain
AN8591.3	Nucleoside diphosphate kinase
AN8599.3	
AN8601.3	
AN8602.3	
AN8604.3	
AN8609.3	
AN8610.3	
AN8611.3	
AN8612.3	
AN8616.3	
AN8618.3	
AN8622.3	Alkaline phosphatase
AN8624.3	
AN8637.3	CatA
AN8638.3	Domain of Unknown function
AN8639.3	Glycosyltransferase family 20
AN8640.3	
AN8641.3	

AN8642.3	Oxidoreductase family
AN8649.3	
AN8661.3	
AN8666.3	Fungal transcriptional regulatory protein, N-terminal
AN8667.3	AreA
AN8668.3	Ipi1
AN8670.3	
AN8683.3	Ferric reductase like transmembrane component
AN8701.3	WD domain, G-beta repeat
AN8709.3	Aspartate/other aminotransferase
AN8712.3	Translation initiation factor 1A
AN8714.3	
AN8721.3	U383
AN8722.3	Sub2
AN8726.3	Amino acid permease
AN8727.3	
AN8730.3	
AN8739.3	
AN8744.3	FMN-dependent dehydrogenase

AN8748.3	Apth1
AN8751.3	Protein kinase
AN8752.3	
AN8754.3	Asparagine synthase
AN8756.3	FAD binding domain
AN8767.3	Ankyrin
AN8768.3	
AN8777.3	AmdS
AN8781.3	
AN8783.3	BimB
AN8790.3	
AN8801.3	2-nitropropane dioxygenase
AN8803.3	Fungal hydrophobin
AN8806.3	
AN8814.3	Major facilitator superfamily
AN8816.3	Amino acid permease
AN8820.3	Pp2B
AN8825.3	Set2
AN8829.3	

AN8833.3	
AN8850.3	Fyv10
AN8851.3	Cbf5
AN8865.3	Bur1
AN8888.3	
AN8889.3	General substrate transporter
AN8890.3	
AN8891.3	Glycoside hydrolase, family 28
AN8896.3	short chain dehydrogenase
AN8899.3	
AN8902.3	Fungal specific transcription factor domain
AN8903.3	TGF-beta receptor
AN8905.3	Cytochrome P450
AN8907.3	Sterol desaturase
AN8908.3	
AN8915.3	H+/oligopeptide symporter
AN8917.3	
AN8924.3	
AN8928.3	ABC transporter

AN8936.3	Major facilitator superfamily
AN8938.3	
AN8940.3	
AN8943.3	
AN8945.3	
AN8953.3	Glycosyl hydrolases family 31
AN8958.3	
AN8959.3	
AN8962.3	Predicted K+/H+-antiporter
AN8963.3	
AN8964.3	FAD binding domain
AN8967.3	FAD-binding protein DIMINUTO
AN8968.3	Isoflavone reductase
AN8969.3	
AN8972.3	
AN8974.3	
AN8977.3	
AN8978.3	AlcR
AN8979.3	Adh1

AN8980.3	
AN8981.3	
AN8982.3	FAD-dependent oxidoreductase
AN8983.3	Synaptic vesicle transporter SVOP
AN8986.3	
AN8989.3	
AN8990.3	
AN8994.3	
AN8998.3	Dioxygenase
AN9001.3	
AN9004.3	
AN9006.3	
AN9007.3	Cytochrome P450
AN9011.3	
AN9019.3	
AN9023.3	Ferric reductase, NADH/NADPH oxidase and related proteins
AN9024.3	
AN9025.3	Fungal Zn(2)-Cys(6) binuclear cluster domain
AN9028.3	

AN9034.3	Related to aldehyde dehydrogenase
AN9035.3	Melibiase
AN9037.3	NarB
AN9048.3	His Kinase A (phosphoacceptor) domain
AN9054.3	
AN9060.3	Zn-finger, C2H2 type
AN9062.3	F1-ATP synthase assembly protein
AN9063.3	Ank repeat
AN9065.3	
AN9066.3	FAD binding domain
AN9069.3	
AN9071.3	
AN9077.3	Swr1
AN9081.3	AMP-binding enzyme
AN9095.3	Ppil4
AN9103.3	Pyridine nucleotide-disulphide oxidoreductase, class I
AN9121.3	
AN9123.3	
AN9127.3	short chain dehydrogenase

AN9128.3	Enoyl-CoA hydratase/isomerase family
AN9129.3	AMP-binding enzyme
AN9130.3	Esterase/lipase/thioesterase
AN9137.3	
AN9138.3	Amidases
AN9141.3	
AN9148.3	UTP--glucose-1-phosphate uridylyltransferase
AN9156.3	
AN9162.3	Acyl-CoA dehydrogenase
AN9165.3	Major facilitator superfamily
AN9171.3	
AN9173.3	Sugar transporter
AN9174.3	Amino acid permease
AN9180.3	
AN9184.3	Sugar (ANd other) transporter
AN9194.3	
AN9203.3	putative extracellular tannase and feruloyl esterase
AN9205.3	
AN9213.3	Fibronectin type III domain

AN9246.3	Putative fumonisin B2 gene in niger
AN9247.3	
AN9248.3	
AN9250.3	
AN9251.3	
AN9262.3	
AN9263.3	
AN9285.3	
AN9286.3	Glycosyl hydrolase family 67
AN9287.3	Lipase/Acylhydrolase with GDSL-like motif
AN9288.3	
AN9289.3	
AN9292.3	
AN9295.3	Sugar (ANd other) transporter
AN9296.3	Cytochrome P450
AN9297.3	
AN9298.3	
AN9300.3	
AN9302.3	

AN9305.3	
AN9306.3	
AN9313.3	Cytochrome P450
AN9314.3	
AN9317.3	
AN9320.3	
AN9323.3	
AN9332.3	
AN9333.3	Major facilitator superfamily
AN9338.3	Endoribonuclease L-PSP
AN9339.3	CatB
AN9340.3	TreA
AN9343.3	
AN9351.3	
AN9355.3	
AN9363.3	Dioxygenase
AN9364.3	
AN9365.3	XynB
AN9370.3	Major facilitator superfamily

AN9373.3	
AN9375.3	
AN9377.3	GTPase
AN9380.3	Polysaccharide deacetylase
AN9386.3	
AN9388.3	
AN9390.3	Chitinase
AN9391.3	
AN9392.3	
AN9399.3	Calcium-binding EF-hand
AN9400.3	Amidase
AN9402.3	Metallophosphoesterase
AN9407.3	Beta-ketoacyl synthase
AN9419.3	Sya
AN9438.3	Lcmt1
AN9441.3	
AN9444.3	
AN9446.3	
AN9452.3	Rms5

AN9458.3	Fungal specific transcription factor
AN9459.3	
AN9470.3	UriC
AN9486.3	Radical SAM superfamily
AN9490.3	Amino acid transporters
AN9502.3	
AN9503.3	
AN9504.3	NimA
AN9505.3	
AN9512.3	
AN9513.3	
AN9514.3	D-isomer specific 2-hydroxyacid dehydrogenase
AN9523.3	
