# Supplementary material for: Systems Analysis Unfolds the Relationship between the Phosphoketolase Pathway and Growth in Aspergillus nidulans
Source: PLoS One. 2008 Dec 4;3(12):e3847. doi: 10.1371/journal.pone.0003847 (PMC2585806; doi:10.1371/journal.pone.0003847)
Supplement: Figure S2 — Metabolic map of differentially regulated genes in a comparison of wild type and a phosphoketolase over-expressing (PHK) strain on xylose medium. (0.38 MB PDF) [file pone.0003847.s010.pdf]

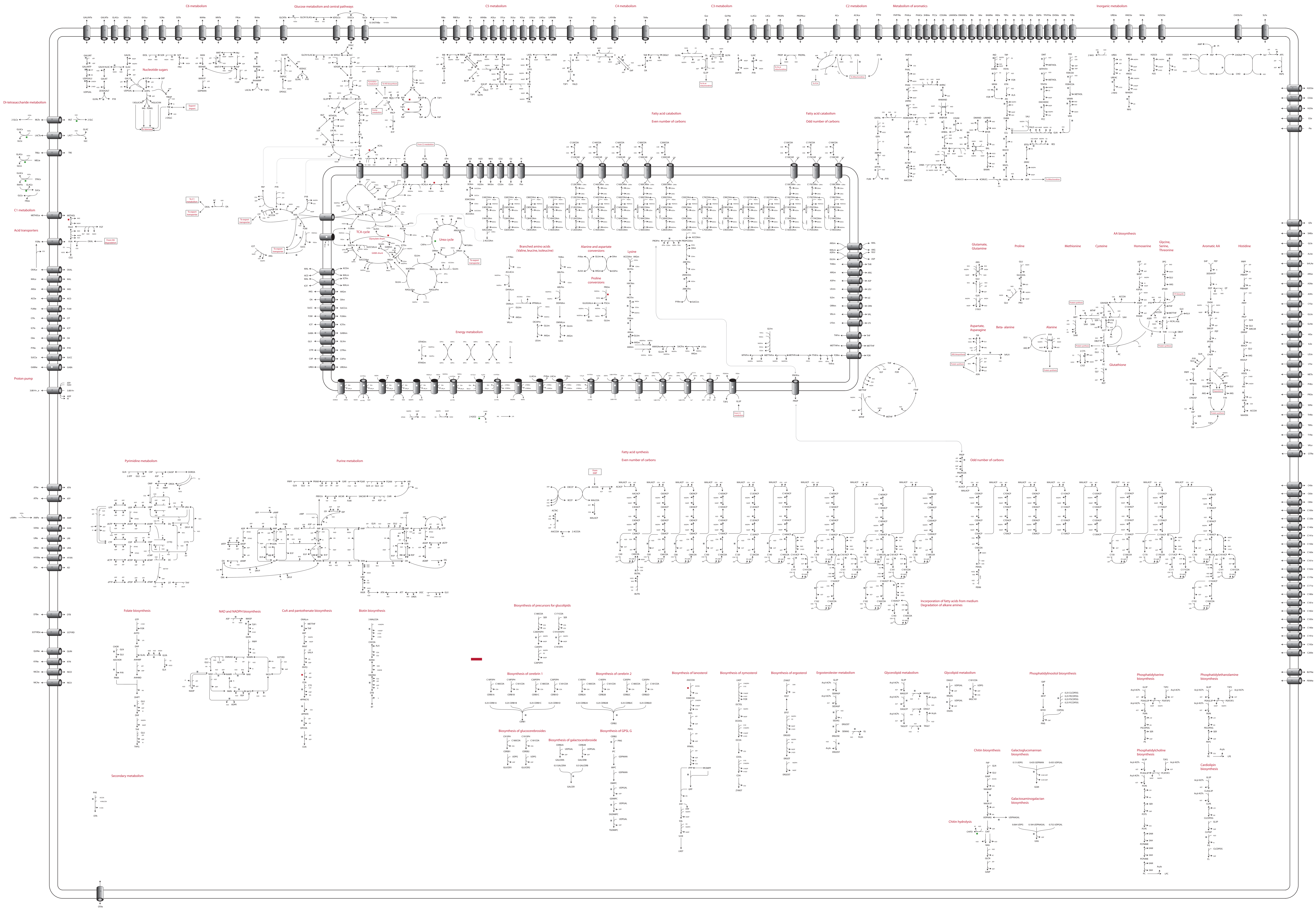
C6 metabolism
Glucose metabolism and central pathways
C5 metabolism
C4 metabolism
C3 metabolism
C2 metabolism
Metabolism of aromatics
Inorganic metabolism
Nucleotide sugars
Di-tetrasaccharide metabolism
C1 metabolism
Acid transporters
Proton pump
Fatty acid catabolism
Even number of carbons
Fatty acid catabolism
Odd number of carbons
TCA cycle
Glyoxylate shunt
GABA shunt
Urea cycle
Branched amino acids
(Valine, leucine, isoleucine)
Alanine and aspartate
conversions
Lysine
Proline
conversions
Energy metabolism
AA biosynthesis
Glutamate,
Glutamine
Proline
Methionine
Cysteine
Homoserine
Glycine,
Serine,
Threonine
Aromatic AA
Histidine
Aspartate,
Asparagine
Beta- alanine
Alanine
Glutathione
Pyrimidine metabolism
Purine metabolism
Fatty acid synthesis
Even number of carbons
Odd number of carbons
Incorporation of fatty acids from medium
Degradation of alkane amines
Folate biosynthesis
NAD and NADPH biosynthesis
CoA and pantothenate biosynthesis
Biotin biosynthesis
Biosynthesis of precursors for glucolipids
Biosynthesis of cerebrin 1
Biosynthesis of cerebrin 2
Biosynthesis of lanosterol
Biosynthesis of zymosterol
Biosynthesis of ergosterol
Ergosterol ester metabolism
Glycerolipid metabolism
Glycolipid metabolism
Phosphatidylinositol biosynthesis
Phosphatidylserine
biosynthesis
Phosphatidylethanolamine
biosynthesis
Biosynthesis of glucocerebrosides
Biosynthesis of galactocerebroside
Biosynthesis of GPSL G
Chitin biosynthesis
Galactoglucomannan
biosynthesis
Phosphatidylcholine
biosynthesis
Cardiolipin
biosynthesis
Galactosaminogalactan
biosynthesis
Chitin hydrolysis
Secondary metabolism
